# Correction: Functional and structural segregation of overlapping helices in HIV-1

**DOI:** 10.7554/eLife.105985

**Published:** 2025-01-15

**Authors:** Maliheh Safari, Bhargavi Jayaraman, Henni Zommer, Shumin Yang, Cynthia Smith, Jason D Fernandes, Alan D Frankel

**Keywords:** Other

Safari M, Jayaraman B, Zommer H, Yang S, Smith C, Fernandes JD, Frankel AD. 2022. Functional and structural segregation of overlapping helices in HIV-1. *eLife* **11**:e72482. doi: 10.7554/eLife.72482.

Published 5 May 2022

Henni Zommer, originally acknowledged in the manuscript, performed critical assay development in support of data provided in Figure 4B. During the submission process, due to complications in communication resulting from her return to her home institute in Israel, as well as the COVID-19 pandemic, the other authors were not able to confirm Dr. Zommer’s review of the manuscript or her agreement with the findings. Dr. Zommer has recently provided communication of her agreement with the findings and we are therefore formally correcting the eLife paper to include Henni Zommer in the author list. She has reviewed the manuscript, supports the conclusion and agrees to be responsible for all the parts of the paper in its published form. All authors have agreed to the change.

The details of Henni Zommer’s contributions are as follows: Henni Zommer was instrumental in developing the Env reporter assay used in Figure 4B. Henni Zommer has reviewed the manuscript and its conclusions.

**New author list:** Maliheh Safari, Bhargavi Jayaraman, Henni Zommer, Shumin Yang, Cynthia Smith, Jason D Fernandes, Alan D Frankel

**Original author list:** Maliheh Safari, Bhargavi Jayaraman, Shumin Yang, Cynthia Smith, Jason D Fernandes, Alan D Frankel

**Details for the omitted author:**

Henni Zommer

Department of Biochemistry and Biophysics, University of California, San Francisco, San Francisco, United States

**Contribution:** Data curation, Investigation

**Competing Interests:** HZ declares no competing interests.

The article has been corrected accordingly.

