# Supplementary figures and images for: Functional and structural segregation of overlapping helices in HIV-1
Source: eLife. 2022 May 5;11:e72482. doi: 10.7554/eLife.72482 (PMC9119678; doi:10.7554/eLife.72482)

Figure 4A

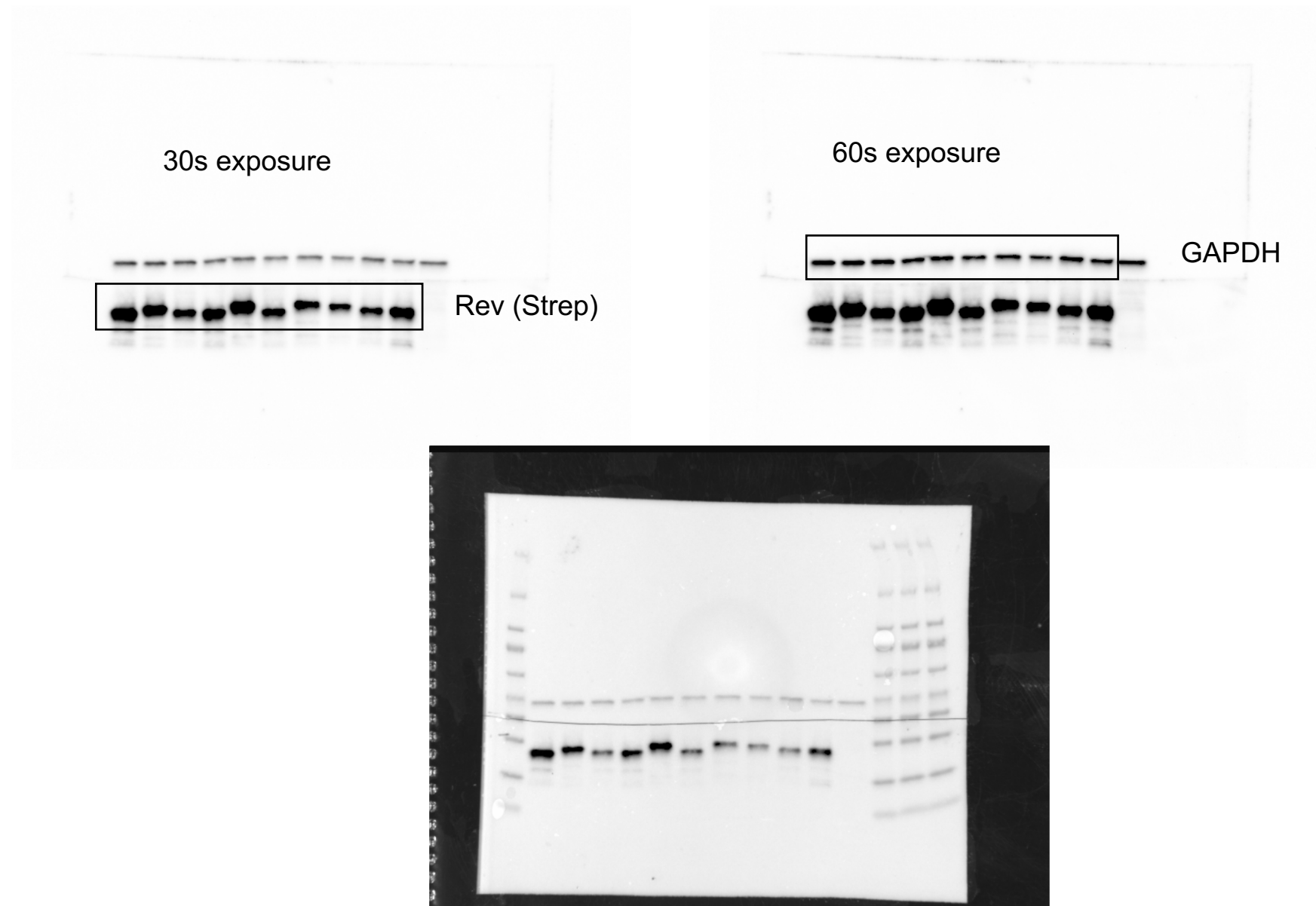

Figure 4B

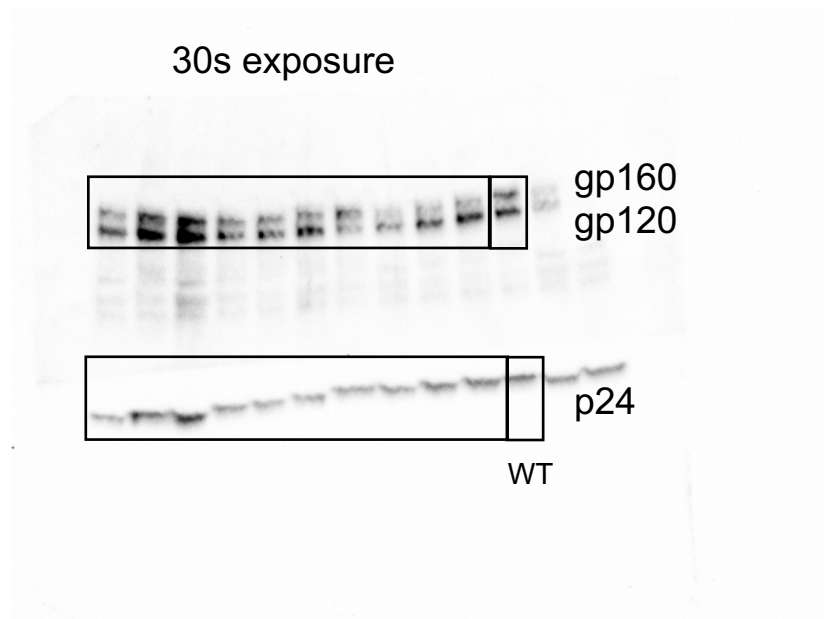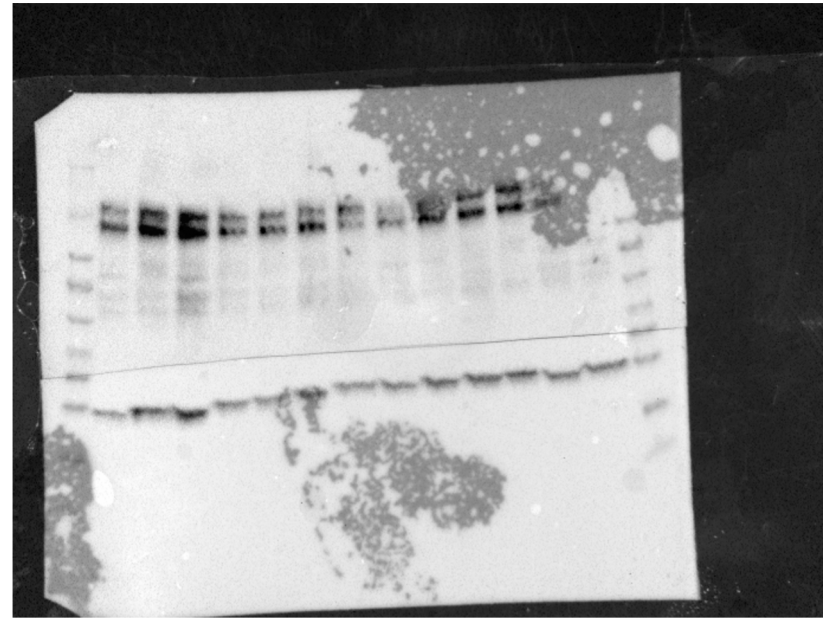

Supplement: Figure 4—source data 2. [file elife-72482-fig4-data2.pdf]

Figure 5A

gp160  
gp120

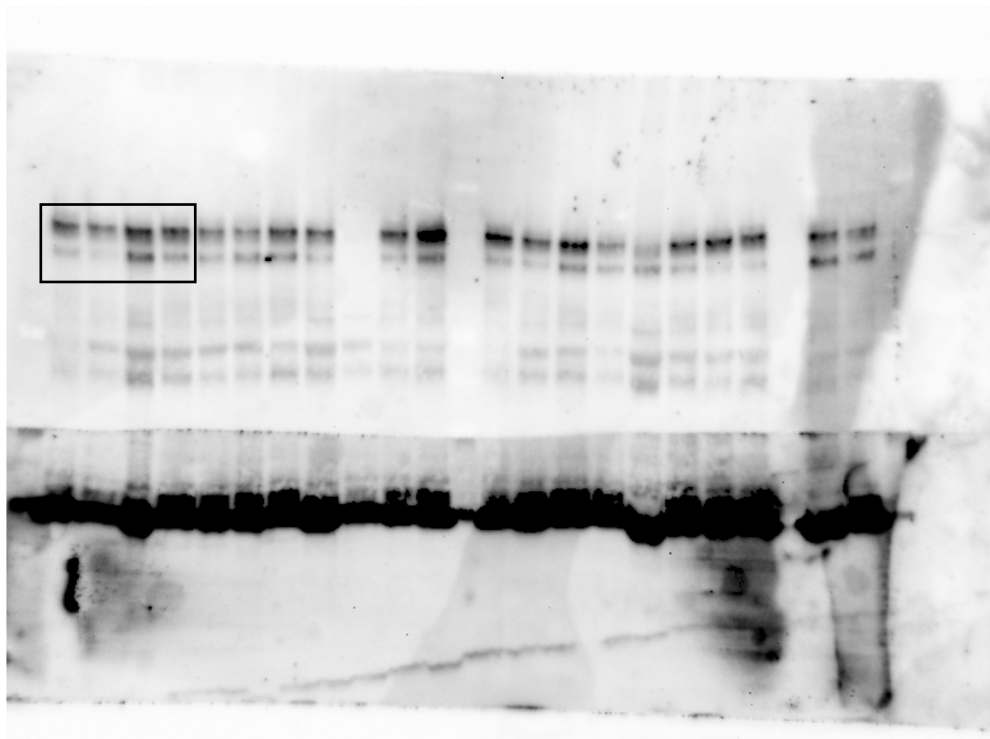

p24

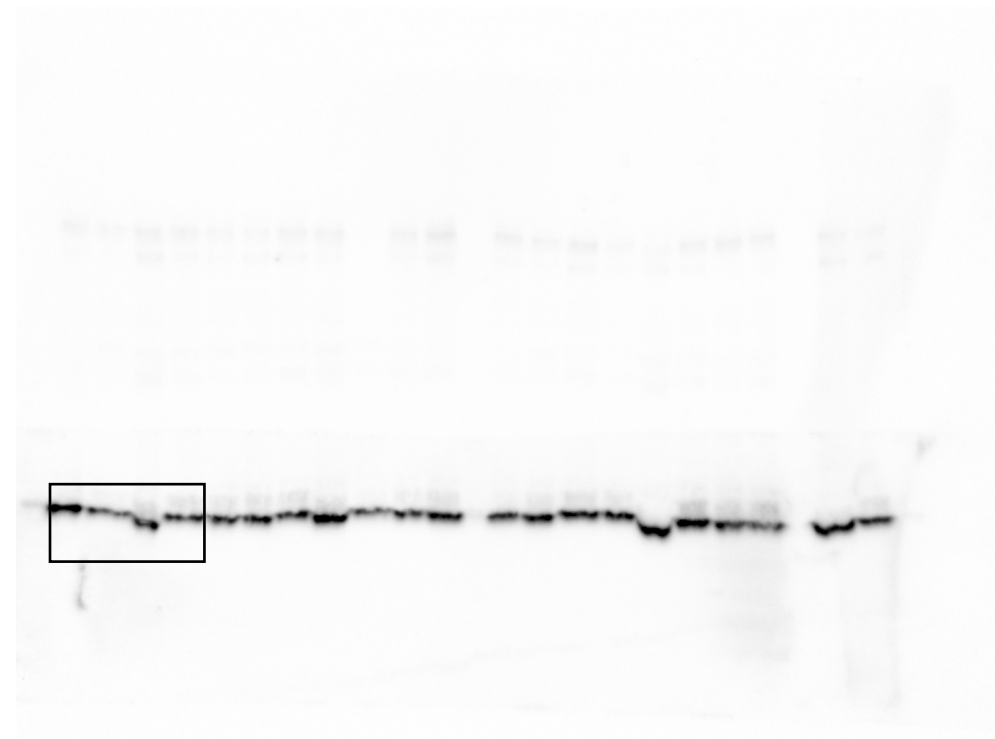

Figure 5B

gp160  
gp120

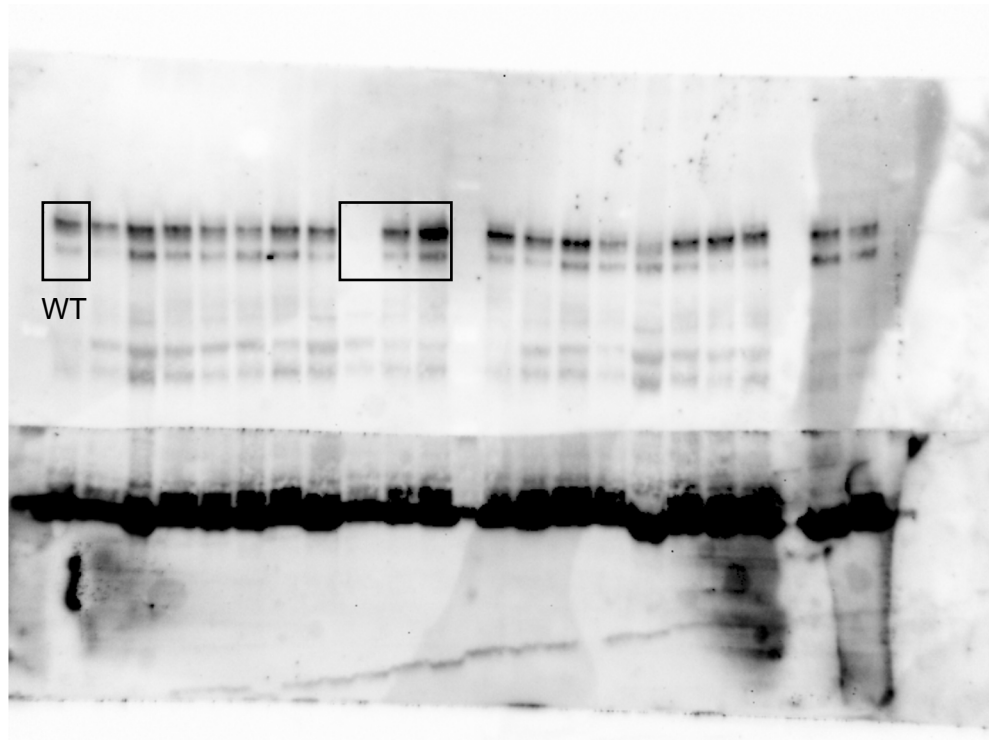

p24

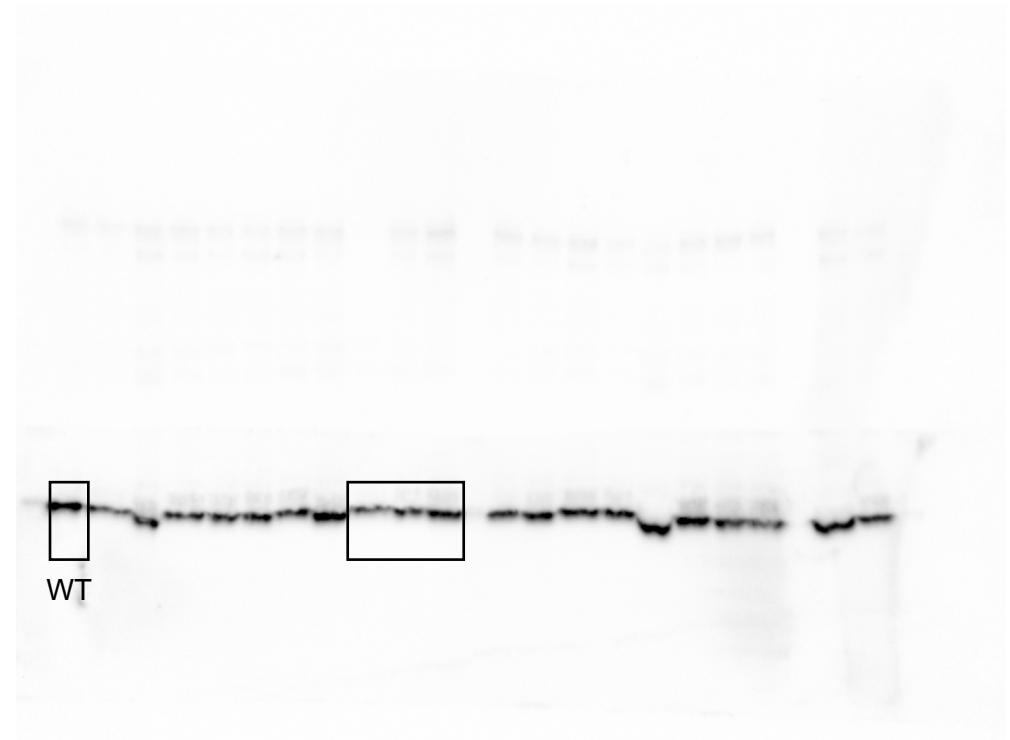

Figure 5C

gp160  
gp120

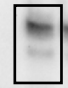

WT

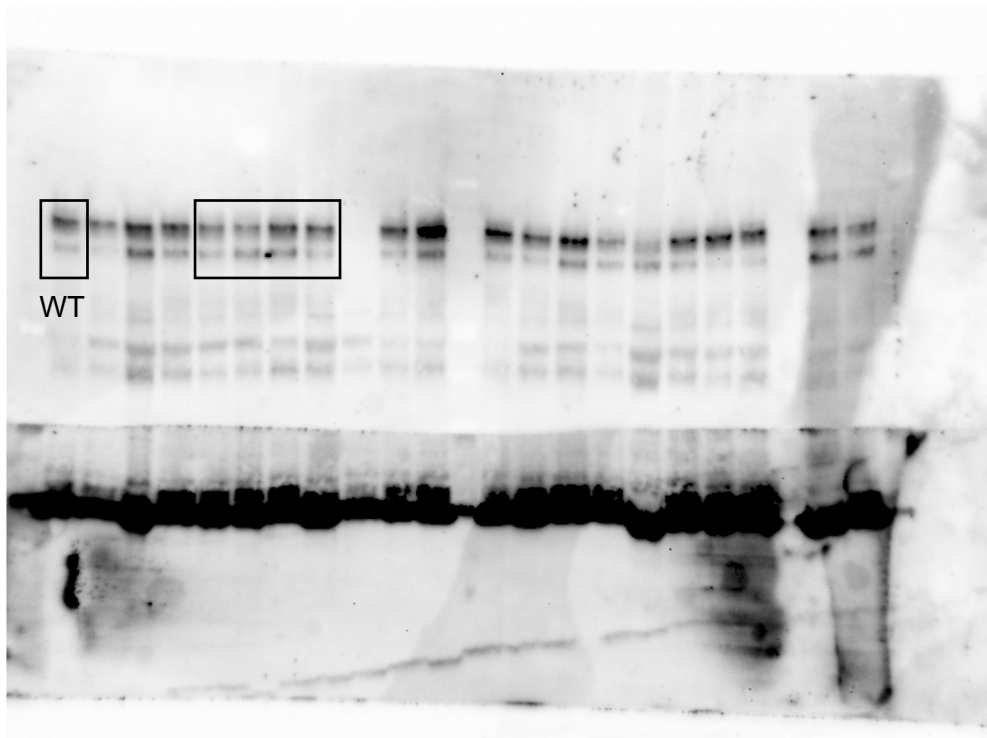

p24

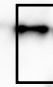

WT

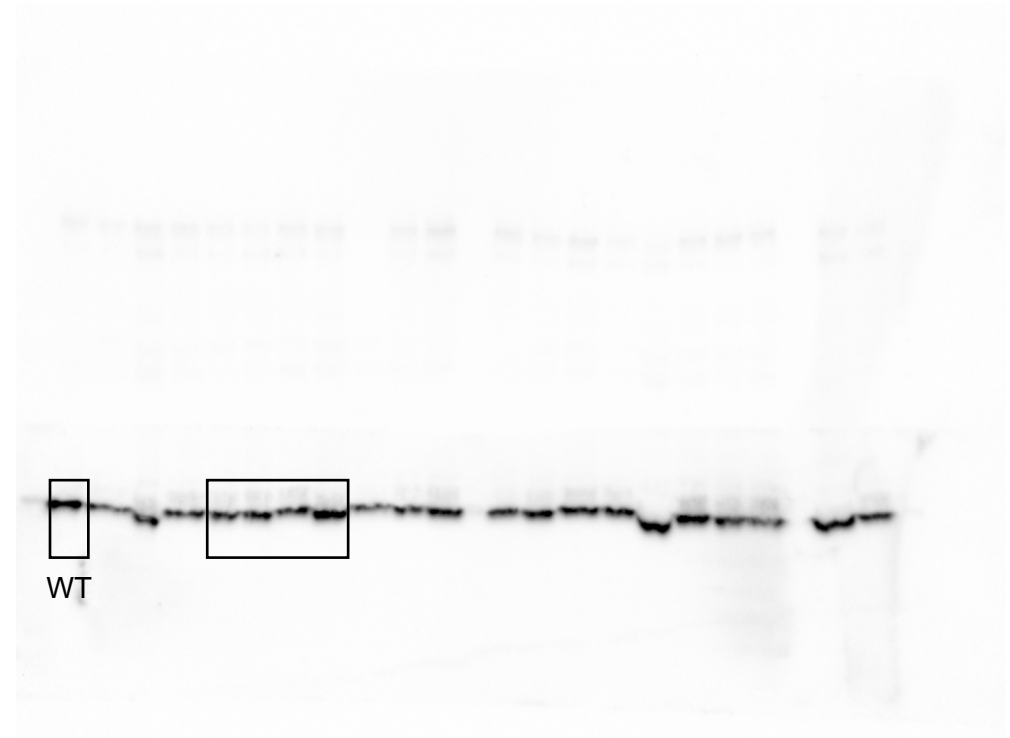

Supplement: Figure 5—source data 3. [file elife-72482-fig5-data3.pdf]

Figure 6A

gp160  
gp120

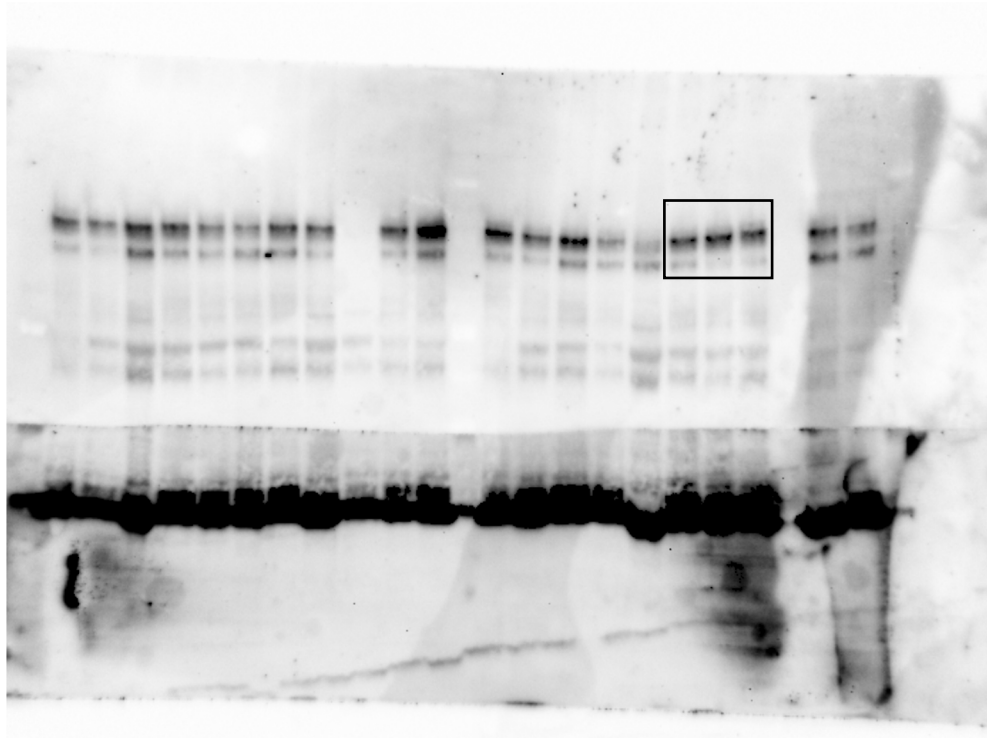

p24

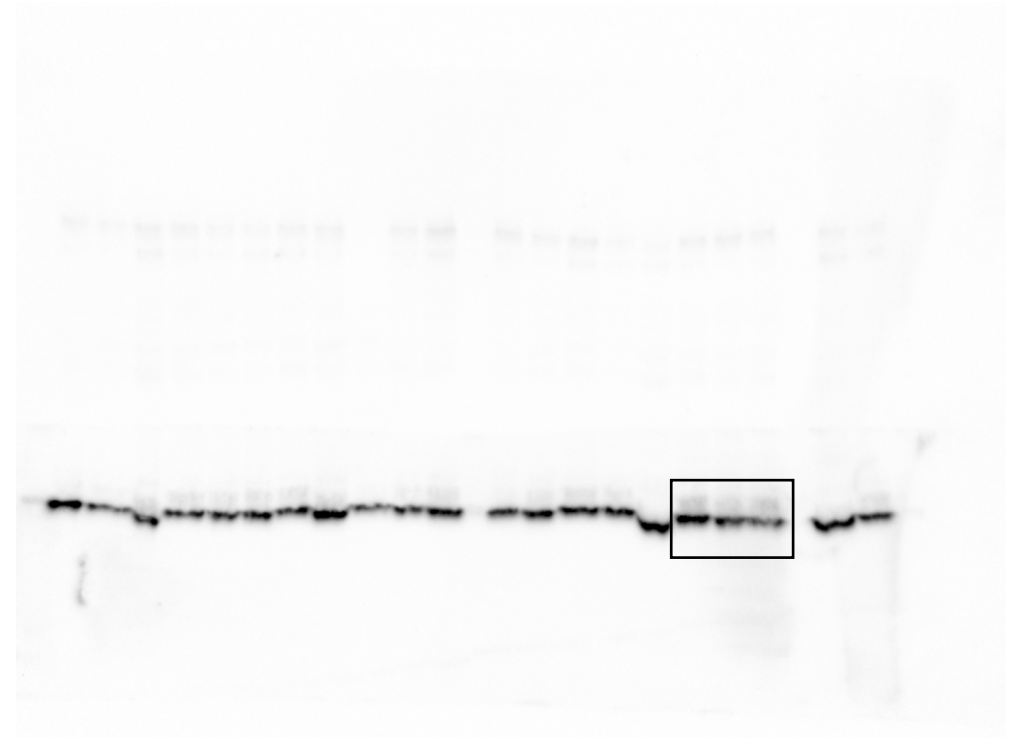

Figure 6B

gp160  
gp120

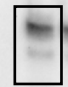

WT

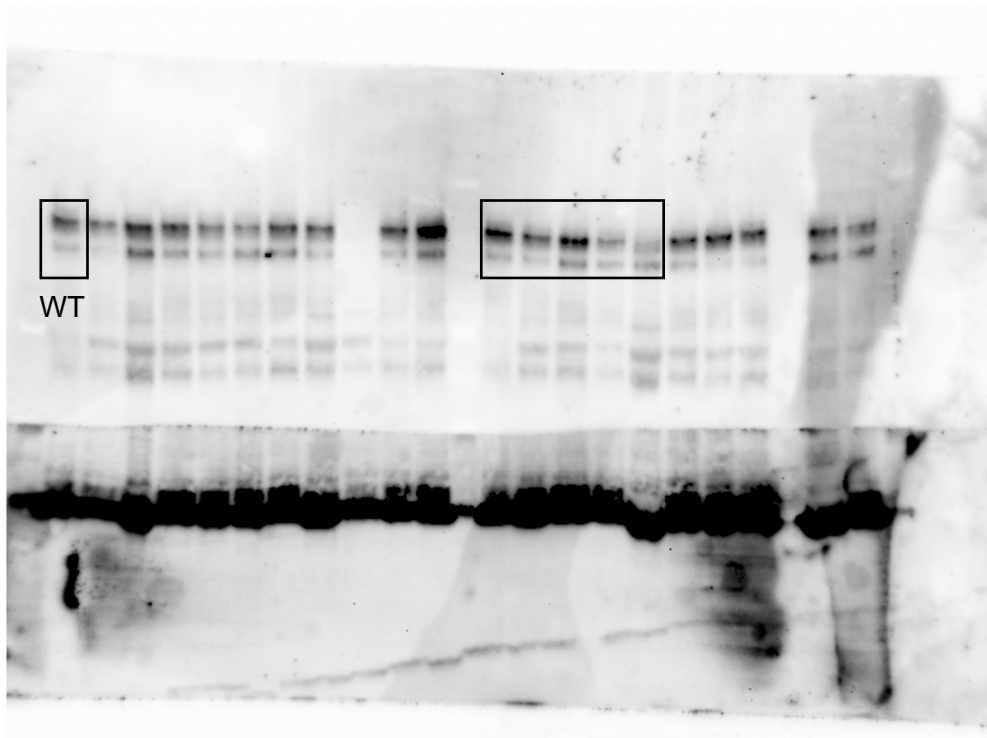

p24

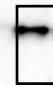

WT

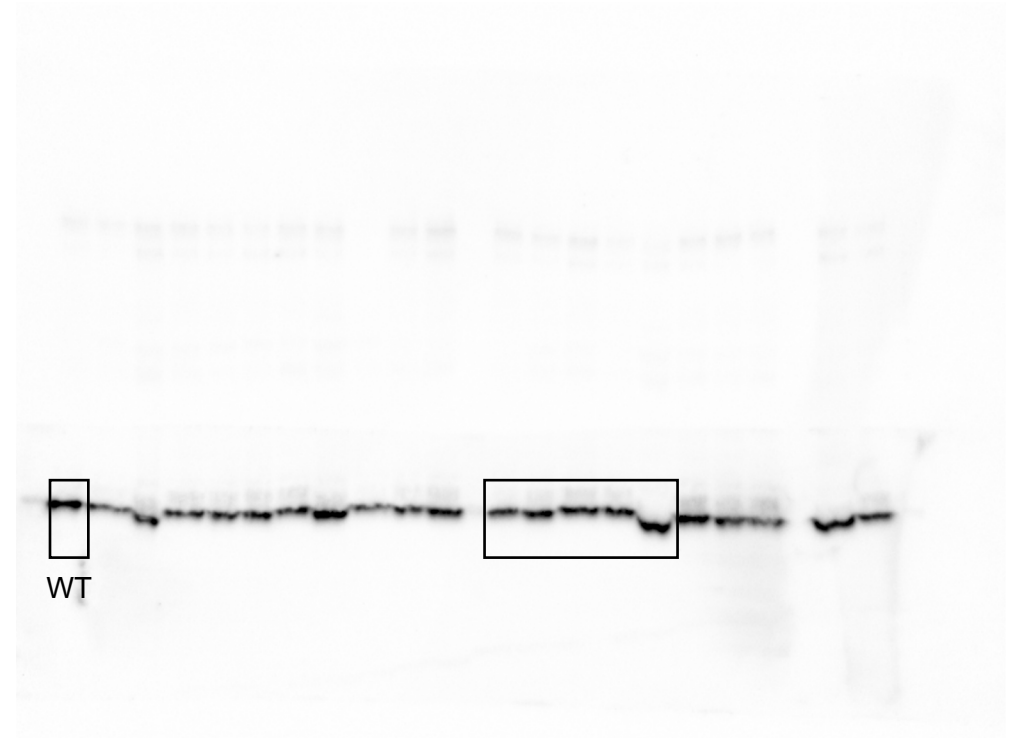

Supplement: Figure 6—source data 1. [file elife-72482-fig6-data1.pdf]
