# Supplementary material for: Functional and structural segregation of overlapping helices in HIV-1
Source: eLife. 2022 May 5;11:e72482. doi: 10.7554/eLife.72482 (PMC9119678; doi:10.7554/eLife.72482)
Supplement: Figure 8—source data 1. [file elife-72482-fig8-data1.docx]

Table of Contents

[Table x: Number of sequences used for each alignment 2](#_Toc89622241)

[Re NES alignment 2](#_Toc89622242)

[HIV-1 M Rev NES alignment 2](#_Toc89622243)

[HIV-1 N Rev NES alignment 186](#_Toc89622244)

[SIVcpzPtt Rev NES alignment 187](#_Toc89622245)

[HIV-1 P Rev NES alignment 188](#_Toc89622246)

[HIV-1 O Rev NES alignment 188](#_Toc89622247)

[SIVgor Rev NES alignment 190](#_Toc89622248)

[SIVcpzpts Rev NES alignment 190](#_Toc89622249)

[SIVmac Rev NES alignment 191](#_Toc89622250)

[HIV-2 Rev NES alignment 192](#_Toc89622251)

[SIVsmm Rev NES alignment 194](#_Toc89622252)

[SIVrcm Rev NES alignment 196](#_Toc89622253)

[Env LLP2 alignments 196](#_Toc89622254)

[HIV-1 M Env LLP2 alignment 196](#_Toc89622255)

[HIV-1 N Env LLP2 alignment 514](#_Toc89622256)

[SIVcpzPtt Env LLP2 alignment 515](#_Toc89622257)

[HIV-1 P Env LLP2 alignment 516](#_Toc89622258)

[HIV-1 O Env LLP2 alignment 516](#_Toc89622259)

[SIVgor Env LLP2 alignment 518](#_Toc89622260)

[SIVcpzpts Env LLP2 alignment 519](#_Toc89622261)

[SIVmac Env LLP2 alignment 519](#_Toc89622262)

[HIV-2 Env LLP2 alignment 521](#_Toc89622263)

[SIVsmm Env LLP2 alignment 524](#_Toc89622264)

[SIVrcm Env LLP2 alignment 526](#_Toc89622265)

# Table 1: Number of sequences used for each alignment

|  | Rev NES | Env LLP2 |
| --- | --- | --- |
| HIV-1 M | 4,054 | 7,004 |
| HIV-1 N | 9 | 11 |
| SIVcpzPtt | 12 | 15 |
| HIV-1 P | 5 | 4 |
| HIV-1 O | 48 | 48 |
| SIVgor | 6 | 8 |
| SIVcpzPts | 9 | 9 |
| SIVmac | 26 | 38 |
| HIV-2 | 49 | 75 |
| SIVsmm | 32 | 30 |
| SIVrcm | 6 | 7 |

# Re NES alignment

## HIV-1 M Rev NES alignment

>B.FR.83.HXB2_LAI_IIIB_BRU.K03455

LPPLERLTL

>A.CD.87.2106.MH705158

LPPLERLHL

>A.CD.87.50.MH705161

LPPLERLHL

>A.CD.87.70641.MH705151

LPPLERLHL

>A.CD.87.P4039.MH705157

LPPLEGLHL

>A.CD.87.PBS6126.MH705153

LPPLERLHL

>A.CD.87.PBS888.MH705133

LPPIERLHL

>A.CH.03.HIV_CH_BID_V3538_2003.JQ403028

LPPLERLRL

>A.NG.09.09NG010499.KX389622

LPPIERLHL

>A.ZA.04.04ZASK162B1.DQ396400

LPPLERLHL

>A1.AU.04.PS1044_Day177.DQ676873

LPPIERLTL

>A1.CD.02.LA01AlPr.KU168256

LPSLERLRL

>A1.CM.03.CM54_7.KU168305

LPPLERLHL

>A1.CM.07.46_10.KP718918

LPPIERLHL

>A1.CM.08.886_24.KP718928

LPPLERLHL

>A1.CY.05.CY022.FJ388893

LPPLERLNL

>A1.CY.05.CY051.FJ388903

LPPLERLHI

>A1.CY.05.CY064.FJ388909

LPPLERLHL

>A1.CY.05.CY106.FJ388925

LPPLERLHL

>A1.CY.05.CY121.FJ388932

LPPLERLHL

>A1.CY.05.CY140.FJ388938

LPPLERLHL

>A1.CY.05.CY153.FJ388942

LPPLERLCL

>A1.CY.06.CY154.FJ388943

LPPLERLHL

>A1.CY.07.CY182.JF683737

LPPLERLHI

>A1.CY.08.CY230.JF683779

LPPLEGLHL

>A1.CY.08.CY235.JF683782

LPPIERLHL

>A1.CY.08.CY236.JF683783

LPPIERLHL

>A1.CY.09.CY243.JF683789

LPPIERLHL

>A1.ES.05.X1608_8.FJ670519

LPPLEGLHL

>A1.ES.06.X2110.FJ670523

LPPIERLCL

>A1.ES.15.100_117.KY496622

LPPIERLCL

>A1.FI.91.FIN91121.AF219261

LPPLERLHL

>A1.GB.13.15171_1_44.3.MF109677

LPPIERLHL

>A1.GB.14.14727_1_50.3.MF109613

LPPLERLNL

>A1.GB.14.14727_1_62.3.MF109623

LPPLERLNL

>A1.IN.00.NARI_FLS_VB99_30.KT152846

LPPLERLQL

>A1.IN.09.NARI_FLS_IVC19_1.KT152839

LPPLERLTL

>A1.IN.97.NARI_FLS_VB6.KT152840

LPPIERLXL

>A1.IN.99.NARI_FLS_VB11.KT152841

LPPIEGLRL

>A1.IN.99.NARI_FLS_VB15.KT152842

LPPIERLHL

>A1.IN.99.NARI_FLS_VB81_12.KT152844

LPPLERLHL

>A1.KE.00.KER2008.AF457052

LPPLERLHL

>A1.KE.00.KER2009.AF457053

LPPLEKLHL

>A1.KE.00.KNH1144.AF457066

LPSLEGLRL

>A1.KE.00.KNH1207.AF457068

LPPIERLHL

>A1.KE.00.KNH1209.AF457069

LPPLERLHL

>A1.KE.00.KNH1211.AF457070

LPPLERLNL

>A1.KE.00.KSM4024.AF457077

LPPLERLHL

>A1.KE.00.KSM4030.AF457079

LPPLERLHL

>A1.KE.00.MSA4069.AF457080

LPPIERLHL

>A1.KE.00.MSA4070.AF457081

LPPLERLHL

>A1.KE.00.MSA4072.AF457083

LPPIERLHL

>A1.KE.00.MSA4076.AF457084

LPPLERLHL

>A1.KE.00.MSA4079.AF457086

LPPLERLHL

>A1.KE.00.NKU3005.AF457089

LPPLERLHL

>A1.KE.01.ML1945.EU110088

LPPLEXLHL

>A1.KE.02.ML1990.EU110092

LPPLERLNL

>A1.KE.02.ML2014.EU110094

LPPIERLHL

>A1.KE.04.04KE169579V3.KT022360

LPPIERLRL

>A1.KE.04.04KE263806V2.KT022361

LPPLERLHI

>A1.KE.04.04KE354207V3.KT022363

PPPIERLHI

>A1.KE.04.04KE378531V2.KT022364

LPPLERLHL

>A1.KE.04.04KE406723V2.KT022365

LPPLERLHI

>A1.KE.04.04KE649309V2.KT022367

LPPLERLQL

>A1.KE.04.04KE809842V2.KT022368

LPPLERLHL

>A1.KE.04.04KE860822V3.KT022369

LPPIERLHL

>A1.KE.04.QG984_21M_ENV_A3.FJ866117

LPPLERLHL

>A1.KE.05.05KE185405V4.KT022370

LPPLERLHL

>A1.KE.05.05KE376579V4.KT022372

LPPLERLHL

>A1.KE.05.05KE520997V4.KT022373

LPPLERLHL

>A1.KE.05.05KE607907V4.KT022374

LPPIERLRL

>A1.KE.05.05KE643439V4.KT022375

LPPLERLHL

>A1.KE.05.05KE851891V4.KT022376

LPPIERLHL

>A1.KE.05.05KE884468V5.KT022377

LPPLERLHL

>A1.KE.05.QF495_23M_ENV_B2.FJ866115

LPPLERLHL

>A1.KE.05.QH209_14M_ENV_A2.FJ866118

LPPLERLHL

>A1.KE.05.QH343_21M_ENV_A10.FJ866119

LPPIERLHL

>A1.KE.05.QH359_21M_ENV_C1.FJ866121

LPPLERLRL

>A1.KE.06.06KE196199V6.KT022378

LPPIERLHL

>A1.KE.06.06KE335214V6.KT022380

LPPLERLHI

>A1.KE.06.06KE404877V7.KT022381

LPPLERLHL

>A1.KE.06.06KE452693V6.KT022382

LPPLERLHL

>A1.KE.06.06KE661996V6.KT022383

LPPIERLHL

>A1.KE.06.06KECst_001.FJ623487

LPPLERLHL

>A1.KE.06.06KECst_005.FJ623481

LPPLERLRL

>A1.KE.06.06KECst_006.FJ623475

LPPLEGLHL

>A1.KE.06.06KECst_009.FJ623480

LPPIERLRL

>A1.KE.06.06KECst_013.FJ623485

LPPLERLHI

>A1.KE.06.06KECst_016.FJ623483

LPPLERLHI

>A1.KE.06.06KECst_017.FJ623488

LPPLERLHL

>A1.KE.06.06KECst_019.FJ623478

LPPIERLHL

>A1.KE.06.06KECst_020.FJ623482

LPPLERLNI

>A1.KE.06.06KECst_021.FJ623477

LPPIERLRL

>A1.KE.06.06KECst_028.FJ623479

LPPLERLHL

>A1.KE.11.DEMA111KE002.KF716474

LPPLERLHI

>A1.KE.11.DEMA11KE001.KF716475

LPPLERLHL

>A1.KE.86.ML170_1986.AF539405

LPPLERLHL

>A1.KE.94.Q23_17.AF004885

LPPLERLHL

>A1.KE.96.QB726_70M_ENV_B3.FJ866111

LPPIERLHL

>A1.KE.97.ML752.AY322193

LPPIERLHL

>A1.KE.99.KNH1088.AF457063

LPPLERLHL

>A1.KE.99.KNH1135.AF457065

LPPIERLHL

>A1.KE.99.KSM4021.AF457075

LPPLERLHL

>A1.PK.14.PK001.KX232594

LPPLERLNI

>A1.PK.14.PK002.KX232595

LPPLERLHL

>A1.PK.14.PK004.KX232597

LPPLERLTL

>A1.PK.14.PK007.KX232600

LPPLERLHL

>A1.PK.14.PK014.KX232607

LPPLERLRL

>A1.PK.14.PK016.KX232609

LPPLERLTL

>A1.PK.14.PK017.KX232610

LPPLERLTL

>A1.PK.14.PK018.KX232611

LPPLERLTL

>A1.PK.14.PK020.KX232613

LPPLERLHL

>A1.PK.15.PK021.KX232614

LPPLERLSL

>A1.PK.15.PK026.KX232618

LPPLERLHL

>A1.PK.15.PK030.KX232620

LPPLERLHL

>A1.PK.15.PK034.KX232624

LPPLERLHL

>A1.PK.15.PK036.KX232626

LPPLERLHL

>A1.RW.06.DEMA106RW003.KU749423

LPPIERLHL

>A1.RW.07.pR463F.JX236677

LPPLERLHL

>A1.RW.07.pR880F.JX236678

LPPLERLHL

>A1.RW.08.DEMA108RW010.KU749424

LPPLERLHL

>A1.RW.11.DEMA111RW002.KF716472

LPPLERLTL

>A1.RW.92.92RW008.AB253421

LPPLERLHL

>A1.RW.92.92RW025A.AB287377

LPPLERLHL

>A1.RW.93.93RW037A.AB287379

LPPLEKLHL

>A1.RW.93.93RW_024.AY713406

LPPLEGLHL

>A1.SE.12.064GR.MF373167

LPPLERLHL

>A1.SE.12.065SE.MF373168

LPPLERLHI

>A1.SE.13.078SE.MF373181

LPPLERLSL

>A1.SE.13.079KE.MF373182

LPPLERLYL

>A1.SE.93.001UG.MF373124

LPPLERLHL

>A1.SE.94.SE7253.AF069670

LPSLETLHL

>A1.SE.94.SE7535.AF069671

LPPLERLNL

>A1.SE.95.SE8538.AF069669

LPPLEGLHL

>A1.SE.95.SE8891.AF069673

LPPIERLHL

>A1.SE.95.UGSE8131.AF107771

LPPLERLHI

>A1.TZ.01.A173.AY253305

LPPLERLHL

>A1.TZ.01.A341.AY253314

LPPIERLHL

>A1.TZ.02.CO0783V0.KX907352

LPPLERLHL

>A1.TZ.03.CO0543V2.KX907348

IPPIERLHL

>A1.TZ.04.CO0272V4.KX907336

LPPIERLHL

>A1.TZ.04.CO0330V4.KX907343

LPPLERLHL

>A1.TZ.04.CO3365V2.KX907372

LPPIERLNL

>A1.TZ.04.CO3718V3.KX907383

LPPIERLHL

>A1.TZ.04.CO3878V2.KX907389

LPQIERLHI

>A1.TZ.05.CO0260V5.KX907341

LPPLERLHL

>A1.TZ.05.CO0439V5.KX907347

LPPLERLHL

>A1.TZ.05.CO3083V4.KX907364

LPPIERLHL

>A1.TZ.05.CO6161V5.KX907401

LPPLERLHL

>A1.TZ.05.CO6592V5.KX907412

LPPIERLHL

>A1.TZ.06.CO0434V7.KX907346

LPPLERLHL

>A1.TZ.06.CO3504V7.KX907378

LPPIERLHL

>A1.TZ.06.CO6637V7.KX907414

LPPLEGLHI

>A1.TZ.06.CO6830V7.KX907423

LPPIERLHL

>A1.TZ.06.CO6974V7.KX907431

LPPLERLHL

>A1.TZ.08.DEMA108TZ002.KY658694

LPPLERLHL

>A1.TZ.08.DEMA108TZ004.KY658695

LPPIERLCL

>A1.TZ.08.DEMA108TZ012.KY658696

LPPLERLSL

>A1.TZ.97.97TZ02.AF361872

LPPIERLHL

>A1.TZ.97.97TZ03.AF361873

LPPLERLSL

>A1.UG.00.PP6_F2_B1.HM027846

LPPIERLHL

>A1.UG.07.PP6_F3_B2.HM027824

LPPIERLHL

>A1.UG.07.p191084.JX236669

LPPLERLNL

>A1.UG.07.p191845.JX236671

LPPLERLHI

>A1.UG.07.p9004SDM.JX236676

LPPIERLHL

>A1.UG.09.DEMA109UG001.KF716478

LPPIERLNL

>A1.UG.09.DEMA109UG017.KP109490

LPPLERLNL

>A1.UG.11.DEMA110UG001.KF859745

LPPLERLHL

>A1.UG.11.DEMA110UG009.KF716486

LPPLERLRI

>A1.UG.85.U455_U455A.M62320

LPPIERLRL

>A1.UG.90.UG273A.L22957

LPPLERLHL

>A1.UG.90.UG275A.L22951

LPPIERLHL

>A1.UG.92.92UG037_A40.AB253429

LPPIERLHL

>A1.UG.92.UG029.AB098332

LPPLERLNL

>A1.UG.98.98UG57134.AF484507

LPPIERLHL

>A1.UG.98.98UG57135.AF484508

LPPIERLSL

>A1.UG.98.98UG57136.AF484509

LPPIERLHI

>A1.UG.98.98UG57142.AF484512

LPPLERLHL

>A1.UG.99.99UGG03379.AF484493

LPPLERLRL

>A1.UG.x.UG031.AB098330

LPPLERLHL

>A1.ZA.00.TV096.KJ948658

LPPLERLHL

>A1.ZA.01.TV314.FJ647148

LPPLERLHI

>A1.ZA.04.503_15344_T10_A1.KT183312

LPPIERLRL

>A2.CD.87.PBS1195.MH705163

LPPLERLHL

>A2.CD.97.97CDKS10.AF286241

LPPIERLHL

>A2.CD.97.97CDKTB48.AF286238

LPPLERLHL

>A2.CM.01.01CM_1445MV.GU201516

LPPLERLHI

>A2.CY.94.94CY017_41.AF286237

LPPLERLHI

>A3.SN.01.DDJ369.AY521631

LPPLEGLHI

>A4.CD.02.02CD_KTB035.AM000055

LPPIERLHL

>A4.CD.97.97CD_KCC2.AM000053

LPPLERLHL

>A4.CD.97.97CD_KTB13.AM000054

LPPLERLHI

>A6.BY.13.PV85.KT983615

LPPIERLNL

>A6.CY.05.CY021.FJ388892

LPPIERLHL

>A6.CY.06.CY171.FJ388950

LPPIDRLHL

>A6.CY.06.CY173.FJ388951

LPPIERLHL

>A6.CY.07.CY213.JF683763

LPPIERLHL

>A6.CY.09.CY255.JF683798

LPPIERLNL

>A6.IT.02.60000.EU861977

LPPLERLRL

>A6.RU.00.RU00051.EF545108

LPPLERLHL

>A6.RU.05.RU_560_1125_JA.JQ292895

LPPIERLHL

>A6.RU.06.RU_915_1016.JQ292896

LPPIERLHL

>A6.RU.06.RU_915_1035.JQ292897

LPPIERLHL

>A6.RU.06.RU_915_1038.JQ292898

LPPIERLHL

>A6.RU.06.RU_915_1041.JQ292899

LPPIERLHL

>A6.RU.06.RU_SP_B_049.JQ292900

LPPIERLHL

>A6.RU.07.Irkutsk_5.JQ292891

LPPIERLHL

>A6.RU.08.DEMA108RU003.KF716491

LPPIERLNL

>A6.RU.08.DEMA108RU004.KF716492

LPPLERLHL

>A6.RU.08.MSK_SK_006_2.KY238327

LPPIERLHL

>A6.RU.08.RUA001.JQ292893

LPPIERLHL

>A6.RU.08.RUA007.JQ292894

LPXIERLHL

>A6.RU.10.10RU6617.JX500696

LPPIERLHL

>A6.RU.10.10RU6792.JX500695

LPPIERLNL

>A6.RU.11.11RU6950.JX500694

LPPIERLHL

>A6.RU.13.ARH001.MG902950

LPPIERLHL

>A6.RU.13.ARH011.MG902951

LPPIERLHL

>A6.UA.01.01UAOD35.DQ823366

LPPIERLHL

>A6.UA.11.DEMA111UA002.KU749399

LPPLERLHL

>A6.UA.11.DEMA111UA008.KU749400

LPPIERLRL

>A6.UA.11.DEMA111UA009.KU749401

LPPIERLHL

>A6.UA.12.DEMA112UA013.KY658681

LPPIERLHL

>A6.UA.12.DEMA112UA014.KU749402

LPPIERLHL

>A6.UA.12.DEMA112UA024.KU749403

LPPIERLHL

>A6.UA.12.DEMA112UA030.KU749404

LPPIERLHL

>A6.UA.12.DEMA112UA034.KU749405

LPPIERLHL

>A6.UA.12.DEMA112UA036.KU749406

LPPIERLHL

>A6.UA.12.DEMA112UA040.KY658682

LPPIERLHL

>A6.UA.12.DEMA112UA042.KU749407

LPPIEGLHL

>A6.UA.97.ukr970063.AF082486

LPPIERLHL

>A6.UZ.02.02UZ694.AY829205

LPPIERLHL

>B.AR.00.ARMS008.AY037269

LPPLERLNL

>B.AR.02.02AR114146.DQ383746

LPSLERLTL

>B.AR.03.03AR137681.DQ383748

LPPLERLTL

>B.AR.03.03AR138910.DQ383749

LPPLERLTL

>B.AR.04.04AR151263.DQ383751

LPPLERLTL

>B.AR.04.04AR151516.DQ383752

LPPLERLTL

>B.AR.07.DEURF07AR001.KY658686

LPPLERLTL

>B.AR.08.DEMB08AR002.KY658683

LPPIERLTL

>B.AR.09.DEMB09AR010.MH078530

LPPLERLTL

>B.AR.10.DEMB10AR006.KY658684

LPPIERLTL

>B.AR.14.DEMB14AR003.KY658685

LPPLERLTL

>B.AR.14.DEMB14AR012.MH078531

LPPIERLNL

>B.AR.15.DEMB15AR013.MH078532

LPSIERLTL

>B.AR.15.DEMB15AR014.MH078533

LPPLERLSL

>B.AR.98.ARCH054.AY037268

LPPLERLTL

>B.AR.99.ARMA132.AY037282

LPPLERLTL

>B.AU.03.PS2008_Day182.DQ676875

LPPLERLTL

>B.AU.03.PS2019_Day171.DQ676881

LPPLERLSL

>B.AU.03.PS3002_Day385.DQ676885

LPPLERLTL

>B.AU.03.PS4048_Day0.DQ676886

LPPLERLTL

>B.AU.04.MS2004_37_012.EF178310

LPPLERLTL

>B.AU.04.MS2004_37_016.EF178314

LPPLDRLTL

>B.AU.04.MS2004_37_040.EF178338

LPPIERLTL

>B.AU.04.MS2004_37_056.EF178354

LPPLERLTL

>B.AU.04.MS2004_37_060.EF178358

LPPLERLTL

>B.AU.04.MS2004_37_076.EF178374

LPPLERLTL

>B.AU.04.MS2004_37_122.EF178420

LPPLERLTI

>B.AU.04.PS1038_Day174.DQ676871

LPPLERLTL

>B.AU.04.PS2016_Day380.DQ676879

LPPLERLTL

>B.AU.04.Phcsffull04.AY818644

LPPLERLTL

>B.AU.86.MBC200.AF042100

LPPLERLTL

>B.AU.87.MBC925.AF042101

LPPLERLTL

>B.AU.93.MBC18_MBCC18.AF042102

LPPLERLTL

>B.AU.95.C24.AF538304

LPPLERLTL

>B.AU.95.C42.AF538305

LPPLERLTL

>B.AU.95.C76.AF538306

LPPLERLTL

>B.AU.95.C92.AF538307

LPPLERLTL

>B.AU.95.MBCC54.AF042103

LPPLERLTL

>B.AU.96.MBCC98.AF042104

LPPLERLTL

>B.AU.96.MBCD36.AF042105

LPPLERLTL

>B.AU.99.1181.AF538302

LPPLERLTL

>B.AU.x.15888_30.AY624304

LPPLERLTL

>B.AU.x.2870718.AY857022

LPPLERLTL

>B.AU.x.4675282.AY857052

LPPLERLTL

>B.AU.x.7894552.AY857127

LPPIERLTL

>B.AU.x.8634991.AY857144

LPPLERLTL

>B.AU.x.9125091.AY857165

LPPLERLTL

>B.AU.x.VH_VHPCR.AF146728

LPPLERLTL

>B.BO.99.BOL0122.AY037270

LPPLERLTL

>B.BR.02.02BR002.DQ358805

LPPIERLTL

>B.BR.02.02BR008.DQ358808

LPPLERLTL

>B.BR.02.02BR011.DQ358809

LPPLERLTL

>B.BR.02.02BR013.DQ358810

LPPLERLTL

>B.BR.02.02BR1013.JN692432

LPPLDRLTL

>B.BR.02.02BR2025.JN692435

LPPLERLTL

>B.BR.02.02BR2032.JN692439

LPSFERLTL

>B.BR.02.02BR2033.JN692440

LPPLERLTL

>B.BR.02.02BR2041.JN692443

LPPLERLTL

>B.BR.02.02BR2042.JN692444

LPPLERLTL

>B.BR.02.04BR1064.JN692433

LPPLERLTL

>B.BR.03.03BR1020.JN692445

LPPLERLTL

>B.BR.03.03BR1046.JN692447

LPPLERLTL

>B.BR.03.BREPM1023.EF637057

LPPLGRLTP

>B.BR.03.BREPM1024.EF637056

LPPLERLTL

>B.BR.03.BREPM1027.EF637054

LPPLEKLTL

>B.BR.03.BREPM1028.EF637053

LPPLERLTL

>B.BR.03.BREPM1032.EF637051

LPPLERLTL

>B.BR.03.BREPM1033.EF637050

LPPIERLTL

>B.BR.03.BREPM1035.EF637049

LPPLERLTL

>B.BR.03.BREPM1038.EF637048

LPPLERLTL

>B.BR.03.BREPM1040.EF637047

LPSLERLTL

>B.BR.03.BREPM2012.EF637046

LPPLERLTL

>B.BR.04.04BR1047.JN692450

LPPLERLTL

>B.BR.04.04BR1049.JN692451

LPPLERLTL

>B.BR.04.04BR1051.JN692452

LPPLERLTL

>B.BR.04.04BR1054.JN692453

LPPLERLTL

>B.BR.04.04BR1055.JN692454

LPPLERLSL

>B.BR.04.04BR1057.JN692455

LPPLEKLTL

>B.BR.04.04BR1068.JN692457

LPPLDRLTL

>B.BR.04.BREPM1066.FJ195090

LPPLERLTL

>B.BR.04.BREPM1070.FJ195086

LPPLERLTL

>B.BR.05.05BR1074.JN692459

LPPLERLTL

>B.BR.05.05BR1077.JN692460

LPPLERLTL

>B.BR.05.05BR1078.JN692461

LPPLERLTL

>B.BR.05.05BR1079.JN692462

LPPLERLTL

>B.BR.05.05BR1080.JN692463

LPPLERLTL

>B.BR.05.05BR1082.JN692465

LPPIERLTL

>B.BR.05.05BR1089.JN692467

LPPLERLTL

>B.BR.05.05BR1092.JN692468

LPPLERLTL

>B.BR.05.05BR1095.JN692471

LPPLERLTL

>B.BR.05.05BR1101.JN692473

LPPIERLTL

>B.BR.05.05BR1104.JN692474

LPPIERLTL

>B.BR.05.05BR1107.JN692475

LPPLERLTL

>B.BR.05.BREPM1081.FJ195091

LPPLERLTL

>B.BR.05.BREPM1084.FJ195088

LPPLERLTL

>B.BR.05.BREPM1093.FJ195089

LPPLERLTL

>B.BR.06.06BR1115.JN692479

LPPLERLTL

>B.BR.06.06BR1119.JN692480

LPPLERLTL

>B.BR.07.BP00047_RH01.JN687739

LPPLERLTL

>B.BR.09.DEMB09BR040.KU749389

LPPLERLTL

>B.BR.10.10BR_MG006.KT427690

LPPLERLTL

>B.BR.10.10BR_MG007.KJ849786

LPPLERLTL

>B.BR.10.10BR_MG009.KT427689

LPQLERLTL

>B.BR.10.10BR_MG012.KT427687

LPPIEKLTL

>B.BR.10.10BR_MG013.KT427686

LPPLERLTL

>B.BR.10.10BR_MG014.KT427685

LPQLERLTL

>B.BR.10.10BR_MG016.KT427684

LPPLERLTL

>B.BR.10.10BR_MG018.KT427683

LPPLERLTI

>B.BR.10.10BR_MG020.KT427682

LPPLERLTL

>B.BR.10.10BR_MG028.KT427680

LPPLERLTL

>B.BR.10.10BR_MG031_2.KT427841

LPPIERLTL

>B.BR.10.10BR_MG035.KJ849804

LPPLERLTL

>B.BR.10.10BR_MG038.KT427676

LPPLERLTL

>B.BR.10.10BR_MG039.KT427675

LPPLERLTL

>B.BR.10.10BR_MG041.KT427673

LPPLERLTL

>B.BR.10.10BR_MG044.KT427671

LPPLERLTL

>B.BR.10.10BR_MG045.KT427670

LPPIERLTL

>B.BR.10.10BR_MG049.KT427669

LPPIERLNL

>B.BR.10.10BR_PE003.KT427747

LPPIERLTL

>B.BR.10.10BR_PE010.KJ849788

LPPLERLTL

>B.BR.10.10BR_PE012.KT427744

LPPLERLTL

>B.BR.10.10BR_PE013.KT427743

LPPLERLTL

>B.BR.10.10BR_PE014.KT427742

LPPLERLTL

>B.BR.10.10BR_PE017.KT427869

LPPLXRLTL

>B.BR.10.10BR_PE019.KT427740

LPPLERLTL

>B.BR.10.10BR_PE020.KT427739

LPPLERLTL

>B.BR.10.10BR_PE021.KT427738

LPPIERLTL

>B.BR.10.10BR_PE022.KT427737

LPQLERLTL

>B.BR.10.10BR_PE024.KT427735

LPPLDRLTI

>B.BR.10.10BR_PE028.KT427734

LPPLDRLTL

>B.BR.10.10BR_PE030.KT427733

LPPLERLTL

>B.BR.10.10BR_PE031.KT427732

LPPLERLTL

>B.BR.10.10BR_PE033.KT427731

LPPLERLTL

>B.BR.10.10BR_PE034.KJ849803

LPPLERLTL

>B.BR.10.10BR_PE035.KT427730

LPPLERLTL

>B.BR.10.10BR_PE036.KT427729

LPPLERLTL

>B.BR.10.10BR_PE037.KT427728

LPPLERLTL

>B.BR.10.10BR_PE038.KT427727

LPPLERLTL

>B.BR.10.10BR_PE040.KT427725

LPPLERLTL

>B.BR.10.10BR_PE041.KT427724

LPPIERLTI

>B.BR.10.10BR_PE042.KT427723

LPPLXRLTL

>B.BR.10.10BR_PE044.KT427721

LPPLERLTL

>B.BR.10.10BR_PE045.KT427720

LPPLEGLTI

>B.BR.10.10BR_PE047.KT427719

LPPLERLTL

>B.BR.10.10BR_PE048.KT427718

LPPLERLTL

>B.BR.10.10BR_PE049.KT427717

LPPLERLNL

>B.BR.10.10BR_PE051.KT427716

LPPLERLTL

>B.BR.10.10BR_PE052.KT427715

LPPLERLTL

>B.BR.10.10BR_PE053.KJ849767

LPPLERLSL

>B.BR.10.10BR_PE054.KT427714

LPPLERLTL

>B.BR.10.10BR_PE055.KT427847

LPPLERLTL

>B.BR.10.10BR_PE058.KT427713

LPPLERLTL

>B.BR.10.10BR_PE061.KT427870

LPSLERLTL

>B.BR.10.10BR_PE067.KT427712

LPPLERLTL

>B.BR.10.10BR_PE068.KT427711

LPPLERLTL

>B.BR.10.10BR_PE070_2.KT427853

LPPLERLTL

>B.BR.10.10BR_PE072.KT427709

LPPLERLTL

>B.BR.10.10BR_PE074.KT427708

LPPLERLTL

>B.BR.10.10BR_PE076.KT427707

LPPLERLTL

>B.BR.10.10BR_PE077.KT427706

LPPLERLTL

>B.BR.10.10BR_PE079.KT427704

LPPLERLTL

>B.BR.10.10BR_PE081.KT427872

LPPLERLTL

>B.BR.10.10BR_PE082.KT427703

LPPLERLTL

>B.BR.10.10BR_PE083.KT427702

LPPLERLTL

>B.BR.10.10BR_PE089.KT427873

LPPLERLTL

>B.BR.10.10BR_PE091.KJ849817

LPPLERLTL

>B.BR.10.10BR_PE093.KT427701

LPPLERLTL

>B.BR.10.10BR_PE095.KT427700

LPPLERLTL

>B.BR.10.10BR_PE096.KT427699

LPSLERLTL

>B.BR.10.10BR_PE097.KJ849818

LPPLERLTL

>B.BR.10.10BR_PE100.KJ849820

LPPLERLTL

>B.BR.10.10BR_PE101.KT427697

LPPLERLTL

>B.BR.10.10BR_PE103.KT427696

LPPLERLTL

>B.BR.10.10BR_PE104.KJ849780

LPPIERLNL

>B.BR.10.10BR_PE105.KT427695

LPPLERLTL

>B.BR.10.10BR_PE106.KT427694

LPPLERLTL

>B.BR.10.10BR_PE110.KT427692

LPPLERLTL

>B.BR.10.10BR_PE112.KT427691

LPPLERLTL

>B.BR.10.10BR_RJ004_2.KT427860

LPPLERLTL

>B.BR.10.10BR_RJ005.KT427792

LPPLERLTL

>B.BR.10.10BR_RJ008.KT427791

LPPLERLNI

>B.BR.10.10BR_RJ016.KT427788

LPPLERLTL

>B.BR.10.10BR_RJ017.KT427787

LPPLERLTI

>B.BR.10.10BR_RJ018_2.KT427861

LPPLERLTL

>B.BR.10.10BR_RJ019.KT427786

LPPLERLNL

>B.BR.10.10BR_RJ020.KT427785

LPPIERLTL

>B.BR.10.10BR_RJ024.KT427784

LPPLERLTL

>B.BR.10.10BR_RJ025.KT427783

LPPLERLTL

>B.BR.10.10BR_RJ030.KT427781

LPPLERLTL

>B.BR.10.10BR_RJ032.KJ849801

LPPLERLTL

>B.BR.10.10BR_RJ033_2.KT427656

LPPLERLTL

>B.BR.10.10BR_RJ040.KT427650

LPPLERLTL

>B.BR.10.10BR_RJ042.KT427777

LPPLERLTL

>B.BR.10.10BR_RJ050.KJ849811

LPPLERLTL

>B.BR.10.10BR_RJ051.KJ849825

LPPLERLTL

>B.BR.10.10BR_RJ052.KT427775

LPPLERLTL

>B.BR.10.10BR_RJ054.KJ849814

LPPLERLTL

>B.BR.10.10BR_RJ064.KT427772

LPPLERLTL

>B.BR.10.10BR_RJ065.KT427866

LPPLERLTL

>B.BR.10.10BR_RJ067.KT427771

LPPLERLTL

>B.BR.10.10BR_RJ068.KT427770

LPPLERLTL

>B.BR.10.10BR_RJ078.KT427769

LPPLERLTL

>B.BR.10.10BR_RJ079.KT427768

LPPLERLTL

>B.BR.10.10BR_RJ081.KT427767

LPPLERLTL

>B.BR.10.10BR_RJ083.KT427766

LPPLERLTL

>B.BR.10.10BR_RJ085.KT427765

LPPLERLTL

>B.BR.10.10BR_RJ086.KT427764

LPPLERLSL

>B.BR.10.10BR_RJ088.KT427763

LPPLERLTL

>B.BR.10.10BR_RJ090.KT427761

LPPLERLTL

>B.BR.10.10BR_RJ097.KT427757

LPPIERLTI

>B.BR.10.10BR_RJ098.KT427756

LPPLERLNL

>B.BR.10.10BR_RJ100.KT427754

LPSLERLTL

>B.BR.10.10BR_RJ101.KT427753

LPPIERLTL

>B.BR.10.10BR_RJ105_2.KT427654

LPPLERLTL

>B.BR.10.10BR_RJ106.KT427751

LPPLEKLTL

>B.BR.10.10BR_RJ107.KT427750

LPPLERLTL

>B.BR.10.10BR_RJ110.KT427749

LPPIERLTL

>B.BR.10.10BR_RJ111.KT427748

LPPLERLTL

>B.BR.10.10BR_SP002.KT427830

LPPLERLTL

>B.BR.10.10BR_SP003.KJ849785

LPPLERLTL

>B.BR.10.10BR_SP004.KT427829

LPPLERLNL

>B.BR.10.10BR_SP006.KT427827

LPPLERLTL

>B.BR.10.10BR_SP007.KT427826

LPPLERLTL

>B.BR.10.10BR_SP008.KJ849821

LPPLERLNL

>B.BR.10.10BR_SP012.KT427824

LPPLERLTL

>B.BR.10.10BR_SP014.KJ849790

LPPLERLNL

>B.BR.10.10BR_SP019.KT427819

LPPLERLTL

>B.BR.10.10BR_SP021.KJ849796

LPPLERLTL

>B.BR.10.10BR_SP027.KT427815

LPPLERLTL

>B.BR.10.10BR_SP032.KT427811

LPPLERLSI

>B.BR.10.10BR_SP036.KT427808

LPPLERLTL

>B.BR.10.10BR_SP038.KJ849805

LPPLERLTL

>B.BR.10.10BR_SP043.KJ849807

LPPLERLTL

>B.BR.10.10BR_SP044.KT427805

LPPLERLTL

>B.BR.10.10BR_SP045.KJ849808

LPPIEKLNL

>B.BR.10.10BR_SP047.KT427804

LPPLERLTL

>B.BR.10.10BR_SP050.KJ849812

LPPLERLTL

>B.BR.10.10BR_SP051.KT427803

LPPLERLTL

>B.BR.10.10BR_SP052.KT427802

LPPLERLTL

>B.BR.10.10BR_SP055.KJ849815

LPPLERLTL

>B.BR.10.10BR_SP065.KT427797

LPPIERLTL

>B.BR.10.10BR_SP067.KT427796

LPPLERLTL

>B.BR.10.10BR_SP073_2.KT427667

LPPLERLTL

>B.BR.10.10BR_SP074.KT427794

LPPLERLTL

>B.BR.10.DEMB10BR038.KU749390

LPPIERLTL

>B.BR.16.HI2016_02.MG571981

LPPLERLTL

>B.BR.16.HI2016_03.MG571982

LPPLERLTL

>B.BR.16.HI2016_04.MG571983

LPPLERLTL

>B.BR.16.HI2016_12.MG571987

LPPLERLTL

>B.BR.16.HI2016_14.MG571990

LPPLERLTL

>B.BR.16.HI2016_15.MG571991

LPPLERLTL

>B.BR.16.HI2016_19.MG571996

LPPLERLTL

>B.BR.16.HI2016_21.MG571999

LPPLERLTL

>B.BR.16.HI2016_22.MG572001

LPQLERLTL

>B.BR.16.HI2016_23.MG572002

LPPLERLTL

>B.BR.16.HI2016_27.MG572006

LPPLERLTL

>B.BR.16.HI2016_31.MG572010

LPPLERLTL

>B.CA.03.HDNDRPI032B2.GU562135

LPPLERLTL

>B.CA.03.PSL024B10.GU562272

LPPLERLTL

>B.CA.04.HDNDRPI034A4.GU562155

LPPLERLTL

>B.CA.06.502_1027_wg01.JF320413

LPPLERLTL

>B.CA.06.502_1799_FL02.JF320427

LPPLERLTL

>B.CA.06.HTM385B7.GU562266

LPPLERLTL

>B.CA.07.502_1191_03.JF320424

LPPLERLTL

>B.CA.96.WC10C_10.AY314061

LPLLERLTI

>B.CA.97.ACTDM580208A15.GU562033

LPPLERLTL

>B.CA.97.CQLDR03A1.GU562058

LPPLERLTL

>B.CA.97.HTM319C1.GU562236

LPPLERLTL

>B.CA.98.ACT54869022A2.GU562001

LPPLERLTL

>B.CA.98.HDNDRPI001B10.GU562080

LPPLERLTL

>B.CA.99.CAN11FULL.AY779558

LPPLERLTL

>B.CA.x.GOL016V01.DQ322223

LPPLEGLTL

>B.CA.x.HDM003V01.DQ322227

LPPIDRLTL

>B.CA.x.HND_DRPI039V01.DQ322225

LPPLERLTL

>B.CA.x.HTM360V05_variant_3.DQ322239

LPPLERLTL

>B.CH.00.HIV_CH_BID_V3529_2000.JQ403022

LPPLERLTL

>B.CH.00.HIV_CH_BID_V3530_2000.JQ403023

LPPLERLNL

>B.CH.00.M1_0007251_NFLG95.KC797171

LPPLERLTL

>B.CH.01.HIV_CH_BID_V3531_2001.JQ403024

LPPLERLTL

>B.CH.01.HIV_CH_BID_V3533_2001.JQ403025

LPPLERLTL

>B.CH.01.HIV_CH_BID_V3534_2001.JQ403026

LPPLERLTL

>B.CH.02.HIV_CH_BID_V3511_2002.JQ403019

LPPLERLTL

>B.CH.02.HIV_CH_BID_V3527_2002.JQ403021

LPPLERLTL

>B.CH.02.HIV_CH_BID_V3539_2002.JQ403029

LPPLDRLTL

>B.CH.02.HIV_CH_BID_V4424_2002.JQ403044

LPPLERLTL

>B.CH.02.HIV_CH_BID_V4478_2002.JQ403047

LPPLERLTL

>B.CH.03.HIV_CH_BID_V4421_2003.JQ403043

LPPLERLTL

>B.CH.03.HIV_CH_BID_V4470_2003.JQ403045

LPPLERLTL

>B.CH.03.HIV_CH_BID_V4474_2003.JQ403046

LPPLERLTL

>B.CH.04.HIV_CH_BID_V4408_2004.JQ403042

LPPLERLTL

>B.CH.08.M2_0803101_NFLG8.KC797225

LPPLERLTL

>B.CN.03.B03.EU363827

LPPLERLTL

>B.CN.03.SHXDC0081.JF932492

LPQLERLTL

>B.CN.05.05CNHB_hp3.DQ990880

LPPLERLTL

>B.CN.06.B04.EU363828

LPPLERLTL

>B.CN.06.B05.EU363829

LPPLERLTL

>B.CN.06.CC056.JF932482

LPPLERLTL

>B.CN.07.AH070011.JF932468

LPPIERLTL

>B.CN.07.AH070014.JF932469

LPPLERLTL

>B.CN.07.AH070017.JF932470

LPPIERLTL

>B.CN.07.AH070018.JF932471

LPPLERLTL

>B.CN.07.AH070057.JF932472

LPPLERLTL

>B.CN.07.BJ070030.JF932473

LPPLEKLTL

>B.CN.07.BJOX003000.e02.KM217584

LPPLERLTL

>B.CN.07.BJOX006000.e05.KM217662

LPPLERLTL

>B.CN.07.BJOX007000.e01.KM217685

LPPIERLTL

>B.CN.07.BJOX014000.e37.KM217802

LPPLERLTL

>B.CN.07.BJOX022000.e02.KM217995

LPPLERLTL

>B.CN.07.CBJC261.JF932474

LPPLERLTL

>B.CN.07.CBJC392.JF932475

LPPLERLTL

>B.CN.07.CBJC394.JF932476

LPPLERLTL

>B.CN.07.CBJC396.JF932477

LPPIERLTL

>B.CN.07.FJ070016.JF932483

LPPLERLTL

>B.CN.07.GS070017.JF932484

LPPLERLTL

>B.CN.07.GZ070002.JF932485

LPPLERLTL

>B.CN.07.GZ070030.JF932486

LPPLERLTL

>B.CN.07.HB070006.JF932487

LPPLERLTL

>B.CN.07.HB070022.JF932488

LPPIERLTL

>B.CN.07.HB070035.JF932489

LPPIERLTL

>B.CN.07.JL070038.JF932490

LPPLERLTL

>B.CN.07.JS070389.JF932491

LPPLERLTL

>B.CN.07.SX070080.JF932493

LPPLERLTL

>B.CN.07.hb070025.JF932499

LPPLERLTL

>B.CN.07.hen1345.JF932500

LPPIERLTL

>B.CN.08.1106.HQ215554

LPPLERLTL

>B.CN.08.BJOX035000.e01.KM218138

LPPLERLTL

>B.CN.08.BJOX046000.e14.KM218195

LPPLERLTL

>B.CN.08.BJOX047000.e19.KM218228

LPPIERLTL

>B.CN.08.CBJC476.JF932478

LPPLERLTL

>B.CN.08.CBJC489.JF932479

LPPLERLTL

>B.CN.08.CBJC500.JF932480

LPPLERLTL

>B.CN.08.CBJC503.JF932481

LPPLERLTL

>B.CN.08.cbjc468.JF932498

LPPLERLTL

>B.CN.09.09LNA014.JX960597

LPPLERLTL

>B.CN.09.09LNA336.JX960599

LPPLERLTL

>B.CN.09.09LNA439.JX960598

LPPLERLTL

>B.CN.09.09YNRL215042sg.KC899011

LPPLERLTL

>B.CN.09.1121.HQ215556

LPPLERLTL

>B.CN.09.DEMB09CN002.KC596066

LPPIERLTL

>B.CN.09.YN09P0014.JF932494

LPSLERLTL

>B.CN.09.ZK042.JF932497

LPPLERLTL

>B.CN.10.DEMB10CN002.JX140658

LPEIERLTL

>B.CN.12.DEMB12CN006.KP109511

LPPIERLTL

>B.CN.12.DEMB12CN010.KP109512

LPPLERLTL

>B.CN.13.BJMP3116B.KU724103

LPPLERLTL

>B.CN.13.BJMP3294B.KU724105

LPPIERLTL

>B.CN.98.YN9802.JF932495

LPPLERLTL

>B.CN.98.YN9838.JF932496

LPPLERLTL

>B.CN.99.plwj11_6.GU647196

LPPLERLTL

>B.CN.x.B06.EU363830

LPPLERLTL

>B.CN.x.RL42.U71182

LPPLERLTL

>B.CO.01.PCM001.AY561236

LPPLERLNL

>B.CO.01.PCM013.AY561237

LPPIERLTL

>B.CO.01.PCM034.AY561238

LPPLERLTL

>B.CO.01.PCM074.AY561240

LPPLERLTL

>B.CU.12.12CU087.KR914675

LPPLERLTL

>B.CU.14.14CU005.KR914676

LPPLERLTL

>B.CU.14.14CU007.KR914678

LPPLERLTL

>B.CU.99.Cu19.AY586542

LPPLERLTL

>B.CU.99.Cu43.AY586543

LPPLERLTL

>B.CY.05.CY018.FJ388890

LPPLERLTL

>B.CY.05.CY020.FJ388891

LPPLERLTL

>B.CY.05.CY028.FJ388895

LPPLERLTL

>B.CY.05.CY032.FJ388898

LPPLERLTL

>B.CY.05.CY033.FJ388957

LPPLERLTL

>B.CY.05.CY035.FJ388958

LPPLERLTL

>B.CY.05.CY036.FJ388959

LPPLERLTL

>B.CY.05.CY037.FJ388899

LPPLERLTL

>B.CY.05.CY055.FJ388904

LPPIDRLTL

>B.CY.05.CY056.FJ388905

LPPLERLTL

>B.CY.05.CY065.FJ388910

LPPIERLTL

>B.CY.05.CY067.FJ388911

LPPLERLTL

>B.CY.05.CY068.FJ388912

LPPLERLTL

>B.CY.05.CY070.FJ388960

LPPLERLTL

>B.CY.05.CY074.FJ388915

LPPLERLTL

>B.CY.05.CY088.FJ388919

LPPLERLTL

>B.CY.05.CY100.FJ388964

LPPLERLTL

>B.CY.05.CY110.FJ388927

LPPIERLTL

>B.CY.05.CY113.FJ388930

LPPLERLNL

>B.CY.05.CY124.FJ388933

LPPLERLTL

>B.CY.05.CY130.FJ388934

LPPLERLTL

>B.CY.05.CY131.FJ388935

LPPLERLTL

>B.CY.05.CY137.FJ388937

LPPLERLTL

>B.CY.05.CY142.FJ388965

LPPLERLTL

>B.CY.05.CY149.FJ388940

LPPLERLTL

>B.CY.05.CY150.FJ388941

LPPLDRLTL

>B.CY.06.CY180.FJ388955

LPPLERLTL

>B.CY.07.CY184.JF683738

LPPLERLSL

>B.CY.07.CY188.JF683741

LPPLERLTL

>B.CY.07.CY189.JF683742

LPPLERLTL

>B.CY.07.CY190.JF683743

LPPLERLTL

>B.CY.07.CY195.JF683747

LPPLERLTL

>B.CY.07.CY197.JF683749

LPPLERLTL

>B.CY.07.CY198.JF683750

LPPLERLTL

>B.CY.07.CY199.JF683751

LPPLERLTL

>B.CY.07.CY201.JF683753

LPPLERLSF

>B.CY.07.CY202.JF683754

LPPLERLTL

>B.CY.07.CY204.JF683756

LPPLERLTL

>B.CY.07.CY214.JF683764

LPPLERLTL

>B.CY.07.CY216.JF683765

LPPLERLTL

>B.CY.08.CY220.JF683769

LPPLERLTL

>B.CY.08.CY224.JF683773

LPPLERLTL

>B.CY.08.CY226.JF683775

LPPLERLTL

>B.CY.08.CY229.JF683778

LPPLERLTL

>B.CY.08.CY232.JF683781

LPPLERLTL

>B.CY.08.CY237.JF683784

LPPLGRLTL

>B.CY.08.CY238.JF683785

LPPHERLTL

>B.CY.09.CY241.JF683787

LPPLERLNL

>B.CY.09.CY244.JF683790

LPPIERLTL

>B.CY.09.CY250.JF683793

LPPLERLTL

>B.CY.09.CY251.JF683794

LPPLERLTL

>B.CY.09.CY253.JF683796

LPPLERLTL

>B.CY.09.CY254.JF683797

LPPLERLTL

>B.CY.09.CY258.JF683801

LPSLERLTL

>B.CY.09.CY262.JF683804

LPPLKRLTL

>B.CY.09.CY263.JF683805

LPPLERLTL

>B.CY.09.CY266.JF683807

LPPLDRLTL

>B.DE.03.HIV_DE_BID_V3307_2003.JQ403048

LPPLERLTL

>B.DE.04.9213_d0.JQ416158

LPPLDRLTL

>B.DE.04.963987.KT124812

LPPLERLTL

>B.DE.04.HIV_DE_BID_V4131_2004.JQ403037

LPPLERLTL

>B.DE.07.906153.KT124803

LPPLERLTL

>B.DE.08.147984.KT124751

LPPLERLTL

>B.DE.08.154162.KT124752

LPPLERLTL

>B.DE.08.176952.KT124755

LPPLERLTL

>B.DE.08.255524.KT124757

LPPIERLKL

>B.DE.08.296004.KT124762

LPPLDRLTL

>B.DE.08.637829.KT124788

LPPLERLTL

>B.DE.08.654207.KT124789

LPPLERLTL

>B.DE.08.822582.KT124794

LPPLDRLTL

>B.DE.08.882283.KT124800

LPPLERLTL

>B.DE.08.954229.KT124810

LPPLERLTL

>B.DE.09.136172.KT124749

LPPIDRLTL

>B.DE.09.159793.KT124753

LPPIERLTL

>B.DE.09.172508.KT124754

LPPLERLTL

>B.DE.09.585067.KT124782

LPPLERLTL

>B.DE.09.923040.KT124805

LPPIERLTL

>B.DE.10.290307.KT124760

LPPLERLTL

>B.DE.10.320435.KT124763

LPPIERLTL

>B.DE.10.464704.KT124772

LPPLERLTL

>B.DE.10.556743.KT124779

LPPLERLTL

>B.DE.10.571373.KT124780

LPPLERLTL

>B.DE.10.587196.KT124783

LPPLERLTL

>B.DE.10.863847.KT124797

LPPLERLTL

>B.DE.10.iso4_w10.KU612900

LPPLERLTL

>B.DE.10.iso5_h10.KU612901

LPPLERLTL

>B.DE.12.328893.KT124765

LPPLERLTL

>B.DE.12.635056.KT124787

LPPLERLTL

>B.DE.12.956306.KT124811

LPPLERLTL

>B.DE.13.366396.KT124767

LPPLERLTL

>B.DE.13.947915.KT124808

LPPLDRLTL

>B.DE.86.D117III_child.AF490512

LPPLERLTL

>B.DE.86.D31.U43096

LPPLERLTL

>B.DE.86.HAN.U43141

LPPLERLTL

>B.DE.x.DEMBXXDE001.KC596067

LPPLERLTL

>B.DK.01.CTL_017.EF514705

LPPLERLTL

>B.DK.01.CTL_018.EF514706

LPPLERLTI

>B.DK.01.CTL_023.EF514707

LPPLERLTL

>B.DK.01.CTL_030.EF514708

LPPLERLTL

>B.DK.01.CTL_033.EF514709

LPPLERLTL

>B.DK.01.CTL_035.EF514710

LPPLERLTL

>B.DK.01.CTL_041.EF514711

LPPLERLTL

>B.DK.04.PMVL_012.EF514699

LPPLERLTL

>B.DK.04.PMVL_013.EF514700

LPPLERLTL

>B.DK.04.PMVL_018.EF514697

LPPLERLTL

>B.DK.04.PMVL_025.EF514702

LPPLERLTL

>B.DK.04.PMVL_027.EF514698

LPPLERLTL

>B.DK.04.PMVL_039.EF514703

LPPXERLTL

>B.DK.04.PMVL_049.EF514701

LPPLERLTL

>B.DK.07.PMVL_011.FJ694790

LPPLERLTL

>B.DO.05.05DO_160884.EU839597

LPPLERLTL

>B.DO.05.05DO_162387.EU839596

LPPLERLTL

>B.DO.05.05DO_163007.EU839598

LPPLERLTL

>B.DO.11.DEMB11DR001.KY658702

LPPLERLTL

>B.EC.89.EC003.AY173959

LPPLERLTL

>B.EC.89.EC102.AY173960

LPPLERLTL

>B.ES.05.R15.KT200351

LPSLERLTL

>B.ES.05.X1890.EU786672

LPPLERLTL

>B.ES.06.R14.KT200350

LPPLERLTL

>B.ES.06.X1958.EU786674

LPPLERLTL

>B.ES.06.X1959.EU786675

LPPLERLTL

>B.ES.06.X1998.EU786676

LPPIERLTL

>B.ES.06.X2102.EU786677

LPPLERLTL

>B.ES.07.X2149.EU786678

LPPLERLTL

>B.ES.07.X2210_3.EU786679

LPPLERLTL

>B.ES.07.X2231.EU786680

LPPLERLTL

>B.ES.08.DEMB08ES001.JX140653

LPPLERLTL

>B.ES.08.ES_X2515_3.GQ372988

LPPLERLTL

>B.ES.08.ES_X2556_3.GQ372990

LPPLERLSL

>B.ES.08.P2021_3.FJ853620

LPPIERLTL

>B.ES.08.R11.KT200349

LPPLERLTL

>B.ES.08.R5.KT200354

LPPLERLTL

>B.ES.08.R9.KT200358

LPPLERLTL

>B.ES.08.X2425_2.FJ670525

LPPLERLTL

>B.ES.08.X2510_2.FJ853622

LPPLERLTL

>B.ES.08.X2555_2.GU362883

LPPIDRLSL

>B.ES.08.X2574_2.GU362886

LPPLDRLTL

>B.ES.09.DEMB09BO001.JX140656

LPPLERLTL

>B.ES.09.DEMB09ES007.KC473841

LPPLERLTL

>B.ES.09.P2149_3.GU362881

LPPLERLTL

>B.ES.09.X2689_2.GU362885

LPPLERLTL

>B.ES.09.X2730_2_nt0544_9494.MF157736

LPPLERLTL

>B.ES.10.DEMB10ES002.KC473842

LPPLERLTL

>B.ES.10.R3.KT200352

LPPLEKLTL

>B.ES.10.R4.KT200353

LPPLERLTL

>B.ES.10.R6.KT200355

LPPLERLTL

>B.ES.10.R7.KT200356

LPPLERLTL

>B.ES.10.R8.KT200357

LPPLERLTL

>B.ES.10.X2899_2s_nt0759_9478.MF157735

LPPLERLTL

>B.ES.13.DEMB13ES010.KP109518

LPPLERLTL

>B.ES.13.P6.KT200348

LPPLEGLTL

>B.ES.14.100_112.KY465967

LPPLERLTI

>B.ES.14.ARP1195.KT276255

LPPLERLTL

>B.ES.14.ARP1196.KT276256

LPPLERLTL

>B.ES.14.ARP1202.KT276262

LPPLDRLTL

>B.ES.14.ARP1203.KT276263

LPPLERLTL

>B.ES.14.ARP1204.KT276264

LPPLERLTL

>B.ES.14.ARP1206.KT276266

LPPLERLTL

>B.ES.14.ARP1207.KT276267

LPPLERLTL

>B.ES.14.ARP1208.KT276268

LPPLERLTL

>B.ES.14.EUR_0031.KU685591

LPPLERLTL

>B.ES.14.EUR_0043.KU685583

LPPLERLTL

>B.ES.14.EUR_0044.KU685584

LPPLDRLTL

>B.ES.14.EUR_0045.KU685585

LPPLERLTL

>B.ES.14.EUR_0046.KU685586

LPPLERLTL

>B.ES.14.EUR_0052.KU685589

LPPLERLTL

>B.ES.14.EUR_0053.KU685590

LPPLERLTL

>B.ES.15.100594.KY989951

LPSLEGLTL

>B.ES.15.100596.KY989953

LPPLERLTL

>B.ES.15.100597.KY989949

LPPLERLTL

>B.ES.15.100598.KY989954

LPPLERLTL

>B.ES.15.100600.KY989956

LPPLERLTL

>B.ES.15.100_116.KY465969

LPPLERLTL

>B.ES.15.100_121.KY514083

LPPLERLTL

>B.FR.03.LA06ToXa.KU168261

LPPLERLTL

>B.FR.05.DEMB05FR001.JX140652

LPPLERLTL

>B.FR.08.DEMB08FR002.JX140654

LPPLERLTL

>B.FR.09.DEMB09FR001.KF716494

LPSLERLTL

>B.FR.09.DEMB09FR002.KF716495

LPPLERLNL

>B.FR.11.DEMB11FR001.KF716496

LPPLERLTL

>B.FR.85.NL43xWC001.AF003887

LPPIERLTL

>B.GB.02.MM27_d0032_ipe012_SGA_8.MG902199

LPPLERLTL

>B.GB.03.MM33d12p.HM586187

LPPLERLTL

>B.GB.04.MM24_d0941_ipe0027_SGA_12.MG902157

LPPLERLTL

>B.GB.04.MM39d11p.HM586193

LPPLERLTL

>B.GB.04.MM42d22_GN1.HM586198

LPPLERLTL

>B.GB.05.MM43d368_GN1.HM586209

LPPLERLTL

>B.GB.05.MM45d22_GN1.HM586210

LPPLERLTL

>B.GB.08.DEMB08UK003.KY658697

LPPLERLTI

>B.GB.13.13592_1_17.3.MF109359

LPPLERLTL

>B.GB.13.13592_1_21.3.MF109364

LPPLERLTL

>B.GB.13.13592_1_26.3.MF109369

LPPLERLNL

>B.GB.13.13592_1_29.3.MF109372

LPPLERLTL

>B.GB.13.13592_1_33.3.MF109376

LPPLERLTL

>B.GB.13.13592_1_4.3.MF109388

LPSLERLTL

>B.GB.13.13659_1_66.3.MF109454

LPPLERLTL

>B.GB.13.13774_1_47.3.MF109472

LPPLERLTL

>B.GB.13.13774_1_50.3.MF109475

LPPLERLTL

>B.GB.13.13774_1_54.3.MF109479

LPPLERLTL

>B.GB.13.13774_1_55.3.MF109480

LPPLERLTL

>B.GB.13.13774_1_63.3.MF109487

LPPLERLTL

>B.GB.13.13774_1_71.3.MF109494

LPPLERLTL

>B.GB.13.13774_1_79.3.MF109501

LPPLERLTL

>B.GB.13.13774_1_84.3.MF109506

LPPLERLTL

>B.GB.13.15171_1_25.4.MF109656

LPPLERLTL

>B.GB.13.15171_1_32.3.MF109664

LPPLERLTL

>B.GB.13.15171_1_35.4.MF109667

LPPLERLTL

>B.GB.13.15171_1_42.3.MF109675

LPPLERLSL

>B.GB.14.13612_1_11.3.MF109395

LPPLERLTL

>B.GB.14.13612_1_12.4.MF109396

LPPLERLTL

>B.GB.14.13612_1_17.3.MF109400

LPPLERLTL

>B.GB.14.13612_1_25.3.MF109408

LPPLERLNL

>B.GB.14.13612_1_38.3.MF109422

LPPLERLSL

>B.GB.14.13612_1_43.3.MF109428

LPPLERLTL

>B.GB.14.13659_1_58.3.MF109446

LPPLERLTL

>B.GB.14.13659_1_64.3.MF109452

LPPLERLTL

>B.GB.14.13659_1_65.3.MF109453

LPPIERLTL

>B.GB.14.13659_1_69.3.MF109456

LPPLERLTL

>B.GB.14.13659_1_75.3.MF109461

LPPLERLTL

>B.GB.14.14535_1_27.3.MF109524

LPPLERLTL

>B.GB.14.14535_1_28.4.MF109525

LPPLERLTL

>B.GB.14.14535_1_5.3.MF109533

LPPLERLTL

>B.GB.14.14535_1_7.3.MF109534

LPPLERLTL

>B.GB.14.14535_1_9.3.MF109536

LPPLERLTL

>B.GB.14.14592_1_44.3.MF109540

LPPLERLTL

>B.GB.14.14592_1_47.3.MF109543

LPPLERLTL

>B.GB.14.14592_1_57.3.MF109551

LPPLERLTL

>B.GB.14.14592_1_64.3.MF109558

LPPLERLTL

>B.GB.14.14667_1_16.3.MF109578

LPPLERLTL

>B.GB.14.14667_1_2.4.MF109590

LPPLERLTL

>B.GB.14.14727_1_40.3.MF109605

LPPLERLTL

>B.GB.14.14727_1_63.3.MF109624

LPEIERLTL

>B.GB.14.14727_1_66.3.MF109627

LPPLERLTL

>B.GB.14.14727_1_69.3.MF109630

LPPIERLTL

>B.GB.83.CAM1.D10112

LPPLERLTL

>B.GB.86.GB8_46R.AJ271445

LPPIERLTL

>B.GB.94.NIBSC_1.KJ019215

LPPLERLTL

>B.GB.x.MANC.U23487

LPPLERLTL

>B.GE.03.03GEMZ004.DQ207940

LPPLERLTL

>B.GE.03.03GEMZ010.DQ207942

LPSLERLTL

>B.GW.14.DEMB14GW004.MH078546

LPPLEKLTL

>B.HK.06.HK002.FJ460499

LPPLERLTL

>B.HK.06.HK003.FJ460500

LPPLERLTL

>B.HT.05.05HT_129389.EU839602

LPPLERLSL

>B.HT.05.05HT_129473.EU839603

LPPLERLTL

>B.HT.05.05HT_129517.EU839600

LPPLERLTL

>B.HT.05.05HT_129696.EU839601

LPPLERLTL

>B.HT.05.05HT_129805.EU839604

LPPLERLTL

>B.HT.11.DEURF11HT001.MH078551

LPPLERLTI

>B.IN.x.11807.EF694037

LPPLERLTL

>B.IT.05.SG1.DQ672623

LPPLERLTI

>B.JM.05.05JM_KJ108.EU839605

LPPLERLNL

>B.JM.09.09JM_PF09WX.HM030564

LPPLERLTL

>B.JM.09.09JM_PF09XN.HM030559

LPPLEKLSL

>B.JM.09.09JM_PF09YT.HM030560

LPPLERLTL

>B.JM.09.09JM_PF0B8J.HM030561

LPPIDRLTL

>B.JM.09.09JM_PF0B97.HM030562

LPPLERLTL

>B.JM.09.09JM_PF0B9L.HM030565

LPPIERLTL

>B.JM.09.09JM_PF0BB5.HM030563

LPPIERLTL

>B.JP.00.117.AB428551

LPPLERLTL

>B.JP.00.DR2508.AB289588

LPPLERLTL

>B.JP.00.DR2510.AB287372

LPPLERLTL

>B.JP.01.DR388.AB289590

LPPLERLNL

>B.JP.02.194.AB428553

LPPLERLTL

>B.JP.03.285.AB428558

LPPLERLTL

>B.JP.04.04JPDR6075B.AB221126

LPPLERLTL

>B.JP.04.DR5913.AB480696

LPPLERLTL

>B.JP.04.DR6174.AB480692

LPPLERLTL

>B.JP.04.DR6175.AB480694

LPPLERLTL

>B.JP.05.426.AB428556

LPPLERLTL

>B.JP.05.DR6538.AB287363

LPPLERLSL

>B.JP.05.DR6737.AB287364

LPPLERLTL

>B.JP.05.DR7060.AB287367

LPPLERLTL

>B.JP.05.DR7065.AB287368

LPPIERLNL

>B.JP.08.NMC104_clone_01.AB731663

LPPLERLTL

>B.JP.09.NMC127_clone_07.AB731667

LPPLERLNL

>B.JP.11.DEMB11JP002.KF716497

LPPLERLTL

>B.JP.11.NMC851C_clone_13.AB731669

LPPLERLTL

>B.JP.12.DEMB12JP001.KF716498

LPPLERLTL

>B.JP.98.DR1120.AB480698

LPPLERLTL

>B.JP.x.DR1673.AB564745

LPPLERLTL

>B.JP.x.DR1712.AB604946

LPPLERLTL

>B.JP.x.DR1777.AB604948

LPPLERLTL

>B.JP.x.JRC03B.AB565496

LPPLERLTL

>B.JP.x.JRC05B.AB565497

LPPLERLTL

>B.JP.x.JRC65B.AB565502

LPPLERLTL

>B.JP.x.pJPDR0796B02.AB565478

LPPLERLTL

>B.JP.x.pJRC57B09.AB641836

LPPLERLSL

>B.KR.02.HP_19_02LGS11_3443.KJ140264

LPPLERLTL

>B.KR.02.HP_20_02KJO10_3480.KJ140265

LPPLERLTL

>B.KR.02.HP_4_02KGJ10_4782.KJ140249

LPPLERLTL

>B.KR.03.03HJY8.JQ316131

LPPLERLTL

>B.KR.03.03KDE11.JQ316128

LPPLEGLTL

>B.KR.03.03KGS5.JQ316132

LPPLERLTL

>B.KR.03.03LSH1.JQ316127

LPPLERLTL

>B.KR.03.03YGS3.JQ316135

LPQLERLTL

>B.KR.04.04CWS5.JQ316133

LPQLERLNL

>B.KR.04.04KJS8.JQ316130

LPPLERLTL

>B.KR.04.04KJin8_1955.DQ295195

LPPLERLTL

>B.KR.04.04KMH5.DQ295193

LPPLERLTL

>B.KR.04.04KMK5.JQ316126

LPQLERLTL

>B.KR.04.04KYR8.DQ295196

LPPLERLNL

>B.KR.04.04LHS6.AY839827

LPQLERLTL

>B.KR.05.05CSR3.DQ837381

LPPLERLTL

>B.KR.05.05YJN2.JQ316134

LPSLERLTL

>B.KR.07.HP_18_07JHS10_3909.KJ140263

LPPLERLTL

>B.KR.12.12KYY10_10742.KF561441

LPQLERLTL

>B.KR.91.91OSG10.KF561442

LPPLERLTL

>B.KR.92.93LSW7_10899.KJ140266

LPPLERLTL

>B.KR.92.HP_10_02SHJ8_6986.KJ140255

LPPLERLTL

>B.KR.92.HP_11_02PGU10_4780.KJ140256

LPPLERLTI

>B.KR.92.HP_16_12JIS11_6075.KJ140261

LPPLERLSL

>B.KR.92.HP_6_03JHJ2_3477.KJ140251

LPPLERLTL

>B.KR.93.HP_17_02LSP11_2268.KJ140262

LPPLERLTL

>B.KR.95.HP_5_95PJH6_10862.KJ140250

LPPLERLTL

>B.KR.97.WK.AF224507

LPPLERLTL

>B.KR.99.99HYH2.JQ316129

LPPLERLTL

>B.NL.99.671_99T12.AY423381

LPPLERLTL

>B.PE.06.502_0491_wg5.JF320183

LPPLERLTL

>B.PE.06.502_0524_FL04.JF320008

LPPLERLTL

>B.PE.06.502_0648_FL02.JF320215

LPPLERLTL

>B.PE.06.502_0841_FL04.JF320208

LPPLERLTL

>B.PE.06.502_2717_RH03.JF320230

LPPLERLTL

>B.PE.06.502_2794_FL05.JF320244

LPPLERLTL

>B.PE.07.502_0525_wg5.JF320191

LPPLERLTL

>B.PE.07.502_1047_wg5.JF320226

LPPLERLTL

>B.PE.07.502_1399_wg4.JF320013

LPPLERLTL

>B.PE.07.502_2254_FL6.JF320018

LPPLERLTL

>B.PE.07.502_2349_wg2.JF320028

LPPLERLTL

>B.PE.07.502_2622_wg1.JF320189

LPPIERLTL

>B.PE.07.502_2649_wg8.JF320019

LPPLERLTL

>B.PE.13.DEMB13PE010.MH078552

LPPLERLTL

>B.PE.14.DEMB14PE008.MH078553

LPPLERLTL

>B.PE.16.DEMB16PE003.MH078554

LPPLERLTL

>B.PE.16.DEMB16PE009.MH078555

LPPIERLNL

>B.PH.15.1003.MH327746

LPPLERLTL

>B.PH.15.DEMB15PH002.KY658689

LPPLDRLTL

>B.PH.15.DEMB15PH003.KY658690

LPPLERLTL

>B.PH.16.1022.MH327757

LPPLERLSL

>B.PL.x.DEMBXXPL001.KC596069

LPPLERLTL

>B.PY.02.02PY_PSP0019.JN251896

LPPLERLTL

>B.PY.02.02PY_PSP0090.JN251901

LPPLERLTL

>B.PY.03.03PY_PSP0115.JN251906

LPPLERLTL

>B.RU.04.04RU128005.AY682547

LPPLERLTL

>B.RU.04.04RU129005.AY751406

LPPLERLTL

>B.RU.04.04RU139089.AY751407

LPPLERLTL

>B.RU.04.04RU139095.AY819715

LPPLERLTL

>B.RU.09.09RU4457.JX500709

LPPLERLTL

>B.RU.10.10RU6629.JX500707

LPPLERLTL

>B.RU.11.11RU21n.JX500708

LPPLERLTL

>B.SE.03.003SE.MF373125

LPPLERLTL

>B.SE.03.005SE.MF373127

LPPLERLTL

>B.SE.05.007SE.MF373129

LPPLERLTL

>B.SE.05.008SE.MF373130

LPPLERLTL

>B.SE.08.028SE.MF373142

LPPLERLTL

>B.SE.09.SE600001.KP411822

LPPLERLTL

>B.SE.10.038US.MF373149

LPPLERLTL

>B.SE.10.040CA.MF373151

LPPLERLTL

>B.SE.10.SE600046.KP411827

LPTLERLNL

>B.SE.11.058SE.MF373161

LPPLERLTL

>B.SE.11.059SE.MF373162

LPPLERLTL

>B.SE.11.SE600023.KP411824

LPLPQRLTL

>B.SE.11.SE600034.KP411825

LPPLERLTL

>B.SE.12.SE600057.KP411828

LPPLERLTL

>B.SE.13.084TH.MF373186

IPSLERLTL

>B.SE.13.089SE.MF373191

LPPLERLTL

>B.SE.14.099US.MF373201

LPPLERLTL

>B.SE.15.101SE.MF373203

LPPLERLTL

>B.SE.15.102SE.MF373204

LPPLERLTL

>B.TH.00.00TH_C3198.AY945710

LPPLERLTL

>B.TH.04.04TH317223.JN248321

LPPLDRLTL

>B.TH.04.04TH601066.JN248329

LPPLDRLTL

>B.TH.04.04TH803686.JN248333

LPPIERLTL

>B.TH.04.04TH808998.JN248335

LPPLERLTL

>B.TH.04.04TH821921.JN248337

LPPLERLTL

>B.TH.05.05TH355614.JN248343

LPPLERLTL

>B.TH.05.05TH356764.JN248344

LPPIERLTL

>B.TH.05.05TH357801.JN248346

LPPLERLTL

>B.TH.05.05TH429730.JN248347

LPPIERLSL

>B.TH.05.05TH440248.JN248348

LPPLERLTL

>B.TH.05.05TH645189.JN248353

LPPLERLTL

>B.TH.05.05TH736580.JN248354

LPPLERLTL

>B.TH.06.AA010a_WG3.JX446800

LPSIERLTL

>B.TH.06.AA011a08R.JX446818

LPPLDRLTL

>B.TH.06.AA093a_RH1.JX447795

LPPLERLTL

>B.TH.06.NPBQC.KJ769147

LPPLERLTL

>B.TH.07.AA040a_WG11.JX447156

LPPLERLSL

>B.TH.08.AA115c03R.JX448103

LPPLERLTL

>B.TH.08.MERLBDTRC10.JN860769

LPPIERLTL

>B.TH.10.DEMB10TH002.KP109514

LPPLERLTL

>B.TH.90.BK132.AY173951

LPPLERLTL

>B.TH.96.M140.DQ354112

LPQLEKLTL

>B.TH.96.M145.DQ354118

LPPLERLTL

>B.TH.96.M149.DQ354119

LPPLERLTL

>B.TH.99.99TH_C1416.AY945711

LPPLERLTL

>B.TT.00.00TT_CRC08767.EU839606

LPPLERLTL

>B.TT.00.00TT_CRC50018.EU839607

LPPLERLTI

>B.TT.00.00TT_CRC50060.EU839609

LPPLERLTL

>B.TT.01.01TT_CRC50069.EU839608

LPPLERLTL

>B.TT.93.SC05_8C11_2344.EU289200

LPPLERLTL

>B.TT.95.SC45_4B5_2631.EU289201

LPPLERLSL

>B.TW.94.TWCYS_LM49.AF086817

LPPLERLTL

>B.UA.01.01UAKV259.DQ823364

LPPLERLTL

>B.US.00.929172.KT124806

LPPLERLTL

>B.US.00.ES1_20.EF363123

LPPLERLTL

>B.US.00.PRB958_06_TB1_4305.EU289199

LPPLERLTL

>B.US.00.RHPA_TF1.JN944917

LPPLERLTL

>B.US.00.THRO_TF1.JN944930

LPPLERLTL

>B.US.00.WITO_TF1.JN944938

LPPLERLTL

>B.US.01.L805.FJ469738

LPPLERLTL

>B.US.01.REJO_TF1.JN944911

LPPLDRLTL

>B.US.01.TRJO_TF1.JN944936

LPPLERLTL

>B.US.02.04013226_2_flH11.FJ496078

LPPLERLTL

>B.US.02.328659.KT124764

LPPLERLTI

>B.US.02.494131.KT124777

LPSLERLTL

>B.US.02.848017.KT124795

LPPLERLTL

>B.US.02.996401.KT124814

LPPLERLTL

>B.US.02.CR0017Q.FJ469687

LPPLERLTL

>B.US.02.CR0023W.FJ469688

LPPLERLTL

>B.US.02.CR0058S.FJ469693

LPPLDRLTL

>B.US.02.F706.FJ469728

LPPLERLTL

>B.US.02.F714.FJ469730

LPPLERLTL

>B.US.02.F719.FJ469734

LPPLERLTL

>B.US.02.F762P.FJ469735

LPPLERLTL

>B.US.02.F797.FJ469737

LPPLERLTL

>B.US.02.HIV_US_BID_V5249_2002.JQ403102

LPPLERLTL

>B.US.02.HIV_US_BID_V5276_2002.JQ403105

LPPLERLTI

>B.US.02.L8124P.FJ469741

LPPLERLTL

>B.US.02.L861P.FJ469748

LPPLERLTL

>B.US.02.MTF.FJ469751

LPPLERLTL

>B.US.02.PRLS08.FJ469757

LPPLERLTL

>B.US.02.PRLS09.FJ469758

LPPLDRLTL

>B.US.02.PRLS16.FJ469760

LPPLERLTL

>B.US.03.1BRHGA_E13_DP.KC312470

LPPIDRLTL

>B.US.03.73739_13874_1.MH060766

LPQIERLTL

>B.US.03.933384.KT124807

LPPIERLTL

>B.US.03.94959_13871_1.MH060965

LPPLERLTL

>B.US.03.981600.KT124813

LPPLERLTL

>B.US.03.AT01480.FJ469686

LPPLERLTL

>B.US.03.CR0154X.FJ469701

LPPLERLTL

>B.US.03.DEMB03JP004.KC473846

LPPLERLTL

>B.US.03.F7157.FJ469731

LPPLERLTL

>B.US.03.HIV_US_BID_V5279_2003.JQ403106

LPPLERLTL

>B.US.03.L8107.FJ469739

LPPIERLTL

>B.US.03.L8116.FJ469740

LPPIERLTL

>B.US.03.L8152.FJ469742

LPPIDRLTL

>B.US.03.L8180.FJ469743

LPPLERLTL

>B.US.03.PRLS01.FJ469753

LPPLERLTL

>B.US.03.PRLS12.FJ469759

LPPLERLTL

>B.US.03.PRLS24.FJ469764

LPPLERLTL

>B.US.03.SH8233.FJ469770

LPPLERLTL

>B.US.04.014837G.FJ469684

LPPIERLTL

>B.US.04.268977.KT124758

LPPLERLTL

>B.US.04.64236_13865_1.MH060681

LPPIERLTL

>B.US.04.71276_13858_1.MH060746

LPPLERLTL

>B.US.04.98158_13868_1.MH060990

LPPLERLTL

>B.US.04.CR0068P.FJ469695

LPPLDRLTL

>B.US.04.CR0080N.FJ469697

LPPLERLTL

>B.US.04.CR0116Y.FJ469698

LPPLERLTL

>B.US.04.CR0345Q.FJ469722

LPPLERLTL

>B.US.04.ES10_53.EF363127

LPPIERLTL

>B.US.04.ES11_2004_culture.KC935957

LPPLEGLTL

>B.US.04.ES8_43.EF363126

LPPLERLTL

>B.US.04.F7165.FJ469732

LPPLERLTL

>B.US.04.HIV_US_BID_V5258_2004.JQ403103

LPPLERLTL

>B.US.04.HIV_US_BID_V5261_2004.JQ403104

LPPLERLTL

>B.US.04.HIV_US_BID_V5282_2004.JQ403107

LPPLDRLTL

>B.US.04.L819.FJ469745

LPPLERLTL

>B.US.04.SAMI_WGA1.EU547186

LPPLERLNL

>B.US.04.UNC2009_1.EF593269

LPPLERLTL

>B.US.04.UNC4484_13.EF593271

LPPLERLTL

>B.US.04.UNC4911_20.EF593272

LPPLERLTL

>B.US.04.UNC5283_17.EF593273

LPPIERLTL

>B.US.04.UNC5548_11.EF593274

LPPLDRLTL

>B.US.04.UNC5734_10.EF593275

LPPLERLTL

>B.US.04.UNC5799_16.EF593276

LPPLERLTL

>B.US.04.USPI71101EI7y04051pcWG2B9.JN024210

LPPLDRLSL

>B.US.04.USPI83747EI6y04121pcWG2B5.JN024100

LPPLERLTL

>B.US.05.01144G.FJ469682

LPPIERLTL

>B.US.05.012286G.FJ469683

LPPLDRLTL

>B.US.05.04013396_0_flE6.FJ496081

LPPLERLTL

>B.US.05.05US_SAJ_NVS12.JF689852

LPPLERLTL

>B.US.05.05US_SAJ_NVS16.JF689854

LPPLERLTL

>B.US.05.05US_SAJ_NVS3.JF689856

LPPLERLTL

>B.US.05.05US_SAJ_NVS5.JF689857

LPPLERLTL

>B.US.05.05US_SAJ_NVS8.JF689859

LPPLERLTL

>B.US.05.05US_SAJ_NVS9.JF689860

LPPLERLTL

>B.US.05.306159_FL.JX863919

LPPLERLTL

>B.US.05.502_1400_FL02.JF320043

LPPLERLTL

>B.US.05.502_1926_FL01.JF320361

LPPLERLTL

>B.US.05.502_2008_FL04.JF320484

LPPLDRLTL

>B.US.05.502_2136_FL02.JF320185

LPPLERLTL

>B.US.05.502_2495_wg02.JF320054

LPPLERLTL

>B.US.05.509452.KT124778

LPPLERLTL

>B.US.05.CR0175S.FJ469703

LPPLERLTL

>B.US.05.CR0208W.FJ469706

LPPLERLTL

>B.US.05.CR0312W.FJ469718

LPPLERLTL

>B.US.05.L8249.DQ886037

LPPLERLTL

>B.US.05.L827.FJ469747

LPPLERLTL

>B.US.05.MDR_1c.KF990605

LPPLERLTL

>B.US.05.STCO_3_A2.KC312435

LPPLERLTL

>B.US.05.USPI38417EI33y05051pcWG2B2.JN024363

LPPLERLTL

>B.US.05.USPI88403EI14y05121pcWG2B7.JN024344

LPPLERLTL

>B.US.06.06US_SAJ_C164_SC.JF689862

LPPLERLTL

>B.US.06.06US_SAJ_C165_TJ.JF689863

LPPLERLTL

>B.US.06.06US_SAJ_C166_SG.JF689864

LPPLERLTL

>B.US.06.06US_SAJ_C167_LH.JF689865

LPPLERLTL

>B.US.06.06US_SAJ_C168_LS.JF689866

LPPLERLTL

>B.US.06.06US_SAJ_C169_JS.JF689867

LPPLERLTI

>B.US.06.06US_SAJ_C170_JP.JF689868

LPPLXRLTL

>B.US.06.06US_SAJ_NVS22.JF689870

LPPLERLTL

>B.US.06.06US_SAJ_NVS23.JF689871

LPPLERLTI

>B.US.06.06US_SAJ_NVS27.JF689872

LPPLERLTL

>B.US.06.06US_SAJ_NVS31.JF689873

LPPLERLTL

>B.US.06.06US_SAJ_NVS32.JF689874

LPPLDRLTL

>B.US.06.06US_SAJ_NVS35.JF689875

LPPLERLTI

>B.US.06.06US_SAJ_NVS39.JF689876

LPPIERLNL

>B.US.06.502_0053_wg06.JF320615

LPPLERLTL

>B.US.06.502_0062_FL04.JF320613

LPPLERLTL

>B.US.06.502_0176_FL06.JF320363

LPPLERLTL

>B.US.06.502_0227_FL05.JF320036

LPPLERLTL

>B.US.06.502_0309_wg13.JF320048

LPPLERLTL

>B.US.06.502_0322_RH04.JF320308

LPPLERLTL

>B.US.06.502_0341_FL05.JF320003

LPPLERLTI

>B.US.06.502_0346_wg02.JF320097

LPPLERLTL

>B.US.06.502_0572_FL06.JF320038

LPPLERLTL

>B.US.06.502_0717_FL02.JF320526

LPPLERLTL

>B.US.06.502_0762_RH05.JF320329

LPPLERLTL

>B.US.06.502_0839_wg01.JF320263

LPPLERLTL

>B.US.06.502_0897_RH05.JF320502

LPPLERLTL

>B.US.06.502_0923_wg07.JF320160

LPPLERLTL

>B.US.06.502_0961_RH08.JF320011

LPPLERLTL

>B.US.06.502_1046_FL04.JF320564

LPPLERLTL

>B.US.06.502_1055_FL01.JF320169

LPPLERLTL

>B.US.06.502_1174_FL09.JF320053

LPPLERLTL

>B.US.06.502_1211_FL01.JF320151

LPPLERLTL

>B.US.06.502_1512_FL01.JF320356

LPPLERLTL

>B.US.06.502_1619_FL06.JF320126

LPPLERLTL

>B.US.06.502_1919_RH01.JF320184

LPPLERLTL

>B.US.06.502_2437_RH01.JF320592

LPPLERLTL

>B.US.06.502_2667_FL03.JF320145

LPPLERLTL

>B.US.06.689801.KT124791

LPPLERLTL

>B.US.06.701010068_E_J1.GU331147

LPPLERLTL

>B.US.06.BP00054_RH01.JN687749

LPPLERLTL

>B.US.06.BP00055_RH01.JN687750

LPPLERLTL

>B.US.06.BP00057_RH02.JN687758

LPPLERLTL

>B.US.06.CH106_TF1.JN944897

LPPLERLTL

>B.US.06.CH58_TF1.JN944907

LPPLERLTL

>B.US.06.CH77_TF1.JN944909

LPPIERLTL

>B.US.06.CR0047U.FJ469691

LPPLERLTL

>B.US.06.CR0078.FJ469696

LPPLERLTL

>B.US.06.CR0131.FJ469700

LPPLERLTL

>B.US.06.CR0215.FJ469708

LPPLERLTL

>B.US.06.CR0222X.FJ469709

LPPIERLTL

>B.US.06.CR0228Q.FJ469710

LPPLERLTL

>B.US.06.CR0276Z.FJ469714

LPPLERLTL

>B.US.06.CR0361T.FJ469723

LPPLERLNL

>B.US.06.HIV_US_BID_V3047_2006.JQ403068

LPPLERLTL

>B.US.06.HIV_US_BID_V4503_2006.JQ403095

LPPLERLTL

>B.US.06.HIV_US_BID_V4506_2006.JQ403097

LPPLERLTL

>B.US.06.MDR_5a.KF990608

LPPLERLTI

>B.US.07.07US_SAJ_C154.JF689877

LPPLERLTL

>B.US.07.07US_SAJ_C156.JF689879

LPPLERLNL

>B.US.07.07US_SAJ_C161_H1.JF689883

LPPLERLTL

>B.US.07.07US_SAJ_C163_H3.JF689885

LPPLERLTL

>B.US.07.07US_SAJ_C166_MS.JF689886

LPPLERLTL

>B.US.07.07US_SAJ_C200.JF689887

LPPLERLTL

>B.US.07.07US_SAJ_NVS42.JF689889

LPPLERLTL

>B.US.07.07US_SAJ_NVS48.JF689890

LPPIERLTL

>B.US.07.07US_SAJ_NVS54.JF689892

LPPLERLTL

>B.US.07.07US_SAJ_NVS55.JF689893

LPPLERLTL

>B.US.07.306344_FL.JX863921

LPPLERLTL

>B.US.07.502_0287_RH1.JF320375

LPPLERLTL

>B.US.07.502_0364_wg2.JF320563

LPPLERLTI

>B.US.07.502_0388_RH06.JF320315

LPPLERLTL

>B.US.07.502_0823_05.JF320530

LPQLEKLTL

>B.US.07.502_0938_RH04.JF320631

LPPIERLTI

>B.US.07.502_0965_RH01.JF320385

LPPIERLTL

>B.US.07.502_1115_wg1.JF320045

LPPIERLTL

>B.US.07.502_1368_RH02.JF320173

LPPLERLTL

>B.US.07.502_1478_wg4.JF320150

LPPLERLTL

>B.US.07.502_1500_RH01.JF320387

LPPLERLTL

>B.US.07.502_1504_RH07.JF320394

LPPLERLTL

>B.US.07.502_1518_RH03.JF320117

LPPLERLTL

>B.US.07.502_1709_04.JF320467

LPPLERLTL

>B.US.07.502_1897_wg6.JF320182

LPPLERLTL

>B.US.07.502_2000_RH03.JF320279

LPPLERLTL

>B.US.07.502_2241_RH13.JF320539

LPPLERLTL

>B.US.07.502_2289_05.JF320197

LPPLERLTL

>B.US.07.502_2305_01.JF320577

LPPLEGLTL

>B.US.07.502_2586_RH04.JF320131

LPPLERLTL

>B.US.07.891439.KT124801

LPPLERLTL

>B.US.07.BP00058_RH01.JN687759

LPPLERLTL

>B.US.07.BP00063_RH01.JN687761

LPPLERLTL

>B.US.07.BP00064_RH01.JN687762

LPPLERLTL

>B.US.07.BP00067_RH01.JN687763

LPPLERLTL

>B.US.07.CR0027M.FJ469689

LPSLERLTL

>B.US.07.CR0214.FJ469707

LPPLERLTI

>B.US.07.CR0275.FJ469713

LPPLERLTL

>B.US.07.CR0295S.FJ469716

LPPLERLTL

>B.US.07.CR0339X.FJ469721

LPPLERLTL

>B.US.07.HIV_US_BID_V3010_2007.JQ403058

LPPLERLTL

>B.US.07.HIV_US_BID_V3020_2007.JQ403059

LPQLERLTL

>B.US.07.HIV_US_BID_V3021_2007.JQ403060

LPPLERLTL

>B.US.07.HIV_US_BID_V3036_2007.JQ403064

LPPIERLTL

>B.US.07.HIV_US_BID_V3044_2007.JQ403066

LPPLERLTL

>B.US.07.HIV_US_BID_V3053_2007.JQ403071

LPPLERLTL

>B.US.07.HIV_US_BID_V3115_2007.JQ403075

LPPLERLTL

>B.US.07.HIV_US_BID_V3118_2007.JQ403077

LPPLERLTL

>B.US.07.HIV_US_BID_V3120_2007.JQ403078

LPPLERLTL

>B.US.07.HIV_US_BID_V3512_2007.JQ403092

LPPLERLTL

>B.US.07.HIV_US_BID_V3515_2007.JQ403093

LPPIERLTL

>B.US.07.HIV_US_BID_V4516_2007.JQ403096

LPPLERLTL

>B.US.07.MCST_B17.KC312583

LPPLERLTL

>B.US.07.WARO_A13.KC312386

LPPLERLTL

>B.US.08.08US_SAJ_C202.JF689894

LPPLERLTL

>B.US.08.08US_SAJ_C203.JF689895

LPPLERLTL

>B.US.08.08US_SAJ_C204.JF689896

LPPLERLTL

>B.US.08.08US_SAJ_C205.JF689897

LPPIEKLSL

>B.US.08.293050.KT124761

LPPLERLTL

>B.US.08.B.700010607.S.0dps.BF13.JX974238

LPPIERLTL

>B.US.08.BP00061_RH01.JN687760

LPPLERLTL

>B.US.08.CH302.PL.041608.UT.7.KY112135

LPPLERLTL

>B.US.08.CH378.PL.080508.UT.15.KY112149

LPPLERLTL

>B.US.08.HIV_US_BID_V3024_2008.JQ403061

LPPLERLTL

>B.US.08.HIV_US_BID_V3027_2008.JQ403062

LPPLERLTL

>B.US.08.HIV_US_BID_V3032_2008.JQ403063

LPPLERLTL

>B.US.08.HIV_US_BID_V3046_2008.JQ403067

LPPLERLTL

>B.US.08.HIV_US_BID_V3048_2008.JQ403069

LPPLERLTL

>B.US.08.HIV_US_BID_V3050_2008.JQ403070

LPELERLNL

>B.US.08.HIV_US_BID_V3114_2007.JQ403074

LPPLEKLTL

>B.US.08.HIV_US_BID_V3122_2008.JQ403080

LPQLERLNL

>B.US.08.HIV_US_BID_V3128_2008.JQ403082

LPPIERLTL

>B.US.08.HIV_US_BID_V4120_2008.JQ403031

LPPIERLTL

>B.US.08.HIV_US_BID_V4124_2008.JQ403035

LPPLDRLTL

>B.US.08.HIV_US_BID_V4388_2008.JQ403083

LPPLERLTL

>B.US.08.HIV_US_BID_V4389_2008.JQ403084

LPPLERLTL

>B.US.08.HIV_US_BID_V4390_2008.JQ403085

LPPLERLTL

>B.US.08.HIV_US_BID_V4391_2008.JQ403086

LPPLDRLTL

>B.US.08.HIV_US_BID_V4392_2008.JQ403087

LPPLERLNL

>B.US.08.HIV_US_BID_V4393_2008.JQ403088

LPPLERLTL

>B.US.08.HIV_US_BID_V4394_2008.JQ403089

LPPLERLTL

>B.US.08.HIV_US_BID_V4397_2008.JQ403091

LPPIERLTL

>B.US.08.HIV_US_BID_V4489_2008.JQ403094

LPPLERLTL

>B.US.09.B.700010470.w60.420dps.3_B11.JX972342

LPPLERLSL

>B.US.09.C1P.GU733713

LPPLERLTL

>B.US.09.DEMB09US002.JX140657

LPPLERLTL

>B.US.09.DEMB09US003.KC473824

LPPLERLTL

>B.US.09.ES38.KC935958

LPPLERLTL

>B.US.09.LTNP1.KC935959

LPPLERLTL

>B.US.10.10CB4_45E6.KF526141

LPPLERLTL

>B.US.10.505_0686a.WG01.MG196775

LPPLERLTL

>B.US.10.505_0695a.WG06.MG196787

LPPLERLTL

>B.US.10.505_1278b.RH01.MG196997

LPPLERLTL

>B.US.10.505_1962a.WG03.MG197136

LPPLERLTL

>B.US.10.505_2483a.WG03.MG197210

LPPLERLTL

>B.US.10.CH0040_3_d1485_ipe032_15_08.MG900412

LPPLERLTL

>B.US.10.CP1.JN397365

LPPLERLTL

>B.US.10.C.HQ846911

LPPLERLTL

>B.US.10.DEMB10US001.KC473825

LPPLERLTL

>B.US.10.DEMB10US003.KC473826

LPPLERLTI

>B.US.10.DEMB10US004.KC473827

LPPLERLTL

>B.US.10.DEMB10US007.KC473828

LPPLERLTL

>B.US.10.DEMB10US009.KC473829

LPPLERLNL

>B.US.10.DEMB10US011.KC473830

LPPLERLTI

>B.US.10.Pt1_DNA_2.KU677990

LPPIERLTL

>B.US.10.Pt4_DNA_5.KU678074

LPPLERLTI

>B.US.10.VC1.JN397364

LPPLERLTL

>B.US.11.19CB1_induced.KF526228

LPPLERLTL

>B.US.11.20CB4_46F1.KF526265

LPQLERLSL

>B.US.11.22CC9_induced.KF526312

LPPLERLTL

>B.US.11.23CB6_induced.KF526323

LPPLERLTL

>B.US.11.361974.KT124766

LPPLERLTL

>B.US.11.479693.KT124773

LPPLERLTL

>B.US.11.481811.KT124775

LPPLERLTL

>B.US.11.505_0071a.WG04.MG196672

LPPLERLTL

>B.US.11.505_0090a.RH2.MG196679

LPPLERLTL

>B.US.11.505_0102a.WG01.MG196689

LPPLERLTL

>B.US.11.505_0396a.WG8.MG196758

LPPLERLTL

>B.US.11.505_0724a.WG02.MG196815

LPPLERLTL

>B.US.11.505_0840a.WG03.MG196840

LPPLERLNL

>B.US.11.505_0896a.WG08.MG196846

LPPIERLTL

>B.US.11.505_1174a.WG06.MG196942

LPPLERLTL

>B.US.11.505_1730a.WG08.MG197084

LPPLERLTL

>B.US.11.505_1982a.WG07.MG197146

LPPLERLTL

>B.US.11.950965.KT124809

LPPIERLTL

>B.US.11.AMBI_CLONE.KU641402

LPPLERLTL

>B.US.11.CP10_3A.KF384798

LPPLERLTL

>B.US.11.CP12_10.KF384799

LPPLERLTL

>B.US.11.CP13_2.KF384800

LPPIERLTL

>B.US.11.CP3_6.KF384801

LPPLERLTL

>B.US.11.CP4_2B.KF384802

LPPLERLTI

>B.US.11.CP5_3A.KF384803

LPPLERLTL

>B.US.11.CP6_2E.KF384804

LPPLERLTL

>B.US.11.CP7_2B.KF384805

LPPLERLTL

>B.US.11.CP8_4.KF384806

LPPLERLTL

>B.US.11.CP9_1A.KF384807

LPPIERLNL

>B.US.11.DEMB11US002.KC473831

LPPLERLTL

>B.US.11.DEMB11US004.KC473832

LPPLERLTL

>B.US.11.DEMB11US006.KC473833

LPPLERLTL

>B.US.11.DEMB11US011.KC473834

LPPLERLTL

>B.US.11.DEMB11US015.KC473835

LPPLERLTL

>B.US.11.ES22_27.KF384808

LPPLERLTL

>B.US.11.ES38.JN397362

LPPIERLTL

>B.US.11.ES39_42.KF384810

LPPLERLTL

>B.US.11.ES40_24.KF384811

LPPLERLTL

>B.US.11.VC3_14.KF384812

LPPLERLTL

>B.US.11.VC5_3.KF384813

LPPLERLTL

>B.US.11.VC6_2.KF384814

LPPLERLTL

>B.US.12.2302_PBEM_13.KY778473

LPPLERLTL

>B.US.12.231603.KT124756

LPPLERLTL

>B.US.12.2452_PBEM_13.KY778615

LPPLERLTL

>B.US.12.383086.KT124768

LPPLERLTL

>B.US.12.409_133_F_w02VT.MH897911

LPPLERLTL

>B.US.12.420_133_F_w02_10VT.MH897913

LPPLERLTL

>B.US.12.421_34_F_w01VT.MH897915

LPSLERLTL

>B.US.12.426_34_F_w09_20VT.MH897916

LPPLERLTL

>B.US.12.432_133_F_w18VT_WG.MH897917

LPPLERLTL

>B.US.12.505_0012a.WG03.MG196642

LPPIERLTL

>B.US.12.505_0049a.WG07.MG196653

LPPLERLTL

>B.US.12.505_0122a.WG06.MG196702

LPPLERLSL

>B.US.12.505_0332a.RH26.MG196732

LPPLERLTL

>B.US.12.505_0645a.WG11.MG196767

LPPLERLTL

>B.US.12.505_0772a.WG2.MG196819

LPPIERLTL

>B.US.12.505_0821a.WG06.MG196831

LPPLERLTL

>B.US.12.505_0829a.RH03.MG196832

LPPLERLTL

>B.US.12.505_0933a.RH6.MG196866

LPPLERLTL

>B.US.12.505_0997a.WG03.MG196879

LPPLERLTL

>B.US.12.505_0998a.RH31.MG196902

LPALERLTL

>B.US.12.505_1012a.WG08.MG196921

LPSLERLTL

>B.US.12.505_1144a.WG02.MG196922

LPPIERLNL

>B.US.12.505_1226a.WG08.MG196952

LPPLERLTL

>B.US.12.505_1276a.WG03.MG196979

LPSLERLSI

>B.US.12.505_1295a.WG10.MG197013

LPPLERLTL

>B.US.12.505_1358a.WG08.MG197023

LPPIERLTL

>B.US.12.505_1413a.WG05.MG197046

LPPLERLTL

>B.US.12.505_1569a.WG11.MG197055

LPPLERLTL

>B.US.12.505_1672a.WG7.MG197064

LPPLERLTL

>B.US.12.505_1677a.WG10.MG197083

LPPLERLTL

>B.US.12.505_1763a.RH14.MG197113

LPPIERLNL

>B.US.12.505_1993a.RH01.MG197164

LPPLDRLTL

>B.US.12.505_2150a.WG07.MG197176

LPPLERLTL

>B.US.12.505_2227a.WG04.MG197187

LPPLERLTL

>B.US.12.505_2474a.WG06.MG197199

LPPLERLTL

>B.US.12.607523.KT124784

LPPLERLTL

>B.US.12.608647.KT124785

LPPLERLTL

>B.US.12.678584.KT124790

LPPLDRLTL

>B.US.12.N8261_FL_CON.KU901976

LPPLERLTL

>B.US.12.T8250_FL_CON.KU901997

LPPLERLSL

>B.US.13.2026_EM_39.KY766175

LPPLERLTI

>B.US.13.2115_EM_19.KY778345

LPPLERLTL

>B.US.13.2275_EM_21.KY778395

LPPLERLTL

>B.US.13.439_133_F_w11VT.MH897918

LPPLERLNL

>B.US.13.505_0280a.WG04.MG196711

LPPLERLTL

>B.US.13.505_0390a.WG12.MG196745

LPPLERLTL

>B.US.13.505_1270a.WG12.MG196964

LPPLERLTL

>B.US.13.505_1371a.WG10.MG197035

LPPLERLTL

>B.US.13.505_1958a.WG09.MG197127

LPSIERLTL

>B.US.13.862898.KT124796

LPPIERLTL

>B.US.13.ARC_1a.MK214316

LPPLERLTL

>B.US.13.CP02.KX505589

LPPLERLTL

>B.US.13.CP03.KX505616

LPPLERLTL

>B.US.13.CP06.KX505652

LPPLERLTL

>B.US.13.CP07.KX505686

LPPLERLTL

>B.US.13.CP08.KX505695

LPPLDRLTL

>B.US.13.CP09.KX505707

LPPLERLTL

>B.US.13.DEMB13US026.KU749387

LPPLERLTL

>B.US.13.DONOR7tCD4Rep4.B1.p7a21.KY057587

LPPLERLTL

>B.US.13.Donor6.B4.p6c23.KY748513

LPSLERLTL

>B.US.13.Donor8.B9.p5k5.KY748576

LPPLERLTL

>B.US.13.G4_RV_1.KT284371

LPPLERLTL

>B.US.13.S1492_T1_Intact.MG171201

LPSLERLTL

>B.US.14.2046_EM_26.KY778299

LPPLERLTL

>B.US.14.2286.KX505396

LPPLERLTL

>B.US.14.2443.KX505419

LPPLERLTL

>B.US.14.2454.KX505435

LPPIERLTL

>B.US.14.2529.KX505475

LPPLERLTL

>B.US.14.2531.KX505508

LPPLERLTL

>B.US.14.CP10.KX505739

LPPLERLTL

>B.US.14.DEMB14US030.KY658693

LPPLERLTL

>B.US.14.Pt2_DNA_28.KU678026

LPPLERLTL

>B.US.14.Pt3_DNA_6_2.KU678060

LPPLERLTL

>B.US.14.Pt7_DNA_5.KU678125

LPPLERLTL

>B.US.15.2521.KX505446

LPPLERLTL

>B.US.15.3693.KX505555

LPPLERLTL

>B.US.15.454_34_F_w01VT.MH897920

LPPLERLTL

>B.US.15.BVP12tCD4Rep2.3.F4.p3f13.KY057390

LPPLERLTL

>B.US.15.DONOR2.A1.3.p7i13.KY612720

LPPLEKLTL

>B.US.15.Donor4.F6.2.3.p7a7.KY748402

LPPLERLTL

>B.US.15.exc_BVP4.3.F4.p7i4.KY057518

LPPLERLTL

>B.US.15.p46n.KT223503

LPPLERLTL

>B.US.16.2609.KX505536

LPPLDRLTL

>B.US.79.NYC12.KJ704789

LPPIERLTL

>B.US.79.NYC4.KJ704792

LPPLERLTL

>B.US.83.RF_HAT3.M17451

LPPLERLTL

>B.US.83.SF2_LAV2_ARV2.K02007

LPPLERLTL

>B.US.84.5019_84.AY835779

LPPLERLTL

>B.US.84.MNCG_MN.M17449

LPP-QRLTL

>B.US.84.NY5CG.M38431

LPPLERLTL

>B.US.84.SC.M17450

LPPLERLTL

>B.US.84.SF33.AY352275

LPPLDRLTL

>B.US.85.5077_85.AY835769

LPPLERLTL

>B.US.85.Ba_L.AB221005

LPPLERLTL

>B.US.86.5096_86.AY835749

LPPLERLTL

>B.US.86.AD87_ADA.AF004394

LPPLERLTL

>B.US.86.YU_2.M93258

LPPLERLTL

>B.US.87.5113_87.AY835758

LPPLEKLTL

>B.US.88.WR27.AF286365

LPPLERLTL

>B.US.89.P896_89_6.U39362

LPPLEKLTL

>B.US.90.90US_873.AY713412

LPPLERLTL

>B.US.90.US1.AY173952

LPPLERLTL

>B.US.90.US2.AY173953

LPPLERLTL

>B.US.90.US4.AY173955

LPPLERLTL

>B.US.90.WEAU160_GHOSH.U21135

LPPLDRLTL

>B.US.91.5048_91.AY835761

LPPLERLTI

>B.US.91.DH12_3.AF069140

LPPLERLTL

>B.US.91.R3B.AY608576

LPPLERLTL

>B.US.91.SUMA_TF1.JN944928

LPPLERLTI

>B.US.92.92US657_1_301657_1.U04908

LPPIERLTL

>B.US.93.WCD32P0793.DQ487188

LPPLDRLTL

>B.US.94.5082_94.AY835773

LPPLERLTL

>B.US.94.94US_33931N.AY713410

LPPLERLTL

>B.US.94.PRB926_04_A9_4237.EU289197

LPPLERLTL

>B.US.95.5073_95.AY835768

LPPLERLTL

>B.US.95.6240_08_TA5_4622.EU289190

LPPLERLTL

>B.US.95.PRB931_06_TC3_4930.EU289198

LPPLERLTL

>B.US.95.Pt9_1995_9.KU678164

LPPLERLTL

>B.US.96.1027_03.AY332237

LPPLERLSL

>B.US.96.1057_01.AY331292

LPPLERLTL

>B.US.96.1333_d2.AY308760

LPPLERLTL

>B.US.96.6244_13_B5_4576.EU289191

LPPLERLTL

>B.US.96.USPI55751EI32y96071pcWG2B15.JN024428

LPPLERLSL

>B.US.97.1001_07.AY331282

LPPLERLTL

>B.US.97.1006_08.AY331284

LPPLERLTL

>B.US.97.1013_03.AY331287

LPPLERLTL

>B.US.97.1018_06.AY331289

LPPLERLTL

>B.US.97.1051_11.KT124744

LPELERLTL

>B.US.97.1053_06.KT124745

LPPLERLTL

>B.US.97.395718.KT124770

LPPLERLTL

>B.US.97.63358_p3_4013.EU289192

LPPLERLTL

>B.US.97.ARES2.AB078005

LPPLERLTL

>B.US.97.ZP6248_07_3A1.JN400469

LPSLERLTL

>B.US.98.1056_TA11_1826.EU289186

LPQLERLTL

>B.US.98.1058_08.AY331294

LPPIERLTL

>B.US.98.1059_09.KT124746

LPPLERLTL

>B.US.98.119534.KT124747

LPPLERLTL

>B.US.98.15384_1.DQ853463

LPPLERLTL

>B.US.98.394242.KT124769

LPPFERLTI

>B.US.98.9021_14_B2_4571.EU289196

LPPLERLTL

>B.US.98.98USHVTN1925c1.AY560107

LPPLERLTL

>B.US.98.98USHVTN3605c9.AY560108

LPPLERLTL

>B.US.98.98USHVTN8229c6.AY560109

LPPLERLTL

>B.US.98.98USHVTN941c1.AY560110

LPPLERLTL

>B.US.98.WC3_0498_4.EF175212

LPPLERLNL

>B.US.99.169_1999.JN599165

LPPIDRLTL

>B.US.99.284160.KT124759

LPPLDRLTL

>B.US.99.489910.KT124776

LPPLERLTL

>B.US.99.868558.KT124798

LPPLERLTL

>B.US.99.AD17_3C_TA4.GU331247

LPPLERLTL

>B.US.99.HIV_US_BID_V5239_1999.JQ403100

LPPLERLTL

>B.US.99.PRB959_03.AY331296

LPPLERLTL

>B.US.x.1229I.FJ469685

LPPLERLTI

>B.US.x.AC160_T9_Day_1034_Dom.EU616649

LPPLERLTL

>B.US.x.AC_16_0_Days_Consen_fa.DQ127537

LPPLERLTL

>B.US.x.AC_59_41_Days_Seq1_fa.DQ127548

LPPLERLTL

>B.US.x.CR0036W.FJ469690

LPPLERLTL

>B.US.x.CR0050Z.FJ469692

LPPLERLTL

>B.US.x.CR0059T.FJ469694

LPPLERLTL

>B.US.x.CR0127W.FJ469699

LPPLERLTL

>B.US.x.CR0164U.FJ469702

LPSLERLTL

>B.US.x.CR0192W.FJ469704

LPPLDRLTL

>B.US.x.CR0206U.FJ469705

LPPIERLTL

>B.US.x.CR0234.FJ469711

LPPLERLTL

>B.US.x.CR0248X.FJ469712

LPPLERLTL

>B.US.x.CR0289Z.FJ469715

LPPLERLTL

>B.US.x.CR0317N.FJ469719

LPALERLTI

>B.US.x.CR0382N.FJ469725

LPPLERLTL

>B.US.x.CR0413T.FJ469726

LPPLERLTL

>B.US.x.F701.FJ469727

LPPLERLTL

>B.US.x.F703.DQ886031

LPPLERLTL

>B.US.x.F710.FJ469729

LPPLERLTL

>B.US.x.F7174.DQ886032

LPPIGRLTL

>B.US.x.F7204.DQ886033

LPPLERLTL

>B.US.x.L8146.DQ886034

LPPLERLTL

>B.US.x.L8185.DQ886036

LPPLERLTL

>B.US.x.L8188.FJ469744

LPPLERLTL

>B.US.x.L896.FJ469750

LPPLDRLTL

>B.US.x.MACS2BR_13.AF491737

LPPLERLTL

>B.US.x.NC7.AF049495

LPPLERLTL

>B.US.x.NCQ.FJ469752

LPPLERLTL

>B.US.x.PRLS05.FJ469756

LPPLERLTL

>B.US.x.PRLS17.FJ469761

LPPLERLTL

>B.US.x.PRLS19.FJ469763

LPPLERLTL

>B.US.x.PRLS28.FJ469766

LPPLERLTL

>B.US.x.SH8127.FJ469767

LPPLERLTL

>B.US.x.SH8183.FJ469768

LPPLERLTL

>B.US.x.SH8229.FJ469769

LPPIERLNL

>B.US.x.SH8241.FJ469771

LPPLERLTL

>B.US.x.sample_C_BID_D617.JX503075

LPPLERLTL

>B.UY.01.01UYTRA1092.AY781126

LPPLERLTL

>B.UY.01.01UYTRA1179.AY781127

LPPLERLTL

>B.UY.02.02UY_TSU1290.JN235958

LPPLERLTL

>B.UY.99.99UY_TRA0177.JN235965

LPPLERLTL

>B.YE.02.02YE507.AY795904

LPPLERLTL

>B.YE.02.02YE508.AY795905

LPPLERLTL

>B.ZA.00.TV047.KJ948657

LPPLERLTL

>B.ZA.02.TV1057.KJ948660

LPPLERLTL

>B.ZA.03.03ZAPS045MB2.DQ396398

LPPLERLTL

>B.ZA.09.DEMB09ZA022.KP109515

LPQIERLTI

>B.ZA.85.R68.MH234643

LPPLERLTL

>B.ZA.86.R1296.MH234639

LPPLERLSL

>B.ZA.87.R459.MH234640

LPPLERLTL

>B.ZA.98.TV016.KJ948656

LPPLERLTL

>B.x.00.LA02FolC.KU168257

LPPLERLTL

>B.x.02.LA03BlEr.KU168258

LPPLERLNL

>B.x.02.LA04GuFu.KU168259

LPPLGRLTL

>B.x.03.LA05MeAl.KU168260

LPPLERLTL

>B.x.10.DEURF10HA002.KY658703

LPPLERLTL

>C.AR.01.ARG4006.AY563170

LPPIERLNI

>C.BR.02.02BR2022.JN692434

LPPIERLNI

>C.BR.04.04BR013.AY727522

LPPIERLNI

>C.BR.04.04BR021.AY727523

LPPIEGLTI

>C.BR.04.04BR038.AY727524

LPPIEGLNI

>C.BR.04.04BR073.AY727525

LPPIERLTI

>C.BR.07.DEMC07BR003.JX140663

LPPIERLSI

>C.BR.09.DEMC09BR036.KU749391

LPPIERLSI

>C.BR.10.10BR_MG032.KT427678

LPPIERLTI

>C.BR.10.10BR_MG040.KT427674

LPPIERLNI

>C.BR.10.10BR_MG050_2.KT427843

LPPIERLTI

>C.BR.10.10BR_PE023.KT427736

LPPIERLDI

>C.BR.10.10BR_SP033.KT427810

LPPIERLHI

>C.BR.10.10BR_SP041.KT427806

LPPLERLNI

>C.BR.10.10BR_SP060.KT427800

LPPIERLTI

>C.BR.10.DEMC10BR024.KU749392

LPPIERLNI

>C.BR.11.DEMC11BR035.KU749393

LPPIERLTI

>C.BR.92.BR025_d.U52953

LPPIERLNI

>C.BR.98.98BR004.AF286228

LPPLERLTI

>C.BW.00.00BW07621.AF443088

LPPIERLHI

>C.BW.00.00BW076820.AF443089

LPPIERLHI

>C.BW.00.00BW087421.AF443090

LPPIERLHI

>C.BW.00.00BW147127.AF443091

LPPLERLHL

>C.BW.00.00BW16162.AF443092

LPPIERLHI

>C.BW.00.00BW1686.AF443093

LPPIERLHI

>C.BW.00.00BW17593.AF443094

LPPIERLDI

>C.BW.00.00BW17732.AF443095

LPPIERLHI

>C.BW.00.00BW17956.AF443097

LPPLERLHI

>C.BW.00.00BW18113.AF443098

LPPIERLHI

>C.BW.00.00BW18802.AF443100

LPPIERLHI

>C.BW.00.00BW192113.AF443101

LPPIERLNI

>C.BW.00.00BW20636.AF443103

LPPIERLRI

>C.BW.00.00BW20872.AF443104

LPPIERLNI

>C.BW.00.00BW2127214.AF443105

LPPIERLNI

>C.BW.00.00BW38193.AF443108

LPPIERLNI

>C.BW.00.00BW38428.AF443109

LPPIERLNI

>C.BW.00.00BW38713.AF443110

LPPIERLHI

>C.BW.00.00BW3886_8.AF443112

LPPIERLHI

>C.BW.00.00BW3970_2.AF443114

LPPLERLHI

>C.BW.00.00BW5031_1.AF443115

LPPLERLHI

>C.BW.00.DEMC00BW010.KY658704

LPPIERLHI

>C.BW.00.DEMC00BW013.KY658705

LPPIERLYL

>C.BW.00.DEMC00BW014.KY658706

LPPIERLNL

>C.BW.10.mpp_00063_amp2.KR861313

LPPIERLHI

>C.BW.10.mpp_00115_amp2.KR861315

LPPIERLHI

>C.BW.10.mpp_00117_amp2.KR861316

LPPIERLHI

>C.BW.10.mpp_00120_amp2.KR861317

LPPIERLTI

>C.BW.10.mpp_00197_amp2.KR861325

LPPIERLHI

>C.BW.10.mpp_00205_amp2.KR861326

LPPIERLHI

>C.BW.10.mpp_00241_amp2.KR861329

LPPIERLHI

>C.BW.10.mpp_00270_amp2.KR861331

LPPIERLHI

>C.BW.10.mpp_00307_amp2.KR861333

LPPIERLHI

>C.BW.10.mpp_00361_amp2.KR861337

LPPIERLHI

>C.BW.10.mpp_00380_amp2.KR861340

LPPIERLNI

>C.BW.10.mpp_00489_amp2.KR861345

LPPIERLHI

>C.BW.11.mpp_00038_amp2.KR861312

LPPIEGLHI

>C.BW.11.mpp_00088_amp2.KR861314

LPPIERLSI

>C.BW.11.mpp_00145_amp2.KR861320

LPPIERLHI

>C.BW.11.mpp_00157_amp2.KR861321

LPPIEGLHI

>C.BW.11.mpp_00160_amp2.KR861322

LPPIERLHI

>C.BW.11.mpp_00168_amp2.KR861323

LPPIERLDI

>C.BW.11.mpp_00188_amp2.KR861324

LPPIERLHI

>C.BW.11.mpp_00259_amp2.KR861330

LPPIERLYI

>C.BW.11.mpp_00286_amp2.KR861332

LPPIERLHI

>C.BW.11.mpp_00354_amp2.KR861336

LPPIEKLHI

>C.BW.11.mpp_00373_amp2.KR861339

LPPIERLHI

>C.BW.11.mpp_00437_amp2.KR861341

LPPIERLNI

>C.BW.11.mpp_00454_amp2.KR861342

LPPIERLNI

>C.BW.12.mpp_00037_amp2.KR861311

LPPIERLHI

>C.BW.12.mpp_00144_amp2.KR861319

LPPLERLHI

>C.BW.12.mpp_00219_amp2.KR861327

LPPIEKLHI

>C.BW.12.mpp_00478_amp2.KR861344

LPPIERLHI

>C.BW.13.bcpp_00147_amp2.KR861270

LPPIERLHI

>C.BW.13.mpp_00238_amp2.KR861328

LPPIERLNI

>C.BW.14.bcpp_00030_amp2.KR861257

LPPIERLHI

>C.BW.14.bcpp_00048_amp2.KR861259

LPPIERLHI

>C.BW.14.bcpp_00090_amp2.KR861264

LPPIERLHI

>C.BW.14.bcpp_00127_amp2.KR861269

LPPLERLHI

>C.BW.14.bcpp_00155_amp2.KR861271

LPPIDRLYI

>C.BW.14.bcpp_00166_amp2.KR861273

LPPIERLHI

>C.BW.14.bcpp_00180_amp2.KR861276

LPPIERLHI

>C.BW.14.bcpp_00201_amp2.KR861280

LPPIERLHI

>C.BW.14.bcpp_00204_amp2.KR861281

LPPIERLHI

>C.BW.14.bcpp_00207_amp2.KR861282

LPPIERLHI

>C.BW.14.bcpp_00211_amp2.KR861283

LPPIEGLHI

>C.BW.14.bcpp_00395_amp2.KR861302

LPPIERLHI

>C.BW.14.bcpp_00403_amp2.KR861304

LPPIERLHI

>C.BW.14.bcpp_00460_amp2.KR861308

LPPIERLHI

>C.BW.96.96BW01.AF110960

LPPIERLHI

>C.BW.96.96BW06K18.AF290030

LPPIERLHI

>C.BW.96.96BW15B03.AF110973

LPPIERLNI

>C.BW.96.96BW17.AF110979

LPPLERLHL

>C.BW.96.96BWM032.AF443075

LPPIERLSI

>C.BW.96.DEMC96BW001.KY658707

LPPIERLHI

>C.BW.96.DEMC96BW002.KY658708

LPPIERLHI

>C.BW.96.DEMC96BW006.KY658709

LPPIERLHI

>C.BW.96.DEMC96BW007.KY658710

LPPIERLHI

>C.BW.96.DEMC96BW009.KY658711

LPPLERLHI

>C.BW.98.98BWMC122.AF443076

LPPIERLHI

>C.BW.98.98BWMC134.AF443077

LPPIERLHI

>C.BW.98.98BWMO1410.AF443079

LPPIERLNI

>C.BW.98.98BWMO36A5.AF443081

LPPIERLHI

>C.BW.98.98BWMO37D5.AF443082

LPPIERLHI

>C.BW.99.99BW393212.AF443083

LPPIERLNI

>C.BW.99.99BW46424.AF443084

LPPIERLHI

>C.BW.99.99BW4745.AF443085

LPPLERLHI

>C.BW.99.99BW47547.AF443086

LPPIEGLHI

>C.BW.99.99BWMC168.AF443087

LPPIERLNI

>C.BW.x.mpp_00140_amp2.KR861318

LPPIERLHI

>C.CN.06.06YNLC028sg.KC898980

LPPLERLRI

>C.CN.06.06YNLC32sg.KC899004

LPPIERLNI

>C.CN.07.07CNYN328.KF835515

LPPIERLHI

>C.CN.07.07CNYN336.KF835522

LPPLERLHI

>C.CN.09.09YNLX090sg.KC898995

LPPLERLNI

>C.CN.09.09YNLX133sg.KC898996

LPPIKKLHI

>C.CN.09.DH21.KF250403

LPPIERLHI

>C.CN.09.DH22.KF250404

LPPIERLHI

>C.CN.10.YNFL19.KC870038

LPPLERLHI

>C.CN.98.YNRL9840.AY967806

LPPIERLHI

>C.CY.05.CY040.FJ388901

IPPIERLHI

>C.CY.05.CY069.FJ388913

LPPIERLHL

>C.CY.06.CY166.FJ388948

LPPIERLHI

>C.CY.06.CY176.FJ388952

LPPIERLNL

>C.CY.07.CY187.JF683740

LPPIERLHI

>C.CY.07.CY203.JF683755

LPPIERLHI

>C.CY.07.CY205.JF683757

LPPIERLHI

>C.CY.08.CY219.JF683768

LPPIERLHI

>C.CY.08.CY221.JF683770

LPPIERLHI

>C.DE.10.622166.KT124786

LPPIERLHI

>C.DJ.91.DJ259A.L22940

LPPIERLNL

>C.DJ.91.DJ373A.L23065

LPPIERLNL

>C.DK.01.CTL_015.EF514713

LPPIERLHI

>C.ES.06.X1936.EU786673

LPPIERLHI

>C.ES.07.Read4_HIV_C.KX228820

LPPIERLHI

>C.ES.08.X2363_2.EU786681

LPPIERLYI

>C.ES.14.ARP1198.KT276258

LPPIERLHI

>C.ES.15.100_118.KY496624

LPPIERLTI

>C.ET.02.02ET_288.AY713417

LPPLERLNL

>C.ET.08.ET106.KU319529

LPPLERLTL

>C.ET.08.ET108.KU319530

LPPIERLNL

>C.ET.08.ET115.KU319531

LPPLERLNL

>C.ET.08.ET119.KU319532

LPPLERRTL

>C.ET.08.ET122.KU319533

LPPIERLHI

>C.ET.08.ET124.KU319534

LPPIERLNI

>C.ET.08.ET126.KU319535

LPPLERLHI

>C.ET.08.ET128.KU319536

LPPIERLNI

>C.ET.08.ET130.KU319537

LPPIERLTL

>C.ET.08.ET135.KU319538

LPPIERLTL

>C.ET.08.ET136.KU319539

LPPIEGRHI

>C.ET.08.ET145.KU319540

LPPIERLTI

>C.ET.08.ET147.KU319541

LPPIERLTL

>C.ET.08.ET148.KU319542

LPPIERLHI

>C.ET.08.ET149.KU319543

LPPIERLNL

>C.ET.08.ET150.KU319544

LPPIERLHI

>C.ET.08.ET154.KU319545

LPPLERLTL

>C.ET.08.ET155.KU319546

LPPIERLHI

>C.ET.08.ET159.KU319547

LPPIERLNL

>C.ET.08.ET164.KU319548

LPPIERLRI

>C.ET.08.ET165.KU319549

LPPIERLNL

>C.ET.08.ET167.KU319550

LPPIERLHI

>C.ET.08.ET171.KU319551

LPPLERLTL

>C.ET.86.ETH2220.U46016

LPPLERLNL

>C.FI.91.FIN9149.AF219263

LPPIERLHL

>C.FI.91.FIN9158.AF219264

LPPIERLHI

>C.FI.92.FIN9277.AF219269

LPPIERLTI

>C.FI.93.FIN9307.AF219270

LPPIERLHI

>C.FI.94.FIN9401.AF219274

LPPIERLHI

>C.GB.14.14535_1_8.3.MF109535

LPPIERLHI

>C.GE.03.03GEMZ033.DQ207941

LPPIERLHI

>C.IL.98.98IS002.AF286233

LPPIERLHI

>C.IL.99.99ET17.AY255826

LPPIERLHI

>C.IL.99.99ET1.AY255823

LPPIDRLTL

>C.IL.99.99ET7.AY255824

LPPLERLHI

>C.IN.00.DEMC00IN005.KP109480

LPPIERLHI

>C.IN.00.DEMC00IN006.KP109481

LPPIERLHI

>C.IN.00.DEMC00IN007.KP109482

LPPIERLHI

>C.IN.00.DEMC00IN008.KP109483

LPPIERLHI

>C.IN.00.DEMC00IN009.KP109484

LPPIERLHI

>C.IN.00.DEMC00IN010.KP109485

LPPIERLNI

>C.IN.03.D24.EF469243

LPPIDRLNI

>C.IN.04.C.IN.04.NIRT379.1.KF766537

LPPIERLRI

>C.IN.05.C.IN.05.NIRT723.1.KF766541

LPPIERLHI

>C.IN.07.NARI_VB52_J19.EU521727

LPPIERLHI

>C.IN.09.T125_2139.KC156210

LPPIERLHI

>C.IN.11.NIRT009.KX069227

LPPLERLHI

>C.IN.11.NIRT010.KX069228

LPPIEGLHI

>C.IN.14.NIRT004.KX069222

LPPIERLHI

>C.IN.15.NIRT001.KX069219

LPPIERLYI

>C.IN.15.NIRT002.KX069220

LPPIERLHI

>C.IN.15.NIRT003.KX069221

LPPIERLHI

>C.IN.15.NIRT005.KX069223

LPPIERLHI

>C.IN.15.NIRT006.KX069224

LPPIERLTL

>C.IN.15.NIRT007.KX069225

LPPIERLTI

>C.IN.15.NIRT008.KX069226

LPPIERLHI

>C.IN.15.SC007.KY713228

LPPIERLNI

>C.IN.15.SC008.KY713229

LPPIERLHI

>C.IN.15.SC013.KY713230

LPPIERLHI

>C.IN.15.SC015.KY713231

LPPIERLNI

>C.IN.15.SC022.KY713233

LPPIERLHI

>C.IN.15.SC062.KY713234

LPPLERLHI

>C.IN.15.SC085.KY713235

LPPIERLHI

>C.IN.15.SHE001.KY713236

LPPIERLHI

>C.IN.93.93IN999.AF067154

LPPLERLHI

>C.IN.94.94IN11246.AF067159

LPPIERLHI

>C.IN.94.94IN476.AF286223

LPPIERLHI

>C.IN.94.94IN_20635_4.AY713414

LPPIERLHI

>C.IN.95.95IN21068.AF067155

LPPIERLHI

>C.IN.98.98IN012.AF286231

LPPIERLNI

>C.IN.98.98IN022.AF286232

LPPLERLHI

>C.IN.99.01IN565_10.AY049708

LPPIERLHI

>C.IN.99.DEMC99IN001.KP109486

LPPIERLHI

>C.IN.99.DEMC99IN002.KP109487

LPPIERLHI

>C.IN.99.DEMC99IN003.KP109488

LPPIERLNI

>C.IN.99.DEMC99IN004.KP109489

LPPIERLHI

>C.IN.x.VB39.EF694033

LPPIERLHI

>C.IN.x.VB49.EF694036

LPPLERLHI

>C.KE.00.KER2010.AF457054

LPPIERLHI

>C.KE.04.04KE344827V3.KT022362

LPPIERLTI

>C.KE.04.04KE585573V2.KT022366

LPPLERLHI

>C.KE.05.05KE369195V4.KT022371

LPPIERLSL

>C.KE.91.KNH1268.AY945738

LPPIERLNL

>C.KE.97.QC406_70M_ENV_F3.FJ866133

LPPIERLTL

>C.MM.99.mIDU101_3.AB097871

LPPIDRLHI

>C.MW.03.0626_E6.KC894075

LPPIERLCI

>C.MW.03.0665_F2.KC894076

LPPIERLYI

>C.MW.03.0682_E4.KC894077

LPPIERLHI

>C.MW.04.0985_H7.KC894078

LPPIERLYI

>C.MW.04.1086_B2.KC894079

LPPIERLHI

>C.MW.04.1176_A3.KC894081

LPPIERLHI

>C.MW.04.1394_C9.KC894082

LPPIERLHI

>C.MW.05.2010_F5.KC894083

LPPIERLHI

>C.MW.05.2060_G9.KC894085

LPPIERLHL

>C.MW.05.3003_2_D3.KC894087

LPPIERLHI

>C.MW.05.3011_2_F4.KC894089

LPPIERLDI

>C.MW.05.3022_2_A2.KC894091

LPPIERLHI

>C.MW.05.3025_2_C3.KC894092

LPPIERLHI

>C.MW.05.3026_2_H9.KC894093

LPPIERLHI

>C.MW.05.3027_2_F11.KC894094

LPPIERLHI

>C.MW.05.3029_2_E2.KC894095

LPPIERLHI

>C.MW.05.3036_2_C12.KC894097

LPPIERLHI

>C.MW.05.3037_6.KC894098

LPPIERLHI

>C.MW.05.3038_1.KC894384

LPPIERLCI

>C.MW.05.3040_2_C8.KC894100

LPPIERLHI

>C.MW.05.3042_2_C9.KC894101

LPPIERLSI

>C.MW.05.3048_2_E3.KC894103

LPPIERLYI

>C.MW.05.3050_2_C3.KC894385

LPPIERLHI

>C.MW.06.2052_G10.KC894084

LPPIERLHI

>C.MW.07.702010141_CH141.w12.KC156212

LPPIERLTI

>C.MW.07.703010054_2A2.KC894106

LPPIERLHI

>C.MW.07.703010085_CH085.w4a.KC156211

LPPLEGLSI

>C.MW.07.703010131_CH131_TF.KC156114

LPPLERLCI

>C.MW.07.703010167_CH167.w8.KC156213

LPPIERLYI

>C.MW.07.703010200_CH200_TFc.KC156119

LPPIERLYI

>C.MW.07.703010217_B6.KC894109

LPPIERLHI

>C.MW.07.703010228_CH228_TFa.KC156120

LPPLERLAI

>C.MW.07.CH0010.w24.e11.KC148599

LPPIERLNI

>C.MW.07.CH0047.w2.e11.KC148663

LPPIERLHL

>C.MW.07.DEMC07MW001.KP109520

LPPIERLHI

>C.MW.08.4403bmLwk4_fl11.KF527172

LPPIERLSI

>C.MW.08.702010293_CH293.w8a.KC156216

LPPIERLHI

>C.MW.08.702010432_CH432.w4.KC156218

LPPIEGLHI

>C.MW.08.702010440_CH440.w4.KC156219

LPPIERLHI

>C.MW.08.703010269_CH269.w24.KC156215

LPPIERLSI

>C.MW.08.CH0275.w24.e1.KC149493

LPPIERLHI

>C.MW.08.CH427.PL.011608.UT.5.KY112213

LPPIERLHI

>C.MW.08.CH455.PL.012908.UT.1.KY112254

LPPLERLNI

>C.MW.08.DEMC08MW002.KP109521

LPPIERLHI

>C.MW.08.DEMC08MW004.KP109522

LPPLERLHI

>C.MW.09.703010256_CH256.w96.KC156214

LPPIERLHI

>C.MW.09.CH1064.PL.080409.A2.08.KY111994

LPPIERLSI

>C.MW.09.DEMC09MW006.KP109523

LPPIERLYI

>C.MW.09.DEMC09MW007.KP109524

LPPLERLHI

>C.MW.09.DEMC09MW008.KP109525

LPPIERLHI

>C.MW.09.DEMC09MW009.KP109526

LPPIERLHI

>C.MW.09.DEMC09MW010.KP109527

LPPLERLHI

>C.MW.10.CH0752_3_d0614_ipe023_7_15.MG901705

LPPIERLSI

>C.MW.11.703011244.3.d0847.ipe015.3.13.MF500628

LPPIERLHI

>C.MW.11.CH0694_3_d1009_ipe018_15_49.MG898649

LPPIERLAI

>C.MW.12.CH1754.3.d0832.ipe021_15.20.MF502153

LPPIEGLHI

>C.MW.13.CH1012_3_d1655_ipe017_180_10.MG899863

LPPIERLTI

>C.MW.96.C034_B2.KC894127

LPPIERLYI

>C.MW.96.C047_H7_3B.KC894128

LPPIERLHI

>C.MW.96.C109_D2.KC894131

LPPIERLNI

>C.MW.96.C111_H2.KC894132

LPPIERLAI

>C.NG.10.10NG020523.KX389612

LPPIERLHI

>C.NP.00.11NP115.KU341731

LPPIERLHI

>C.NP.07.11NP091.KU341730

LPPIERLNI

>C.NP.08.11NP004.KU341722

LPPIERLHI

>C.NP.08.11NP084.KU341728

LPPIERLNI

>C.NP.09.11NP076.KU341726

LPPIERLHI

>C.NP.10.11NP065.KU341725

LPPLERLHI

>C.NP.11.11NP014.KU341723

LPPIEGLHI

>C.NP.11.11NP016.KU341724

LPPIERLNI

>C.PK.14.DEMC14PK009.KU749412

LPPIERLAI

>C.SE.03.004ZM.MF373126

QPPIERLHI

>C.SE.04.006CD.MF373128

LPPIERLHI

>C.SE.05.010BI.MF373131

LPPIERLTL

>C.SE.05.011BI.MF373132

LPPIERLTI

>C.SE.05.SE600415.KP411838

LPPIERLHI

>C.SE.06.013ET.MF373134

LPSLERLNL

>C.SE.06.014ET.MF373135

LPPIERLHI

>C.SE.06.016SE.MF373136

LPPIERLHI

>C.SE.06.017BI.MF373137

LPPIERLDI

>C.SE.06.018SE.MF373138

LPPIERLTL

>C.SE.06.019KE.MF373139

LPPIERLTL

>C.SE.06.SE600516.KP411839

LPPIEDLHI

>C.SE.07.SE600108.KP411830

LPPIERLTL

>C.SE.07.SE600119.KP411831

LPPIERLHI

>C.SE.07.SE600122.KP411832

LPPLERLHI

>C.SE.08.SE600210.KP411833

LPPIERLSL

>C.SE.08.SE600213.KP411834

FPPMERLIL

>C.SE.09.031ER.MF373145

LPPIERLNL

>C.SE.09.033ET.MF373146

LPPIERLTL

>C.SE.09.035ZA.MF373148

LPPIERLSL

>C.SE.09.SE600314.KP411836

LPPIERLHI

>C.SE.10.041SE.MF373152

LPPIERLNL

>C.SE.10.043SE.MF373154

LPPLERLTI

>C.SE.10.SE600412.KP411837

LPPLERLTL

>C.SE.11.053SE.MF373156

LPPIERLSI

>C.SE.11.054ET.MF373157

LPEIERLHI

>C.SE.11.055ER.MF373158

LPPIERLHI

>C.SE.11.056IT.MF373159

LPPLERLTL

>C.SE.11.061SO.MF373164

LPPIERLNI

>C.SE.11.062ZA.MF373165

LPPIERLHI

>C.SE.11.063BI.MF373166

LPPLERLTL

>C.SE.12.066SE.MF373169

LPPLERLNI

>C.SE.12.067SE.MF373170

LPPIERLTL

>C.SE.12.068SE.MF373171

LPPIERLTL

>C.SE.12.072SO.MF373175

LPPIERLTL

>C.SE.12.073NP.MF373176

LPPIEGLHI

>C.SE.12.074SE.MF373177

LPPIERLNL

>C.SE.12.075ZA.MF373178

LPPIERLHI

>C.SE.12.076SO.MF373179

PPPLERLNR

>C.SE.13.085BI.MF373187

LPPIERLTI

>C.SE.13.090ET.MF373192

LPPLERLTL

>C.SE.13.SE600311.KP411835

LPRMERFIL

>C.SE.14.091SE.MF373193

LPPIEGLHI

>C.SE.14.095CD.MF373197

LPPIERLHI

>C.SE.14.096SE.MF373198

LPPIERLTL

>C.SE.14.097SO.MF373199

LPPLERLHI

>C.SE.15.100ET.MF373202

LPPIERLTL

>C.SN.90.90SE_364.AY713416

LPPIERLHI

>C.SN.96.5056135.MH705137

LPPIERLYI

>C.TZ.01.A125.AY253304

LPPIERLHI

>C.TZ.01.A246.AY253308

LPPIDRLHI

>C.TZ.01.A301.AY253312

LPPIERLHI

>C.TZ.01.A388.AY253317

LPPIERLHI

>C.TZ.01.A93.AY253303

LPPIERLHI

>C.TZ.01.BD16_10.AY253320

LPPIERLSI

>C.TZ.01.BD22_11.AY253321

LPPIERLHI

>C.TZ.01.BD39_8.AY253313

LPPIERLHI

>C.TZ.01.BD9_11.AY253322

LPPIERLHI

>C.TZ.02.CO178.AY734556

LPPLERLHI

>C.TZ.02.CO3056.AY734550

LPPIEGLHI

>C.TZ.02.CO328.AY734551

LPPIERLHI

>C.TZ.02.CO3305.AY734558

LPPIERLHI

>C.TZ.02.CO6130.AY734559

LPPLERLHL

>C.TZ.02.CO6721.AY734560

LPPIERLHI

>C.TZ.03.CO0077V1.KX907339

LPPIERLHI

>C.TZ.03.CO0921V2.KX907356

LPPIERLHI

>C.TZ.03.CO0984V2.KX907358

LPPIERLHI

>C.TZ.03.CO3873V1.KX907388

LPPIEGLHI

>C.TZ.03.CO6471V1.KX907407

LPPIERLNI

>C.TZ.03.CO6838V1.KX907424

LPPIERLHI

>C.TZ.03.CO6854V1.KX907426

LPPIGRLHI

>C.TZ.03.CO6980V0.KX907433

LPPLERLHI

>C.TZ.04.CO0041V3.KX907337

LPPLXRLHI

>C.TZ.04.CO0377V4.KX907345

LPPIERLTI

>C.TZ.04.CO3728V2.KX907384

LPPIERLHI

>C.TZ.04.CO3979V3.KX907392

LPPIERLHI

>C.TZ.04.CO6033V4.KX907394

LPPIERLHI

>C.TZ.04.CO6040V4.KX907395

LPPIERLHI

>C.TZ.04.CO6322V4.KX907405

LPPIERLHI

>C.TZ.04.CO6631V3.KX907413

LPPIERLHI

>C.TZ.04.CO6644V2.KX907415

LPPIEGLHI

>C.TZ.05.CO0053V5.KX907338

LPPIERLNI

>C.TZ.05.CO0933V4.KX907357

LPPIERLHI

>C.TZ.05.CO3063V5.KX907363

LPPIERLNI

>C.TZ.05.CO3168V4.KX907366

LPPIERLYI

>C.TZ.05.CO3426V5.KX907374

LPPIERLNI

>C.TZ.05.CO3637V5.KX907380

LPPLERLHI

>C.TZ.05.CO3672V4.KX907382

LPPIERLYI

>C.TZ.05.CO3800V5.KX907385

LPPIERLHI

>C.TZ.05.CO6106V5.KX907398

LPPLERLHI

>C.TZ.05.CO6647V5.KX907416

LPPIERLTI

>C.TZ.05.CO6785V5.KX907420

LPPIERLHI

>C.TZ.06.CO3278V6.KX907368

LPPIERLHI

>C.TZ.06.CO3440V7.KX907375

LPPIERLHI

>C.TZ.06.CO3892V6.KX907390

LPPIDRLHI

>C.TZ.06.CO3969V6.KX907391

LPPIERLHI

>C.TZ.06.CO6022V7.KX907393

LPPIERLNI

>C.TZ.06.CO6139V7.KX907399

LPPLERLNI

>C.TZ.06.CO6146V7.KX907400

LPPIERLHI

>C.TZ.06.CO6847V7.KX907425

LPPIERLHI

>C.TZ.08.707010457_CH457.w8.KC156220

LPPIERLHI

>C.TZ.97.97TZ05.AF361875

LPPIERLHI

>C.TZ.98.98TZ013.AF286234

LPPIERLYI

>C.TZ.98.98TZ017.AF286235

LPPIERLHI

>C.UG.90.UG268A2.L22948

LPPLERLTI

>C.US.11.17TB4_4G8.KF526226

LPPIERLNL

>C.US.14.M0118v1d14_5M04_C5.KT982201

LPPIERLHI

>C.US.98.98US_MSC3018.AY444800

LPPLERLNI

>C.US.98.98US_MSC5016.AY444801

LPPIERLHI

>C.UY.01.TRA3011.AY563169

LPPIERLNI

>C.YE.02.02YE511.AY795906

LPPIERLHI

>C.ZA.00.1119MB.AY463229

LPPIERLHI

>C.ZA.00.1134MB.AY463217

LPPIEGLHI

>C.ZA.00.1157M3M.AY585266

LPPIERLHI

>C.ZA.00.1165MB.AY463230

LPEIERLHI

>C.ZA.00.1168MB.AY463231

LPPIERLHI

>C.ZA.00.1170MB.AY463225

LPPIEKLHI

>C.ZA.00.1171MB.AY463232

LPPIERLHI

>C.ZA.00.1176MB.AY463218

LPPIERLHI

>C.ZA.00.1178MB.AY463233

LPPIERLHI

>C.ZA.00.1189MB.AY838565

LPPIERLHI

>C.ZA.00.1195MB.AY463220

LPPIERLHI

>C.ZA.00.1197MB.AY463234

LPPIERLHI

>C.ZA.00.1210MB.AY463221

LPPIEKLHI

>C.ZA.00.1214MB.AY463236

LPPIERLHI

>C.ZA.00.1217MB.AY463226

LPPIERLHI

>C.ZA.00.1225MB.AY463227

LPPIEGLHI

>C.ZA.00.1228MB.AY463222

LPPIEKLHI

>C.ZA.00.J112MA.AY838568

LPPIERLHI

>C.ZA.00.J38MA.AY463228

LPPLERLHI

>C.ZA.01.01ZATM45.AY228557

LPPIEGLHI

>C.ZA.01.2004MB.AY463235

LPPIERLAI

>C.ZA.01.2134MB.AY463237

LPPLERLHI

>C.ZA.01.J54Ma.AY463223

LPPIEKLHI

>C.ZA.02.02ZAPS001MB1.DQ275648

LPPIESLHI

>C.ZA.02.02ZAPS005MB1.DQ351235

LPPIERLHI

>C.ZA.02.02ZAPS006MB1.DQ351220

LPPIERLHI

>C.ZA.02.02ZAPS008MB1.DQ275647

LPPIERLHI

>C.ZA.02.02ZAPS013MB1.DQ351222

LPPIEELHI

>C.ZA.02.02ZAPS014MB1.DQ351218

LPPIERLHI

>C.ZA.02.02ZAPS015MB1.DQ369995

LPPIEGLHL

>C.ZA.03.03ZAPS017MB1.DQ351224

LPPIERLHI

>C.ZA.03.03ZAPS020MB1.DQ275653

LPPIERLHI

>C.ZA.03.03ZAPS021MB1.DQ369978

LPPIEKLHI

>C.ZA.03.03ZAPS023MB1.DQ351225

LPPIERLHI

>C.ZA.03.03ZAPS024MB1.DQ396367

LPPIERLNI

>C.ZA.03.03ZAPS025MB1.DQ351226

LPPIERPHI

>C.ZA.03.03ZAPS026MB1.DQ369985

LPPIERLHI

>C.ZA.03.03ZAPS030MB1.DQ369996

LPPIERLDI

>C.ZA.03.03ZAPS032MB1.DQ445633

LPPLERLHI

>C.ZA.03.03ZAPS034MB1.DQ369979

LPPIERLHI

>C.ZA.03.03ZAPS043MB1.DQ351227

LPPIERLHL

>C.ZA.03.03ZAPS044MB1.DQ396384

LPPIERLHI

>C.ZA.03.03ZAPS046MB1.DQ369984

LPPIERLHI

>C.ZA.03.03ZAPS049MB1.DQ369986

LPPIERLHI

>C.ZA.03.03ZAPS050MB1.DQ369980

LPPLERLHI

>C.ZA.03.03ZAPS052MB1.DQ369987

LPPLERLHI

>C.ZA.03.03ZAPS054MB2.DQ369988

LPPIERLHI

>C.ZA.03.03ZAPS055MB1.DQ396373

LPPIERLHI

>C.ZA.03.03ZAPS056MB1.DQ396374

LPPIENLHI

>C.ZA.03.03ZAPS059MB2.DQ445634

LPPIERLCI

>C.ZA.03.03ZAPS063MB1.DQ396388

LPPIERLHI

>C.ZA.03.03ZAPS066MB2.DQ396375

LPPIERLHI

>C.ZA.03.03ZAPS067MB2.DQ396389

PPPIERLHI

>C.ZA.03.03ZAPS071MB1.DQ396376

LPPIERLHI

>C.ZA.03.03ZAPS074MB2.DQ351228

LPPIERLHI

>C.ZA.03.03ZAPS077B1.DQ093591

LPPIEELHI

>C.ZA.03.03ZAPS083MB1.DQ351229

LPPIERLHI

>C.ZA.03.03ZAPS086MB1.DQ275654

LPPIERLYI

>C.ZA.03.03ZAPS088MB1.DQ275651

LPPIERLHI

>C.ZA.03.03ZAPS094MB1.DQ396377

LPPIEELHI

>C.ZA.03.03ZAPS095MB1.DQ275652

LPPIEKLHI

>C.ZA.03.03ZAPS097MB1.DQ351230

LPPIEGLHI

>C.ZA.03.03ZAPS099MB1.DQ275655

LPPLERLHI

>C.ZA.03.03ZAPS103MB2.DQ275656

LPPLERLHI

>C.ZA.03.03ZAPS105MB2.DQ445632

LPPIERLHI

>C.ZA.03.03ZAPS112MB2.DQ396386

LPPIERLHI

>C.ZA.03.03ZAPS116MB1.DQ445635

LPPIERLHI

>C.ZA.03.03ZAPS118MB1.DQ396368

LPPIERLHI

>C.ZA.03.03ZAPS122MB1.DQ396370

LPPIERLNI

>C.ZA.03.03ZAPS125MB1.DQ396390

LPPIERLHI

>C.ZA.03.03ZAPS126MB1.DQ275657

LPPIERLHI

>C.ZA.03.03ZAPS131MB1.DQ396380

LPPIEGLHI

>C.ZA.03.03ZAPS136MB1.DQ351231

LPPIERLHI

>C.ZA.03.03ZAPS140MB1.DQ369981

LPPLERLHI

>C.ZA.03.03ZAPS143MB1.DQ396391

LPPIERLHI

>C.ZA.03.03ZAPS151MB1.DQ396392

LPPIERLHI

>C.ZA.03.03ZAPS152MB1.DQ396399

LPPIERLHI

>C.ZA.03.03ZASK005B2.DQ011175

LPPTEKLHI

>C.ZA.03.03ZASK010B2.DQ164104

LPPIERLHI

>C.ZA.03.03ZASK011B2.AY901965

LPPIERLHI

>C.ZA.03.03ZASK013B2.DQ275660

LPPLERLHI

>C.ZA.03.03ZASK016MB2.DQ351233

LPPLERLHI

>C.ZA.03.03ZASK019B2.AY878063

LPPIERLHI

>C.ZA.03.03ZASK020B2.AY878064

LPPIERLHI

>C.ZA.03.03ZASK026B2.DQ011165

LPPIERLHI

>C.ZA.03.03ZASK036B1.AY901966

LPPIERLHI

>C.ZA.03.03ZASK039B2.AY878068

LPPIENLHI

>C.ZA.03.03ZASK058B2.AY901967

LPPIERLHI

>C.ZA.03.03ZASK061B1.AY901968

LPPIERLHI

>C.ZA.03.03ZASK062B1.DQ164113

LPPIERLHI

>C.ZA.03.03ZASK066B1.AY901969

LPPLERLTI

>C.ZA.03.03ZASK078B1.AY901971

LPPIERLHI

>C.ZA.03.03ZASK084B1.AY901981

LPPIERLYI

>C.ZA.03.03ZASK092B1.AY878057

LPPIERLHI

>C.ZA.03.03ZASK094B1.AY878070

LPPLERLTI

>C.ZA.03.03ZASK097B1.AY878060

LPPIERLHI

>C.ZA.03.03ZASK098B1.AY878061

LPPIERLHV

>C.ZA.03.03ZASK104B1.DQ396395

LPPIERLHI

>C.ZA.03.03ZASK107B1.DQ056410

LPPIERLHI

>C.ZA.03.03ZASK110B1.DQ056411

LPPIERLHI

>C.ZA.03.03ZASK111B1.DQ056404

LPPIERLHI

>C.ZA.03.03ZASK113B1.DQ351237

LPPIERLHI

>C.ZA.03.03ZASK117B1.DQ056408

LPPIERLHI

>C.ZA.03.03ZASK120B1.DQ011176

LPPIERLHI

>C.ZA.03.03ZASK211B1.DQ093601

LPPIERLHI

>C.ZA.03.03ZASK212B1.DQ093596

LPPIERLHI

>C.ZA.03.03ZASK213B1.DQ093607

LPPIERLNI

>C.ZA.03.03ZASK215M6W.DQ275661

LPPIERLHI

>C.ZA.03.03ZASK224MB1.DQ275664

LPPIERLHI

>C.ZA.03.03ZASK226B1.DQ164108

LPPIERLHI

>C.ZA.03.03ZASK232B1.DQ093589

LPPIERLHI

>C.ZA.03.03ZASK233B1.DQ351234

LPPIERLHI

>C.ZA.03.04ZAPS194MB1.DQ275650

LPPIERLHI

>C.ZA.03.04ZASK082B1.AY901972

LPPLERLHI

>C.ZA.03.503_14197_T1.KT183301

LPPIERLHI

>C.ZA.03.SK023B2.AY772690

LPPIERLHI

>C.ZA.03.SK029B2.AY772691

LPPIERLHI

>C.ZA.03.SK033B2.AY772692

LPPIERLHI

>C.ZA.03.SK040B1.AY703908

LPPIENLHI

>C.ZA.03.SK041B1.AY772693

LPPIERLHI

>C.ZA.03.SK043B1.AY772700

LPPIERLHT

>C.ZA.03.SK065B1.AY772694

LPPIERLHI

>C.ZA.03.SK091B1.AY772701

LPPIERLHI

>C.ZA.03.SK112B1.AY772695

LPPLERLHI

>C.ZA.04.04ZAPS160B1.DQ164107

LPPIEKLHI

>C.ZA.04.04ZAPS161B1.DQ164115

LPPIERLHI

>C.ZA.04.04ZAPS165MB1.DQ351221

LPPIERLHI

>C.ZA.04.04ZAPS168B1.DQ164121

LPPIERLHI

>C.ZA.04.04ZAPS169MB1.DQ396393

LPPIERLHI

>C.ZA.04.04ZAPS172MB1.DQ275659

LPPLERLHI

>C.ZA.04.04ZAPS177MB1.DQ396394

LPPIERLSI

>C.ZA.04.04ZAPS188B1.DQ164122

LPPIERLHI

>C.ZA.04.04ZAPS189B1.DQ164117

LPPIERLHI

>C.ZA.04.04ZAPS190B1.DQ093602

LPPIERLHI

>C.ZA.04.04ZAPS195B1.DQ164118

LPPIERLHI

>C.ZA.04.04ZAPS198MB1.DQ445637

LPPIERLHI

>C.ZA.04.04ZAPS199B1.DQ164110

LPPLERLCI

>C.ZA.04.04ZAPS202B1.DQ093598

LPPIERLHI

>C.ZA.04.04ZAPS205B1.DQ093599

LPPIERLTI

>C.ZA.04.04ZAPS206B1.DQ164126

LPEIERLHI

>C.ZA.04.04ZAPS216B1.DQ093600

LPPIERLHI

>C.ZA.04.04ZASK031B2.AY901974

LPPIERLHI

>C.ZA.04.04ZASK083B2.DQ093585

LPPIEKLHI

>C.ZA.04.04ZASK128B1.DQ093586

LPPIEGLHI

>C.ZA.04.04ZASK131B1.DQ056412

LPPIERLHI

>C.ZA.04.04ZASK132B1.DQ093594

LPPIERLHI

>C.ZA.04.04ZASK136B1.DQ011177

LPPIERLHI

>C.ZA.04.04ZASK139B1.AY878072

LPPIEKLHI

>C.ZA.04.04ZASK142B1.DQ011167

LPPLERLHI

>C.ZA.04.04ZASK145B1.AY901976

LPPIERLHI

>C.ZA.04.04ZASK146.AY772699

LPPIERLHI

>C.ZA.04.04ZASK147B1.DQ164114

LPPLERLHI

>C.ZA.04.04ZASK148B1.DQ093590

LPPIERLHI

>C.ZA.04.04ZASK150B1.DQ011178

LPPIERLHI

>C.ZA.04.04ZASK151B1.AY901977

LPPIERLHI

>C.ZA.04.04ZASK156B1.DQ011171

LPPIERLTI

>C.ZA.04.04ZASK159B1.DQ011179

LPPIERLHI

>C.ZA.04.04ZASK161B1.DQ011170

LPPIERLHI

>C.ZA.04.04ZASK163B1.AY901979

LPPIERLHI

>C.ZA.04.04ZASK164B1.DQ056405

LPPIERLHI

>C.ZA.04.04ZASK165B1.DQ396387

LPPIERLHI

>C.ZA.04.04ZASK168B1.AY878058

LPPIEKLHI

>C.ZA.04.04ZASK169B1.DQ396381

LPPIERLHI

>C.ZA.04.04ZASK170B1.DQ093595

LPPIERLHI

>C.ZA.04.04ZASK173B1.DQ093604

LPPIERLNI

>C.ZA.04.04ZASK174B1.AY901980

LPPIERLHI

>C.ZA.04.04ZASK175B1.DQ164129

LPPIERLHI

>C.ZA.04.04ZASK180B1.AY878059

LPPLERLHI

>C.ZA.04.04ZASK181B1.AY878062

LPPLERLHI

>C.ZA.04.04ZASK182B1.AY878054

LPPIERLHI

>C.ZA.04.04ZASK184B1.DQ056418

LPPIEGLHI

>C.ZA.04.04ZASK192B1.DQ396382

LPPIERLCI

>C.ZA.04.04ZASK193B1.DQ396396

LPPIERLHI

>C.ZA.04.04ZASK196B1.DQ056413

LPPIEGLHI

>C.ZA.04.04ZASK201B1.DQ396397

LPPIERLHI

>C.ZA.04.04ZASK202B1.DQ011180

LPPLERLHI

>C.ZA.04.04ZASK206B1.DQ056415

LPPIERLNL

>C.ZA.04.04ZASK208B1.DQ056406

LPPLERLHI

>C.ZA.04.04ZASK217B1.DQ056417

LPPIERLNI

>C.ZA.04.04ZASK234B1.DQ093605

LPPIERLSI

>C.ZA.04.CAP30_5w_F4.GQ999973

LPPIERLHI

>C.ZA.04.CAP61_8w_F3.GQ999975

LPPIERLHI

>C.ZA.04.SK133B1.AY772698

LPPIEGLHI

>C.ZA.04.SK134B1.AY703909

PPPIERLHL

>C.ZA.04.SK143B1.AY703910

LPPIERLHI

>C.ZA.04.SK144B1.AY703911

LPPIERLTI

>C.ZA.05.05ZAPSK240B1.DQ369991

LPPIERLHI

>C.ZA.05.05ZASK243B1.DQ396372

LPPLERLHI

>C.ZA.05.05ZASK244B1.DQ369992

LPPIERLHI

>C.ZA.05.05ZASK245B1.DQ369982

LPPIERLHI

>C.ZA.05.05ZASK246B1.DQ369983

LPPIEQLHI

>C.ZA.05.CAP174_4w.GQ999981

LPPIERLRI

>C.ZA.05.CAP200_B8a.KC894135

LPPIERLHI

>C.ZA.05.CAP206_8w_F1.GQ999982

LPPIERLYI

>C.ZA.05.CAP210_5w.GQ999983

LPPIERLTI

>C.ZA.05.CAP228_8w_F2.GQ999984

LPPLERLHI

>C.ZA.05.CAP244_8w_F1.GQ999986

LPPIERLHI

>C.ZA.05.CAP248_9w.GQ999987

LPPIERLHI

>C.ZA.05.CAP255_8w_F1.GQ999988

LPPIERLHI

>C.ZA.05.CAP256_6w.GQ999989

LPPLEGLHI

>C.ZA.05.CAP257_7w_F1.GQ999990

LPPLERLHI

>C.ZA.05.CAP63_5w_F4.GQ999976

LPPIEKLHI

>C.ZA.05.CAP65_6w.GQ999977

LPPIERLTI

>C.ZA.05.CAP84_3w_F2.GQ999978

LPPIERLHI

>C.ZA.05.CAP85_5w_F1.GQ999979

LPPIERLHI

>C.ZA.05.CAP88_5w_F2.GQ999980

LPPIERLHI

>C.ZA.06.704010017_B7.KC894111

LPPIERLHI

>C.ZA.06.CAP177_1A3.KC894133

LPPIERLHI

>C.ZA.06.CAP221_B14.KC894137

LPPIERLHI

>C.ZA.07.34_D12.KC894386

LPPLERLHI

>C.ZA.07.503_01009_4.KT183064

LPPIERLSI

>C.ZA.07.503_02051_66.KT183087

LPPIERLHI

>C.ZA.07.503_02182_B5.KT183090

LPPIERLTI

>C.ZA.07.503_02660_1.KT183094

LPPIEKLHI

>C.ZA.07.503_02854_C1.KT183103

LPPIERLHI

>C.ZA.07.503_05649_G6.KT183148

LPPIERLNI

>C.ZA.07.503_07352_C1.KT183196

LPPIERLHI

>C.ZA.07.503_07518_E9.KT183211

LPPIERLHI

>C.ZA.07.503_07711_D6.KT183216

LPPIERLHI

>C.ZA.07.503_09003_5.KT183229

LPPIERLHI

>C.ZA.07.503_10430_2.KT183253

LPPIEGLSI

>C.ZA.07.503_11204_39.KT183265

LPPIERLSI

>C.ZA.07.503_11441_B1.KT183271

LPPIERLHI

>C.ZA.07.503_13503_A5.KT183279

LPPIERLHI

>C.ZA.07.704010042_CH042_TF.KC156123

LPPIERLHI

>C.ZA.07.704010069_C6.KC894114

LPPIEKLHI

>C.ZA.07.704010083_B8.KC894116

LPSIEKLHI

>C.ZA.07.704010124_E6.KC894117

LPPIERLHI

>C.ZA.07.704809221_1B3.KC894118

LPPIEKLHI

>C.ZA.07.704810053_2B7.KC894119

LPPIERLHI

>C.ZA.07.705010067_CH067_TF.KC156125

LPPIERLHI

>C.ZA.07.705010162_CH162.mo6.KC156115

LPPIERLHI

>C.ZA.07.705010198_CH198_TF.KC156130

LPPIERLHI

>C.ZA.07.706010164_CH164_TF.KC156127

LPPIERLSI

>C.ZA.07.BP00005_RH03.JN687704

LPPIERLHI

>C.ZA.07.BP00008_RH01.JN687706

LPPIERLHI

>C.ZA.07.BP00009_SUP_RH01.JN687717

LPPIEKLHI

>C.ZA.07.BP00010_RH01.JN687718

LPPIERLHI

>C.ZA.07.BP00035_RH01.JN687736

LPPIERLSI

>C.ZA.07.DEMC07ZA011.JX140664

LPPIERLHI

>C.ZA.08.503_00146_D3.KT183053

LPPIERLHI

>C.ZA.08.503_00885_10.KT183056

LPPLERLHI

>C.ZA.08.503_01918_H10.KT183083

LPPIERLHI

>C.ZA.08.503_03257_D1b.KT183125

LPPIEGLHL

>C.ZA.08.503_03576_12.KT183128

LPPIERLCI

>C.ZA.08.503_04394_C2.KT183135

LPPIERLSI

>C.ZA.08.503_05780_4.KT183153

LPPIERLHI

>C.ZA.08.503_06150_1.KT183155

LPPIERLHI

>C.ZA.08.503_06310_27.KT183168

LPPIERLHI

>C.ZA.08.503_06485_10_2.KT183172

LPPIERLTI

>C.ZA.08.503_06877_E1.KT183183

LPPIERLCI

>C.ZA.08.503_07001_A1.KT183188

LPPIERLHI

>C.ZA.08.503_07464_A5.KT183201

LPPIERLHI

>C.ZA.08.503_08252_19.KT183218

LPPIEGLHI

>C.ZA.08.503_09289_T4.KT183243

LPPLERLHI

>C.ZA.08.503_10071_D2B.KT183245

LPPIERLHI

>C.ZA.08.503_10230_16.KT183250

LPPIERLSI

>C.ZA.08.503_11070_A10.KT183258

LPPIERLHI

>C.ZA.08.503_11474_32.KT183274

LPPIERLHI

>C.ZA.08.503_15383_RH2.KT183332

LPPIEKLHI

>C.ZA.08.503_15405_30.KT183336

LPPLERLSI

>C.ZA.08.705010185_CH185.mo6.KC156116

LPPIERLHI

>C.ZA.08.705010534_CH534.w12.KC156221

LPPIERLHI

>C.ZA.08.BP00014_RH01.JN687725

LPPIERLHI

>C.ZA.08.BP00016_RH01.JN687726

LPPIERLHI

>C.ZA.08.BP00018_RH01.JN687728

LPPIERLHI

>C.ZA.08.BP00019_RH01.JN687729

LPPIERLHI

>C.ZA.08.BP00023_RH02.JN687730

LPPIERLCI

>C.ZA.08.BP00025_RH03.JN687732

LPPIERLHI

>C.ZA.08.CH0078.w60.e12.KC149008

LPPIERLHI

>C.ZA.08.DEMC08ZA011.JX140666

LPPIEKLHI

>C.ZA.09.503_01244_10.KT183068

LPPLERLHI

>C.ZA.09.503_02869_RH10.KT183114

LPPIEKLHI

>C.ZA.09.503_05130_2.KT183141

LPPIERLHI

>C.ZA.09.503_13580_04.KT183289

LPPIERLTI

>C.ZA.09.DEMC09ZA008.JX140667

LPPLERLHI

>C.ZA.09.DEMC09ZA009.JX140668

LPPIERLHI

>C.ZA.10.DEMC10ZA001.JX140669

LPPLERLHI

>C.ZA.11.CH0152_3_d1360_ipe026_30_34.MG901120

LPPIERLNI

>C.ZA.12.DEMC12ZA087.KP109516

LPPIERLHI

>C.ZA.12.DEMC12ZA096.KP109517

LPPIERLHI

>C.ZA.13.DEMC13ZA146.KU749415

LPPLERLHI

>C.ZA.13.DEMC13ZA149.KU749416

LPPIERLHI

>C.ZA.13.DEMC13ZA152.KU749417

LPPIERLHI

>C.ZA.13.HIV1_FR004_D11_CVL_1.MH933705

LPPIENLHI

>C.ZA.13.HIV1_FR006_D1_CVL_1.MH933704

LPPIERLHI

>C.ZA.14.HIV1_FR014_D7_plasma_1.MH933706

LPPLERLHI

>C.ZA.89.pZAC_R3714.JN188292

LPPIERLHI

>C.ZA.97.97ZA003.AY118165

LPPIERLHI

>C.ZA.97.97ZA012.AF286227

LPPIERLHI

>C.ZA.98.98ZA445.AY158533

LPPIERLHI

>C.ZA.98.98ZA502.AY158534

LPPIERLNL

>C.ZA.98.98ZA528.AY158535

LPPIERLHI

>C.ZA.98.CTSC2.AY043176

LPPIERLHI

>C.ZA.98.TV012.AY162225

LPPIERLHI

>C.ZA.99.99ZACM9.AF411967

LPPIERLHI

>C.ZA.99.99ZALT21.EU293446

LPPLERLHI

>C.ZA.99.99ZALT42.EU293448

LPPLERLHI

>C.ZA.99.99ZALT45.EU293449

LPPIERLHI

>C.ZA.99.99ZALT46.EU293450

LPPIERLNI

>C.ZA.99.99ZALT4.EU293444

LPPIERLHI

>C.ZA.99.99ZALT5.EU293445

LPPIERLHI

>C.ZA.99.99ZATM10.AY228556

LPPIERLHI

>C.ZA.99.DU179.AY043174

LPPIERLHI

>C.ZA.99.DU422.AY043175

LPPIERLHI

>C.ZA.99.ZA8119636.KU168308

LPPIERLHI

>C.ZA.99.ZASW7.AF411966

LPPIEGLHI

>C.ZM.02.02ZM110.AB254142

LPPIERLNI

>C.ZM.02.02ZM114.AB254146

LPPIERLHI

>C.ZM.02.02ZMBC.AB254149

LPPIERLHI

>C.ZM.02.02ZMDB.AB254153

LPPIERLHI

>C.ZM.02.02ZMJM.AB254156

LPPLERLHI

>C.ZM.03.ZM246F_flA10.FJ496186

LPPIERLTI

>C.ZM.03.ZM247F_flA1.FJ496195

LPPIERLHI

>C.ZM.03.ZM249M_flC1.FJ496209

LPPLERLHL

>C.ZM.05.ZM373_200373_4.GU329051

LPPIEGLHI

>C.ZM.07.DEMC07ZM013.KU749425

LPPIERLHI

>C.ZM.09.DEMC09ZM004.KF716466

LPPIERLNI

>C.ZM.09.DEMC09ZM015.KU749426

LPPIEGLHI

>C.ZM.09.Z331F_15Apr09_PL_NFLG_SGA22.KR820304

LPPIERLHI

>C.ZM.09.Z331M_18Apr09_PL_NFLG_SGA10.KR820314

LPPIEKLHI

>C.ZM.09.Z3576F_28Mar09_PL_NFLG_SGA1.KR820324

LPPIERLCI

>C.ZM.09.Z3576M_18Apr09_PL_NFLG_SGA10.KR820326

LPPIERLCI

>C.ZM.09.Z3618F_11Jul09_PL_NFLG_SGA11.KR820342

LPPIERLHI

>C.ZM.09.Z3678F_23Sep09_PL_NFLG_SGA19.KR820376

LPPIEGLHI

>C.ZM.10.Z4248F_11Jun10_PL_NFLG_SGA1.KR820394

LPPIERLHI

>C.ZM.10.Z4248M_4Jun10_PL_NFLG_SGA1.KR820415

LPPIERLHI

>C.ZM.11.DEMC11ZM003.KP109494

LPPIERLHI

>C.ZM.11.DEMC11ZM005.KP109495

LPPIERLRI

>C.ZM.11.DEMC11ZM006.KF716467

LPPLERLHI

>C.ZM.11.DEMC11ZM008.KP109496

LPPIERLNI

>C.ZM.89.ZAM18.AB485645

LPPIERLHI

>C.ZM.96.96ZM651.AF286224

LPPIERLHI

>C.ZM.x.HIV1084i.AY805330

LPPIERLHI

>C.x.03.LA07SeJe.KU168262

LPPIERLTI

>C.x.x.pCe3045_2D5.KC894102

LPPIERLHI

>D.BR.10.10BR_RJ095.KJ787684

LPPLERLTL

>D.BR.10.10BR_RJ108.KJ787683

LPPLERLTL

>D.BR.96.patient_96BRRJ100.DQ141204

LPPLERLTL

>D.CD.02.CG_0382_02V_NGSID3.KY392769

LPPIERLTL

>D.CD.02.LA18ZiAn.KU168272

LPPLERLTL

>D.CD.03.LA17MuBo.KU168271

LPPLERLSL

>D.CD.84.84ZR085.U88822

LPPLERINL

>D.CD.87.PBS5635.MH705152

LPPIERLTL

>D.CM.01.01CM_0009BBY.AY371155

LPPLERLNL

>D.CM.01.01CM_0175BA.AY371156

LPPLERLNL

>D.CM.10.DEMD10CM009.JX140670

LPPIERLNL

>D.CY.06.CY163.FJ388945

LPPLERLNL

>D.FI.93.FIN93167.AF219271

LPPLERLNL

>D.FI.93.FIN93178.AF219272

LPPLERLTL

>D.GB.13.15228_1_51.3.MF109689

LPPLERLTL

>D.GB.13.15228_1_72.3.MF109710

LPPLERLTL

>D.GB.13.15228_1_75.4.MF109713

LPPLDRLTL

>D.GB.14.14535_1_3.3.MF109532

LPPLERLTL

>D.KE.01.NKU3006.AF457090

LPPLERLNL

>D.KE.11.DEMD11KE003.KF716476

LPPLERLTL

>D.KE.93.QA465_59M_ENV_A1.FJ866136

LPPLERLNL

>D.KE.95.QA013_70I_ENV_M12.FJ866135

LPPLERLNL

>D.KE.97.ML415_2.AY322189

LPPLERLSL

>D.KE.97.QB857_110I_ENV_B3.FJ866138

LPPIEKLTI

>D.KE.99.QD435_100M_ENV_A4.FJ866139

LPPLERLNL

>D.SE.12.077UG.MF373180

LPPLERLNL

>D.SN.90.SE365A2.L22945

LPPVERLNL

>D.TD.99.MN011.AJ488926

LPPIERLNL

>D.TD.99.MN012.AJ488927

LPPIERLNL

>D.TZ.01.A280.AY253311

LPPLERLNL

>D.TZ.04.CO6405V4.KX907406

LPPIERLNL

>D.UG.05.p190049.JX236668

LPPLERLSL

>D.UG.07.p191647.JX236670

LPPLERLNL

>D.UG.07.p191882.JX236673

LPPLERLTL

>D.UG.07.pSC191727.JX236679

LPPLEKLNL

>D.UG.08.p191859.JX236672

LPPLERLNL

>D.UG.10.DEMD10UG004.KF716479

LPPLERLNI

>D.UG.11.DEMD11UG003.KF716480

LPPLERLTI

>D.UG.90.UG269A.L22949

LPPLERLNL

>D.UG.90.UG274A2.L22950

LPPLERLTL

>D.UG.94.94UG114.U88824

LPPLERLNL

>D.UG.95.42_877.MH705143

LPPLERLNL

>D.UG.98.98UG57128.AF484502

LPPLDRLNL

>D.UG.98.98UG57130.AF484504

LPPLERLNL

>D.UG.98.98UG57131.AF484505

LPPIERLNL

>D.UG.98.98UG57132.AF484506

LPPLEKLSL

>D.UG.98.98UG57140.AF484511

LPPIERLNL

>D.UG.98.98UG57143.AF484514

LPPLERLNL

>D.UG.99.99UGA03349.AF484518

LPPIERLNL

>D.UG.99.99UGA07412.AF484477

LPPLERLNL

>D.UG.99.99UGA08483.AY304496

LPPLERLTL

>D.UG.99.99UGB21875.AF484480

LPPLERLNL

>D.UG.99.99UGB25647.AF484481

LPPLERLTL

>D.UG.99.99UGB32394.AF484483

LPPLERLNL

>D.UG.99.99UGD23550.AF484485

LPPLERLNL

>D.UG.99.99UGD26830.AF484486

LPPLERLTL

>D.UG.99.99UGE13613.AF484515

LPPLERLSL

>D.UG.99.99UGE23438.AF484489

LPPLERLTL

>D.UG.99.99UGF03726.AF484519

LPPLERLTL

>D.UG.99.99UGF05734.AF484490

LPPLERLSL

>D.UG.99.99UGG10555.AF484494

LPPLERLNL

>D.UG.99.99UGG35093.AF484495

LPPLERLSL

>D.UG.99.99UGK09259.AF484498

LPPLERLTL

>D.UG.99.99UGK09958.AF484499

LPPIEKLNI

>D.US.07.HIV_US_BID_V3121_2007.JQ403079

LPPLERLTI

>D.YE.01.01YE386.AY795903

LPPLERLNL

>D.YE.02.02YE516.AY795907

LPPIERLNL

>D.ZA.84.R2.AY773338

LPPLQRLTL

>D.ZA.85.R214.AY773339

LPPLERLTL

>D.ZA.85.R286.AY773340

LPPLERLTL

>F1.AO.06.AO_06_ANG32.FJ900266

LPPLERLHI

>F1.AO.06.AO_06_ANG40.FJ900267

LPPLERLHI

>F1.AO.06.AO_06_ANG58.FJ900268

LPPLERLHI

>F1.AR.02.ARE933.DQ189088

LPPLERLHI

>F1.BE.93.VI850.AF077336

LPPLERLHI

>F1.BR.01.01BRRJSB153.MG365763

LPPLERLHI

>F1.BR.02.02BR082.FJ771006

LPPLERLHI

>F1.BR.06.06BR564.FJ771008

LPPLERLHI

>F1.BR.06.06BR579.FJ771009

LPPLERLNI

>F1.BR.07.07BR844.FJ771010

LPPLERLHI

>F1.BR.08.08BRRJ35.MG365767

LPPLERLHI

>F1.BR.10.10BR_PE107.KJ849782

LPPLERLHI

>F1.BR.10.10BR_RJ015.KJ849791

LPPLERLHI

>F1.BR.10.10BR_RJ055.KT427774

LPPLERLHI

>F1.BR.10.10BR_RJ084_2.KT427868

LPPLENLHI

>F1.BR.10.DEMF110BR015.KU749395

LPPIERLNL

>F1.BR.11.11BRRJPR69.MG365766

LPPLERLHI

>F1.BR.11.11BRRJPR90.MG365768

LPPLERLNI

>F1.BR.11.DEMF111BR037.KU749396

LPPLERLHI

>F1.BR.12.12BRRJP05.MG365762

LPPLERLHI

>F1.BR.12.12BRRJPR51.MG365764

LPPLERLHI

>F1.BR.89.BZ126.AY173957

LPPLERLNI

>F1.BR.89.BZ163.L22085

LPPPERLTI

>F1.BR.93.93BR020_1.AF005494

LPPLERLHI

>F1.CY.08.CY222.JF683771

LPSLERLHI

>F1.DE.x.MVP_30846.EU446022

LPPLERLHI

>F1.ES.11.DEMF110ES001.JX140671

LPPLERLNL

>F1.ES.11.P2944.KY639271

LPPLERLNL

>F1.ES.11.VA0053_nfl.KJ883138

LPPLERLNI

>F1.ES.14.100347.MF381271

LPPLERLHI

>F1.ES.15.100350.MF381286

LPPLERLHI

>F1.ES.16.100349.MF381284

LPPLERLHI

>F1.ES.x.P1146.DQ979023

LPPLERLHI

>F1.ES.x.X1093_2.DQ979025

LPPLERLHI

>F1.ES.x.X1670.DQ979024

LPPIERLHI

>F1.FI.93.FIN9363.AF075703

LPPLERLHI

>F1.FR.04.LA22LeRe.KU168276

LPPLERLHI

>F1.FR.96.96FR_MP411.AJ249238

LPPLERLHI

>F1.GB.14.13659_1_45.3.MF109434

LPPLERLHI

>F1.GB.14.14535_1_29.3.MF109526

LPPLERLHI

>F1.RO.03.LA20DuCl.KU168274

LPPLERLHI

>F1.RO.96.BCI_R07.AB485658

LPPLERLHI

>F1.x.03.LA21LeAn.KU168275

LPPLERLHI

>F2.CM.01.A1699.MH705144

LPPLERLHI

>F2.CM.02.02CM_0016BBY.AY371158

LPPLERLHI

>F2.CM.10.DEMF210CM001.JX140672

LPPLERLHI

>F2.CM.10.DEMF210CM007.JX140673

LPPLERLHI

>F2.CM.11.DEMF211CM025.KU749420

LPPLERLHI

>F2.CM.11.DEURF11CM026.KU749422

LPPLERLHI

>F2.CM.95.95CM_MP255.AJ249236

LPPLERLTI

>F2.CM.95.95CM_MP257.AJ249237

LPLLEKLHI

>F2.CM.97.CM53657.AF377956

LPPLERLHI

>G.BE.96.DRCBL.AF084936

LPPLERLHL

>G.CD.03.LA23LiEd.KU168277

LPPLERLHI

>G.CD.87.87_2580.MH705162

LPPIERLHL

>G.CD.87.P406.MH705155

LPPLERLHI

>G.CD.87.PBS1191.MH705134

LPPLERLHL

>G.CM.01.01CM_4049HAN.AY371121

LPPIERLCL

>G.CM.01.A1786.FJ389367

LPPLERLRL

>G.CM.03.CM44_10.KU168302

LPPLERLSL

>G.CM.04.178_15.FJ389363

LPPLERLHL

>G.CM.04.314_40.FJ389364

LPPLERLHL

>G.CM.04.515_28.FJ389365

LPPLERLHI

>G.CM.04.944_5.FJ389366

LPPLEGLXL

>G.CM.05.144_26.MH705145

LPPLDRLHI

>G.CM.06.740_14.KP718915

LPPIERLHI

>G.CM.07.920_49.KP718923

LPPLERLHI

>G.CM.07.BS48.KR017776

LPPLERLHL

>G.CM.08.789_10.KP718925

LPPLERLCI

>G.CM.10.DEMG10CM008.JX140676

LPPLERLHI

>G.CM.10.DEURF10CM020.KP109502

LPPIERLNL

>G.CM.11.DEMG11CM046.KY658701

LPPIERLSL

>G.CM.96.96CMABB55.AY772535

LPPLERLHL

>G.CN.06.sh52.HM067749

LPPLERLCL

>G.CN.08.GX_2084_08.JN106043

LPPIERLHL

>G.CN.13.GZ8H3748.13.MH431770

LPPLERLHL

>G.CN.16.224GX.KY275364

LPPIDRLHL

>G.CU.99.Cu74.AY586547

LPPLERLSL

>G.CU.99.Cu85.AY586548

LPPLERLTL

>G.CU.99.Cu87.AY586549

LPPLERLTL

>G.ES.00.X558.AF423760

LPPLERLRL

>G.ES.05.P962.EU786670

LPPLERLHL

>G.ES.05.X1628_2.FJ670520

LPPLERLSL

>G.ES.08.P1981_2.FJ670530

LPPLERLHL

>G.ES.09.P2196_3s_nt0558_9495.MF157743

LPPLERLHL

>G.ES.09.X2634_2.GU362882

LPPLERLHI

>G.ES.14.ARP1201.KT276261

LPPLERLCL

>G.ES.14.EUR_0033.KU685592

LPPLERLHL

>G.ES.99.X138.AF450098

LPPLERLTL

>G.GB.13.15171_1_17.3.MF109647

LPPLERLHL

>G.GB.14.14592_1_74.4.MF109568

LPPLERLHL

>G.GB.14.14667_1_38.4.MF109598

LPPLERLTL

>G.GH.03.03GH175G.AB287004

LPPLERLCL

>G.GW.08.LA57LmNe.KU168300

LPPLERLHL

>G.KE.06.06KE275457V6.KT022379

LPPLERLHI

>G.KE.09.DEMG09KE001.KF716477

LPPLERLHI

>G.KE.93.HH8793_1_1.AF061640

LPPLERLHL

>G.NG.01.01NGPL0669.DQ168576

LPPLERLHL

>G.NG.01.01NGPL0674.DQ168575

LPPLERLHL

>G.NG.01.01NGPL0760.DQ168579

LPPLERLHL

>G.NG.01.PL0567.DQ168573

LPPLERLHL

>G.NG.08.08NG_SC13.JN248582

LPPIERLHL

>G.NG.09.09NG010079.KX389636

LPPLERLHL

>G.NG.09.09NG010105.KX389635

LPPLERLHI

>G.NG.09.09NG010157.KX389631

LPPLERLHI

>G.NG.09.09NG010205.KX389628

LPPLERLSL

>G.NG.09.09NG010261.KX389626

LPPIERLTL

>G.NG.09.09NG010315.KX389625

LPPLERLNL

>G.NG.09.09NG_SC21.JN248584

LPPLERLCL

>G.NG.09.09NG_SC26.JN248586

LPPIERLRL

>G.NG.09.09NG_SC31.JN248591

LPPIERLHI

>G.NG.09.09NG_SC62.JN248593

LPPLERLHL

>G.NG.10.10NG020133.KX389620

LPPLERLHL

>G.NG.10.10NG020303.KX389618

LPPIERLNL

>G.NG.10.10NG020420.KX389615

LPPIEXLNL

>G.NG.11.11NG050158.KX389641

LPPIERLRL

>G.NG.11.11NG050272.KX389642

LPPLERLHI

>G.NG.11.11NG050489.KX389645

LPPLERLHL

>G.NG.11.DEMG11NG006.KY953200

LPPLERLSL

>G.NG.11.DEMG11NG007.KY953201

LPPIERLRL

>G.NG.11.DEMG11NG008.KY953202

LPPLERLHL

>G.NG.12.12NG060248.KX389646

LPPLERLHL

>G.NG.12.12NG060409.KX389648

LPPIERLNL

>G.NG.92.92NG083_JV10832.U88826

LPPLEGLSL

>G.PT.x.PT2695.AY612637

LPPLERLHL

>G.PT.x.PT3037.FR846408

LPPLERLHL

>G.PT.x.PT3306.FR846409

LPPLERLHL

>G.PT.x.PT988.FR846410

LPPLERLRL

>G.RU.12.RU_SRD_2012.MF614606

LPPLERLHI

>G.RU.89.RU_ShRMT_1989.MF614605

LPPLERLHI

>G.SE.93.SE6165_G6165.AF061642

LPPLERLHL

>G.ZA.01.TV546.KJ948662

LPPLERLTI

>G.x.03.LA24HoCe.KU168278

LPPIDRLSL

>H.BE.93.VI991.AF190127

LPPLERLTL

>H.BE.93.VI997.AF190128

LPPLERLTL

>H.CD.01.CG_0260_02V_NGSID16.KY392779

LPPLERLSL

>H.CD.01.CG_0536_02_NGSID14.KY392777

LPPLERLTI

>H.CD.01.CG_0538_02_NGSID15.KY392778

LPPLERLTL

>H.CD.04.LA19KoSa.KU168273

LPPLERLTL

>H.CF.02.LA25LeMi.KU168279

LPPLERLTL

>H.CF.90.056.AF005496

LPPLERLTL

>H.GB.00.00GBAC4001.FJ711703

LPPLERLTL

>J.CD.02.CG_0331_02V_NGSID13.KY392776

LPPIERLRL

>J.CD.03.LA26DiAn.KU168280

LPPIERLRL

>J.CD.97.J_97DC_KTB147.EF614151

LPPIERLRL

>J.CM.04.04CMU11421.GU237072

LPPIERLHI

>J.SE.94.SE9173_7022.AF082395

LPPIERLRL

>K.CD.87.P3844.MH705156

LPPLERLNL

>K.CD.97.97ZR_EQTB11.AJ249235

LPPLERLSL

>K.CM.96.96CM_MP535.AJ249239

LPPIEKLNL

>01_AE.CF.90.90CF11697.AF197340

LPPLERLHI

>01_AE.CF.90.90CF4071.AF197341

LPPLERLHL

>01_AE.CF.90.90CR402_CAR_E_4002.U51188

LPPLERLHL

>01_AE.CM.11.1156_26.KP718930

LPPLERLTL

>01_AE.CN.02.YN0203.JX112860

LPPLERLSL

>01_AE.CN.02.YN0221.JX112861

LPPLERLNL

>01_AE.CN.02.YN0229.JX112863

LPPLERLNL

>01_AE.CN.02.YN0236.JX112866

LPPLERLHL

>01_AE.CN.05.05GX002.GU564222

LPPLEGLKL

>01_AE.CN.05.05GX013.GU564224

LPPLERLQL

>01_AE.CN.05.05GX014.GU564225

LPPLERLRL

>01_AE.CN.05.05GX034.GQ845124

LPPLERLHL

>01_AE.CN.05.05GX156.GU564228

LPPLERLHL

>01_AE.CN.05.05GX162.GU564229

LPPLERLQL

>01_AE.CN.05.FJ051.DQ859178

LPPLERLHL

>01_AE.CN.05.Fj052.EF036528

LPPLERLHL

>01_AE.CN.05.Fj055.EF036527

LPPLERLHL

>01_AE.CN.05.Fj056.EF036529

LPPIERLHL

>01_AE.CN.05.Fj057.EF036530

LPPLERLHL

>01_AE.CN.05.Fj065.EF036534

LPPLERLHL

>01_AE.CN.05.Fj066.EF036535

LPPLERLHL

>01_AE.CN.06.AE02.EU363850

LPPLERLHI

>01_AE.CN.06.FJ054.DQ859180

LPPLERLHI

>01_AE.CN.06.Fj064.EF036533

LPPLERLHL

>01_AE.CN.07.07CNYN312.KF835499

LPPLERLHI

>01_AE.CN.07.07CNYN315.KF835502

LPPLERLHL

>01_AE.CN.07.07CNYN316.KF835503

LPPLERLHL

>01_AE.CN.07.07CNYN317.KF835504

LPPLERLHL

>01_AE.CN.07.07CNYN318.KF835505

LPPLERLSL

>01_AE.CN.07.07CNYN326.KF835513

LPPLERLNL

>01_AE.CN.07.07CNYN327.KF835514

LPPLERLHL

>01_AE.CN.07.07CNYN329.KF835516

LPPLERLHL

>01_AE.CN.07.07CNYN332.KF835518

LPPLERLQL

>01_AE.CN.07.07CNYN333.KF835519

LPPLERLHL

>01_AE.CN.07.07CNYN337.KF835523

LPPLERLNL

>01_AE.CN.07.07CNYN342.KF835527

LPPLERLQL

>01_AE.CN.07.07CNYN343.KF835528

LPPLERLHL

>01_AE.CN.07.07CNYN354.KF835533

LPPLEGLHL

>01_AE.CN.07.07CNYN357.KF835536

LPPLERLNL

>01_AE.CN.07.07CNYN359.KF835538

LPPLERLHL

>01_AE.CN.07.07CNYN364.KF835542

LPPLERLHL

>01_AE.CN.07.07CNYN366.KF835543

LPPLERLHI

>01_AE.CN.07.07JSWX045.FJ441290

LPPLERLHL

>01_AE.CN.07.BJOX028000.e04.KM218078

LPPLERLQL

>01_AE.CN.07.FJ070010.JX112809

LPPLERLQL

>01_AE.CN.07.FJ070013.JX112810

LPPLERLHI

>01_AE.CN.07.FJ070017.JX112811

LPPLERLQL

>01_AE.CN.07.FJ070033.JX112813

LPPLERLHL

>01_AE.CN.07.FJ070035.JX112814

LPPLERLQL

>01_AE.CN.07.FJ070040.JX112817

LPPLERLQL

>01_AE.CN.07.FJ070043.JX112818

LPPLERLRL

>01_AE.CN.07.GD070010.JX112819

LPPLERLQL

>01_AE.CN.07.GD070058.JX112820

LPPLERLTL

>01_AE.CN.07.GD070059.JX112821

LPPLERLHL

>01_AE.CN.07.GD070090.JX112823

LPPLERLQL

>01_AE.CN.07.GD070092.JX112824

LPPLERLQL

>01_AE.CN.07.GD070096.JX112825

LPPLERLQL

>01_AE.CN.07.GD070118.JX112826

LPPLERLQL

>01_AE.CN.07.GD070120.JX112827

LPPLERLHL

>01_AE.CN.07.GX070003.JX112829

LPPLERLQL

>01_AE.CN.07.GX070005.JX112830

LPPLERLHI

>01_AE.CN.07.GX070006.JX112831

LPPLERLQL

>01_AE.CN.07.GX070043.JX112832

LPPLERLQL

>01_AE.CN.07.GX070044.JX112833

LPPLERLHI

>01_AE.CN.07.GX070076.JX112834

LPPLERLQL

>01_AE.CN.07.GX070143.JX112835

LPPLERLQL

>01_AE.CN.07.GX070145.JX112836

LPPLERLQL

>01_AE.CN.07.GX070149.JX112837

LPPLERLQL

>01_AE.CN.07.GX070154.JX112838

LPPLERLQL

>01_AE.CN.07.GX070167.JX112839

LPPLERLQL

>01_AE.CN.07.GZ070004.JX112840

LPPLERLQL

>01_AE.CN.07.GZ070123.JX112843

LPPLERLTL

>01_AE.CN.07.GZ070126.JX112844

LPPLERLHL

>01_AE.CN.07.GZ070127.JX112845

LPPLERLQL

>01_AE.CN.07.JS070901.JX112850

LPPLEKLHI

>01_AE.CN.07.JS071001.JX112851

LPPLERLQL

>01_AE.CN.07.JS071101.JX112853

LPPLERLHL

>01_AE.CN.07.LN070008.JX112854

LPPLERLQL

>01_AE.CN.07.LN070013.JX112856

LPPLERLHL

>01_AE.CN.08.08LNA002.JX960612

LPPLERLQL

>01_AE.CN.08.08LNA003.JX960606

LPPLERLHI

>01_AE.CN.08.08LNA004.JX960617

LPPLERLRL

>01_AE.CN.09.09LNA005.JX960630

LPPLERLHL

>01_AE.CN.09.09LNA007.JX960627

LPPLERLHL

>01_AE.CN.09.09LNA013.JX960623

LPPLERLQL

>01_AE.CN.09.09LNA025.JX960634

LPPLERLQL

>01_AE.CN.09.09LNA040.JX960615

LPPLERLQL

>01_AE.CN.09.09LNA086.JX960639

LPPLERLHI

>01_AE.CN.09.09LNA136.JX960631

LPPLERLHL

>01_AE.CN.09.09LNA340.JX960607

LPPLERLHL

>01_AE.CN.09.09LNA353.JX960628

LPPLERLQL

>01_AE.CN.09.09LNA379.JX960603

LPPLERLHI

>01_AE.CN.09.09LNA480.JX960636

LPPLERLQL

>01_AE.CN.09.10LNA016.JX960629

LPPLERLQL

>01_AE.CN.09.10LNA105.JX960626

LPPLERLHL

>01_AE.CN.09.1109.HQ215555

LPPLERLNL

>01_AE.CN.09.1119.HQ215553

LPPLERLQL

>01_AE.CN.09.YN09P0011.JX112867

LPPLERLQL

>01_AE.CN.09.YN09P0015.JX112868

LPPLERLHI

>01_AE.CN.09.ZK056.JX112870

LPPLERLHL

>01_AE.CN.10.10LNA057.JX960633

LPPLERLHL

>01_AE.CN.10.10LNA103.JX960608

LPPLERLHI

>01_AE.CN.10.10LNA124.JX960609

LPPLERLTL

>01_AE.CN.10.10LNA471.JX960637

LPPLERLQL

>01_AE.CN.10.10LNA571.JX960611

LPPLERLHL

>01_AE.CN.10.10LNA821.JX960616

LPPLERLQL

>01_AE.CN.10.10LNA976.JX960632

LPPLERLQL

>01_AE.CN.10.CYM059.JX112796

LPPLERLHL

>01_AE.CN.10.CYM075.JX112797

LPPLERLTL

>01_AE.CN.10.CYM105.JX112798

LPPLERLHL

>01_AE.CN.10.CYM124.JX112799

LPPLERLHL

>01_AE.CN.10.CYM136.JX112800

LPPLERLHL

>01_AE.CN.10.CYM138.JX112801

LPPLERLHL

>01_AE.CN.10.CYM139.JX112802

LPPLERLNL

>01_AE.CN.10.CYM140.JX112803

LPPLERLHL

>01_AE.CN.10.CYM143.JX112804

LPPLERLHL

>01_AE.CN.10.CYM147.JX112805

LPPLERLNL

>01_AE.CN.10.CYM152.JX112807

LPPLERLNL

>01_AE.CN.10.CYM154.JX112808

LPPLERLHL

>01_AE.CN.10.DE00110CN007.KP109506

LPPLERLHL

>01_AE.CN.10.DE00110CN009.KP109507

LPPLERLQL

>01_AE.CN.10.JL100005.JX112846

LPPLERLHL

>01_AE.CN.10.JL100020.JX112849

LPPLERLQL

>01_AE.CN.10.YNFL03.KC870029

LPPLERLHL

>01_AE.CN.10.YNFL20.KC870039

LPPLERLHL

>01_AE.CN.10.YNFL23.KC870041

LPPLERLHL

>01_AE.CN.11.DE00111CN003.KC596065

LPPLERLTL

>01_AE.CN.12.DE00112CN011.KP109508

LPPLERLHL

>01_AE.GB.10.Donor_N094_20_Month.KP873161

LPPLERLHL

>01_AE.HK.04.HK001.DQ234790

LPSLERLHL

>01_AE.IR.10.10IR.THR48F.AB703616

LPPIERLSL

>01_AE.JP.11.DE00111JP003.KF859741

LPPLERLHL

>01_AE.JP.x.DR0492.AB253423

LPPLERLHL

>01_AE.JP.x.DR2594.AB253668

LPPLERLHL

>01_AE.JP.x.JRC77AE.AB565504

LPPLERLTL

>01_AE.MM.14.fKSDU26.KU820849

LPPLERLHL

>01_AE.MM.99.mCSW105.AB097872

LPPIERLHL

>01_AE.PH.15.1008.MH327750

LPPLERLHL

>01_AE.PH.15.DE00115PH006.MH078560

LPPLERLSL

>01_AE.PH.15.DE00115PH012.KY658688

LPPIERLHL

>01_AE.PH.16.1010.MH327752

LPPLERLHL

>01_AE.PH.16.1024.MH327759

LPPIERLNL

>01_AE.PH.16.1025.MH327760

LPPLERLHL

>01_AE.PH.16.1027.MH327762

LPPLERLNL

>01_AE.PH.16.1029.MH327764

LPPLERLHL

>01_AE.PH.16.1031.MH327766

LPPLERLHL

>01_AE.PH.16.DE00116PH013.MH078562

LPPIERLHL

>01_AE.PH.17.1021.MH327756

LPPLERLHL

>01_AE.PH.17.1026.MH327761

LPPLERLTL

>01_AE.PH.17.1028.MH327763

LPPLERLHL

>01_AE.SE.09.034SE.MF373147

LPPLERLHL

>01_AE.SE.11.057SE.MF373160

LPPIERLRL

>01_AE.SE.11.SE601017.KP411840

LHRLERLNL

>01_AE.SE.11.SE601018.KP411841

LPPLERLHH

>01_AE.SE.11.SE601021.KP411842

LPPLERLNL

>01_AE.SE.12.069SE.MF373172

LPPLERLHL

>01_AE.SE.12.070SE.MF373173

LPPLERLSL

>01_AE.TH.00.00TH_C2101.AY945716

LPPLERLNL

>01_AE.TH.00.00TH_C2257.AY945717

LPPLERLHL

>01_AE.TH.00.00TH_C3347.AY945721

LPPLDRLHL

>01_AE.TH.00.00TH_C4118.AY945722

LPSLERLHI

>01_AE.TH.00.C1705.DQ789392

LPPLERLNL

>01_AE.TH.00.OUR200I.AY358066

LPPLDRLTL

>01_AE.TH.00.OUR721I.AY358067

LPPIERLHL

>01_AE.TH.00.OUR724I.AY358060

LPPLERLNL

>01_AE.TH.00.OUR746I.AY358061

LPPLERLHL

>01_AE.TH.00.OUR810I.AY358063

LPPLERLTL

>01_AE.TH.01.01TH_C1436.AY945713

LPPLERLNL

>01_AE.TH.01.01TH_C2570.AY945719

LPPLERLHL

>01_AE.TH.01.LA09DuCe.KU168264

LPPLERLHL

>01_AE.TH.01.OUR414I.AY358050

LPPIERLHL

>01_AE.TH.01.OUR609I.AY358040

LPPLERLNL

>01_AE.TH.01.OUR642I.AY358041

LPPLERLHL

>01_AE.TH.01.OUR647I.AY358056

LPPLERLHL

>01_AE.TH.01.OUR674I.AY358038

LPPLEGLSL

>01_AE.TH.01.OUR702I.AY358059

LPPLDRLHL

>01_AE.TH.01.OUR788I.AY358068

LPPLERLSL

>01_AE.TH.02.OUR769I.AY358062

LPPLERLHL

>01_AE.TH.03.TH7229.KU168309

LPPLERLSL

>01_AE.TH.04.04TH107542.JN248318

LPPLERLSL

>01_AE.TH.04.04TH328531.JN248324

LPPLERLTL

>01_AE.TH.04.04TH427990.JN248327

LPPLERLHL

>01_AE.TH.04.04TH505841.JN248328

LPPLERLHL

>01_AE.TH.04.04TH613543.JN248330

LPPLERLTL

>01_AE.TH.04.04TH807015.JN248334

LPPLERLTL

>01_AE.TH.04.04TH817196.JN248336

LPPLEGLRL

>01_AE.TH.04.AA027a_wg4.JX447021

LPPLERLHI

>01_AE.TH.04.AA075a_WG7.JX447542

LPPLERLHL

>01_AE.TH.04.BKM.DQ314732

LPPLERLHL

>01_AE.TH.05.05TH130087.JN248339

LPPLERLHL

>01_AE.TH.05.05TH327568.JN248341

LPPLERLTL

>01_AE.TH.05.05TH342968.JN248342

LPPLERLHL

>01_AE.TH.05.05TH741452.JN248355

LPPLERLHL

>01_AE.TH.05.05TH841749.JN248356

LPPLERLHL

>01_AE.TH.05.AA004a_wg4a.JX446712

LPPLERLHL

>01_AE.TH.05.AA023a13R.JX446977

LPPLERLTL

>01_AE.TH.05.AA029b08R.JX447048

LPPLERLHL

>01_AE.TH.05.AA033a_wg6a.JX447081

LPPLERLHL

>01_AE.TH.05.AA049a_WG13.JX447268

LPPLERLHL

>01_AE.TH.05.AA062b07R.JX447395

LPPLERLHL

>01_AE.TH.05.AA064a_WG2.JX447413

LPPLERLHL

>01_AE.TH.05.AA066a09R.JX447447

LPPLERLHL

>01_AE.TH.05.AA077a_RH10.JX447561

LSPLERLHL

>01_AE.TH.05.AA078a11R.JX447579

LPPLERLHL

>01_AE.TH.05.AA079a_WG4.JX447592

LPPLERLNL

>01_AE.TH.05.AA081a14.JX447619

LPPLERLHI

>01_AE.TH.05.AA097a09R.JX447855

LPPLERLHL

>01_AE.TH.05.AA101a_WG1.JX447936

LPPLERLQL

>01_AE.TH.05.AA103a06R.JX447958

LPPLERLHL

>01_AE.TH.05.AA107a_wg4.JX448022

LPPLERLTL

>01_AE.TH.05.AA122a02R.JX448217

LPPLERLRL

>01_AE.TH.05.AA125a11R.JX448256

LPSLEGLKL

>01_AE.TH.05.AA126a07R.JX448271

LPPLERLRL

>01_AE.TH.06.AA002a_WG1.JX446666

LPPLERLQL

>01_AE.TH.06.AA003b12R.JX446699

LPPLERLQL

>01_AE.TH.06.AA006a02.JX446736

LPPLERLHL

>01_AE.TH.06.AA014a01.JX446855

LPPLERLHL

>01_AE.TH.06.AA017a_wg1.JX446899

LPPLERLHL

>01_AE.TH.06.AA022a_RH2.JX446961

LPPIERLHI

>01_AE.TH.06.AA034a_wg2.JX447089

LPPLERLHL

>01_AE.TH.06.AA038a_WG3.JX447132

LPPLERLHL

>01_AE.TH.06.AA042a10R.JX447203

LPPLERLHL

>01_AE.TH.06.AA044a_RH2.JX447222

LPPLERLHL

>01_AE.TH.06.AA055a_WG4.JX447312

LPPLERLHL

>01_AE.TH.06.AA056a_WG5.JX447316

LPPLERLNL

>01_AE.TH.06.AA058a04R_434239.JX447346

LPPLERLQL

>01_AE.TH.06.AA059a_WG5.JX447356

LPPLERLHL

>01_AE.TH.06.AA063a_WG37.JX447409

LPPLERLHI

>01_AE.TH.06.AA068a_14.JX447465

LPPLERLHL

>01_AE.TH.06.AA073a_RH1.JX447515

LPPLDRLNL

>01_AE.TH.06.AA082a_WG9.JX447646

LPPLERLHL

>01_AE.TH.06.AA085a_wg2.JX447682

LPPIERLHL

>01_AE.TH.06.AA086a06R.JX447699

LPPLERLHL

>01_AE.TH.06.AA088a_wg14.JX447712

LPPLERLHL

>01_AE.TH.06.AA099a_WG9.JX447891

LPPLERLHL

>01_AE.TH.06.AA100b15R.JX447903

LPPLERLHL

>01_AE.TH.06.AA104a_RH3.JX447984

LPPLERLHL

>01_AE.TH.06.AA109a01R.JX448039

IPPLERLHL

>01_AE.TH.06.AA116a_01R.JX448118

LPPLERLHL

>01_AE.TH.06.AA121a04R.JX448198

LPPLERLHL

>01_AE.TH.06.AA123a04R.JX448238

LPPLERLNL

>01_AE.TH.06.AA127a02R.JX448279

LPPLERLHL

>01_AE.TH.06.AA129a03R.JX448292

LPPLERLHI

>01_AE.TH.07.AA005a07R.JX446728

LPPLERLSL

>01_AE.TH.07.AA008a_RH10.JX446776

LPPLERLHL

>01_AE.TH.07.AA009b01R.JX446790

LPPLERLQL

>01_AE.TH.07.AA012a_RH12.JX446845

LPPLERLRI

>01_AE.TH.07.AA015a_WG4.JX446877

LPPLERLHL

>01_AE.TH.07.AA019a_WG11.JX446918

LPPLERLHL

>01_AE.TH.07.AA026a02R.JX447013

LPPLERLHL

>01_AE.TH.07.AA028a_wg3.JX447028

LPPLERLHL

>01_AE.TH.07.AA030a04.JX447057

LPPLERLHL

>01_AE.TH.07.AA031a02.JX447064

LPPLERLSL

>01_AE.TH.07.AA036a05R.JX447118

LPPLERLHL

>01_AE.TH.07.AA050a_WG7.JX447283

LPPLERLHL

>01_AE.TH.07.AA061a02R.JX447381

LPPLERLHL

>01_AE.TH.07.AA065a15R.JX447437

LPPLERLHL

>01_AE.TH.07.AA069a01R.JX447480

LPPIERLTL

>01_AE.TH.07.AA072a03.JX447503

LPPLERLHI

>01_AE.TH.07.AA080a09R.JX447608

LPPLERLHL

>01_AE.TH.07.AA089a05.JX447721

LPPLERLHL

>01_AE.TH.07.AA091a10R.JX447752

LPPLERLSL

>01_AE.TH.07.AA098a04R.JX447876

LPPLERLNL

>01_AE.TH.07.AA105a10R.JX448005

LPPLERLHL

>01_AE.TH.07.MERLBDTRC2.JN860761

LPPIERLHL

>01_AE.TH.07.MERLBDTRC4.JN860763

LPPLERLHL

>01_AE.TH.07.MERLBDTRC5.JN860764

LPPIERLHL

>01_AE.TH.07.MERLBDTRC7.JN860766

LPPIERLHL

>01_AE.TH.07.MERLBDTRC8.JN860767

LPXIEXLTL

>01_AE.TH.08.AA001a07R.JX446655

LPPLERLHL

>01_AE.TH.08.AA007a_WG10.JX446756

LPPLERLHL

>01_AE.TH.08.AA013a04.JX446850

LPPLERLHL

>01_AE.TH.08.AA016a14R.JX446888

LPPLERLNL

>01_AE.TH.08.AA032a04.JX447074

LPPLERLQL

>01_AE.TH.08.AA037a_WG6.JX447127

LPPLERLHL

>01_AE.TH.08.AA052a08.JX447303

LPPLERLHL

>01_AE.TH.08.AA054a06.JX447308

LPPLERLTL

>01_AE.TH.08.AA060a_WG1.JX447359

LPPLERLHL

>01_AE.TH.08.AA067a_WG12.JX447458

LPPLERLHL

>01_AE.TH.08.AA070a_RH01.JX447493

LPPLERLHL

>01_AE.TH.08.AA102a_RH1.JX447941

LPPLERLHL

>01_AE.TH.08.AA108a_WG6.JX448028

LPPLERLHL

>01_AE.TH.08.AA110a01R.JX448051

LPPLERLHL

>01_AE.TH.08.AA112a02R.JX448072

LPPIERLHL

>01_AE.TH.08.AA113a_RH1.JX448086

LPPLERLRL

>01_AE.TH.08.AA131a01R.JX448312

LPPLERLHL

>01_AE.TH.08.MERLBDTRC9.JN860768

LPPLERLHL

>01_AE.TH.09.40061v05_02.KY580553

LPPLERLHL

>01_AE.TH.09.AA045a01R.JX447238

LPPLERLNL

>01_AE.TH.09.AA090a_WG11.JX447727

LPPLERLHL

>01_AE.TH.09.AA111a_WG11.JX448059

LPPLERLSL

>01_AE.TH.10.2544617P000R_Sa.MF957678

LPPLERLHL

>01_AE.TH.10.40265v14_01.KY580673

LPPLERLHL

>01_AE.TH.10.DE00110TH001.KP109513

LPPLERLHL

>01_AE.TH.11.2547010P000R_Sa.MF957618

LPPLERLHL

>01_AE.TH.11.40436v09_04.KY580697

LPPLERLHL

>01_AE.TH.93.93TH062.AB220947

LPPLERLHL

>01_AE.TH.93.93TH253.U51189

LPPLERLHL

>01_AE.TH.95.95TNIH047.AB032741

LPPLERLHL

>01_AE.TH.97.97TH_NP1525.AY713420

LPPLERLHI

>01_AE.TH.97.97TH_NP1695.AY713419

LPPLERLNL

>01_AE.TH.98.98TH_R1166.AY945728

LPPLERLQL

>01_AE.TH.99.99TH_C1080.AY945712

LPPLERLHL

>01_AE.TH.99.99TH_C2405.AY945718

LPPLERLNL

>01_AE.TH.99.99TH_NI1052.AY713423

LPPLERLTL

>01_AE.TH.99.99TH_R1149.AY945727

LPPLERLNL

>01_AE.TH.99.99TH_R3006.AY945731

LPPLERLHL

>01_AE.TH.99.99TH_R3265.AY945732

LPPLERLHL

>01_AE.TH.99.OUR008I.AY358065

LPPIERLNL

>01_AE.TH.99.OUR164I.AY358045

LPPLERLHL

>01_AE.TH.x.NP03.AB485654

LPQLEKLNL

>01_AE.US.05.306163_FL.JX863920

LPPIERLHL

>01_AE.US.98.98US_MSC2008.AY444805

LPPLEGLHL

>01_AE.VN.97.97VNAG201.FJ185245

LPPLERLQL

>01_AE.VN.97.97VNAG204.FJ185247

LPPLERLHL

>01_AE.VN.97.97VNAG210.FJ185251

LPPLERLQL

>01_AE.VN.97.97VNAG212.FJ185252

LPPLERLRL

>01_AE.VN.97.97VNAG214.FJ185253

LPPLE*LPL

>01_AE.VN.97.97VNAG218.FJ185255

LPPLERLHL

>01_AE.VN.97.97VNAG221.FJ185257

LPPLERLQL

>01_AE.VN.97.97VNHCM306.FJ185259

LPPLERLHL

>01_AE.VN.97.97VNHCM314.FJ185241

LPPLERLHL

>01_AE.VN.97.97VNHCM319.FJ185242

LPPLERLNL

>02_AG.CI.16.DE00216CI019.MH078536

LPPIERLSL

>02_AG.CI.16.DEURF16CI022.MH078539

LPPLERLSI

>02_AG.CI.99.928.EU513199

LPPIERLSL

>02_AG.CM.00.LA10GuVa.KU168265

LPPIERLSL

>02_AG.CM.01.01CM_0002BBY.AY371122

LPPLERLCL

>02_AG.CM.01.01CM_0005BBY.AY371123

LPPIERLRL

>02_AG.CM.01.01CM_0074NY.AY371131

LPPIERLSL

>02_AG.CM.01.01CM_0131NY.AY371137

LPPLERLSL

>02_AG.CM.01.01CM_0158ND.AY371132

LPPIERLCL

>02_AG.CM.01.01CM_0186BA.GU201500

LPPIERLCL

>02_AG.CM.01.01CM_0925MO.AY371134

LPPIERLCL

>02_AG.CM.01.01CM_1237NG.AY371136

LPPIERLCL

>02_AG.CM.01.01CM_1475MV.AY371138

LPPIERLCL

>02_AG.CM.01.01CM_4410HAL.AY371142

LPPLERLHL

>02_AG.CM.02.02CM_0014BBY.AY371126

LPPIERLCL

>02_AG.CM.02.02CM_0015BBY.AY371127

LPPIERLSL

>02_AG.CM.02.02CM_1669LE.AY371139

LPPIERLCL

>02_AG.CM.02.02CM_1677LE.AY371140

LPPIERLHL

>02_AG.CM.02.02CM_1901LE.AY371146

LPPIERLSI

>02_AG.CM.02.02CM_1970LE.AY371128

LPPLERLSL

>02_AG.CM.02.02CM_2162SA.AY371129

LPPIERLSL

>02_AG.CM.02.02CM_2348SA.AY371130

LPPIERLRL

>02_AG.CM.02.02CM_3153MN.GU201513

LPPIERLRL

>02_AG.CM.02.02CM_3217MN.GU201514

LPPIERLSL

>02_AG.CM.02.02CM_4082STN.AY371141

LPPIERLCL

>02_AG.CM.04.211.EU513187

LPPIERLCL

>02_AG.CM.04.235.EU513195

LPPIERLSL

>02_AG.CM.04.251.EU513196

LPPIERLSL

>02_AG.CM.04.253.EU513191

LPPIERLSL

>02_AG.CM.04.255.EU513184

LPPIERLCL

>02_AG.CM.04.263.EU513182

LPPIERLCI

>02_AG.CM.04.266.EU513193

LPPVERLRL

>02_AG.CM.04.33.EU513186

LPPIERLSL

>02_AG.CM.04.459_16.MH705136

LPPIERLRL

>02_AG.CM.05.278.EU513198

LPPIERLCL

>02_AG.CM.07.CM100_17.KU168310

LPPIERLRL

>02_AG.CM.08.BP00079_RH04.JN687776

LPPIERLSL

>02_AG.CM.08.DE00208CM001.JX140646

LPPIERLCI

>02_AG.CM.08.DE00208CM004.JX140647

LPPIERLSL

>02_AG.CM.10.DE00210CM013.KF859739

LPPIERLCL

>02_AG.CM.10.DE00210CM019.KU749419

LPPIERLCL

>02_AG.CM.97.97CM_MP807.AJ286133

LPPIERLCL

>02_AG.CM.97.CM52885.AF377954

LPPIERLSL

>02_AG.CM.99.pBD6_15.AY271690

LPPIERLCI

>02_AG.CM.x.BP00078_RH01.JN687775

LPPIERLSL

>02_AG.CY.05.CY048.FJ388902

LPPIERLRL

>02_AG.CY.07.CY206.JF683758

LPPIERLCL

>02_AG.CY.08.CY240.JF683786

LPPIEGLRL

>02_AG.CY.09.CY247.JF683792

LPPIERLCL

>02_AG.CY.09.CY252.JF683795

LPPIERLRL

>02_AG.CY.09.CY256.JF683799

LPPIERLCL

>02_AG.DE.09.701114.KT124792

LPPIERLCL

>02_AG.EC.x.ECU42.AY151002

LPPIERLCL

>02_AG.ES.06.P1261.EU786671

LPPLERLCL

>02_AG.ES.06.P1423.EU884501

LPPIERLSL

>02_AG.ES.09.P2222_2s_nt0547_9473.MF157742

LPPIERLSL

>02_AG.ES.14.100593.KY989950

LPPIERLCL

>02_AG.ES.99.R74_s_nt0545_9481.MF157740

LPPIERLSL

>02_AG.FR.91.DJ263.AF063223

LPPIERLCL

>02_AG.FR.91.DJ264.AF063224

LPPLERLCL

>02_AG.GB.13.13592_1_38.3.MF109381

LPPIERLCI

>02_AG.GB.13.15171_1_26.3.MF109657

LPPIERLCL

>02_AG.GB.13.15228_1_71.3.MF109709

LPPIERLCL

>02_AG.GB.14.13612_1_7.4.MF109432

LPPIERLCL

>02_AG.GB.14.13659_1_50.3.MF109439

LPPLERLSL

>02_AG.GB.14.14592_1_56.3.MF109550

LPPIERLYI

>02_AG.GB.14.14592_1_65.3.MF109559

LPPIERLCL

>02_AG.GH.03.03GH181AG.AB286856

LPPIERLHL

>02_AG.GH.03.03GH182AG.AB286857

LPPIERLSL

>02_AG.GH.03.03GH184AG.AB286859

LPPIERLCL

>02_AG.GH.03.03GH189AG.AB286862

LPPIERLTL

>02_AG.GH.03.03GH197AG.AB286863

LPPIERLSL

>02_AG.GH.03.GHNJ185.AB231895

LPPIERLCI

>02_AG.GH.03.GHNJ188.AB231896

LPPIERLCL

>02_AG.GH.03.GHNJ196.AB231898

LPPIERLTL

>02_AG.GH.97.97GH_AG1.AB049811

LPPLERLSL

>02_AG.GH.x.I_2496.AB485633

LPPLERLCL

>02_AG.GW.05.CC_0048.FJ694792

LPPLERLSL

>02_AG.GW.14.DE00214GW002.MH078541

LPPLERLSL

>02_AG.GW.14.DE00214GW005.MH078542

LPPIERLCL

>02_AG.GW.14.DE00214GW007.MH078543

LPPIERLCL

>02_AG.GW.14.DE00214GW009.MH078544

LPPIERLCL

>02_AG.GW.14.DE00214GW020.MH078545

LPPIERLCL

>02_AG.KR.12.12MHI11_10746.KF561437

LPPIERLNL

>02_AG.KR.12.12MHR9.KF561435

LPPIERLSL

>02_AG.LR.x.POC44951.AB485636

LPPIERLCL

>02_AG.NG.01.PL0710.DQ168577

LPPIERLSL

>02_AG.NG.09.09NG010060.KX389639

LPPIERLRL

>02_AG.NG.09.09NG010170.KX389630

LPPIERLSL

>02_AG.NG.09.09NG010181.KX389629

LPPIERLSL

>02_AG.NG.09.09NG010325.KX389624

LPPIERLSL

>02_AG.NG.09.09NG_SC24.JN248585

LPPIGRLSL

>02_AG.NG.09.09NG_SC29.JN248589

LPPIERLNL

>02_AG.NG.09.09NG_SC30.JN248590

LPPIERLNL

>02_AG.NG.09.09NG_SC61.JN248592

LPPIERLSL

>02_AG.NG.10.10NG020065.KX389621

LPPLERLRL

>02_AG.NG.10.10NG020307.KX389617

LPPIERLRL

>02_AG.NG.10.10NG020437.KX389614

LPPIERLSL

>02_AG.NG.10.10NG030161.KX389610

LPPIERLNL

>02_AG.NG.11.11NG050135.KX389640

LPPIERLSL

>02_AG.NG.11.11NG050356.KX389643

LPPIERLNL

>02_AG.NG.11.11NG050366.KX389644

LPPIERLCI

>02_AG.NG.11.DE00211NG009.KY953197

LPPIERLTL

>02_AG.NG.11.DE00211NG010.KY953198

LPPIERLTL

>02_AG.NG.12.12NG060304.KX389647

LPPIERLCL

>02_AG.NG.12.12NG060418.KX389649

LPPLERLCL

>02_AG.NG.x.IBNG.L39106

LPPIERLNL

>02_AG.PK.14.PK005.KX232598

LPPIERLCL

>02_AG.PK.15.DE00215PK035.KY658712

LPPIERLRL

>02_AG.PK.15.PK024.KX232616

LPPLENLHL

>02_AG.PK.15.PK032.KX232622

LPPLERLCL

>02_AG.SE.11.SE602019.KP411843

LPPIERLSL

>02_AG.SE.11.SE602020.KP411844

LPPIERLRL

>02_AG.SE.11.SE602024.KP411845

LPPIERLHL

>02_AG.SE.14.098GN.MF373200

LPPIERLSL

>02_AG.SE.94.SE7812.AF107770

LPPIERLRL

>02_AG.SN.13.5294.KT223759

LPPLERLSL

>02_AG.SN.13.9580.KT223760

LPPLEKLCI

>02_AG.SN.98.98SE_MP1211.AJ251056

LPPIERLCL

>02_AG.SN.98.98SE_MP1213.AJ251057

LPPIERLTL

>02_AG.TH.07.AA039a10R.JX447154

LPPLERLSL

>02_AG.UG.10.DEURF10UG006.KF716488

LPPIERLRI

>02_AG.US.00.00US_MSC3083.AY444811

LPPIERLSL

>02_AG.US.06.502_2696_FL01.JF320297

LPPIERLRL

>02_AG.US.99.99US_MSC1134.AY444809

LPPLERLSL

>02_AG.x.00.LA11ZaCh.KU168266

LPPLERLCI

>02_AG.x.00.LA12FroG.KU168267

LPPIERLSL

>03_AB.RU.97.KAL153_2.AF193276

LPPLERLTL

>04_cpx.CY.94.94CY032_3.AF049337

LPPLEKLTL

>04_cpx.GR.00.BP00048_SUP_RH05.JN687742

LPPLERLTL

>04_cpx.GR.00.DE00400GR002.JX140648

LPPLERLTL

>04_cpx.GR.04.BP00052_SUP_RH01.JN687746

LPPIEKLSL

>04_cpx.GR.04.BP00053_SUP_RH01.JN687748

LPPLEKLTL

>04_cpx.GR.91.GR11_97PVCH.AF119820

LPPLEKLTL

>04_cpx.GR.97.GR84_97PVMY.AF119819

LPPIEKLTL

>05_DF.BE.93.VI961.AF076998

LPPLERLNL

>05_DF.BE.x.VI1310.AF193253

LPPLERLNL

>05_DF.ES.99.X492.AY227107

LPPLERLTL

>06_cpx.AU.96.BFP90.AF064699

LPPIERLRL

>06_cpx.GB.13.13592_1_3.8.MF109383

LPPIERLHL

>06_cpx.GH.03.03GH173_06.AB286852

LPPIERLHL

>06_cpx.ML.95.95ML127.AJ288982

LPPIERLRL

>06_cpx.ML.95.95ML84.AJ245481

LPPIERLRL

>06_cpx.NG.10.10NG030498.KX389609

LPPLERLSL

>06_cpx.SN.97.97SE1078.AJ288981

LPPIERLRL

>07_BC.CN.03.BC14_BJ25_51.EU363844

LPPLERLHI

>07_BC.CN.05.BC07_YN109_6.EU363837

LPPLERLHI

>07_BC.CN.05.XJDC6431_2.EF368372

LPPLERLHI

>07_BC.CN.05.XJDC6441.EF368370

LPPIERLHI

>07_BC.CN.05.XJN0084.EF368371

LPPIERLHI

>07_BC.CN.05.pXJDC6291_13.KC492737

LPPIERLHI

>07_BC.CN.06.Sichuan_2006_SC006.JX392378

LPPIEGLHI

>07_BC.CN.06.Sichuan_2006_SC008.JX392379

LPPIERLHI

>07_BC.CN.06.Xinjiang_2006_709.JX392383

LPPIERLHI

>07_BC.CN.06.Xinjiang_2006_713.JX392384

LPPIERLHI

>07_BC.CN.07.07CNYN303.KF835494

LPPLEGLHI

>07_BC.CN.07.07CNYN323.KF835510

LPPIERLHI

>07_BC.CN.07.07CNYN324.KF835511

LPPIEGLHI

>07_BC.CN.07.07CNYN349.KF835532

LPPIERLHI

>07_BC.CN.07.BJOX002000.e03.KM218237

LPPLERLHI

>07_BC.CN.07.CNGZD.JQ423923

LPPLERLHI

>07_BC.CN.07.GD070145.KF250371

LPPIERLHI

>07_BC.CN.07.HB070037.KF250373

LPSLERLHI

>07_BC.CN.07.JS070205.KF250374

LPPLERLHI

>07_BC.CN.07.NX070014.KF250375

LPPIERLHI

>07_BC.CN.07.XJ070248.KF250377

LPPIERLHI

>07_BC.CN.07.xj070241.KF250385

LPPIERLHI

>07_BC.CN.08.1114.HQ215552

LPPLERLCI

>07_BC.CN.09.09LNA446.JX960600

LPPLERLCI

>07_BC.CN.09.09LNA745.JX960602

LPPLERLCI

>07_BC.CN.x.BC12_SC142_6.EU363842

LPPIERLNI

>07_BC.MM.13.mSSDU163.KU820832

LPPLERLYI

>07_BC.TW.13.pCRF07.KF234628

LPPLERLHI

>08_BC.CN.00.p00CH_WS035_08_BC51.AB746344

LPPLERLHI

>08_BC.CN.05.BC06_YNP45_45.EU363836

LPPLERLHI

>08_BC.CN.06.BC01_YN161_5.EU363831

LPPLERLHI

>08_BC.CN.07.07CNYN302.KF835493

LPPLERLHI

>08_BC.CN.07.07CNYN306.KF835495

LPPLERLHI

>08_BC.CN.07.07CNYN307.KF835496

LPPLERLHI

>08_BC.CN.07.07CNYN310.KF835497

LPPLERLHI

>08_BC.CN.07.07CNYN313.KF835500

LPPLERLSI

>08_BC.CN.07.07CNYN314.KF835501

LPPLERLSI

>08_BC.CN.07.07CNYN319.KF835506

LPPLERLNI

>08_BC.CN.07.07CNYN320.KF835507

LPPLERLHI

>08_BC.CN.07.07CNYN322.KF835509

LPPLERLHI

>08_BC.CN.07.07CNYN325.KF835512

LPPLERLHI

>08_BC.CN.07.07CNYN344.KF835529

LPPLERLHI

>08_BC.CN.07.07CNYN346.KF835530

LPPLERLHL

>08_BC.CN.07.07CNYN350.KF835531

LPPLERLHI

>08_BC.CN.07.07CNYN351.KF835535

LPPLERLHI

>08_BC.CN.07.07CNYN355.KF835534

LPPLERLHI

>08_BC.CN.07.07CNYN363.KF835541

LPPLERLHI

>08_BC.CN.07.07CNYN369.KF835545

LPPLERLNI

>08_BC.CN.07.07CNYN371.KF835547

LPPLERLTI

>08_BC.CN.07.2007CNGX_HK.JF719819

LPPLERLHI

>08_BC.CN.07.GD070077.KF250369

LPPLERLHI

>08_BC.CN.97.97CNGX_9F.AY008717

LPPLERLHI

>08_BC.MM.13.mSSDU220.KU820840

LPPLERLNI

>08_BC.MM.13.mSSDU91.KU820846

LPPLERLNI

>09_cpx.CI.00.00IC_10092.AJ866553

LPPLEKLHL

>09_cpx.SN.95.95SN7808.AY093604

LPPLERLHL

>09_cpx.US.99.99DE4057.AY093607

LPPLERLHI

>103_01B.CN.15.HE150308.MH388438

LPPLERLHL

>10_CD.TZ.96.96TZ_BF071.AF289549

LPPLERLNL

>10_CD.TZ.96.96TZ_BF110.AF289550

LPPLERLNL

>11_cpx.CM.01.01CM_0186ND.AY371149

LPPIERLHI

>11_cpx.CM.02.02CM_2190SA.AY371151

LPPLERLSL

>11_cpx.CM.02.02CM_4118STN.AY371153

LPPIERLHL

>11_cpx.CM.02.A1575.KP718934

LPPIENLHI

>11_cpx.CM.02.A1774.KP718935

LPPIERLHL

>11_cpx.CM.04.119_28.KP718937

LPPIERLHL

>11_cpx.CM.04.1230_24.KP718938

LPQIERLHI

>11_cpx.CM.04.260_50.KP718936

LPPIERLRL

>11_cpx.CM.06.263_26.KP718914

LPPIERLHL

>11_cpx.CM.07.62_11.KR822830

LPPIERLSL

>11_cpx.CM.08.1252_11.KP718929

LPPLERLHI

>11_cpx.CM.11.DE01111CM045.KY658700

LPPIERLHL

>11_cpx.CM.11.DEURF11CM015.KP109503

LPPLERLSL

>11_cpx.CM.95.95CM_1816.AF492624

LPPLERLNL

>11_cpx.CM.96.96CM_4496.AF492623

LPPIERLXL

>11_cpx.CM.97.MP818.AJ291718

LPPIERLHL

>11_cpx.CY.09.CY259.JF683802

LPPIEKLHL

>11_cpx.FR.99.MP1298.AJ291719

LPPIERLHL

>11_cpx.FR.99.MP1307.AJ291720

LPPIERLHL

>11_cpx.GR.x.GR17.AF179368

LPPIERLHL

>11_cpx.NG.09.09NG010131.KX389633

LPPIERLHL

>11_cpx.SE.16.104CM.MF373206

LPPIERLSL

>11_cpx.x.03.LA15PoCa.KU168270

LPPIERLHL

>12_BF.AR.97.A32879.AF408629

LPPLERLTL

>12_BF.AR.97.A32989.AF408630

LPPLERLTL

>12_BF.AR.99.ARMA159.AF385936

LPPLERLTL

>12_BF.ES.00.X0531_2_nt0555_9485.MF157739

LPPLERLTL

>12_BF.PE.13.DEURF13PE006.MH078556

LPPLDRLTL

>12_BF.UY.99.URTR23.AF385934

LPPLERLTL

>12_BF.UY.99.URTR35.AF385935

LPPLERLTL

>13_cpx.CM.02.02CM_3226MN.AY371154

LPPLERLHI

>13_cpx.CM.02.02CM_A1394.DQ845388

LPPIERLHI

>13_cpx.CM.04.04CM_173_9.DQ845386

LPPLERLHL

>13_cpx.CM.04.04CM_632_28.DQ845387

LPPLERLRL

>13_cpx.CM.06.363_24.MH705141

LPPIERLHL

>13_cpx.CM.08.228_10.KP718924

LPPLERLHI

>13_cpx.CM.08.833_62.KP718926

LPPLERLHL

>13_cpx.CM.96.96CM_1849.AF460972

LPPLERLHL

>13_cpx.CM.96.96CM_4164.AF460974

LPPLERLHL

>13_cpx.GB.14.13612_1_33.3.MF109417

LPPLERLHI

>14_BG.ES.00.X477.AF423759

LPPLERLHL

>14_BG.ES.00.X623.AF450097

LPPLERLHL

>15_01B.TH.02.02TH_OUR1331.AF529572

LPPLERLKL

>15_01B.TH.02.02TH_OUR1332.AF529573

LPPLERLKL

>15_01B.TH.04.04TH409819.JN248325

LPPLERLSL

>15_01B.TH.05.05TH522586.JN248351

LPPLERLTL

>15_01B.TH.05.05TH637314.JN248352

LPPLERLTL

>15_01B.TH.99.99TH_MU2079.AF516184

LPPLERLSL

>15_01B.TH.99.99TH_R2399.AF530576

LPPLERLHL

>16_A2D.KE.05.05KE493170V5.KT022403

LPPLERLHL

>16_A2D.KE.05.05KE725124V4.KT022406

LPPLERLHL

>16_A2D.KE.91.KNH1271.AY945736

LPPIERLHI

>16_A2D.KR.97.97KR004.AF286239

LPPLERLHL

>17_BF.AR.02.AR02_ARG1139.EU581825

LPPLERLTL

>17_BF.AR.02.AR02_ARG2233.EU581826

LPPLERLTL

>17_BF.AR.99.ARMA038.AY037281

LPPLERLTL

>17_BF.BO.02.BO02_BOL119.EU581827

LPPLERLTL

>17_BF.PE.02.PE02_PCR0155.EU581828

LPPIERLTL

>17_BF.PY.02.PY02_PSP0073.EU581824

LPPLDRLTL

>17_BF.PY.02.PY02_PSP0096.EU581823

LPPIEKLTL

>18_cpx.CM.11.B4043_15.KP718931

LPPLERLNL

>18_cpx.CM.97.CM53379.AF377959

LPPLERLNL

>18_cpx.CU.99.CU14.AY586541

LPPLERLTL

>18_cpx.CU.99.CU68.AY894993

LPPLERLTL

>18_cpx.CU.99.CU76.AY586540

LPPLERLTL

>18_cpx.GB.13.13592_1_35.4.MF109378

LPPLERLNL

>19_cpx.CU.99.CU29.AY588971

LPPLERLHL

>19_cpx.CU.99.CU38.AY588970

LPPLERLHL

>19_cpx.CU.99.CU7.AY894994

LPPLERLHL

>19_cpx.ES.14.EUR_0040.KU685581

LPPLERLHL

>19_cpx.ES.14.EUR_0041.KU685582

LPPLERLHL

>20_BG.ES.14.ARP1210.KT276270

LPPLDRLTL

>20_BG.ES.99.R77.AY586544

LPPLERLTL

>21_A2D.KE.04.QG393_60M_ENV_A1.FJ866128

LPPLERLNL

>21_A2D.KE.91.KNH1254.AY945737

LPPLERLNL

>21_A2D.KE.99.KER2003.AF457051

LPPIERLNL

>21_A2D.KE.99.KSM4001.AF457072

LPPIERLNL

>22_01A1.CM.01.01CM_0001BBY.AY371159

LPPLERLHL

>22_01A1.CM.02.02CM_1867LE.AY371165

LPPLERLHL

>22_01A1.CM.02.02CM_3097MN.GQ229529

LPPLERLHL

>22_01A1.CM.10.LB005.JN864050

LPPLERLHI

>22_01A1.CM.10.LB011.JN864051

LPPIERLSL

>22_01A1.CM.10.LB013.JN864058

LPPLERLHI

>22_01A1.CM.10.LB054.JN864059

LPPLERLHL

>23_BG.CU.03.CB118.AY900571

LPPLERLTL

>23_BG.CU.03.CB347.AY900572

LPPIERLTL

>24_BG.CU.03.CB378.AY900574

LPPLERLTL

>24_BG.CU.03.CB471.AY900575

LPPLERLTL

>25_cpx.CM.01.101BA.DQ826726

LPPLERLHI

>25_cpx.CM.02.1918LE.AY371169

LPPLERLHL

>25_cpx.SA.03.J11233.EU697906

LPPLERLHL

>25_cpx.SA.03.J11451.EU697908

LPPLERLHI

>26_A5U.CD.02.02CD_LBTB084.FM877781

LPPLERLTL

>26_A5U.CD.02.02CD_MBTB047.FM877782

LPPLERLSL

>26_A5U.CD.97.97CD_KTB119.FM877777

LPPLERLHL

>27_cpx.CD.97.97CDKTB49.AJ404325

LPPIERLCL

>27_cpx.FR.04.04CD_FR_KZS.AM851091

LPPLERLCL

>28_BF.BR.05.0614SV.JF804809

LPPLDRLTI

>28_BF.BR.05.0679SV.JF804812

LPPLERLTL

>28_BF.BR.99.BREPM12313.DQ085872

LPPLERLTL

>28_BF.BR.99.BREPM12609.DQ085873

LPPIERLTL

>28_BF.BR.99.BREPM12817.DQ085874

LPPLERLTL

>29_BF.BR.01.BREPM16704.DQ085876

LPPIERLTL

>29_BF.BR.02.BREPM119.AY771590

LPPLERLTL

>29_BF.BR.05.0063SP.JF804806

LPPLERLSL

>29_BF.BR.05.0264RI.JF804807

LPPLERLTL

>29_BF.BR.05.0647SV.JF804811

LPPIERLTL

>29_BF.BR.05.0744SV.JF804814

LPPLERLTL

>29_BF.BR.99.99UFRJ_1.AY455778

LPPLERLTL

>29_BF.BR.99.BREPM11948.DQ085871

LPPIERLTL

>31_BC.BR.02.110PA.EF091932

LPPIERLNI

>31_BC.BR.04.04BR137.AY727526

LPPIERLTI

>31_BC.BR.04.04BR142.AY727527

LPPIERLNI

>32_06A6.EE.01.EE0369.AY535660

LPPIERLRL

>32_06A6.EE.06.A14.KM606632

LPPIEELRL

>33_01B.ID.07.JKT189_C.AB547463

LPPLERLSL

>33_01B.MY.05.05MYKL007_1.DQ366659

LPPIERLNL

>33_01B.MY.05.05MYKL015_2.DQ366660

LPPLERLTL

>33_01B.MY.05.05MYKL045_1.DQ366662

LPPLERLTL

>33_01B.MY.07.07MYKLD47.EU031913

LPPLERLTL

>34_01B.TH.99.OUR2478P.EF165541

LPPLERLTL

>35_AD.AF.05.05AF026.EF158043

LPSLERLNL

>35_AD.AF.05.05AF094.EF158040

LPPLERLHL

>35_AD.AF.05.05AF104.EF158042

LPPLERLHL

>35_AD.AF.06.047H.GQ477443

LPPLERLHL

>35_AD.AF.06.051H.GQ477444

LPPLEKLHL

>35_AD.AF.06.077H.GQ477442

LPPLERLRI

>35_AD.AF.07.169H.GQ477446

LPPLERLQL

>35_AD.AF.07.273H.GQ477448

LPPLERLHL

>35_AD.IR.10.10IR.THR09F.AB703608

LPPLERLHL

>35_AD.IR.10.10IR.THR41F.AB703615

LPPLERLHL

>35_AD.IR.11.11IR.KSH24F.AB703609

LPPLERLHL

>35_AD.IR.11.11IR.KSH29F.AB703611

LPPIERLHL

>35_AD.IR.11.11IR.KSH31F.AB703612

LPPLERLXL

>35_AD.IR.11.11IR.SYZ38F.AB703614

LPPLERLHL

>36_cpx.CM.00.00CMNYU1162.EF087995

LPPLERLHL

>36_cpx.CM.07.BS40.KR017774

LPPLERLHL

>37_cpx.CM.00.00CMNYU926.EF116594

LPPLERLHL

>37_cpx.CM.06.1130_39.KP718917

LPPLERLNL

>37_cpx.CM.97.CM53392.AF377957

LPPLERLHI

>37_cpx.CY.07.CY192.JF683745

LPSLERLHI

>38_BF.UY.99.99UY_TRA0123.JN235962

LPPLERLHI

>38_BF1.UY.03.UY03_3389.FJ213783

LPPLERLHI

>38_BF1.UY.04.UY04_3987.FJ213781

LPPIDQLRI

>38_BF1.UY.04.UY04_4022.FJ213782

LPPLERLHI

>38_BF1.UY.05.UY05_4752.FJ213780

LPPLERLHI

>39_BF.BR.03.03BRRJ103.EU735534

LPPLERLHI

>39_BF.BR.03.03BRRJ327.EU735536

LPPLERLHI

>39_BF.BR.04.04BRRJ179.EU735535

LPPLERLHI

>40_BF.BR.04.04BRRJ115.EU735538

LPPLERLTL

>40_BF.BR.04.04BRSQ46.EU735540

LPPLERLTL

>40_BF.BR.05.05BRRJ200.EU735539

LPPLERLTL

>41_CD.TZ.05.CO6577V5.KX907411

LPPLERLHI

>42_BF.LU.03.luBF_05_03.EU170155

LPPLERLTL

>43_02G.GB.13.15171_1_18.3.MF109648

LPPLERLHL

>43_02G.SA.03.J11223.EU697904

LPPLERLHL

>43_02G.SA.03.J11232.EU697905

LPPLERLHL

>43_02G.SA.03.J11243.EU697907

LPPLERLHL

>43_02G.SA.03.J11456.EU697909

LPPLERLHL

>44_BF.CL.00.CH80.FJ358521

LPPLERLHI

>44_BF.CL.01.CH12.AY536235

LPPLERLNI

>44_BF.PE.16.DEURF16PE007.MH078557

LPPLERLHI

>45_cpx.FR.04.04FR_AUK.EU448295

LPPLERLHL

>46_BF.BR.01.01BR125.DQ358802

LPPLERLHI

>46_BF.BR.01.01BRRJUD508.MG365771

LPPLERLHI

>46_BF.BR.07.07BR_FPS625.HM026456

LPPLERLHI

>46_BF.BR.07.07BR_FPS742.HM026457

LPPLERLHI

>46_BF.BR.07.07BR_FPS783.HM026458

LPPLERLHI

>46_BF.BR.07.07BR_FPS810.HM026459

LPPLERLHI

>46_BF.BR.07.07BR_FPS812.HM026460

LPPLERLHI

>47_BF.BR.10.10BR_RJ026.KJ849798

LPPLERLTL

>47_BF.ES.08.P1942.GQ372987

LPPLERLTL

>47_BF.ES.08.X2457_2.FJ670529

LPPIERLTL

>48_01B.MY.07.07MYKT014.GQ175881

LPPLERLTL

>48_01B.MY.07.07MYKT016.GQ175882

LPPLERLTL

>48_01B.MY.07.07MYKT021.GQ175883

LPPLERLTL

>49_cpx.BW.98.BW2117.AF192135

LPPIERLRL

>49_cpx.GM.02.N18380.HQ385477

LPPIERLSL

>49_cpx.GM.03.N26677.HQ385479

LPSLERLCL

>50_A1D.GB.00.8179.JN417236

LPPLERLNL

>50_A1D.GB.03.33365.JN417239

LPPLERLNL

>50_A1D.GB.10.11762.JN417241

LPPLERLTL

>50_A1D.GB.10.12792.JN417240

LPPLERLNL

>51_01B.MN.12.12MNG12712.LC312714

LPPLERLTL

>51_01B.MY.11.11MYKL055.KJ485697

LPPIERLTL

>51_01B.SG.11.11SG_HM021.JN029801

LPPLERLTL

>52_01B.MY.03.03MYKL018_1.DQ366664

LPPLERLTL

>52_01B.TH.00.00TH_R1741.AY945734

LPPLERLTL

>53_01B.MY.10.10MYKJ067.JX390612

LPSLERLCL

>53_01B.MY.10.10MYKJ079.JX390611

LPPLERLHL

>53_01B.MY.11.11FIR164.JX390610

LPPIERLNL

>54_01B.MY.07.07MYKLD49.EU031915

LPPIERLHL

>54_01B.MY.08.08MYKL044.JX390977

LPPLERLHL

>54_01B.MY.09.09MYSB023.JX390976

LPPIERLHL

>55_01B.CN.08.08CYM047.JF340054

LPPLERLHI

>55_01B.CN.10.HNCS102056.JX574661

LPPLERLHI

>55_01B.CN.11.ANHUI_FY64.KC183777

LPPLERLHI

>55_01B.CN.11.GDDG318.JX574662

LPPLERLHI

>56_cpx.FR.10.URF5_patient_A.JN882655

LPPIERLCL

>57_BC.CN.07.341.HM776939

LPPIERLHI

>57_BC.CN.09.09YNLC216002sg.KC898985

LPPLERLHI

>57_BC.CN.09.09YNLC496sg.KC898991

LPPIEKLHI

>57_BC.CN.09.09YNLX19sg.KC899008

LPPLEGLHI

>57_BC.CN.09.1439.JX679207

LPPIERLHI

>57_BC.CN.09.YNFL37.KC870044

LPPIERLHI

>57_BC.CN.10.DH17.KF250400

LPPIERLNI

>58_01B.MY.09.09MYPR37.KC522031

LPPLERLHL

>58_01B.MY.10.10MYKJ036.KC522035

LPPLERLHL

>58_01B.MY.10.10MYPR87.KF425293

LPPLERLHL

>58_01B.MY.11.11MY1RJ704.KC522033

LPPLERLHL

>58_01B.MY.11.11MY1ZK731.KC522032

LPPLERLNL

>59_01B.CN.07.GD070126.KF011494

LPPLERLHL

>59_01B.CN.09.09LNA423.JX960635

LPPLERLHL

>59_01B.CN.11.11CN.LNSY300876.KJ484434

LPPLERLHL

>60_BC.IT.11.BAV499.KC899079

LPPIERLNI

>60_BC.IT.11.BAV514.KC899080

LPPIERLNI

>60_BC.IT.11.BAV636.KC899081

LPPIERLNI

>61_BC.CN.10.JL100010.KC990124

LPPIEGLHI

>62_BC.CN.10.YNFL13.KC870034

LPPIERLHI

>62_BC.CN.10.YNFL15.KC870035

LPPIERLHI

>63_02A.RU.10.10RU6637.JN230353

LPPIERLSL

>63_02A.RU.13.RU_8169.KJ197201

LPSIERLSL

>63_02A.RU.13.RU_8501.KJ197202

LPSIERLSL

>64_BC.CN.09.09YNLX047sg.KC898994

LPPIEKLHI

>64_BC.CN.09.09YNLX219037sg.KC899009

LPPIERLHI

>64_BC.CN.09.YNFL31.KC870042

LPPLERLHI

>64_BC.CN.09.YNFL33.KC870043

LPPIERLHI

>64_BC.CN.10.YNFL10_1.KC870032

LPPIERLHI

>64_BC.CN.10.YNFL16.KC870036

LPPIERLHI

>64_BC.CN.10.YNFL22.KC870040

LPPIERLHI

>65_cpx.CN.10.YNFL01.KC870027

LPPIERLYI

>65_cpx.CN.10.YNFL02.KC870028

LPPIERLHI

>65_cpx.CN.11.ANHUI_HF104.KC183778

LPPIERLHI

>65_cpx.CN.x.JL15030.MH051841

LPPIERLHI

>67_01B.CN.11.ANHUI_HF115.KC183779

LPPLERLHL

>68_01B.CN.10.JS2010001.KF758551

LPPLERLHL

>68_01B.CN.11.ANHUI_WH73.KC183782

LPPLDRLSL

>69_01B.JP.03.03JP_5091K231.AB845344

LPPLERLHL

>69_01B.JP.05.05JPMYC113SP420.LC027100

LPPLERLHL

>69_01B.JP.05.05JP_5091K448.AB845347

LPPLERLHL

>69_01B.JP.10.10JP_5091N172.AB845348

LPPLERLSL

>69_01B.JP.10.10JP_5091N200.AB845349

LPPLERLHL

>70_BF1.BR.10.10BR_PE004.KJ849758

LPPIERLTL

>70_BF1.BR.10.10BR_PE016.KJ849761

LPPLERLTL

>70_BF1.BR.10.10BR_PE025.KJ849762

LPPLERLTL

>70_BF1.BR.10.DE07010BR033.KU749388

LPPLERLTL

>71_BF1.BR.02.02BR033.DQ358811

LPPLERLTL

>71_BF1.BR.10.10BR_PE008.KJ849759

LPQLERLTL

>71_BF1.BR.10.10BR_PE009.KJ849760

LPPLERLSL

>71_BF1.BR.10.10BR_PE026_2.KJ849763

LPPLDRLTL

>71_BF1.BR.10.10BR_PE064.KJ849769

LPPIERLTL

>71_BF1.BR.10.10BR_PE066.KJ849770

LPPLERLTL

>71_BF1.BR.10.10BR_PE071.KJ849771

LPPLERLTL

>71_BF1.BR.10.10BR_PE084.KJ849773

LPPLERLTL

>71_BF1.BR.10.10BR_PE087.KJ849775

LPPLERLTL

>71_BF1.BR.10.10BR_PE088.KJ849776

LPPLERLTL

>71_BF1.BR.10.10BR_PE090.KJ849777

LPPLERLTL

>71_BF1.BR.10.10BR_PE092.KJ849778

LPPLERLTL

>71_BF1.BR.10.10BR_SP026.KT427816

LPPLERLTL

>72_BF1.BR.10.10BR_MG002.KJ671534

LPPLERLTL

>72_BF1.BR.10.10BR_MG003.KJ671533

LPPLERLTL

>72_BF1.BR.10.10BR_MG004.KJ671535

LPPLERLTL

>72_BF1.BR.10.10BR_MG005.KJ671537

LPPIERLTL

>72_BF1.BR.10.10BR_MG008.KJ671536

LPPLERLTL

>73_BG.ES.11.X3208.KM248765

LPPLERLHL

>74_01B.MY.10.10MYKJ052.KR019770

LPPLERLTL

>74_01B.MY.10.10MYPR268.KR019771

LPPLERLSL

>74_01B.MY.11.11MYPR416.KR019772

LPPIERLNL

>77_cpx.MY.13.13MYNBB108.KX673818

LPPLERLNL

>77_cpx.MY.14.14MYNBB090.KX673820

LPPLERLNL

>78_cpx.CN.13.YNTC19.KU161143

LPPLERLTL

>78_cpx.CN.13.YNTC35.KU161144

LPPLERLTL

>78_cpx.CN.13.YNTC88.KU161145

LPPLERLHL

>79_0107.CN.15.SX15DT013.KY216146

LPPLERLCI

>79_0107.CN.15.SX15JC06.KY216147

LPPLERLCI

>80_0107.CN.11.YA285.MH843712

LPPLERLQL

>82_cpx.MM.13.mSSDU12.KU820825

LPPLERLTL

>82_cpx.MM.13.mSSDU160.KU820831

LPPLERLTL

>83_cpx.MM.13.mSSDU109.KU820823

LPPLERLHL

>83_cpx.MM.13.mSSDU118.KU820824

LPPLERLHL

>83_cpx.MM.13.mSSDU151.KU820829

LPPLERLHL

>85_BC.CN.14.14CN_SCYB11.KU992935

LPPLERLHI

>85_BC.CN.14.14CN_SCYB12.KU992936

LPPLERLNI

>85_BC.CN.14.14CN_SCYB18.KU992937

LPPLERLHI

>85_BC.CN.14.14CN_SCYB1.KU992928

LPPLERLHI

>85_BC.CN.14.14CN_SCYB20.KU992930

LPPLERLHI

>85_BC.CN.14.14CN_SCYB2.KU992929

LPPLERLHI

>85_BC.CN.14.14CN_SCYB3.KU992931

LPPLERLHI

>85_BC.CN.14.14CN_SCYB4.KU992932

LPPIERLHI

>85_BC.CN.14.14CN_SCYB7.KU992934

LPPLERLHI

>86_BC.CN.13.15YNHS18.KX582249

LPPLERLTL

>86_BC.CN.13.15YNHS23.KX582250

LPPLERLTL

>86_BC.CN.13.15YNHS26.KX582251

LPPIERLTL

>87_cpx.CN.09.09YNLC497sg.KC898992

LPPLERLHL

>87_cpx.CN.09.09YNRL215050sg.KC899012

LPPLERLHL

>87_cpx.CN.12.DH32.KF250408

LPPLERLHL

>88_BC.CN.05.05YNRL07sg.KC898975

LPPLERLTL

>88_BC.CN.05.05YNRL25sg.KC898979

LPPLERLTL

>88_BC.CN.09.DH19.KF250402

LPPLERLTL

>89_BF.BO.99.BOL0137.AY037271

LPPLERLTL

>89_BF.ES.10.P2633.KX818199

LPPLERLTL

>89_BF.ES.12.P3177.KX818200

LPPLEGLTL

>90_BF1.BR.07.BRGO3047.KY628216

LPPLERLTL

>90_BF1.BR.07.BRGO3145.KY628218

LPPLERLTL

>90_BF1.BR.09.BRTO10_66.KY628225

LPPLERLTL

>90_BF1.BR.10.BRG04141.KY628219

LPPLERLTL

>90_BF1.BR.10.BRGOAP801.KY628223

LPPLERLSL

>90_BF1.BR.11.BRGO6043.KY628221

LPPLERLSL

>91_01C.CN.13.YNLC27.MH909568

LPPLERLTL

>91_01C.CN.13.YNLC28.MH909569

LPPLERLRL

>91_01C.CN.13.YNLC30.MH909570

LPPLERLTL

>92_C2U.CD.02.CG_0151_02V_NGSID1.KY392767

LPPIERLCI

>92_C2U.CD.08.DRC699.MF372647

LPPIERLCI

>92_C2U.CD.08.DRC796.MF372645

LPPLDRLSL

>92_C2U.CD.08.DRC819.MF372648

LPPIERLCI

>92_C2U.x.04.LA08SySa.KU168263

LPPIDRLRI

>93_cpx.CD.08.DRC367.MF372646

LPPIERLSI

>93_cpx.CD.08.DRC653.MF372651

LPPIERLSI

>93_cpx.CD.08.DRC817.MF372649

LPPIERLSI

>94_cpx.FR.14.24FR0113_MW71FOBV.MH141493

LPPLERLTL

>94_cpx.FR.16.05FR0916_MW95FOBV.MH141491

LPPLERLTL

>96_cpx.CN.10.JL.RF01.KF850149

LPPLERLHL

>96_cpx.CN.13.13YNBS54IDU.MG518476

LPPLERLHL

>96_cpx.CN.13.13YNBS66IDU.MG518477

LPPLERLHL

>98_06B.FR.09.A_Bordeaux.MH479275

LPPIERLRL

>0102A.CM.01.01CM_0190MA.AY371145

LPPIERLCL

>0102A.CM.07.567_16.KP718921

LPPIERLTL

>0102A.CM.07.663_13.KP718922

LPPIERLCI

>0102A1.CY.07.CY196.JF683748

LPPLERLHL

>0107.CN.07.JL070032.KC990127

LPPLERLQL

>0107.CN.07.MSM0720.KC833436

LPPLERLCI

>0107.CN.10.10LNA015.KU051564

LPPIERLHI

>0107.CN.10.JL.RF03.KJ184176

LPPLERLQL

>0107.CN.12.kang124_NFL.KJ778897

LPPLERLNL

>0107.CN.13.BJMP3002.KM974719

LPPLERLHL

>0107.CN.13.BJMP3026.KM974720

LPPLERLNL

>0107.CN.14.BJ2015EU16.MH029899

LPPLERLCI

>0107.CN.14.GXDY460B.KT619126

LPPIERLHI

>0107.CN.14.LN301538.KX434794

LPPLERLTL

>0107.CN.14.XC2014EU09.KT592380

LPPLERLHL

>0107.CN.15.15zj016.KX185086

LPPLERLYI

>0107.CN.15.BJ2015EU19.KY950610

LPPLERLCI

>0107.CN.15.LN321945.KX434796

LPPLERLTL

>0107.CN.15.M62.MH396608

LPPLERLQL

>0107.CN.15.SN121.MF373389

LPPLERLNL

>0107.CN.15.YA1996_00_NFLG.MF084205

LPPLERLCI

>0107.CN.15.zj032.KX159285

LPPLERLCI

>0107.CN.16.GX2016EU09.MF073269

LPPLERLCI

>0107.CN.16.GX2016EU10.MH377336

LPPLDRLSL

>0107.CN.16.TJIH0345.MH801989

LPPLERLCI

>0107.CN.x.305.MH684584

LPPLERLHL

>0107.CN.x.TJIH0069.MH682099

LPPIERLCI

>0107.TW.08.TN_H8.KT372798

LPPIERLHI

>0108.CN.12.GXDY_1299_NLFG.KF541292

LPPLERLHI

>0108.CN.14.12YN10551.KU356857

LPPLERLHI

>01A1A6.CY.06.CY178.FJ388953

LPPLERLHI

>01A1F2.CM.07.BS72.KR017779

LPPLERLHI

>01A1G.CD.76.Z321_Z321B.U76035

LPPIERLRI

>01A1G.CM.06.DEURF06CM001.KF716464

LPPLERLCL

>01ADF2.CM.01.01CM_0908MO.AY371170

LPPLERLNL

>01B.CN.07.07CNYN370.KF835546

LPPLERLHL

>01B.CN.11.JS2011001.KM111555

LPPLERLTL

>01B.CN.13.01B.CN.2012.11092.KU501256

LPPLERLHL

>01B.CN.13.BJMP3037B.KP418805

LPPLERLSL

>01B.CN.14.12YN10135.KT999999

LPSLERLTL

>01B.CN.15.AH150299.MH431788

LPPLERLTL

>01B.CN.15.SH150507.MH615836

LPPLERLTL

>01B.CY.06.CY179.FJ388954

LPPLDRLHL

>01B.JP.03.03JP_5091K279.AB859012

LPPLERLHL

>01B.JP.x.pHIV_1_Y271B01AE64.AB646691

LPPLERLTL

>01B.MM.00.mIDU502.AB097865

LPPLERLSL

>01B.MM.13.mSSDU187.KU820835

LPPLERLHL

>01B.MM.99.mCSW104.AB097867

LPPLERLHL

>01B.MY.05.05MYKL043_1.DQ366666

LPPLERLNL

>01B.MY.06.06MMYKLD46.EF495062

LPPLERLHL

>01B.MY.08.08MYKL056.KT438782

LPPLDRLTL

>01B.MY.10.10MYKJ086.KT438783

LPPLERLNL

>01B.MY.10.10MYPR226.KJ206289

LPPLERLTL

>01B.MY.10.10MYPR70.KT438784

LPPLERLTL

>01B.PH.15.1001.MH327744

LPPLERLHL

>01B.PH.15.DEURF15PH001.KY658692

LPPLERLHL

>01B.PH.15.DEURF15PH005.KY658691

LPPIERLHL

>01B.PH.16.1009.MH327751

LPPLERLHL

>01B.PH.16.DEURF16PH011.MH078564

LPPLERLHL

>01B.SE.10.SE600035.KP411826

LPPLERLTL

>01B.TH.02.OUR740I.AY358073

LPPLERLTL

>01B.TH.02.OUR840I.AY358070

LPPLERLNL

>01B.TH.02.OUR847I.AY358069

LPPLERLTL

>01B.TH.03.03TH700065.JN248316

LPPLERLHL

>01B.TH.04.04TH228466.JN248319

LPPIERLHL

>01B.TH.04.04TH312908.JN248320

LPPLERLTL

>01B.TH.04.04TH321566.JN248322

LPPLERLHL

>01B.TH.04.04TH322500.JN248323

LPPLERLTL

>01B.TH.04.04TH423323.JN248326

LPPLERLHL

>01B.TH.04.04TH704320.JN248331

LPPLERLTL

>01B.TH.04.04TH801743.JN248332

LPPLERLHL

>01B.TH.05.05TH140456.JN631793

LPPLERLTL

>01B.TH.05.05TH245651.JN248340

LPPLERLHI

>01B.TH.05.05TH443479.JN248349

LPPLERLTL

>01B.TH.05.05TH518944.JN248350

LPPLERLTL

>01B.TH.05.05TH852327.JN248357

LPPLERLSL

>01B.TH.05.AA095a_WG21.JX447830

LPPIERLHL

>01B.TH.05.AA106c05R.JX448016

LPPLERLHL

>01B.TH.06.AA020a_wg2.JX446927

LPPIEKLHL

>01B.TH.06.AA025a_WG13.JX447000

LPPLERLHL

>01B.TH.06.AA084a_WG10.JX447668

LPPLERLHL

>01B.TH.07.MERLBDTRC3.JN860762

LPPLERLTL

>01B.TH.07.MERLBDTRC6.JN860765

LPPLERLTL

>01B.TH.13.2544878P000_Sa.MF957578

LPPLERLTL

>01B.TH.99.OUR044I.AY358042

LPPLERLHL

>01B.TH.99.OUR2574.DQ354123

LPPLERLTL

>01B.TH.x.TH283.AF468970

LPPLERLTL

>01B.TH.x.TH_13_26.AY082968

LPPLEKLTL

>01B.US.98.99US_MSC5043.AY444812

LPPLERLHL

>01BC.CN.07.07CNYN330.KF835517

LPPLERLHL

>01BC.CN.07.07CNYN334.KF835520

LPPLERLHL

>01BC.CN.07.07CNYN358.KF835537

LPPLERLHL

>01BC.CN.08.BH095.KF803580

LPPLERLQL

>01BC.CN.10.DH02.KF250395

LPPIERLNI

>01BC.CN.10.DH07.KF250398

LPPIDRLNI

>01BC.CN.10.JL.RF05.KJ184177

LPPLERLHL

>01BC.CN.11.ANHUI_BB17.KC183774

LPPLERLHL

>01BC.CN.11.ANHUI_WH69.KC183781

LPPLERLHL

>01BC.CN.12.DH28.KF250407

LPPLERLHI

>01BC.CN.13.01BC.CN.2011.11312.KU501257

LPPLERLTL

>01BC.CN.13.S15_5h4_3h2_FL.KP170487

LPPLERLHL

>01BC.CN.13.SZ44LS7251.KX378999

LPPLERLHI

>01BC.CN.13.SZ95LS8027.KX379000

LPPLERLQL

>01BC.CN.14.BJ.2014.MSM.SP01.KP668994

LPPLDRLNI

>01BC.CN.14.XC2014EU01.KX353919

LPPLERLHL

>01BC.CN.15.AH150183.KY200513

LPPLERLNL

>01BC.CN.15.Guangzhou.MF379808

LPPLERLNL

>01BC.CN.15.JS150021.KY200514

LPPLERLNL

>01BC.CN.15.JS150029.KY200515

LPPLERLHL

>01BC.CN.15.ZJCIQ15005.KX010453

LPPLERLHL

>01BC.CN.16.GX2016EU13.MG519330

LPPLERLHI

>01BC.CN.16.GX2016EU23.MG064457

LPPLERLHI

>01BC.CN.x.JL16013.MH051842

LPPLERLHL

>01BC.MM.00.mCSW503.AB097866

LPPLERLHL

>01BC.MM.08.08mLDTD011.KP455640

LPPLEGLHL

>01BC.MM.13.mSSDU139.KU820827

LPPLERLNI

>01BC.MM.13.mSSDU153.KU820830

LPPLERLNL

>01BC.MM.13.mSSDU199.KU820838

LPPLERLHL

>01BC.MM.13.mSSDU247.KU820841

LPPLDRLHL

>01BC.MM.14.mKSDU24.KU820848

LPPLERLTL

>01BC.MM.99.mIDU107.AB097868

LPPLERLTL

>01BC.PH.17.1023.MH327758

LPPLERLNL

>01C.CN.08.08YN080.HM138656

LPPLERLHL

>01C.CN.10.DH18.KF250401

LPPLERLSL

>01C.CN.13.kang019a_NFL.KJ778895

LPPLERLHL

>01C.CN.14.12YN10159.KT321211

LPPLERLNI

>01C.CN.15.JS150129.KY200516

LPPLERLHL

>01C.CN.15.JS150132.KY200517

LPPLERLHI

>01C.IN.14.NARI_FLS_YCM_151.KT074935

LPPLERLHL

>01C.IN.98.NARI_FLS_VB5.KT175204

LPPLERLCL

>01C.MM.13.mSSDU101.KU820822

LPPLERLHL

>01C.TH.x.NP1809.AY262830

LPPLERLHL

>01F2G.CM.02.LT31.JN864056

LPPLERLHL

>0206.BF.x.LA13BF17.KU168268

LPPIERLRL

>0206.BJ.13.LA58Benin.KU168301

LPPIERLSL

>0206.GH.03.03GH195AG_06.AB286853

LPPIERLRL

>0206.NE.00.NE36.AJ508597

LPPIERLRL

>0206.NE.97.NE03.AJ508595

LPPIERLSL

>0206.SN.12.LA56Senegl.KU168299

LPPIDRLSL

>0209.CI.01.01IC_17395.AJ866554

LPPLERLCL

>0209.CI.01.01IC_PCI127.AJ866558

LPPIERLCL

>0209.CI.16.DEURF16CI004.MH078537

LPPIERLCL

>0209.CI.97.97IC_PCI3.AJ866555

LPPLERLCL

>0209.GH.97.G829.AF184155

LPPLERLNL

>0213.CM.01.01CM_0096MA.GU201497

LPPIERLCL

>0222.CM.02.02CAMLT04.EU743964

LPPLERLCL

>0222.CM.06.NYU488.JN864047

LPPLERLHL

>0222.CM.08.BDSH129.JN864052

LPPIERLCI

>0222.CM.10.LB045.JN864053

LPPIERLSL

>0225.CM.x.2931HA.DQ826727

LPPLERLNL

>0263.UZ.02.02UZ0683.AY829214

LPPIERLCL

>02A.CI.16.DEURF16CI011.MH078538

LPPLERLCL

>02A.CM.02.02CM_2339SA.AY371143

LPPIERLCL

>02A.CM.08.DEURF08CM005.KF716465

LPPLERLRL

>02A.ES.05.X230_10.FJ670515

LPPLERLNL

>02A.ES.14.ARP1205.KT276265

LPPIERLRL

>02A.ES.14.EUR_0048.KU685587

LPPIERLCL

>02A.GW.04.DL3039.KR067669

LPPIERLSL

>02A.GW.08.DL3773.KR067670

LPPIERLSL

>02A.GW.10.DL3234.KR067667

LPPLERLSL

>02A.GW.13.DL5308.KR067671

LPPLERLCL

>02A.GW.14.DEURF14GW011.MH078547

LPPLERLSL

>02A.GW.14.DEURF14GW012.MH078548

LPPIERLCL

>02A.GW.14.DEURF14GW016.MH078549

LPPIERLSL

>02A.GW.14.DEURF14GW019.MH078550

LPPLERLTL

>02A.NG.09.09NG_SC27.JN248587

LPPIERLSL

>02A.NG.10.10NG020382.KX389616

LPPIERLSL

>02A1.CM.03.CM1193_8.KU168304

LPPIERLSL

>02A1.CM.04.242.EU513188

LPPIERLSL

>02A1.CM.04.257.EU513185

LPPIERLSL

>02A1.CM.05.280.EU513183

LPPLERLCL

>02A1.CM.08.CM1031_19.KU168307

LPPIEKLSL

>02A1.EE.06.A48.KM606636

LPPIERLRL

>02A1.GH.97.97GH_AG2.AB052867

LPPLERLCL

>02A1.GW.06.DL4186.KR067668

LPPIERLCL

>02A1.PK.14.DEURF14PK006.KU749413

LPPIERLCL

>02A1.PK.14.PK003.KX232596

LPPLERLCL

>02A1.PK.14.PK008.KX232601

LPPLERLRL

>02A1.PK.14.PK012.KX232605

LPPLERLHL

>02A1.PK.14.PK015.KX232608

LPPLERLHL

>02A1.PK.14.PK019.KX232612

LPPLERLHL

>02A1.PK.15.DEURF15PK023.KY658718

LPPIERLCL

>02A1.PK.15.PK025.KX232617

LPPIERLCL

>02A1.PK.15.PK033.KX232623

LPPLERLHL

>02A1.PK.15.PK038.KX232627

LPPIERLCL

>02A1.PK.15.PK040.KX232629

LPPLERLHL

>02A1.RU.11.11RU6939.JX500706

LPPIERLCL

>02A1F2.CM.10.DEURF10CM024.KU749421

LPPLERLSL

>02A1G.CM.02.02CM_3205MN.GU201504

LPPLERLHL

>02A1G.CM.07.BS29.KR017773

LPPIERLRL

>02A3.CM.07.BS13.KR017772

LPPFERLRL

>02A3.GH.03.GHNJ176.AB231894

LPPIERLSL

>02A3.SN.96.DDJ362.AY521632

LPPIERLCL

>02A6.RU.08.DE00208RU002.KU749432

LPPLERLCL

>02A6.RU.11.11RU6900.JX500697

LPPLERLCL

>02A6.SE.10.039KZ.MF373150

LPPIERLCL

>02B.ES.13.ARP1194.KT276254

LPPIERLCI

>02B.ES.99.99SP_11339.DQ926899

LPPIERLSL

>02B.FR.02.URF4.JN882652

LPPLERLTL

>02B.FR.09.URF2.JN882654

LPPIERLTL

>02B.FR.09.URF3.JN882651

LPPLERLTL

>02B.GB.13.13774_1_86.3.MF109508

LPPLERLTL

>02B.GB.14.13612_1_14.3.MF109397

LPPLERLSL

>02BD.FR.08.URF1.JN882653

LPPIERLSL

>02BF1.GB.14.14727_1_76.3.MF109637

LPPLERLHI

>02BG.CY.07.CY200.JF683752

LPPIERLCL

>02C.BE.93.VI1035.AJ276595

LPPLEGLTL

>02D.CM.03.CM10_10.KU168303

LPPLERLNL

>02D.CY.08.CY217.JF683766

LPPVERLNL

>02D.GH.03.GHNJ193.AB231897

LPPLERLTL

>02D.GH.03.p03GH194AG09.AB480044

LPPIERLCL

>02F2.CM.07.BS11.KR017771

LPPLERLHI

>02F2G.CM.02.LT66.JN864057

LPPLERLHI

>02G.BE.94.VI1197.AJ276596

LPPLERLSL

>02G.CM.02.02CM_3228MN.AY371147

LPPIERLSL

>02G.GB.13.15228_1_70.3.MF109708

LPPLERLCL

>02G.GB.14.13612_1_1.3.MF109402

LPPLERLCI

>02G.GH.03.p03GH179AG_G02.AB480047

LPPLERLCL

>02G.NG.09.09NG010129.KX389634

LPPIERLSL

>02G.NG.09.09NG010145.KX389632

LPPIERLRL

>02G.NG.09.09NG010208.KX389627

LPPIERLSL

>02G.NG.09.09NG010483.KX389623

LPPIERLNL

>02G.NG.10.10NG020468.KX389613

LPPIERLSL

>02G.NG.92.92NG003.U88825

LPPIERLSL

>02GK.CI.01.01IC_PCI123.AJ866557

LPPIERLCL

>02H.AO.93.93AOHDC251.KU310618

LPPIERLTL

>02O.CM.07.YBF282.KX398187

LPPLEQLSI

>02O.FR.10.RBF222.KY359382

LPPIERLCL

>02O.FR.17.RBF243.KY995542

LPPIERLCL

>02U.CY.06.CY158.FJ388944

LPPLERLHL

>02U.NG.10.10NG020545.KX389611

LPPIENLHL

>06A1.BJ.x.B76.AJ293865

LPPIERLRL

>06G.CM.08.814_43.MH705140

LPPIERLSL

>07B.TW.04.TW_D60.DQ230842

LPPIERLHI

>11A1.CM.07.BS57.KR017778

LPPIERLHL

>13U.CM.04.258.EU513192

LPPLERLSL

>14F1.ES.15.100_120.KY557336

LPPLERLHI

>1819.CU.99.CU64.AY894995

LPPLERLHL

>22A1U.CM.01.01CM_1152NG.AY371163

LPPLERLHL

>22DU.CM.01.01CM_4008HAN.AY371168

LPPLERLTL

>26C.CD.02.02CD_LBTB032.FM877779

LPPLERLHI

>26C.CD.97.97CD_KFE267.FM877778

LPPLERLHL

>26C.CD.97.MBFE250.FM877783

LPPLERLNL

>26CU.CD.97.97CD_KMST91.FM877784

LPPLERLRL

>32A6.EE.06.41.KM606628

LPPIERLRL

>32A6.EE.06.A19.KM606633

LPPIERLRL

>32A6.EE.06.A49.KM606637

LPPIERLRL

>32G.EE.06.A45.KM606635

LPPIERLRL

>50B.GB.13.13592_1_12.3.MF109354

LPPLERLNL

>50B.GB.14.14535_1_2.4.MF109527

LPPLERLNL

>A1A2.KE.05.05KE398838V4.KT022402

LPPLERLHL

>A1A2.KE.05.05KE726081V5.KT022407

LPPLERLHL

>A1A2C.KE.04.04KE389016V3.KT022386

LPPIERLHL

>A1A2C.KE.07.DEURF07KE014.KU749429

LPPLERLHL

>A1A2CD.KE.06.DEURF06KE011.KU749427

LPPLERLNL

>A1A2D.KE.91.KNH1239.AY945739

LPPLERLHL

>A1B.CY.08.CY225.JF683774

LPPLEKLTL

>A1B.CY.09.CY257.JF683800

LPPLERLSL

>A1B.ES.09.X2733_2.KC113007

LPSLERLTL

>A1B.GB.13.13592_1_28.3.MF109371

LPPLERLTI

>A1B.US.10.DEURF10US008.KC473836

LPPLERLTL

>A1BG.RU.15.RU_KKV_2015.MF614611

LPPLERLHL

>A1BG.RU.15.RU_ZRS_2015.MF614615

LPPIERLHL

>A1C.CA.04.04CA7750.EU220698

LPPIERLNI

>A1C.IN.01.1579A.DQ083238

LPPLERLCL

>A1C.IN.06.NARI_FLS_IVC3_1.33.KT175203

LPPIERLNL

>A1C.IN.09.NARI_FLS_09_387.KC911635

LPPLERLHL

>A1C.IN.15.SC017.KY713232

LPPLERLHI

>A1C.IN.95.95IN21301.AF067156

LPPLERLHL

>A1C.IN.99.NARI_FLS_VB27_69.KT175211

LPPIERLNI

>A1C.KE.00.KISII5011.AF457061

LPPLERLHL

>A1C.KE.00.MSA4080.AF457087

LPPIERLHL

>A1C.KE.04.04KE663257V3.KT022391

LPPIERLHL

>A1C.KE.04.04KE780221V3.KT022393

LPPIERLHL

>A1C.KE.04.04KE870929V2.KT022395

LPPIERLHI

>A1C.KE.05.05KE757760V5.KT022408

LPPLERLHI

>A1C.KE.06.06KE161801V6.KT022413

LPPLERLHL

>A1C.KE.06.06KECst_004.FJ623489

LPPIERLHL

>A1C.KE.10.20225v14_06.KY580534

LPPIERLRL

>A1C.KE.10.DEURF10KE001.KF716469

LPPLEGLKL

>A1C.KE.10.DEURF10KE003.KF716471

LPPIERLHL

>A1C.KE.11.DEURF11KE020.KU749431

LPPLERLHL

>A1C.KE.99.KNH1097.AF457064

LPPIERLHL

>A1C.RW.06.DEURF06RW006.KU749428

LPPLERLNL

>A1C.RW.92.92RW009_06.U88823

LPPIERLTI

>A1C.SE.08.026KE.MF373141

LPPLERLTI

>A1C.SE.11.052SE.MF373155

LPPIERLHL

>A1C.SE.11.060SE.MF373163

LPPIERLTI

>A1C.SE.14.092SE.MF373194

LPPIERLHI

>A1C.SE.14.093ER.MF373195

LPPIERLNL

>A1C.SE.96.SE9488.AF071474

LPPLERLHL

>A1C.TZ.01.A306.AY253318

LPPIDRLHI

>A1C.TZ.01.A355.AY253315

LPPIERLNI

>A1C.TZ.01.A359.AY253319

LPPIKRLHI

>A1C.TZ.02.CO346.AY734552

LPPIERLHL

>A1C.TZ.02.CO3710.AY734553

LPPLERLHL

>A1C.TZ.02.CO3720.AY734562

LPPIERLHI

>A1C.TZ.02.CO6770.AY734554

LPPIERLHL

>A1C.TZ.02.CO6968.AY734555

LPPIERLHI

>A1C.TZ.03.CO0329V1.KX907342

LPPLERLHL

>A1C.TZ.03.CO0844V2.KX907354

LPPIERLHI

>A1C.TZ.03.CO3301V1.KX907369

LPPIERLHI

>A1C.TZ.03.CO3415V1.KX907373

LPPIERLHL

>A1C.TZ.03.CO3473V1.KX907377

LPPIERLHI

>A1C.TZ.03.CO3589V1.KX907379

LPPIERLHI

>A1C.TZ.04.CO0098V3.KX907340

LPPIERLNI

>A1C.TZ.04.CO3256V2.KX907367

LPPIERLNI

>A1C.TZ.04.CO6540V3.KX907409

LPPIERLHI

>A1C.TZ.04.CO6867V2.KX907427

LPPIERLSI

>A1C.TZ.05.CO6728V5.KX907418

LPPIERLHI

>A1C.TZ.06.CO3022V7.KX907360

LPPIERLHL

>A1C.TZ.06.CO3058V6.KX907362

LPPIERLNI

>A1C.TZ.06.CO3651V7.KX907381

LPPLERLHI

>A1C.TZ.06.CO3812V6.KX907386

LPPIERLHI

>A1C.TZ.06.CO6917V7.KX907429

LPPIERLNI

>A1C.TZ.08.DEURF08TZ009.KY658699

LPPIERLSL

>A1C.TZ.97.97TZ01.AF361871

LPPIERLHI

>A1C.TZ.97.97TZ06.AF361876

LPPIERLHI

>A1C.TZ.97.97TZ08.AF361878

LPPIERLHI

>A1C.TZ.97.97TZ09.AF361879

LPPIEGLHI

>A1C.UG.99.99UGK30889.AF484501

LPPIERLQL

>A1C.ZA.07.503_03011_29.KT183118

LPPIERLTL

>A1CD.KE.00.KER2017.AF457056

LPPLERLHL

>A1CD.KE.02.ML2000.EU110093

LPPXERLXL

>A1CD.KE.04.04KE888800V3.KT022397

LPPLERLNL

>A1CD.KE.04.04KE956490V3.KT022398

LPPLERLNL

>A1CD.KE.05.05KE215154V4.KT022400

LPPLERLHI

>A1CD.KE.05.05KE295198V5.KT022401

LPPLERLNL

>A1CD.KE.06.06KE484390V7.KT022414

LPPLERLTL

>A1CD.KE.06.06KE688699V6.KT022415

LPPIERLTL

>A1CD.KE.08.DEURF08KE018.KU749430

LPPIERLHL

>A1CD.KE.10.DEURF10KE002.KF716470

LPPLERLHL

>A1CD.SE.95.SE8603.AF075702

LPPLERLNL

>A1CD.TZ.03.CO0335V1.KX907344

LPPLERLNI

>A1CD.TZ.03.CO6095V1.KX907397

LPPLERLNI

>A1CD.TZ.04.CO0735V4.KX907350

LPPIERLHI

>A1CD.TZ.04.CO0778V2.KX907351

LPPFERLTI

>A1CD.TZ.04.CO0815V3.KX907353

LPPLERLNL

>A1CD.TZ.04.CO3103V3.KX907365

LPPLERLNL

>A1CD.TZ.04.CO3817V2.KX907387

LPPIERLHI

>A1CD.TZ.04.CO6041V3.KX907396

LPPLERLNL

>A1CD.TZ.04.CO6273V4.KX907404

LPPLERLNL

>A1CD.TZ.04.CO6763V2.KX907419

LPPIERLNL

>A1CD.TZ.05.CO3326V4.KX907370

LPPLERLNL

>A1CD.TZ.05.CO6172V5.KX907402

LPPIERLHI

>A1CD.UG.10.DEURF10UG007.KF716489

LPPIERLTL

>A1CDGKU.ZA.99.CM4_99ZACM4.AF411964

LPPIERLHI

>A1CG.KE.02.ML1979PCR.EU110096

LPPLERLNL

>A1D.CM.x.CMN05_5.KU168311

LPPLERLNL

>A1D.DK.96.FSA.DQ912822

LPPLERLTL

>A1D.GB.14.14592_1_70.3.MF109564

LPPLERLNL

>A1D.KE.00.KER2021.AF457058

LPPLERLNL

>A1D.KE.00.KSM4028.AF457078

LPPLERLHL

>A1D.KE.00.MSA4071.AF457082

LPPIERLHL

>A1D.KE.02.ML1974.EU110090

LPPLERLNL

>A1D.KE.03.03KE543869V2.KT022385

LPPLERLTI

>A1D.KE.04.04KE414659V2.KT022387

LPPFERLNL

>A1D.KE.04.04KE489686V2.KT022388

LPPLERLSL

>A1D.KE.04.04KE556528V2.KT022389

LPPLERLNL

>A1D.KE.04.04KE629642V3.KT022390

LPPLERLHI

>A1D.KE.04.04KE719832V2.KT022392

LPPLERLHL

>A1D.KE.04.04KE830543V3.KT022394

LPPLERLTL

>A1D.KE.04.04KE880505V2.KT022396

LPPLERLHI

>A1D.KE.05.05KE176519V4.KT022399

LPPLERLRL

>A1D.KE.05.05KE530245V4.KT022404

LPSLEGLHL

>A1D.KE.05.05KE613437V4.KT022405

LPPLERLTL

>A1D.KE.05.05KE897156V5.KT022409

LPPLERLNL

>A1D.KE.05.05KE961896V5.KT022410

LPPLERLNL

>A1D.KE.05.05KE963379V4.KT022411

LPPLERLNL

>A1D.KE.06.06KE149896V6.KT022412

LPPLERLNL

>A1D.KE.06.06KE809646V6.KT022416

LPSLERLNL

>A1D.KE.06.06KE894822V7.KT022417

LPPLERLNL

>A1D.KE.06.06KECst_010.FJ623491

LPPIERLHL

>A1D.KE.06.06KECst_015.FJ623490

LPPIERLHI

>A1D.KE.09.DEURF09KE002.KF716468

LPPIERLHL

>A1D.KE.09.DEURF09KE004.KP174771

LPPLERLNL

>A1D.KE.96.QA790_204I_ENV_C1.FJ866125

LPPLERLNL

>A1D.KE.99.KISII5003.AF457059

LPPLERLHL

>A1D.KE.99.KSM4015.AF457073

LPPLERLHL

>A1D.KE.x.MS208.FJ443124

LPPLERLNL

>A1D.SE.10.042SE.MF373153

LPPLERLTL

>A1D.SE.93.SE6954.AF075701

LPPLERLTL

>A1D.SE.94.SE7108.AF071473

LPPLERLNL

>A1D.TZ.01.A387.AY253316

LPPIERLTL

>A1D.TZ.03.CO3468V1.KX907376

LPLLERLSL

>A1D.TZ.04.CO0907V4.KX907355

LPPIERLNL

>A1D.TZ.04.CO3038V3.KX907361

LPPIERLHL

>A1D.TZ.08.DEURF08TZ006.KY658698

LPPLERLHL

>A1D.TZ.96.TZBFL0011.AF442569

LPPIERLTL

>A1D.TZ.96.TZBFL0025.AF442565

LPPLERLNL

>A1D.TZ.96.TZBFL0088.AF442570

LPPLERLHL

>A1D.TZ.97.TZBFL0086.AF442566

LPPLERLNL

>A1D.UG.00.PP1_F4_A1.HM027851

LPPLERLTL

>A1D.UG.00.PP4_F2_A4.HM027850

LPPLERLTL

>A1D.UG.03.PP1_F3_B1.HM027870

LPPLERLHI

>A1D.UG.05.PP1_M_B1.HM027828

LPPLERLTL

>A1D.UG.07.p191947.JX236674

LPPLERLTL

>A1D.UG.08.p191982.JX236675

LPPIERLNL

>A1D.UG.09.DEURF09UG002.KF859746

LPPLERLNL

>A1D.UG.09.DEURF09UG004.KF716481

LPPIERLTL

>A1D.UG.09.DEURF09UG005.KF859747

LPPLERLTL

>A1D.UG.09.DEURF09UG014.KP109491

LPPLERLTL

>A1D.UG.09.DEURF09UG015.KP109492

LPPLERLTL

>A1D.UG.10.DEURF10UG005.KF716487

LPPLERLTL

>A1D.UG.10.DEURF10UG008.KF716490

LPPIERLTL

>A1D.UG.10.DEURF10UG011.KF716482

LPPLERLTL

>A1D.UG.10.DEURF10UG012.KF716483

LPPLERLNI

>A1D.UG.10.DEURF10UG016.KP109493

LPPIERLHL

>A1D.UG.11.10463v09_10.KY580490

LPPIERLHL

>A1D.UG.11.DEURF11UG002.KP109497

LPPLEKLTL

>A1D.UG.11.DEURF11UG004.KF716484

LPPLERLTL

>A1D.UG.11.DEURF11UG006.KF716485

LPPLERLHL

>A1D.UG.90.UG266.AY352657

LPPLERLTL

>A1D.UG.92.UG035.AY352656

LPPIERLHL

>A1D.UG.98.98UG57129.AF484503

LPPLERLNI

>A1D.UG.98.98UG57137.AF484521

LPPIERLTL

>A1D.UG.99.99UGB26587.AF484482

LPPLERLHL

>A1D.UG.99.99UGC06443.AF484479

LPPLERLNL

>A1D.UG.99.99UGC38442.AF484517

LPPLERLTL

>A1D.UG.99.99UGE22831.AF484488

LPPLERLTL

>A1D.UG.99.99UGF27390.AF484492

LPPLERLTL

>A1D.UG.99.99UGK38855.AF484520

LPPLERLNL

>A1D.ZA.00.TV101.KJ948659

LPPLEGLHL

>A1DG.ES.09.DEURF09GQ001.KC473845

LPPLERLTL

>A1DHK.NO.97.97NOGIL3.AJ237565

LPPLERLTL

>A1F1.CY.07.CY191.JF683744

LPPLERLHI

>A1F2.CM.01.01CM_1404MV.AY371164

LPPLERLHL

>A1F2.CM.11.CHU3903.KP718932

LPPLERLRI

>A1G.CD.02.CG_0379_02V_NGSID7.KY392772

LPPLERLHL

>A1G.CM.03.CM62_15.KU168306

LPPLERLHL

>A1G.CM.04.252.EU513190

LPPIERLRL

>A1G.GB.14.14592_1_42.3.MF109538

LPPLERLTL

>A1G.GB.14.14727_1_68.6.MF109629

LPPIERLHI

>A1G.KE.99.KNH1043.AF457062

LPPLERLHL

>A1GH.CD.02.CG_0378_02V_NGSID6.KY392771

LPPIERLHI

>A1GHU.GA.x.VI354.AF076474

LPPLERLTL

>A1H.CM.01.01CM_4038STN.GU201508

LPPLERLRL

>A1H.CM.08.867_10.KP718927

LPPLERLTL

>A1H.CM.11.CHU2727.KP718933

LPPLERLHL

>A1K.CM.07.280_10.KP718919

LPPLERLHL

>A1O.FR.06.BCF212.KY359380

LPPLERLHL

>A1O.FR.13.BCF204.KY359385

LPPLEQLSI

>A1O.FR.13.RBF237.KY359381

LPPLERLHI

>A1U.BR.10.10BR_SP011.KJ849789

LPPLERLCL

>A1U.CY.05.CY111.FJ388928

LPPIERLHL

>A1U.SE.93.SE6594.AF069672

LPPLERLNL

>A2C.ZA.98.DU178_98ZADU178.AF411965

LPPLERLHL

>A2C.ZM.89.ZAM174.U86768

LPPIERMHL

>A2CD.KE.01.ML1956.EU110089

LPPLERLNL

>A2G.CD.97.97CDKP58.AF316544

LPPLERLHL

>AC.ZA.04.04ZAPS204B1.DQ093606

LPPIERLHL

>ACD.TZ.01.HS123.AY945709

LPPLERLNL

>ACU.CD.08.DRC768.MF372644

LPPLERLCI

>AD.US.99.99US_MSC4068.AY444799

LPPIERLRL

>ADGU.CU.99.CU67.AY894996

LPPLERLHL

>AF2.CM.02.02CM_3163MN.AY371160

LPPLERLHI

>AF2G.CM.97.CM52876.AF377958

LPPLERLHI

>AGU.CM.01.01CM_0989MO.AY371166

LPPIERLHL

>AGU.CM.06.770_8.MH705142

LPPLERLNL

>AHJU.CM.01.01CM_1296NG.AY371162

LPPIERLHI

>AJ.CD.87.PBS0724.MH705159

LPPLERLHL

>AKU.US.x.L873.DQ886038

LPPLERLSL

>AU.CD.87.PBS1342.MH705135

LPPLERLHL

>BC.BR.01.01_BR_RGS45.GQ365651

LPPIERLTI

>BC.BR.01.01_BR_RGS69.GQ365652

LPPIERLNI

>BC.BR.05.05_BR_NSP24.GQ365650

LPPIERLNI

>BC.BR.06.06BR1114.JN692478

LPPIERLNI

>BC.BR.07.DEURF07BR002.KC596068

LPPIERLDI

>BC.BR.10.10BR_PE018.KJ849795

LPPLERLTI

>BC.BR.10.10BR_PE057_2.KT427849

LPPLERLTL

>BC.BR.10.10BR_RJ009.KT427790

LPPIERLTI

>BC.BR.10.10BR_RJ039.KT427778

LPPIERLNI

>BC.BR.10.10BR_RJ091.KT427760

LPPIERLHI

>BC.BR.10.10BR_SP015.KT427822

LPPLERLTL

>BC.BR.92.92BR023.HM100716

LPPIERLNI

>BC.CN.05.05YNRL08sg.KC898976

LPSLERLHI

>BC.CN.05.05YNRL09sg.KC898977

LPPLERLHI

>BC.CN.05.05YNRL20sg.KC898978

LPPLERLTL

>BC.CN.05.BC11.EU363841

LPPLERLHI

>BC.CN.06.06YNRL106sg.KC898981

LPPLERLTL

>BC.CN.06.BC08.EU363838

LPPLERLHI

>BC.CN.07.07CNYN335.KF835521

LPPLERLTL

>BC.CN.07.07CNYN338.KF835524

LPPIERLHI

>BC.CN.07.07CNYN339.KF835525

LPPIERLTL

>BC.CN.07.07CNYN340.KF835526

LPPLERLNI

>BC.CN.07.07CNYN360.KF835539

LPPIERLHI

>BC.CN.07.07CNYN361.KF835540

LPPIERLHI

>BC.CN.07.07CNYN367.KF835544

LPPLERLHI

>BC.CN.07.07YNLC08sg.KC898982

LPPLERLTL

>BC.CN.07.07YNLC18sg.KC898983

LPPIERLHI

>BC.CN.07.07YNLC22sg.KC899007

LPPIERLTL

>BC.CN.07.BC04.EU363834

LPPIERLNI

>BC.CN.07.BC05.EU363835

LPPIERLHI

>BC.CN.07.cq070084.KF250381

LPPLERLHI

>BC.CN.07.gd070112.KF250382

LPPLERLSI

>BC.CN.07.gx070056.KF250383

LPPLERLHI

>BC.CN.07.jx070017.KF250384

LPPIERLHI

>BC.CN.08.08YN065.GU362013

LPPLERLTL

>BC.CN.09.09YNLC10sg.KC898984

LPPLERLTL

>BC.CN.09.09YNLC216027sg.KC898986

LPPIEGLHI

>BC.CN.09.09YNLC216031sg.KC898987

LPPIERLHI

>BC.CN.09.09YNLC216036sg.KC898988

LPPIEGLHI

>BC.CN.09.09YNLC492sg.KC898989

LPPIERLHI

>BC.CN.09.09YNLC499sg.KC898993

LPPLERLTL

>BC.CN.09.09YNRL215025sg.KC899010

LPPLERLTL

>BC.CN.09.09YNYJ217010sg.KC899013

LPPLERLTL

>BC.CN.09.09YNYJ217016sg.KC899014

LPPLERLHI

>BC.CN.09.09YNYJ217036sg.KC899003

LPPIERLHI

>BC.CN.09.09YNYJ479sg.KC899015

LPPLERLTL

>BC.CN.10.DH04.KF250397

LPPIERLHI

>BC.CN.10.DH15.KF250399

LPPLERLHI

>BC.CN.10.JL100091.KF011493

LPPIERLHI

>BC.CN.12.DH23.KF250405

LPPIERLHI

>BC.CN.12.DH24.KF250406

LPPLERLTL

>BC.CN.12.DH33.KF250409

LPPLERLTL

>BC.CN.12.DH36.KF250410

LPPIERLHI

>BC.CN.14.12YN10192.KT960983

LPPIEKLTL

>BC.CN.14.XC2014EU20.KU886698

LPPLERLTI

>BC.CN.14.YN10134.KY406739

LPPIERLHI

>BC.CN.15.SN153.MG742702

LPPLERLTL

>BC.CN.96.YN4007.KF250379

LPPLERLTL

>BC.CN.98.YNRL9828.AY967805

LPPLERLTL

>BC.DO.05.05DO_147691.EU839599

LPPIERLTI

>BC.IN.02.INDNARI_0218440.EU000514

LPPLERLTL

>BC.IN.02.NARI7_3.EU000511

LPPLERLIL

>BC.IN.02.NARI9_3.EU000508

LPPLERLTL

>BC.IN.07.MAN_86.HQ453404

LPPIERLNI

>BC.IN.07.NARI_L5_NEM.J12.FJ515876

LPPLERLNL

>BC.IN.08.Man_40.HM573466

LPPLERLTL

>BC.IN.99.NARI10_2.EU000516

LPPLERLTL

>BC.MM.14.fKSDU97.KU820852

LPPIERLHI

>BC.MM.14.mKSDU81.KU820850

LPPLERLTL

>BC.MM.14.mKSDU92.KU820851

LPQIERLHI

>BC.SE.13.087SE.MF373189

LPPMERLNL

>BC.UY.02.02UY_TRA3026.JN235957

LPPIERLNI

>BC.ZA.09.503_01288_T4.KT183077

LPPIERLHI

>BCF1.AR.04.04AR160677.DQ383754

LPPIERLTL

>BCF1.BR.10.10BR_MG010.KJ849787

LPPLERLSL

>BCF1.BR.10.10BR_PE029.KJ849800

LPPLERLTI

>BCF1.BR.10.10BR_RJ001.KT427793

LPPLERLTL

>BCG.ES.15.100_119.KY496623

LPPIERLSL

>BD.ZA.85.R605.MH234642

LPPLERLTL

>BF1.AR.00.ARCH003.AY037267

LPPLERLTL

>BF1.AR.02.02AR115455.DQ383747

LPPLERLTL

>BF1.AR.04.04AR143170.DQ383750

LPPLDRLTL

>BF1.AR.04.04AR158637.DQ383753

LPPLERLTL

>BF1.AR.05.05AR163052.DQ383755

LPPLERLTL

>BF1.AR.12.DEURF12AR009.KY658687

LPPLERLTL

>BF1.AR.14.DEURF14AR011.MH078535

LPPLERLTL

>BF1.AR.97.A050.AF408631

LPPLERLTL

>BF1.AR.97.A32878.AF408632

LPPLERLTL

>BF1.AR.98.ARCH014.AY037266

LPPLERLTL

>BF1.AR.99.A025.AF408626

LPPLERLTL

>BF1.AR.99.A027.AF332867

LPPLERLTL

>BF1.AR.99.A047.AF408627

LPPLERLTL

>BF1.AR.99.A063.AF408628

LPPLERLTL

>BF1.AR.99.ARMA006.AY037278

LPPLERLSL

>BF1.AR.99.ARMA029.AY037283

LPPLERLTL

>BF1.AR.99.ARMA037.AY037277

LPPLEKLSL

>BF1.AR.99.ARMA097.AY037280

LPPLERLTL

>BF1.BR.00.BREPM13853.DQ085875

LPPLERLTL

>BF1.BR.01.01BR042.DQ358799

LPQLERLTL

>BF1.BR.01.01BR047.DQ358800

LPPIERLTL

>BF1.BR.01.01BR226.DQ358803

LPPLERLTL

>BF1.BR.01.01BR323.DQ358804

LPPLERLHI

>BF1.BR.02.02BR005.DQ358806

LPPLERLTL

>BF1.BR.02.02BR006.DQ358807

LPPLERLTL

>BF1.BR.02.02BR034.DQ358812

LPPLERLHI

>BF1.BR.02.02BR2028.JN692437

LPSLERLTI

>BF1.BR.02.02BRRJSB167.MG365770

LPPLERLHI

>BF1.BR.03.03BR2018.JN692449

LPPLERLTP

>BF1.BR.03.03BR2019.JN692448

LPPLDRLTL

>BF1.BR.03.BREPM1026.EF637055

LPPLERLTL

>BF1.BR.03.BREPM1029.EF637052

LPPLERLSL

>BF1.BR.04.04BR1067.JN692456

LPPLERLTL

>BF1.BR.05.0008SP.JF804805

LPPLERLTL

>BF1.BR.05.0341RI.JF804808

LPPLERLTL

>BF1.BR.05.0632SV.JF804810

LPPIGGLTL

>BF1.BR.05.0736SV.JF804813

LPPLEKLTL

>BF1.BR.06.06BR_FPS561.HM026455

LPPLERLNI

>BF1.BR.07.BP00044_RH02.JN687738

LPPLERLTL

>BF1.BR.09.09BRRJRDS205.MG365769

LPPLERLHI

>BF1.BR.10.10BR_MG017_2.KJ849793

LPPLEKLTL

>BF1.BR.10.10BR_MG030.KT427679

LPPLERLTI

>BF1.BR.10.10BR_MG034.KJ849802

LPPLERLTL

>BF1.BR.10.10BR_MG052.KT427668

LPPLERLTL

>BF1.BR.10.10BR_PE002.KJ849757

LPPLERLTL

>BF1.BR.10.10BR_PE005.KT427746

LPPLERLNL

>BF1.BR.10.10BR_PE043.KT427722

LPPLERLTL

>BF1.BR.10.10BR_PE059.KJ849768

LPPLERLTL

>BF1.BR.10.10BR_PE063.KT427871

LPPLERLTL

>BF1.BR.10.10BR_PE069.KT427710

LPPLERLHI

>BF1.BR.10.10BR_PE078.KT427705

LPPLERLHI

>BF1.BR.10.10BR_PE086.KJ849774

LPPLERLTL

>BF1.BR.10.10BR_PE099.KT427698

LPPLERLTL

>BF1.BR.10.10BR_RJ012.KT427649

LPPLERLTL

>BF1.BR.10.10BR_RJ014.KT427789

LPPIDRLTL

>BF1.BR.10.10BR_RJ029.KT427782

LPPIERLTL

>BF1.BR.10.10BR_RJ031.KT427780

LPPIERLTL

>BF1.BR.10.10BR_RJ036.KT427779

LPPLERLHI

>BF1.BR.10.10BR_RJ041.KJ849826

LPPLERLTL

>BF1.BR.10.10BR_RJ044.KT427776

LPPLERLHI

>BF1.BR.10.10BR_RJ053.KJ849813

LPPLERLTL

>BF1.BR.10.10BR_RJ075.KT427651

LPPLERLTL

>BF1.BR.10.10BR_RJ093.KT427759

LPPLERLHI

>BF1.BR.10.10BR_RJ094.KT427758

LPPXERLTL

>BF1.BR.10.10BR_RJ104.KT427652

LPPLERLTL

>BF1.BR.10.10BR_SP005.KT427828

LPPIERLTI

>BF1.BR.10.10BR_SP010.KT427825

LPPLERLTL

>BF1.BR.10.10BR_SP013.KT427823

LPPLERLHI

>BF1.BR.10.10BR_SP017.KJ849794

LPPLDRLTL

>BF1.BR.10.10BR_SP018.KT427820

LPSLERLTL

>BF1.BR.10.10BR_SP024_2.KT427660

LPPLERLTL

>BF1.BR.10.10BR_SP035.KT427809

LPPLERLTL

>BF1.BR.10.10BR_SP037.KT427807

LPPLERLTL

>BF1.BR.10.10BR_SP048.KJ849810

LPPLERLTL

>BF1.BR.10.10BR_SP054.KT427801

LPPLERLTL

>BF1.BR.10.10BR_SP057.KJ849816

LPPLERLHI

>BF1.BR.10.10BR_SP068.KT427795

LPPLERLTL

>BF1.BR.16.HI2016_11.MG571986

LPPIERLTL

>BF1.BR.16.HI2016_32.MG572011

LPPIERLTL

>BF1.BR.93.93BR029_4.AF005495

LPPLERLHI

>BF1.BR.94.94BR_RJ_41.AY455781

LPPLEKLHI

>BF1.BR.94.94BR_RJ_59.AY455784

LPPLERLHI

>BF1.BR.94.94UFRJ_58.AY455785

LPPLERLHI

>BF1.BR.99.99BRRJSB067.MG365765

LPPLEKLHI

>BF1.BR.99.99UFRJ_16.AY455782

LPPLEKLHI

>BF1.BR.99.99UFRJ_2.AY455780

LPPLERLTI

>BF1.BR.99.99UFRJ_9.AY455783

LPPLERLHI

>BF1.BR.99.BREPM108.AY771589

LPPLERLTL

>BF1.BR.99.BREPM11871.DQ085867

LPPLERLTL

>BF1.BR.99.BREPM11884.DQ085868

LPPLERLTL

>BF1.BR.99.BREPM11932.DQ085870

LPPLERLTL

>BF1.BR.99.BREPM269.AY771591

LPPLERLHI

>BF1.BR.99.BREPM275.AY771592

LPPLERLTL

>BF1.BR.99.BREPM278.AY771593

LPPLERLTL

>BF1.CL.01.CH3.AY536233

LPPLERLTL

>BF1.CL.01.CH6.AY536234

LPPLERLTL

>BF1.ES.08.DEURF08ES008.KU749408

LPPLERLTL

>BF1.ES.08.ES_X2524_2.GQ372989

LPPIERLSL

>BF1.ES.08.X2432_2.FJ853621

LPPLERLHI

>BF1.ES.09.DEMBF09ES003.JX140660

LPPLERLTL

>BF1.ES.09.DEMBF09ES006.JX140661

LPPIERLSL

>BF1.ES.14.ARP1199.KT276259

LPPLDRLTL

>BF1.ES.14.ARP1209.KT276269

LPPLDRLTL

>BF1.ES.14.ARP1212.KT276271

LPPIERLTL

>BF1.IT.01.50610.GU595156

LPPLERLTL

>BF1.IT.01.53143.GU595149

LPPLERLTL

>BF1.IT.02.30638.GU595148

LPPLERLTL

>BF1.IT.02.57954.GU595150

LPPLERLTL

>BF1.IT.02.59211.GU595151

LPPLERLTL

>BF1.IT.05.83166.GU595152

LPPLERLTL

>BF1.IT.06.89072.GU595153

LPPLESLNL

>BF1.IT.08.IT_BF_PRIN_454.FJ904244

LPPLERLTL

>BF1.JP.04.DR6082.AB480298

LPPLERLHI

>BF1.JP.04.DR6190.AB480300

LPPLERLTI

>BF1.JP.x.DR0769.AB253430

LPPLERLTL

>BF1.PY.02.02PY_PSP0060.JN251897

LPPLERLTL

>BF1.PY.02.02PY_PSP0067.JN251898

LPPIDRLTL

>BF1.PY.02.02PY_PSP0070.JN251899

LPPLERLTL

>BF1.PY.02.02PY_PSP0087.JN251900

LPPXERLSL

>BF1.PY.02.02PY_PSP0093.JN251902

LPPLERLTL

>BF1.PY.02.02PY_PSP0094.JN251903

LPPLERLTL

>BF1.PY.03.03PY_PSP0114.JN251905

LPPLERLTL

>BF1.SE.15.103MK.MF373205

LPPLERLCI

>BF1.UY.00.00UY_TSU0020.JN235952

LPPLERLTL

>BF1.UY.01.01UYTRA1020.AY781128

LPPLERLTL

>BF1.UY.01.01UY_TRA1141.JN235953

LPPLERLTL

>BF1.UY.01.01UY_TRA2083.JN235956

LPPLERLTL

>BF1.UY.99.99UY_TRA0107.JN235960

LPPLERLHI

>BF1.UY.99.99UY_TRA0129.JN235963

LPPLERLHI

>BF1.UY.99.URTR17_URTR017.AY037272

LPPLERLTL

>BF1.VE.99.V62.AY536236

LPPLERLTL

>BG.RU.14.RU_KNCh_2014.MF614609

LPPLERLHL

>BG.RU.14.RU_VDL_2014.MF614608

LPPLERLHL

>BG.RU.15.RU_KAE_2015.MF614612

LPPLERLHL

>BG.RU.16.RU_ChTS_2016.MF614614

LPPLERLHL

>CD.BW.00.00BWMO35.1.AY074891

LPPIERLHI

>CD.KE.00.MSA4077.AF457085

LPPLERLNL

>CD.KE.01.ML1076.EU110086

LPPLERLTL

>CD.MW.08.CH492.PL.031108.A2.7.KY112292

LPPIERLHI

>CD.NP.11.11NP079.KU341727

LPPIERLHI

>CD.RW.11.DEURF11RW001.KF716473

LPPIERLTI

>CD.SE.08.029SE.MF373143

LPPIERLHI

>CD.SE.13.081ER.MF373183

LPPLERLNL

>CD.TZ.01.A247.AY253309

LPPIERLNI

>CD.TZ.04.CO3337V2.KX907371

LPPLERLTL

>CD.TZ.04.CO6480V4.KX907408

LPPIERLTI

>CD.TZ.05.CO6811V5.KX907421

LPPLERLTL

>CD.TZ.06.CO3016V6.KX907359

LPPIERLTL

>CD.TZ.97.97TZ07.AF361877

LPPLERLNI

>CD.UG.99.99UGK10192.AF484500

LPPLERLNI

>CD.ZA.13.DEURF13ZA135.KU749418

LPPLERLHL

>CF1.BR.07.DEURF07BR043.KU749397

LPPIERLTI

>CF1.BR.10.10BR_SP023.KT427818

LPPIERLNI

>CF1.ES.15.100348.MF381270

LPPLERLHI

>CF1.SE.13.086SE.MF373188

LPPIERLDI

>CU.CD.02.CG_0373_02V_NGSID2.KY392768

LPPLERLHI

>CU.CD.12.VIR90.MF372650

LPPIERLCI

>CU.JP.04.DR5782.AB286849

LPPLERLTL

>DF1G.ES.04.X963_4.FJ670527

LPPIERLTL

>DG.CM.06.876_14.KP718916

LPPIERLNL

>DG.CY.05.CY063.FJ388908

LPPIERLTL

>DG.GB.13.15228_1_62.3.MF109700

LPPLERLHI

>DG.KE.06.06KECst_014.FJ623495

LPPLERLSL

>DJ.KR.04.04KBH8.DQ054367

LPPIERLTI

>DO.CM.08.YBF274.KX579838

LPPLERLNL

>DO.FR.08.RBF208.GQ351296

LPPLEQLXI

>F1F2.CD.02.CG_0349_02_NGSID5.KY392770

LPPLERLHI

>F1F2.RU.08.D88_845.GQ290462

LPPLERLHI

>F2KU.BE.94.VI1126.AF076475

LPPLERLNL

>GK.NG.09.09NG010067.KX389638

LPPLERLHL

>GKU.SE.95.SE9010.AY352655

LPPIERLRL

>JU.AO.93.93AOHDC250.KU310619

LPPLERLHL

>UO.FR.16.RBF240.KY359384

LPPIERLHI

>U.CA.01.TV749.HM215251

LPPLERLSL

>U.CA.99.TV721.HM215249

LPPLERLSL

>U.CD.01.CG_0066_01_NGSID18.KY392780

LPPLERLHL

>U.CD.02.CG_0059_01_NGSID8.KY392773

LPPLERLTL

>U.CD.02.CG_0575_02_NGSID12.KY392775

LPPLERLNL

>U.CD.83.83CD003_Z3.AF286236

LPPLERLNL

>U.CD.90.90CD121E12.AF457101

LPPLERLHL

>U.CD.x.BCF_Kita.AB485665

LPPIERLCL

>U.CM.07.469_66.KP718920

LPPLERLTL

>U.CY.05.CY090.FJ388921

LPPLENLSL

>U.CY.08.CY223.JF683772

LPPIERLHI

>U.ES.10.DEURF10DZ001.JX140679

LPPIEKLSL

>U.GR.99.99GR303.AY046058

LPSIERLHL

>U.NG.11.DEURF11NG005.MH078558

LPPIERLRL

>U.NG.11.DEURF11NG011.MH078559

LPPLERLHL

>U.NL.01.U_NL_01_H10986_C11.EF029069

LPPLERLTL

## HIV-1 N Rev NES alignment

>N.CM.02.DJO0131.AY532635

LPPLDRLTL

>N.CM.04.04CM_1015_04.DQ017382

LPPLERLTL

>N.CM.04.04CM_1131_03.DQ017383

LPPLXRLTL

>N.CM.06.U14296.GQ324962

LPPLDRLTL

>N.CM.06.U14842.GQ324958

LPPLDRLTL

>N.CM.14.14003718.MF767262

LPPLDRLTL

>N.CM.15.S4858.KY498771

LPPIDRLTL

>N.CM.95.YBF30.AJ006022

LPPLDRLTL

>N.CM.97.YBF106.AJ271370

LPPLGRLTL

## SIVcpzPtt Rev NES alignment

>CPZ.CM.01.SIVcpzCAM13.AY169968

LPELDKLSL

>CPZ.CM.05.LB715.KP861923

LPPLERLSL

>CPZ.CM.05.SIVcpzDP943.EF535993

LPPIDRLTL

>CPZ.CM.05.SIVcpzEK505.DQ373065

LPPLDRLSL

>CPZ.CM.05.SIVcpzLB7.DQ373064

LPPIDRLSL

>CPZ.CM.05.SIVcpzMB66.DQ373063

LPPIERLTL

>CPZ.CM.05.SIVcpzMB897.EF535994

LPPLERLTL

>CPZ.CM.05.SIVcpzMT145.DQ373066

LPPIERLCI

>CPZ.CM.98.CAM3.AF115393

LPPIERLTI

>CPZ.CM.98.CAM5.AJ271369

LPPIDRLTL

>CPZ.GA.88.GAB1.X52154

LPELDKLSL

>CPZ.GA.88.SIVcpzGAB2.AF382828

LPPLERLSL

## HIV-1 P Rev NES alignment

>GQ328744

LPDLSRLHI

>GU111555

LPDLSRLHI

>HQ179987

LPDISQLHI

>JA446000

LPDISQLHI

>KY953207

LPDLSRLHI

## HIV-1 O Rev NES alignment

>O.BE.87.ANT70.L20587

LPPLEQLSI

>O.CM.03.1225_51.MH705149

LPPLEQLSI

>O.CM.03.296.MH705148

LPALEQLNI

>O.CM.06.20_02.MH705150

LPPLEQLSI

>O.CM.91.MVP5180.L20571

LPPLEQLNI

>O.CM.94.BCF06.AB485666

LPPLEQLSI

>O.CM.94.LA50YBF16.KU168293

LPDLEQLNI

>O.CM.95.LA29YBF26.KU168281

LPPLEQLSI

>O.CM.95.LA33YBF37.KU168285

LPPLEQLSI

>O.CM.96.96CMA102.AY169803

LPPLEQLSI

>O.CM.96.96CMABB009.AY169806

LPPLEQLNI

>O.CM.96.96CMABB637.AY169810

LPPLEQLSI

>O.CM.96.LA51YBF35.KU168294

LPPLEQLSL

>O.CM.96.LA52YBF39.KU168295

LPPLEQLSI

>O.CM.97.97CMABB447.AY169813

LPSLDQLSI

>O.CM.97.97CMABB497.AY169809

LPPLEQLSI

>O.CM.98.98CMA104.AY169802

LPPLEQLSI

>O.CM.98.98CMA105.AY169816

LPPLEQLSI

>O.CM.98.98CMABB141.AY169807

LPPLEQLSI

>O.CM.98.98CMABB197.AY169811

LPPLERLSI

>O.CM.98.98CMABB212.AY169804

LPPLEQLSI

>O.CM.98.98CMU2901.AY169812

LPPLEQLNI

>O.CM.98.98CMU5337.AY169808

LPPLEQLSI

>O.CM.99.99CMU4122.AY169815

LPPLEQLSI

>O.CM.99.AB260.MH705147

LPPLEQLSI

>O.CM.99.DSC1320.AY489739

LPPLEQLSI

>O.CM.x.pCMO2_3.AY618998

LPPIEQLSI

>O.DE.x.DEOXXDE004.KF859742

LPPLEQLNI

>O.ES.01.Read25_HIV_GroupO.KX228804

LPPLEQLSI

>O.FR.01.LA46BCF03.KU168289

LPPLEQLSI

>O.FR.02.LA34RBF130.KU168286

LPPLEQLSI

>O.FR.03.LA31BCF108.KU168283

LPPLEQLSI

>O.FR.03.LA32RBF140.KU168284

LPPLEQLSI

>O.FR.05.LA49RBF189.KU168292

LPPLEQLNI

>O.FR.06.RBF206.KY112585

LPSLEHLSI

>O.FR.92.VAU.AF407418

LPPLEQLSL

>O.FR.94.LA45BCF02.KU168288

LPPLEQLSI

>O.FR.95.LA47BCF09.KU168290

LPPLEQLSI

>O.FR.95.LA48BCF13.KU168291

LPPLEQLSI

>O.FR.98.LA30RBF125.KU168282

LPPLEHLSI

>O.FR.98.LA53BCF109.KU168296

LPPLEQLSI

>O.FR.99.LA54BCF120.KU168297

LPPLEQLSI

>O.GA.10.10Gab1190.JX245014

LPPLEQLSI

>O.GA.11.11Gab6352.JX245015

LPSLEHLSI

>O.SN.99.99SE_MP1299.AJ302646

LPPLEQLSI

>O.SN.99.99SE_MP1300.AJ302647

LPPLEQLSI

>O.US.10.LTNP.JN571034

LPPLEQLSI

>O.US.x.DEOXXUS001.KF859744

LPPLEQLSI

## SIVgor Rev NES alignment

>GOR.CM.04.SIVgorCP684con.FJ424871

LPDISHLTI

>GOR.CM.07.SIVgor2139_287.FJ424866

LPDISHLTI

>GOR.CM.07.SIVgorCP2135con.FJ424863

LPDISHLTI

>GOR.CM.12.SIVgor_BPID1.KP004989

LPDLGQLHI

>GOR.CM.12.SIVgor_BQID2.KP004991

LPSLEQLSI

>GOR.CM.13.SIVgor_BPID15.KP004990

LPDISHLHI

## SIVcpzpts Rev NES alignment

>CPZ.CD.06.BF1167.JQ866001

LPDLSKLTL

>CPZ.CD.90.ANT.U42720

LPDLQNLSL

>CPZ.TZ.00.TAN1.AF447763

IPDLSKLHL

>CPZ.TZ.01.TAN2.EF394357

LPDLSKLHL

>CPZ.TZ.02.TAN3_1.DQ374658

LPDLSKLHL

>CPZ.TZ.06.SIVcpzTAN13.JQ768416

LPDLSQLRL

>CPZ.TZ.06.TAN5.JN091691

IPDLSKLHL

>CPZ.TZ.09.UG38.JN091690

LPDLSKLRL

>CPZ.US.85.US_Marilyn.AF103818

LPPIGGLTI

## SIVmac Rev NES alignment

>AAW32420 rev protein [Simian immunodeficiency virus]

IQQLQNLAI

>AAV65328 rev protein [Simian immunodeficiency virus]

IQQLQNLAI

>AAV65319 rev protein [Simian immunodeficiency virus]

XQQLQNLAI

>AAU94545 rev protein [Simian immunodeficiency virus]

IQQLQBLAI

>AAU94537 rev protein [Simian immunodeficiency virus]

IQQLQNLAI

>AAU94528 rev protein [Simian immunodeficiency virus]

IQQLQNLAI

>AAU94519 rev protein [Simian immunodeficiency virus]

IQQLQNLAI

>AAU94510 rev protein [Simian immunodeficiency virus]

IQQLQNLAI

>AAU94501 rev protein [Simian immunodeficiency virus]

IQQLQNLAI

>AAU94492 rev protein [Simian immunodeficiency virus]

IQQLQNLAI

>AAU94483 rev protein [Simian immunodeficiency virus]

IQQLQNLAI

>AAU94474 rev protein [Simian immunodeficiency virus]

VQQLQNLAI

>AAU94464 rev protein [Simian immunodeficiency virus]

IQQLQNLAI

>AAU14058 rev protein [Simian immunodeficiency virus]

IQQLQNLAI

>AAU14049 rev protein [Simian immunodeficiency virus]

IQQLQNLAI

>AAU14040 rev protein [Simian immunodeficiency virus]

IQQLQNLAI

>AAU14031 rev protein [Simian immunodeficiency virus]

IQQLQNLAI

>AAU08051 rev protein [Simian immunodeficiency virus]

VQQLQNLAI

>AAU08046 rev protein [Simian immunodeficiency virus]

IQQLQNLAI

>AAU08036 rev protein [Simian immunodeficiency virus]

IQQLQNLAI

>AAU08028 rev protein [Simian immunodeficiency virus]

IQQLQNLAI

>AAT99867 rev protein [Simian immunodeficiency virus]

IQQLQNLAI

>AAT99454 rev protein [Simian immunodeficiency virus]

IQQLQNLAI

>AAT97127 rev protein [Simian immunodeficiency virus]

IQQLQNLAI

>AAT72789 rev protein [Simian immunodeficiency virus]

IQQLQNLAI

>AAT72775 rev protein [Simian immunodeficiency virus]

IQQLQNLAI

## HIV-2 Rev NES alignment

>A.CI.88.UC2.U38293

IQHLQGLTI

>A.DE.x.BEN.M30502

IQHLQRLTI

>A.DE.x.PEI2_KR_KRCG.U22047

IQRLQGLTI

>A.FR.00.LA38.KY025539

IQHLQGLTI

>A.FR.01.LA42.KY025543

IQHLQGLSI

>A.FR.02.LA36GomM.KU168287

IQQLQGLTI

>A.FR.93.LA37.KY025538

IQHLQGLTI

>A.FR.96.LA40.KY025541

IQHLQGLTI

>A.FR.98.LA39.KY025540

VRHLQELTI

>A.FR.98.LA41.KY025542

IQHLQELTI

>A.GH.x.GH1.M30895

IQDLQRLTI

>A.GM.87.D194.J04542

IQQLQGLTI

>A.GM.88.CBL20.MH681607

IQHLQGLTI

>A.GM.x.ISY_SBL_6669_85.J04498

IQQLQGLTI

>A.GM.x.MCN13.AY509259

IQHLQELTI

>A.GW.10.TD024.MH681608

VQQLQGLTI

>A.GW.10.TD031.MH681610

VQQLQELTI

>A.GW.10.TD062.MH681609

IQGLQGLTI

>A.GW.86.FG_clone_NIHZ.J03654

IQHLQGLTI

>A.GW.87.CAM2CG.D00835

IQHLQELTI

>A.GW.x.MDS.Z48731

VQQLQGLTI

>A.IN.07.NNVA.EU980602

IQQLQGLTI

>A.IN.95.CRIK_147.DQ307022

IQQLQGLTI

>A.JP.08.NMC786_clone_41.AB731742

IRDLQELTI

>A.NL.01.RH2.13.MF595856

IQHLQGLTI

>A.NL.01.RH2.14.MF595858

VQQLQGLTI

>A.NL.01.RH2.3.MF595854

IQHLQGLTI

>A.NL.01.RH2.7.MF595862

IQHLQGLTI

>A.NL.02.RH2.5.MF595860

IQHLQELTI

>A.NL.03.RH2.21.MF595864

IRNLQELTI

>A.NL.03.RH2.24.MF595866

IRNLQELTI

>A.PT.x.ALI.AF082339

VQHLQGLTI

>A.SN.85.RODR.MH681611

IQHLQGLTI

>A.SN.85.ROD.M15390

IQHLQGLTI

>A.SN.86.ST_JSP4_27.M31113

IQHLQGLTI

>A.x.x.MCN13.MA255545

IQHLQELTI

>B.CI.88.UC1.L07625

VQRLQELTV

>B.CI.x.20_56.AB485670

VQRLQELTI

>B.CI.x.EHO.U27200

IQRLQNLII

>B.FR.00.LA44.KY025545

VRRLQELTV

>B.FR.98.LA43.KY025544

IQQLQNLTV

>B.GH.86.D205_ALT.X61240

IQRLQNLTV

>B.JP.01.IMCJ_KR020_1.AB100245

IQQLQKLTV

>F.US.08.NWK08.KP890355

VQQLQRLNI

>G.CI.92.Abt96.AF208027

IQQLQSLTI

>H2_01_AB.CI.90.7312A.L36874

IRRLQNLTV

>H2_01_AB.JP.04.NMC307_20.AB731738

VRRLQNLAI

>H2_01_AB.JP.07.NMC716_01.AB731740

IRRLQNLAI

>H2_01_AB.JP.08.NMC842_10.AB731744

IRRLQNLAI

## SIVsmm Rev NES alignment

>SMM.CI.79.SIVsmCI2.JX860430

VQQLQGLSI

>SMM.LR.89.SIVsmLIB1.JX860431

VQQLQSLAI

>SMM.SL.92.SIVsmSL92A.JX860432

IQQLQSLTI

>SMM.SL.92.SL92B.AF334679

IQQFDSLSI

>SMM.US.04.G078.JX860415

VQQLQSLAI

>SMM.US.04.G932.JX860416

IRQLQNLAI

>SMM.US.04.M919.JX860417

IQQLQDLSL

>SMM.US.04.M922.JX860418

IQQLQDLSL

>SMM.US.04.M923.JX860419

IQQLQGLAI

>SMM.US.04.M926.JX860420

VQQLQSLSL

>SMM.US.04.M934.JX860421

VQQLQDLSL

>SMM.US.04.M935.JX860422

IQQLQDLSL

>SMM.US.04.M940.JX860423

VQQLQSLAI

>SMM.US.04.M946.JX860424

VQQLQDLSL

>SMM.US.04.M947.JX860425

VQQLQRLAI

>SMM.US.04.M949.JX860426

VQQLQSLAI

>SMM.US.04.M950.JX860427

IQQLQDLSL

>SMM.US.04.M951.JX860428

IQQLQNLAI

>SMM.US.04.M952.JX860429

IQQLQDLSL

>SMM.US.05.D215.JX860413

IQQLQSLAI

>SMM.US.06.FTq.JX860414

VRQLQNLAI

>SMM.US.11.SIVsmE660_FL10.JQ864084

IQQLQGLAI

>SMM.US.11.SIVsmE660_FL14.JQ864087

IQQLQGLAI

>SMM.US.11.SIVsmE660_FL6.JQ864085

IQQLQGLAI

>SMM.US.11.SIVsmE660_FL8.JQ864086

IQQLQGLAI

>SMM.US.86.CFU212.JX860407

VQQLQNLAI

>SMM.US.x.H9.M80194

IQQLQGLAI

>SMM.US.x.PGM53.AF077017

VQQLQDLSL

>SMM.US.x.SIVsmH635F_L3.DQ201172

IQQLQGLAI

>SMM.US.x.pE660.CG7G.JX648292

IQQLQGLAI

>SMM.US.x.pE660.CG7V.JX648291

IQQLQGLAI

>STM.US.89.STM_37_16.M83293

VQQLQGLSI

## SIVrcm Rev NES alignment

>RCM.CM.00.SIVagi_00CM312.HM803690

VEQLNQLTI

>RCM.CM.02.SIVrcm_02CM8081.HM803689

VEQLQQLAI

>AF382829_7 rev protein [Simian immunodeficiency virus]

IADLQKLQL

>ADK78276 rev protein [Simian immunodeficiency virus]

VEQLNQLTI

>ADK78267 rev protein [Simian immunodeficiency virus]

VEQLQQLAI

>AF349680_7 rev protein [Simian immunodeficiency virus]

LSNLQQLTL

# Env LLP2 alignments

## HIV-1 M Env LLP2 alignment

>B.FR.83.HXB2_LAI_IIIB_BRU.K03455

SYHRLRDLLL

>A.CD.87.2106.MH705158

SYHHLRDFIL

>A.CD.87.50.MH705161

SYHRLRDFIL

>A.CD.87.70641.MH705151

SYHRLRDFIL

>A.CD.87.P4039.MH705157

SYHRLRDFIL

>A.CD.87.PBS6126.MH705153

SYHRLRDFIL

>A.CD.87.PBS888.MH705133

SYHRLRDFIS

>A.CH.03.HIV_CH_BID_V3538_2003.JQ403028

SYHRLRDFAL

>A.CM.98.98CM_MP1014.AM279354

SYHRLRDFIL

>A.NG.09.09NG010499.KX389622

SYHRLRDFIL

>A.ZA.04.04ZASK162B1.DQ396400

SYHRLRDFIL

>A1.AU.04.PS1044_Day177.DQ676873

SYHRLRDLLS

>A1.BE.94.VI1383_20.FM165645

SYHLLRDFIL

>A1.BE.97.PIC771_15.FM165628

SYHRLRDFIL

>A1.BE.x.VI2809.EU191615

SYHRLRDFIL

>A1.CD.02.LA01AlPr.KU168256

SYHRLRDFVL

>A1.CM.03.CM54_7.KU168305

SYHRLRDFIL

>A1.CM.07.46_10.KP718918

SYHQLRDFIL

>A1.CM.08.886_24.KP718928

SYHRLRDFIL

>A1.CM.97.97CM_MP582.AM279343

SYHRLRDFIL

>A1.CM.97.97CM_MP640.AM279366

SYHRLRDFIL

>A1.CM.97.97CM_MP812.AM279344

SYHHLRDFIL

>A1.CM.99.99CM_MP1433.AM279348

CYHRLRDFIL

>A1.CY.05.CY023.FJ388894

SYHRLRDFIL

>A1.CY.05.CY051.FJ388903

SYHRLRDFIL

>A1.CY.05.CY064.FJ388909

SYHRLRDFIL

>A1.CY.05.CY106.FJ388925

SYHRLRDFIL

>A1.CY.05.CY121.FJ388932

SYHRLRDFIL

>A1.CY.05.CY140.FJ388938

SYHRLRDFIL

>A1.CY.05.CY153.FJ388942

SYRRLRDFVL

>A1.CY.06.CY154.FJ388943

SYHRLRDFIL

>A1.CY.06.CY164.FJ388946

IYHRLINFIL

>A1.CY.07.CY182.JF683737

SYHHLRDFIL

>A1.CY.07.CY207.JF683759

SYHRLRDFIL

>A1.CY.07.CY208.JF683760

SFLRLRDFIV

>A1.CY.07.CY209.JF683761

SYHRLRDFIL

>A1.CY.08.CY218.JF683767

SYHRLRDFTL

>A1.CY.08.CY230.JF683779

SYHRLRDFIS

>A1.CY.08.CY235.JF683782

SYHRLRDFIL

>A1.CY.08.CY236.JF683783

SYHRLRDFIL

>A1.CY.09.CY243.JF683789

SYHRLRDFIL

>A1.ES.05.X1608_8.FJ670519

SYHRLRDFIL

>A1.ES.06.X2110.FJ670523

SYHQLRDFVL

>A1.ES.15.100_117.KY496622

SYHRLRDFVS

>A1.ES.x.R23.JX422201

SYHRLRDFVL

>A1.FI.91.FIN91121.AF219261

SYHRLKDFIL

>A1.FI.91.FIN9199.AF219265

SYHRLRDFIL

>A1.GB.08.A220_C1.HQ595767

SYRRLRDFIL

>A1.GB.08.CH080117_e_p1.HM204635

SYHRLRDFIL

>A1.GB.08.CH080510_e_p2.HM204648

SYHRLRDFIL

>A1.GB.08.I230_A10.HQ595782

SYHRLRDFTL

>A1.GB.08.K160_H10.HQ595784

SYHHLRDFIL

>A1.GB.13.15171_1_44.3.MF109677

SYHRLRDFTL

>A1.GB.14.13612_1_42.3.MF109427

SYHLLRNFIL

>A1.GB.14.14727_1_50.3.MF109613

SYHRLRDLIL

>A1.GB.14.14727_1_62.3.MF109623

SYHRLRDLTL

>A1.GB.x.MA246.Y13718

SYHRLRDFTL

>A1.GB.x.MC108.Y13717

SYHRLRDFIL

>A1.GM.00.N9845.HQ385459

SYHRLRDFTL

>A1.GM.05.N33456.HQ385452

SYRHLRDFIL

>A1.GM.09.N057856.HQ385453

SYHRLRDLVL

>A1.IN.00.NARI_FLS_VB99_30.KT152846

SYHRLRDFSL

>A1.IN.09.NARI_FLS_IVC19_1.KT152839

SYHRLRDLLL

>A1.IN.97.NARI_FLS_VB6.KT152840

SYHRLRDFXL

>A1.IN.99.NARI_FLS_VB11.KT152841

SYHRLRDFVL

>A1.IN.99.NARI_FLS_VB15.KT152842

SYHRLRDFIL

>A1.IN.99.NARI_FLS_VB81_12.KT152844

SYHRLSDFIL

>A1.KE.00.00KE_KER2008.AY736809

SYHHLRDFIL

>A1.KE.00.00KE_KER2018.AY736810

LYHRLRDFIL

>A1.KE.00.00KE_KNH1144.AY736812

SYHRLRDFAL

>A1.KE.00.00KE_KNH1207.AY736815

SYHRLRDFIL

>A1.KE.00.00KE_KNH1209.AY736813

SYHRLRDFIL

>A1.KE.00.00KE_KSM4030.AY736816

SYHRLRDFIL

>A1.KE.00.KER2009.AF457053

SYHRLRNFIL

>A1.KE.00.KER2012.AF457055

SYHRLRDFIL

>A1.KE.00.KNH1199.AF457067

SYHRLRDFIL

>A1.KE.00.KNH1211.AF457070

SYHRLRDLIL

>A1.KE.00.KSM4024.AF457077

SYHRLRDFIL

>A1.KE.00.MSA4069.AF457080

SYRRLRDFIS

>A1.KE.00.MSA4070.AF457081

SYHRLRDFIL

>A1.KE.00.MSA4072.AF457083

SYHRLRDFIL

>A1.KE.00.MSA4076.AF457084

SYHRLRDFIL

>A1.KE.00.MSA4079.AF457086

SYHRLRDFIL

>A1.KE.00.NKU3005.AF457089

SYHRLRDFIL

>A1.KE.01.ML1945.EU110088

SYHRLRDFIL

>A1.KE.02.ML1990.EU110092

LYHRLRDLTL

>A1.KE.02.ML2014.EU110094

SYRRLRDFIL

>A1.KE.04.04KE169579V3.KT022360

SYHQLRDFVL

>A1.KE.04.04KE263806V2.KT022361

SYHRLRDFIL

>A1.KE.04.04KE354207V3.KT022363

SHHRLRDFIS

>A1.KE.04.04KE378531V2.KT022364

SYHRLRDFIL

>A1.KE.04.04KE406723V2.KT022365

SYHRLRDFIL

>A1.KE.04.04KE649309V2.KT022367

SYHRLRDFSL

>A1.KE.04.04KE809842V2.KT022368

SYHRLRDFIL

>A1.KE.04.04KE860822V3.KT022369

SYHRLRDFIL

>A1.KE.04.QG984_21M_ENV_A3.FJ866117

SYHRLKDFIL

>A1.KE.05.05KE185405V4.KT022370

SYHRLRDFIL

>A1.KE.05.05KE376579V4.KT022372

SYHLLRDFIL

>A1.KE.05.05KE520997V4.KT022373

SYHRLRDFIL

>A1.KE.05.05KE607907V4.KT022374

SYHRLRDFAL

>A1.KE.05.05KE643439V4.KT022375

SYHRLRDCIL

>A1.KE.05.05KE851891V4.KT022376

CYHRLRDFIL

>A1.KE.05.05KE884468V5.KT022377

LYHRLRDFTL

>A1.KE.05.QF495_23M_ENV_D1.FJ866116

SYHRLRDFIL

>A1.KE.05.QH209_14M_ENV_A2.FJ866118

SYHRLRDFIL

>A1.KE.05.QH343_21M_ENV_B5.FJ866120

CYHRLRDFIL

>A1.KE.05.QH359_21M_ENV_C1.FJ866121

SYHRLRDFVL

>A1.KE.06.06KE335214V6.KT022380

SYHRLRDFIL

>A1.KE.06.06KE404877V7.KT022381

SYHRLRDFIL

>A1.KE.06.06KE452693V6.KT022382

SYHRLRDFTL

>A1.KE.06.06KE661996V6.KT022383

SYHRLRDFIL

>A1.KE.06.06KECst_001.FJ623487

SYHRLRDFIL

>A1.KE.06.06KECst_005.FJ623481

SYHRLRDFVS

>A1.KE.06.06KECst_006.FJ623475

SYHRLRDFIS

>A1.KE.06.06KECst_007.FJ623476

SYHRLRDFIL

>A1.KE.06.06KECst_009.FJ623480

SYHRLRDFVL

>A1.KE.06.06KECst_013.FJ623485

SYHRLRDFIL

>A1.KE.06.06KECst_016.FJ623483

SYHRLRDFIL

>A1.KE.06.06KECst_017.FJ623488

SYHRLRDFTL

>A1.KE.06.06KECst_019.FJ623478

SYHRLRDFIL

>A1.KE.06.06KECst_020.FJ623482

CYHRLRDLIS

>A1.KE.06.06KECst_021.FJ623477

SYHRLRDFAL

>A1.KE.06.06KECst_025.FJ623486

SYHRLRDFIL

>A1.KE.06.06KECst_028.FJ623479

SYHRLRDFIL

>A1.KE.07.21020_13.HM215275

SYHRLRDFIL

>A1.KE.08.Donor_64.KT252544

SYHLLRDFVL

>A1.KE.09.12151802.HQ540689

SYHRLRDFIL

>A1.KE.11.023_KE.KU921708

SYHRLRDFVL

>A1.KE.11.024_KE.KU921712

SYHRLRDFIS

>A1.KE.11.103_KE.KU921727

SYHRLRDFIL

>A1.KE.11.209_KE.KU921732

SYHRLRDFIL

>A1.KE.11.211_KE.KU921734

SYHRLRDFIS

>A1.KE.11.263_KE.KU921738

SYHRLRDFIL

>A1.KE.11.320_KE.KU921746

SYHHLRDFAL

>A1.KE.11.339_KE.KU921752

SYHRLRDFIL

>A1.KE.11.366_KE.KU921756

SYHRLKNFIL

>A1.KE.11.374_KE.KU921759

CYHRLRDFIL

>A1.KE.11.388_KE.KU921772

SYHRLRDFIL

>A1.KE.11.391_KE.KU921778

SYHRLRDFSL

>A1.KE.11.405_KE.KU921780

SYQRLRDFTL

>A1.KE.11.407_KE.KU921783

SYHRLRDFIL

>A1.KE.11.426_KE.KU921785

SYHRLRDFIL

>A1.KE.11.467_KE.KU921788

LYRRLRDFIL

>A1.KE.11.511_KE.KU921804

SYHRLRDCSL

>A1.KE.11.655_KE.KU921831

SYHRLRDFIS

>A1.KE.11.657_KE.KU921835

SYHRLRDFIL

>A1.KE.11.713_KE.KU921841

SYHRLRDCIL

>A1.KE.11.717_KE.KU921852

SYHRLRDFIL

>A1.KE.11.DEMA111KE002.KF716474

SYHRLRDFIL

>A1.KE.11.DEMA11KE001.KF716475

SYHRLRDFTL

>A1.KE.86.ML013_10.AY322184

SYHRLRDLIL

>A1.KE.86.ML170_1986.AF539405

SYHRLRDFIL

>A1.KE.90.K89_KENYA_KE89.L22943

SYHRLRDFIL

>A1.KE.94.MF520.W14M.HH2.KX168075

SYHRLRDFIL

>A1.KE.94.Q23_17.AF004885

SYHRLRDFIL

>A1.KE.94.Q259_w6_PNS59d.AF407151

SYHRLRDFIS

>A1.KE.94.Q842_d16.AF407162

SYHRLRDFIL

>A1.KE.95.Q461_e2.AF407156

SYHRLRDFIL

>A1.KE.95.QB850.73p.E3.KT008652

SYHRLRDFIL

>A1.KE.96.Q769_b9_PNS79d.AF407157

SYHRLRDFIL

>A1.KE.96.QB726_70M_ENV_C4.FJ866112

SYHRLRDFIL

>A1.KE.97.ML605_3.AY322190

SYHRLRDFIL

>A1.KE.97.ML752.AY322193

SYHRLRDFIL

>A1.KE.98.QA413_1007M_ENV_E15.FJ396018

SYHRLRDFIL

>A1.KE.99.99KE_KNH1088.AY736811

SYHRLRDFIL

>A1.KE.99.99KE_KNH1135.AY736814

CYHRLRDFIL

>A1.KE.99.KSM4021.AF457075

SYHRLRDFIL

>A1.KE.x.BG505_W6M_ENV_C2.DQ208458

CYHRLRDFIL

>A1.KE.x.BI206_W6P_ENV_C1.DQ208467

SYHRLRDFIS

>A1.KE.x.BJ613_W6M_ENV_E1.DQ208448

SYHRSRDFIL

>A1.PK.14.DEMA114PK013.KU749411

SYHRLRDFIS

>A1.PK.14.PK001.KX232594

SYHRLRDLIL

>A1.PK.14.PK002.KX232595

SYHRLRDFIL

>A1.PK.14.PK004.KX232597

SYHRLRDLLL

>A1.PK.14.PK007.KX232600

SYHRLRDFIW

>A1.PK.14.PK016.KX232609

SYHRLRDLLL

>A1.PK.14.PK017.KX232610

SYHRLRDLLL

>A1.PK.14.PK018.KX232611

SYHRLRDLLL

>A1.PK.14.PK020.KX232613

SYHRLRDFIL

>A1.PK.15.PK021.KX232614

SYLHLRDLVL

>A1.PK.15.PK026.KX232618

SYHRLRDFIL

>A1.PK.15.PK030.KX232620

SYHRLRDFIL

>A1.PK.15.PK034.KX232624

SYHRLRDFIL

>A1.PK.15.PK036.KX232626

SYRRLRDFIL

>A1.RW.06.DEMA106RW003.KU749423

SYHRLRDFIL

>A1.RW.06.R1141M_17Oct06_3A3.KX983643

SYHHLRDFIL

>A1.RW.07.R283F_7Dec07_3C4.KX983542

SYHLLRNLVL

>A1.RW.07.R774F_3SEP07_B22.KX983566

SYHRLRDFIL

>A1.RW.07.pR463F.JX236677

SYHRLRDFIL

>A1.RW.07.pR880F.JX236678

SYHRLRDFIL

>A1.RW.08.DEMA108RW010.KU749424

SYHRLRDFIL

>A1.RW.08.PC64_M03c001.MF565934

SYHRLRDFIL

>A1.RW.08.R1135M_11Feb08_3B9.KX983624

SYHRLRDFIL

>A1.RW.11.DEMA111RW002.KF716472

SYHRLRDLLL

>A1.RW.92.92RW008.AB253421

SYHRLRDFIL

>A1.RW.92.92RW025A.AB287377

SYHRLRDFIL

>A1.RW.93.93RW037A.AB287379

SYHLLRNFIL

>A1.RW.93.93RW_024.AY713406

CYHRLRDFTL

>A1.RW.x.PVPI_KIG93.L07082

SYHRLRDFIL

>A1.SE.12.064GR.MF373167

SYRRLRDCIL

>A1.SE.12.065SE.MF373168

IYHRLRDFIL

>A1.SE.13.078SE.MF373181

SYHRLRDLVL

>A1.SE.13.079KE.MF373182

SYHRLRDFIL

>A1.SE.93.001UG.MF373124

SYHRLRDFIL

>A1.SE.94.SE7253.AF069670

SYHRLRHFIL

>A1.SE.94.SE7535.AF069671

SYHRLRDLIL

>A1.SE.95.SE8538.AF069669

SYHRLRDFIL

>A1.SE.95.SE8891.AF069673

CYRRLRDFIL

>A1.SE.95.UGSE8131.AF107771

SYHRLRDFIS

>A1.TZ.00.216_F2_A1.HQ659604

SYHRLRDFIL

>A1.TZ.00.21_F1_D2.HQ659597

SYHRLRDFIL

>A1.TZ.00.398_F1_A1.HQ697994

SYHRLRDFIL

>A1.TZ.00.515_F4_A9.HQ698029

SYHRLRDFIL

>A1.TZ.01.A173.AY253305

SYHRLRDFTL

>A1.TZ.01.A341.AY253314

SYHRLRDFIL

>A1.TZ.02.CO0783V0.KX907352

SYHRLRDFIL

>A1.TZ.03.CO0543V2.KX907348

RYHRLRDFTL

>A1.TZ.04.CO0272V4.KX907336

SYHQLRDFIL

>A1.TZ.04.CO0330V4.KX907343

SYHRLRDFIL

>A1.TZ.04.CO3365V2.KX907372

SYHRLRDLIL

>A1.TZ.04.CO3718V3.KX907383

SYHRLRDFIL

>A1.TZ.04.CO3878V2.KX907389

SYHRLRDFIL

>A1.TZ.05.CO0260V5.KX907341

SYHRLRDFIL

>A1.TZ.05.CO0439V5.KX907347

SYHRLRDFIL

>A1.TZ.05.CO3083V4.KX907364

SYHRLRDCIL

>A1.TZ.05.CO6161V5.KX907401

SYHRLRDFIL

>A1.TZ.05.CO6592V5.KX907412

SYHRLRDFIL

>A1.TZ.06.CO0434V7.KX907346

SYHRLRDFIL

>A1.TZ.06.CO3504V7.KX907378

SYHRLRDFIL

>A1.TZ.06.CO6637V7.KX907414

SYHRLRDFIL

>A1.TZ.06.CO6830V7.KX907423

CYHRLRDFIL

>A1.TZ.06.CO6974V7.KX907431

SYHRLRDFIL

>A1.TZ.08.707010095_D8.HQ615968

SYHRLRDFTL

>A1.TZ.08.707010117_C3.HQ615970

SYHRLRDFIL

>A1.TZ.08.707010240_A7.HQ615971

SYHHLRDFIL

>A1.TZ.08.707010627_E3.HQ615979

SYHRLRDFIL

>A1.TZ.08.CH0175_e2.HM204620

SYHRLRDFVL

>A1.TZ.08.CH0219_e4.HM204621

SYHRLRDFIL

>A1.TZ.08.DEMA108TZ002.KY658694

SYHRLRDFIL

>A1.TZ.08.DEMA108TZ004.KY658695

SYHQLRDFVL

>A1.TZ.08.DEMA108TZ012.KY658696

SYHHLRDLVL

>A1.TZ.97.97TZ02.AF361872

SYHRLRDFIL

>A1.TZ.97.97TZ03.AF361873

IYHRLRDLAL

>A1.UG.00.PP6_F2_B1.HM027846

SYHRLRDFIL

>A1.UG.07.0086_A1.JX877522

CYHRLRDFIL

>A1.UG.07.191955_A11.HM215272

SYHRLRDLLL

>A1.UG.07.PP6_F3_B2.HM027824

SYHRLRDFIL

>A1.UG.07.p191084.JX236669

SYHHLRDLIL

>A1.UG.07.p191845.JX236671

SYHRLRDFTL

>A1.UG.07.p9004SDM.JX236676

CYHRLRDFIL

>A1.UG.09.DEMA109UG001.KF716478

SYRRLKDLIL

>A1.UG.09.DEMA109UG017.KP109490

SYHRLRDLTL

>A1.UG.09.M160b_p1.JF680921

SYHHLRDFIL

>A1.UG.11.DEMA110UG001.KF859745

SYHLLRDFIL

>A1.UG.11.DEMA110UG009.KF716486

SYHRLRDFGL

>A1.UG.85.U455_U455A.M62320

SYHRLRDFAL

>A1.UG.90.UG273A.L22957

SYHRLRDFIL

>A1.UG.90.UG275A.L22951

SYHRLRDFIL

>A1.UG.92.92UG037_A40.AB253429

SYHRLRDFIL

>A1.UG.92.UG029.AY494973

SYHQLRDFIL

>A1.UG.97.368MPc02.EU853062

SYHRLRDFIL

>A1.UG.97.pt185.EU281999

SYHRLRDFIS

>A1.UG.98.120FIc01.EU852958

SYHRLRDFIL

>A1.UG.98.120MPC01.EU852954

SYHRLRDFIL

>A1.UG.98.98UG57134.AF484507

SYHRLRDFIL

>A1.UG.98.98UG57135.AF484508

SYHRLRDLVL

>A1.UG.98.98UG57136.AF484509

SYHRLRDFIL

>A1.UG.98.98UG57142.AF484512

SYHRLRDFIL

>A1.UG.99.601MPC7.EU853030

SYHRLRDFIL

>A1.UG.99.99UGA07072.AF484478

SYHRLRDFIL

>A1.UG.99.99UGG03379.AF484493

SYHRLRDFVL

>A1.UG.x.UG031.AB098330

SYHRLRDFIS

>A1.US.x.HCPI_13_01.GU367412

SYHRLRDFTL

>A1.ZA.00.TV096.KJ948658

SYHRLRDFIW

>A1.ZA.01.TV314.FJ647148

SYHRLRDFIL

>A1.ZA.04.503_15344_T10_A1.KT183312

SYHRLRDCVL

>A1.ZA.06.C.x.06.CF01_C8.KC863311

SYHHLRDFIL

>A1.ZA.09.707PKE02N3.HM623589

SYHRLRDLVL

>A1.ZA.09.MSM066.KF725913

SYHRLRDFIL

>A2.CD.87.PBS1195.MH705163

SYHRLRDCIL

>A2.CD.97.97CDKS10.AF286241

SYHRLRDCIL

>A2.CD.97.97CDKTB48.AF286238

SYHRLRDCIL

>A2.CM.01.01CM_1445MV.GU201516

SYHRLRDCIS

>A2.CY.94.94CY017_41.AF286237

SYHRLRDCIL

>A3.SN.01.DDI579.AY521629

SYHRLRDFTL

>A3.SN.01.DDJ369.AY521631

SYHRLRDFTL

>A3.SN.96.DDJ360.AY521630

SYHRLRDFTL

>A4.CD.97.97CD_KCC2.AM000053

VYHRSRDFIL

>A4.CD.97.97CD_KTB13.AM000054

SYHRLRDFIL

>A6.BY.13.PV85.KT983615

SYHRLRDLIS

>A6.CY.05.CY021.FJ388892

SYHQLRDFIL

>A6.CY.06.CY171.FJ388950

SYHRLIDFIS

>A6.CY.07.CY213.JF683763

LYHRLRDFIS

>A6.CY.09.CY255.JF683798

IYHRLRDLIS

>A6.GB.13.15228_1_59.3.MF109697

SYHRLRDLIS

>A6.IT.02.60000.EU861977

SYHRLKDFVS

>A6.RU.00.RU00051.EF545108

LYHRLRDFIL

>A6.RU.05.RU_560_1125_JA.JQ292895

LYHRLRDFIL

>A6.RU.06.06_RU_SP_SC1233_VI_2D6.GU481566

SYHRLRDFIL

>A6.RU.06.06_RU_SP_SC1457_VI_E12.GU481615

SYHRLRDLIS

>A6.RU.06.RU_39_1119_JA_A6_4.HQ896489

SYHRLRDFIS

>A6.RU.06.RU_915_1013_A8.HQ385830

SYHRLRDFIS

>A6.RU.06.RU_915_1016.JQ292896

SYHRLRDFIL

>A6.RU.06.RU_915_1035.JQ292897

SYHRLRDFIS

>A6.RU.06.RU_915_1038.JQ292898

SYHRLRDFIS

>A6.RU.06.RU_915_1041.JQ292899

SYHRLRDFIS

>A6.RU.06.RU_915_1074_F2.HQ385841

SYRRLRDFIL

>A6.RU.06.RU_915_1080_D4.HQ385843

SYHRLRDFIS

>A6.RU.06.RU_D_03_B10.HQ385846

SYHRLRDFIL

>A6.RU.06.RU_SP_B_049.JQ292900

SYHRLRDFIS

>A6.RU.07.07_RU_SP_R497_IV_B16.GU481434

SYHRLRDFIS

>A6.RU.07.07_RU_SP_R526_IorII_A30.GU481486

SYHRLRDFIS

>A6.RU.07.07_RU_SP_SC3208_VI_H7.GU481644

SYHRLRDFIL

>A6.RU.07.Irkutsk_5.JQ292891

SYHRLRDFIS

>A6.RU.07.RU_064_07_B2.HQ385818

SYHRLRDFIS

>A6.RU.07.RU_26_07_A8.HQ385820

SYHRLRDFIS

>A6.RU.07.RU_35_07_D2.JF952018

SYHRLRDFIL

>A6.RU.07.RU_41_07_A9_2.HQ338112

SYHRLRDFIL

>A6.RU.08.08_RU_SP_K08_VI_C11.GU481276

SYHRLRDLIL

>A6.RU.08.08_RU_SP_K84_IV_A3.GU481277

SYHRLRDFIS

>A6.RU.08.08_RU_SP_R053_IV_D11.GU481350

LYHRLGDFIS

>A6.RU.08.08_RU_SP_R589_VI_0A3.GU481544

SYHLLRDFIL

>A6.RU.08.08_RU_SP_SC3410_VI_C2.GU481661

SYHRLRDFIS

>A6.RU.08.DEMA108RU003.KF716491

SYHRLRDLIS

>A6.RU.08.DEMA108RU004.KF716492

SYHRLRDFIL

>A6.RU.08.MSK_SK_006_2.KY238327

SYHRLRDFIS

>A6.RU.08.PokA1Ru.FJ864679

SYHRLRDFIS

>A6.RU.08.RUA001.JQ292893

SYHRLRDFIS

>A6.RU.08.RUA004g.HQ834965

SYHRLRDLIS

>A6.RU.08.RUA007.JQ292894

SYHRLRDFIL

>A6.RU.08.RUA009h.JF430898

SYHRLRDFIS

>A6.RU.08.RUA021c3.HQ834967

SYHRLRDFIS

>A6.RU.10.10RU6617.JX500696

SYHRLRDFIS

>A6.RU.10.10RU6792.JX500695

SYHRLRDLIS

>A6.RU.11.11RU6950.JX500694

SYHRLRDFIL

>A6.RU.11.AHI_A_1_2C12.MH603967

CYHRLRDFIL

>A6.RU.11.AHI_D_10_2B11.MH604120

CYHRLRDFIS

>A6.RU.11.AHI_K_1_2B6.MH604308

SYHRLRDFIS

>A6.RU.11.AHI_M_2_A4.MH604056

SYHLLRDFIS

>A6.RU.11.AHI_N_1_2A12.MH604408

SYHRLRDFIL

>A6.RU.11.CHR_E_B11.MH604109

SYHRLRDFIS

>A6.RU.11.CHR_H_A2.MH604508

SYHRLRDFIL

>A6.RU.11.CHR_I_B10.MH604524

SYHRLRDFIS

>A6.RU.11.CHR_J_D7.MH604541

SYHRLRDFIS

>A6.RU.13.ARH001.MG902950

SYHRLRDFIS

>A6.RU.13.ARH011.MG902951

SYHRLRDFIS

>A6.RU.x.RUA022a2.HQ616082

SYHRLRDFIS

>A6.UA.01.01UADN139.DQ823357

SYHRLRDFIS

>A6.UA.11.DEMA111UA002.KU749399

SYRRLRDFIL

>A6.UA.11.DEMA111UA008.KU749400

SYRLLRDFVS

>A6.UA.11.DEMA111UA009.KU749401

SYHRLRDFIL

>A6.UA.12.DEMA112UA013.KY658681

CYRRLRDFIL

>A6.UA.12.DEMA112UA014.KU749402

SYHQLRDFIL

>A6.UA.12.DEMA112UA024.KU749403

SYHRLRDFIL

>A6.UA.12.DEMA112UA030.KU749404

SYHRLKDFIL

>A6.UA.12.DEMA112UA034.KU749405

SYHRLRDFIL

>A6.UA.12.DEMA112UA036.KU749406

SYHRLRDFIL

>A6.UA.12.DEMA112UA040.KY658682

SYHRLRDFIL

>A6.UA.12.DEMA112UA042.KU749407

SYHRLRDFIS

>A6.UA.97.ukr970063.AF082486

SYRRLRDFIS

>B.AR.00.85096FL_2000.KY968401

SYRRLSDLLL

>B.AR.00.85323FL_2000.KY968402

SYHRLRDLLL

>B.AR.00.85891FL_2000.KY968403

SYHRLRDLLL

>B.AR.00.86218FL_2000.KY968404

SYHRLRDLLL

>B.AR.00.ARMS008.AY037269

SYHHLRDLIL

>B.AR.01.101815FL_2001.KY968394

SYHRLTDFLL

>B.AR.01.107878FL_2001.KY968395

SYHQLRNLLL

>B.AR.01.89825FL_2001.KY968407

SYHRLRDLLL

>B.AR.01.89886FL_2001.KY968408

SYHRLRDLLL

>B.AR.02.02AR114146.DQ383746

SYHRLRDLLL

>B.AR.02.116877FL_2002.KY968396

SYHRLRDLLL

>B.AR.03.03AR137681.DQ383748

SYRRLRDLLL

>B.AR.03.03AR138910.DQ383749

SYHRLRDLLL

>B.AR.03.134742FL_2003.KY968397

SYRRLRDLLL

>B.AR.04.04AR151263.DQ383751

SYHRLRDLLL

>B.AR.04.04AR151516.DQ383752

SYRHLRDLLL

>B.AR.04.145447FL_2004.KY968398

SYHRLRDLLL

>B.AR.04.159687FL_2004.KY968399

SYHHLRDFLL

>B.AR.07.DEURF07AR001.KY658686

SYHHLRDLLL

>B.AR.08.DEMB08AR002.KY658683

SYHQLRDLLL

>B.AR.09.DEMB09AR010.MH078530

SYHRLRDLLL

>B.AR.10.DEMB10AR006.KY658684

CYHRLRDLLL

>B.AR.14.DEMB14AR003.KY658685

SYHHLRDLLL

>B.AR.14.DEMB14AR012.MH078531

SYHRLRDLIL

>B.AR.15.DEMB15AR013.MH078532

SYHRLRDLLL

>B.AR.15.DEMB15AR014.MH078533

IYHRLRDLVL

>B.AR.98.ARCH054.AY037268

IYHRLRDLLL

>B.AR.99.ARMA132.AY037282

SYHRLRDLLL

>B.AT.x.Au02.FJ952154

SYHRLRDLLL

>B.AU.03.PS2008_Day182.DQ676875

SYHRLRDLLL

>B.AU.03.PS2019_Day171.DQ676881

SYHRLRDFLL

>B.AU.03.PS3002_Day385.DQ676885

SYHHLRDLLL

>B.AU.03.PS4048_Day0.DQ676886

SYHRLRDLLL

>B.AU.04.MS2004_37_060.EF178358

SYHRLRDLLL

>B.AU.04.PS1038_Day174.DQ676871

SYHRLRDLLL

>B.AU.04.PS2016_Day380.DQ676879

SYHRLRDLLL

>B.AU.86.MBC200.AF042100

SYHRLRDLLS

>B.AU.87.MBC925.AF042101

SYHRLRDLLL

>B.AU.93.MBC18_MBCC18.AF042102

SYHRLRDLLL

>B.AU.95.C24.AF538304

SYHRLRDLLL

>B.AU.95.C76.AF538306

SYRLLRDLLL

>B.AU.95.C92.AF538307

SYHRLRDLLL

>B.AU.95.MBCC54.AF042103

SYHHLRDLLL

>B.AU.96.MBCC98.AF042104

SYHHLRDLLL

>B.AU.96.MBCD36.AF042105

LYHHLRDLLL

>B.AU.99.1181.AF538302

SYHRLRDLLL

>B.AU.x.15888_12.AY624305

SYRHLRDLLL

>B.AU.x.2870718.AY857022

SYHRLRDLLL

>B.AU.x.4675282.AY857052

SYHRLRDLLL

>B.AU.x.7894552.AY857127

SYHRLRDLLL

>B.AU.x.8634991.AY857144

SYHRLRDLLL

>B.AU.x.9125091.AY857165

SYHRLRDLLL

>B.AU.x.CB1pbmc2.JN002002

SYHRLRDLLL

>B.AU.x.CB3csf2.JN002009

SYHRLRDLLL

>B.AU.x.HCPI_07_01.GU367406

SYHRLRDLLL

>B.AU.x.HCPI_08_02.GU367398

SYRHLRDLLL

>B.AU.x.HCPI_10_01.GU367395

SYHCLRDLLL

>B.AU.x.HCPI_11_01.GU367404

SYHRLRDLHL

>B.AU.x.VH_VHPCR.AF146728

SYHRLRDLLL

>B.BE.05.N2_WEEK_8.FJ653258

SYHRLRDLLL

>B.BE.90.VI423___53.DQ313249

IYHRLRDLLL

>B.BE.91.SIMI84_HSIMI84.L07421

SYHRLRDLLL

>B.BE.93.VI843_172.DQ313250

SYHRLRDLLL

>B.BE.94.VI1399.DQ313253

LYHRLRDLLL

>B.BE.96.VI1273.DQ313252

SYHRLRDLLL

>B.BE.x.VI886_1.EU191616

SYHRLRDLLL

>B.BO.99.BOL0122.AY037270

SYHRLRDLLL

>B.BR.02.02BR002.DQ358805

SYHRLRDLLL

>B.BR.02.02BR008.DQ358808

SYHRLRDLLL

>B.BR.02.02BR011.DQ358809

SYHRLRDLLL

>B.BR.02.02BR013.DQ358810

SYHRLRDLLL

>B.BR.02.02BR1013.JN692432

SYHRLIDLLL

>B.BR.02.02BR2025.JN692435

SYHRLRDLLL

>B.BR.02.02BR2032.JN692439

LYHRLRDLLL

>B.BR.02.02BR2033.JN692440

SYHRLRDLLL

>B.BR.02.02BR2041.JN692443

LYHRLRDLLL

>B.BR.02.02BR2042.JN692444

SYRRLRDLLL

>B.BR.02.04BR1064.JN692433

SYHRLRDLLL

>B.BR.03.03BR1020.JN692445

SYHHLRDLLL

>B.BR.03.03BR1046.JN692447

SYHRLKDLLL

>B.BR.03.BREPM1023.EF637057

SYPRLEDLPP

>B.BR.03.BREPM1024.EF637056

SYHHLRDLLL

>B.BR.03.BREPM1027.EF637054

SYRHLRNLLL

>B.BR.03.BREPM1028.EF637053

SYHLLRDLLL

>B.BR.03.BREPM1032.EF637051

SYHRLRDLLL

>B.BR.03.BREPM1033.EF637050

SYRRLRDLLL

>B.BR.03.BREPM1035.EF637049

SYHRLRDLLL

>B.BR.03.BREPM1038.EF637048

SYRHLRDLLL

>B.BR.03.BREPM1040.EF637047

SYHRLRDLLL

>B.BR.03.BREPM2012.EF637046

SYHRLRDLLL

>B.BR.04.04BR1047.JN692450

SYRHLRDLLL

>B.BR.04.04BR1049.JN692451

SYHRLRDLLL

>B.BR.04.04BR1051.JN692452

SYHRLRDLLL

>B.BR.04.04BR1054.JN692453

SYHRLRDLLL

>B.BR.04.04BR1055.JN692454

SYHLLRDFLL

>B.BR.04.04BR1057.JN692455

SYHHLRNLLL

>B.BR.04.04BR1068.JN692457

SYHRLTDLLL

>B.BR.04.BREPM1066.FJ195090

SYHRLRDLLL

>B.BR.04.BREPM1070.FJ195086

IYHRLRDLLL

>B.BR.05.05BR1074.JN692459

SYHRLRDLLL

>B.BR.05.05BR1077.JN692460

SYHRLRDLLL

>B.BR.05.05BR1078.JN692461

SYHHLRDLLL

>B.BR.05.05BR1079.JN692462

SYHRLRDLLL

>B.BR.05.05BR1080.JN692463

SYHHLRDLLL

>B.BR.05.05BR1082.JN692465

SYHQLRDLLL

>B.BR.05.05BR1089.JN692467

SYHRLRDLLL

>B.BR.05.05BR1092.JN692468

SYHRLRDLLL

>B.BR.05.05BR1095.JN692471

SYHLLRDSLL

>B.BR.05.05BR1101.JN692473

SYHRLRDLLL

>B.BR.05.05BR1104.JN692474

SYHRLRDLLL

>B.BR.05.05BR1107.JN692475

SYHRLRDLLL

>B.BR.05.BREPM1081.FJ195091

SYHRLRDLLL

>B.BR.05.BREPM1084.FJ195088

SYHRLRDLLL

>B.BR.05.BREPM1093.FJ195089

SYHRLRDLLL

>B.BR.06.06BR1115.JN692479

SYHRLRDLLL

>B.BR.06.06BR1119.JN692480

SYHRLRDLLL

>B.BR.06.P1357_E2.HQ236571

SYHRLRDLLS

>B.BR.06.subject_498_day1_clone_25.HQ377443

SYRRLRDLLL

>B.BR.06.subject_784_day1_clone_16.HQ377487

LYHRLRDLLL

>B.BR.07.BP00047_RH01.JN687739

SYRHLRDLLL

>B.BR.07.subject_485_week48_clone_02.HQ377423

SYHRLRDLLL

>B.BR.07.subject_500_day1_clone_04.HQ377459

SYHRLRDLLL

>B.BR.09.C522_p1.JF680907

SYHRLRDLLL

>B.BR.09.DEMB09BR040.KU749389

SYHRLRDLLL

>B.BR.10.10BR_MG006.KT427690

SYHHLRDLLL

>B.BR.10.10BR_MG007.KJ849786

SYHRLRDSLL

>B.BR.10.10BR_MG009.KT427689

SYRNLRDLLL

>B.BR.10.10BR_MG011.KT427688

--------AL

>B.BR.10.10BR_MG012.KT427687

SYRQLRNLLS

>B.BR.10.10BR_MG013.KT427686

SYHRLRDLLL

>B.BR.10.10BR_MG014.KT427685

SYRNLRDLLL

>B.BR.10.10BR_MG016.KT427684

SYHRLRDLLL

>B.BR.10.10BR_MG018.KT427683

IYHRLRDLLL

>B.BR.10.10BR_MG020.KT427682

SYHRLRDLLL

>B.BR.10.10BR_MG024.KT427681

SYHRLADLLS

>B.BR.10.10BR_MG028.KT427680

SYHRLRDLLL

>B.BR.10.10BR_MG031_2.KT427841

SYHRLRDLLL

>B.BR.10.10BR_MG035.KJ849804

SYHRLRDLLL

>B.BR.10.10BR_MG038.KT427676

SYHRLRDLLL

>B.BR.10.10BR_MG039.KT427675

SYHRLRDLLL

>B.BR.10.10BR_MG041.KT427673

SYHRLRDLLL

>B.BR.10.10BR_MG044.KT427671

IYHHLRDLLL

>B.BR.10.10BR_MG045.KT427670

SYHRLRDLLL

>B.BR.10.10BR_MG049.KT427669

SYRQLRDLIL

>B.BR.10.10BR_PE003.KT427747

IYHQLRDLLL

>B.BR.10.10BR_PE006.KT427745

SYHRLRDLLL

>B.BR.10.10BR_PE010.KJ849788

SYHRLRDLLL

>B.BR.10.10BR_PE012.KT427744

SYHRWRDLLL

>B.BR.10.10BR_PE013.KT427743

SYHRLSDLLL

>B.BR.10.10BR_PE014.KT427742

SYHRLRDLLS

>B.BR.10.10BR_PE019.KT427740

SYRRLSDLLL

>B.BR.10.10BR_PE020.KT427739

SYHRLRDLLL

>B.BR.10.10BR_PE021.KT427738

LYHRLRDLLL

>B.BR.10.10BR_PE022.KT427737

SYHSLRDLLL

>B.BR.10.10BR_PE024.KT427735

CYHRLTDLLS

>B.BR.10.10BR_PE028.KT427734

SYHRLTDLLL

>B.BR.10.10BR_PE030.KT427733

SYHRLRDLLL

>B.BR.10.10BR_PE031.KT427732

SYHRLRDLLL

>B.BR.10.10BR_PE033.KT427731

SYHRLRDLLL

>B.BR.10.10BR_PE034.KJ849803

SYHRLRDLLL

>B.BR.10.10BR_PE035.KT427730

SYRRLRDLLL

>B.BR.10.10BR_PE036.KT427729

SYHRLRDLLL

>B.BR.10.10BR_PE037.KT427728

SYHRLRDLLL

>B.BR.10.10BR_PE038.KT427727

SYHRLRDLLL

>B.BR.10.10BR_PE040.KT427725

SYXXLRDLLX

>B.BR.10.10BR_PE041.KT427724

CYHRLRDLLL

>B.BR.10.10BR_PE042.KT427723

SYHRLRDLLL

>B.BR.10.10BR_PE044.KT427721

SYHRLRDLLL

>B.BR.10.10BR_PE045.KT427720

CYRLLRDLLL

>B.BR.10.10BR_PE047.KT427719

SYHRLRDLLL

>B.BR.10.10BR_PE048.KT427718

SYHRLRDLLL

>B.BR.10.10BR_PE049.KT427717

SYHRLRDLIL

>B.BR.10.10BR_PE051.KT427716

SYHRLRDLLL

>B.BR.10.10BR_PE052.KT427715

SYHRLRDLLL

>B.BR.10.10BR_PE053.KJ849767

IYHHLRDFLL

>B.BR.10.10BR_PE054.KT427714

SYHRLRDLLL

>B.BR.10.10BR_PE055.KT427847

SYRHLRDLLL

>B.BR.10.10BR_PE058.KT427713

LYHRLRDLLL

>B.BR.10.10BR_PE061.KT427870

SYHRLRDLLL

>B.BR.10.10BR_PE067.KT427712

SYHRLRDLLL

>B.BR.10.10BR_PE068.KT427711

SYHHLRDLLL

>B.BR.10.10BR_PE070_2.KT427853

SYHRLRDLLL

>B.BR.10.10BR_PE072.KT427709

SYHRLRDLLL

>B.BR.10.10BR_PE074.KT427708

SYHRLRDLLL

>B.BR.10.10BR_PE076.KT427707

SYHRLRDLLL

>B.BR.10.10BR_PE077.KT427706

SYHRLRDLLL

>B.BR.10.10BR_PE079.KT427704

SYHRLRDLLL

>B.BR.10.10BR_PE081.KT427872

SYHRLRDLLL

>B.BR.10.10BR_PE082.KT427703

SYHRLRDLLL

>B.BR.10.10BR_PE083.KT427702

LYHRLRDLLL

>B.BR.10.10BR_PE089.KT427873

SYHRLRDLLL

>B.BR.10.10BR_PE091.KJ849817

SYHRLRDLLW

>B.BR.10.10BR_PE093.KT427701

SYHRLRDLLL

>B.BR.10.10BR_PE095.KT427700

SYHRLRDLLL

>B.BR.10.10BR_PE096.KT427699

SYHRLRDLLL

>B.BR.10.10BR_PE097.KJ849818

SYHRLRDLLL

>B.BR.10.10BR_PE100.KJ849820

IYHRLRDLLL

>B.BR.10.10BR_PE101.KT427697

SYHRLRDLLL

>B.BR.10.10BR_PE103.KT427696

SYHRLRDLLL

>B.BR.10.10BR_PE104.KJ849780

SYHRLRDLIL

>B.BR.10.10BR_PE105.KT427695

SYHRLRDLLL

>B.BR.10.10BR_PE106.KT427694

SYHRLRDLLL

>B.BR.10.10BR_PE110.KT427692

SYHRLRDLLL

>B.BR.10.10BR_PE112.KT427691

SYRRLRDLLL

>B.BR.10.10BR_RJ005.KT427792

SYHRLRDLLL

>B.BR.10.10BR_RJ008.KT427791

SYLRLRDLIL

>B.BR.10.10BR_RJ016.KT427788

SYHRLRDLLL

>B.BR.10.10BR_RJ017.KT427787

SYHRLKDLLL

>B.BR.10.10BR_RJ018_2.KT427861

SYHRLRDLLL

>B.BR.10.10BR_RJ019.KT427786

SYHHLRDLIL

>B.BR.10.10BR_RJ020.KT427785

SYHRLRDLLL

>B.BR.10.10BR_RJ024.KT427784

SYHLLRDLLL

>B.BR.10.10BR_RJ025.KT427783

SYRHLRDLLL

>B.BR.10.10BR_RJ030.KT427781

SYHRLRDLLL

>B.BR.10.10BR_RJ032.KJ849801

SYHRLRDLLL

>B.BR.10.10BR_RJ033_2.KT427656

SYRHLRDLLL

>B.BR.10.10BR_RJ040.KT427650

SYHRLRDLLL

>B.BR.10.10BR_RJ042.KT427777

SYHRLRDLLL

>B.BR.10.10BR_RJ050.KJ849811

SYHRLRDLLL

>B.BR.10.10BR_RJ051.KJ849825

SYHRLRDLLL

>B.BR.10.10BR_RJ052.KT427775

SYHRLRDLLL

>B.BR.10.10BR_RJ054.KJ849814

SYHRLRDLLL

>B.BR.10.10BR_RJ065.KT427866

SYHHLRDLLL

>B.BR.10.10BR_RJ067.KT427771

SYHRLRDLLS

>B.BR.10.10BR_RJ068.KT427770

SYHRLRDLLL

>B.BR.10.10BR_RJ078.KT427769

SYHRLRDLLL

>B.BR.10.10BR_RJ079.KT427768

SYHRLRDLLL

>B.BR.10.10BR_RJ081.KT427767

SYHRLRDLLL

>B.BR.10.10BR_RJ083.KT427766

SYHRLSDLLL

>B.BR.10.10BR_RJ085.KT427765

SYRHLRDLLL

>B.BR.10.10BR_RJ086.KT427764

SYHHLRDFLL

>B.BR.10.10BR_RJ088.KT427763

SYHRLRDLLL

>B.BR.10.10BR_RJ090.KT427761

SYHRLRDLLL

>B.BR.10.10BR_RJ097.KT427757

SYHRLRDLLL

>B.BR.10.10BR_RJ098.KT427756

SYHRLRDLIL

>B.BR.10.10BR_RJ099.KT427755

SYHHLRDLLL

>B.BR.10.10BR_RJ100.KT427754

SYHRLRDLLL

>B.BR.10.10BR_RJ101.KT427753

SYHRLRDLLL

>B.BR.10.10BR_RJ105_2.KT427654

SYHRLRDLLL

>B.BR.10.10BR_RJ106.KT427751

SYHRLRNLLL

>B.BR.10.10BR_RJ107.KT427750

SYRHLRDLLL

>B.BR.10.10BR_RJ110.KT427749

SYHRLRDLLL

>B.BR.10.10BR_RJ111.KT427748

SYHRLRDLLL

>B.BR.10.10BR_SP002.KT427830

SYHRLRDLLS

>B.BR.10.10BR_SP003.KJ849785

SYHRLRDLLL

>B.BR.10.10BR_SP004.KT427829

SYHRLRDLIL

>B.BR.10.10BR_SP006.KT427827

SYHHLRDLLL

>B.BR.10.10BR_SP007.KT427826

SYHHLRDLLL

>B.BR.10.10BR_SP008.KJ849821

SYHLLRDLIL

>B.BR.10.10BR_SP012.KT427824

SYHRLRDLLL

>B.BR.10.10BR_SP014.KJ849790

SYRRLRDLIL

>B.BR.10.10BR_SP019.KT427819

SYHRLRDLLL

>B.BR.10.10BR_SP021.KJ849796

SYRHLRDLLL

>B.BR.10.10BR_SP027.KT427815

SYHRLRDLLL

>B.BR.10.10BR_SP032.KT427811

SYHRWRDFLL

>B.BR.10.10BR_SP036.KT427808

SYHRLRDLLL

>B.BR.10.10BR_SP038.KJ849805

IYHRLRDLLL

>B.BR.10.10BR_SP043.KJ849807

LYHRLSDLLL

>B.BR.10.10BR_SP044.KT427805

SYRHLRDLLL

>B.BR.10.10BR_SP045.KJ849808

SYHQLRNLIL

>B.BR.10.10BR_SP047.KT427804

SYHRLRDLLL

>B.BR.10.10BR_SP050.KJ849812

SYHRLRDLLL

>B.BR.10.10BR_SP051.KT427803

SYHRLRDLLL

>B.BR.10.10BR_SP052.KT427802

SYHRLRDLLL

>B.BR.10.10BR_SP055.KJ849815

SYHRLRDLLL

>B.BR.10.10BR_SP062.KT427799

SYHRLRDLLS

>B.BR.10.10BR_SP065.KT427797

LYHRLRDLLL

>B.BR.10.10BR_SP067.KT427796

SYHHLRDLLL

>B.BR.10.10BR_SP073_2.KT427667

SYRHLRDLLL

>B.BR.10.10BR_SP074.KT427794

SYHRLRDLLL

>B.BR.10.DEMB10BR038.KU749390

IYHRLRDLLL

>B.BR.11.2011BRRJNEUT26.KX181912

SYHRLRDLLL

>B.BR.11.2011BRRJNEUT4.KX181894

SYHRLRDLLL

>B.BR.11.2011BRRJNEUT6.KX181896

SYRHLRDLLL

>B.BR.12.2012BRRJNEUT17.KX181905

SYRRLRDLLL

>B.BR.12.2012BRRJNEUT20.KX181906

SYRLLRDFLL

>B.BR.12.2012BRRJNEUT23.KX181909

SYHRLRDLLL

>B.BR.13.2013BRRJNEUT10.KX181900

SYHRLRDLLL

>B.BR.13.2013BRRJNEUT11.KX181901

SYHHLRDLLL

>B.BR.13.2013BRRJNEUT13.KX181903

SYRHLRDLLL

>B.BR.13.2013BRRJNEUT1.KX181891

LYRHLRDLLL

>B.BR.13.2013BRRJNEUT21.KX181907

SYHRLRDLLL

>B.BR.13.2013BRRJNEUT22.KX181908

SYHLLRDLLL

>B.BR.13.2013BRRJNEUT24.KX181910

SYHQLRDLIL

>B.BR.13.2013BRRJNEUT25.KX181911

SYHHLRDLLL

>B.BR.13.2013BRRJNEUT28.KX181914

SYHRLRDLLL

>B.BR.13.2013BRRJNEUT5.KX181895

SYHRLRDLLL

>B.BR.13.2013BRRJNEUT7.KX181897

SYHHLRDSLL

>B.BR.13.2013BRRJNEUT8.KX181898

SYHRLSDLLL

>B.BR.14.2014BRRJNEUT9.KX181899

SYHRLTDLIL

>B.BR.15.2015BRRJNEUT27.KX181913

SYHRLRDLLL

>B.BR.15.2015BRRJNEUT29.KX181915

SYHHLRDLLL

>B.BR.15.2015BRRJNEUT30.KX181916

SYHRLRDLLL

>B.BR.16.HI2016_02.MG571981

SYHRLSDLLL

>B.BR.16.HI2016_03.MG571982

SYHRLRDLLL

>B.BR.16.HI2016_04.MG571983

SYHRLRDLLL

>B.BR.16.HI2016_12.MG571987

SYRHLRDLLL

>B.BR.16.HI2016_14.MG571990

SYHRLRDLLL

>B.BR.16.HI2016_15.MG571991

SYHRLRDLLL

>B.BR.16.HI2016_19.MG571996

SYRHLRDLLL

>B.BR.16.HI2016_21.MG571999

SYHRLRDLLL

>B.BR.16.HI2016_22.MG572001

SYHSLRDLLL

>B.BR.16.HI2016_23.MG572002

SYHHLRDLLL

>B.BR.16.HI2016_27.MG572006

SYHRLRDLLL

>B.BR.16.HI2016_31.MG572010

SYHRLRDLLL

>B.BR.89.BZ167.AY173956

SYHRLRDLLL

>B.CA.00.CANA6FULL.AY779552

SYHRLRDLLL

>B.CA.03.HDNDRPI032B2.GU562135

SYHRLRDLLL

>B.CA.03.PSL024B10.GU562272

SYHHLRDLLL

>B.CA.04.HDNDRPI034A4.GU562155

LYHRLRDLLL

>B.CA.05.P6_DAY_1.FJ653506

SYHRLSDLLL

>B.CA.06.502_1027_wg01.JF320413

SYHHLRDLLL

>B.CA.06.502_1799_FL02.JF320427

SYHRLRDLLL

>B.CA.06.HTM385B7.GU562266

SYHRLRDLLL

>B.CA.07.502_1191_03.JF320424

LYHRLRDLLL

>B.CA.96.WC10C_10.AY314061

IYHCLRDLLL

>B.CA.97.ACTDM580208A15.GU562033

SYRRLRDLLL

>B.CA.97.CQLDR03A1.GU562058

SYHHLRDLLL

>B.CA.97.HTM319C1.GU562236

SYHRLRDLLL

>B.CA.98.ACT54869022A2.GU562001

SYHRLRDLLL

>B.CA.98.HDNDRPI001B10.GU562080

SYHRLRDLLL

>B.CA.x.GOL016V01.DQ322223

SYHRLRDLLL

>B.CA.x.HDM003V01.DQ322227

SYHRLTDLLL

>B.CA.x.HND_DRPI039V01.DQ322225

SYHRLRDLLL

>B.CA.x.HTM360V05_variant_3.DQ322239

SYRRLRDLLS

>B.CH.00.HIV_CH_BID_V3529_2000.JQ403022

SYHHLRDLLL

>B.CH.00.HIV_CH_BID_V3530_2000.JQ403023

SYHRLRDLIL

>B.CH.00.M1_0007251_NFLG95.KC797171

SYHHLRDLLL

>B.CH.01.HIV_CH_BID_V3531_2001.JQ403024

SYRRLRDLLL

>B.CH.01.HIV_CH_BID_V3533_2001.JQ403025

SYHRLRDLLL

>B.CH.01.HIV_CH_BID_V3534_2001.JQ403026

SYHRLRDLLL

>B.CH.01.NAB1pre_cl_12.EU023916

LYHRLRDLLL

>B.CH.02.HIV_CH_BID_V3511_2002.JQ403019

SYHRLRDLLL

>B.CH.02.HIV_CH_BID_V3527_2002.JQ403021

SYHRLRDLLL

>B.CH.02.HIV_CH_BID_V3539_2002.JQ403029

SYHRLIDLLS

>B.CH.02.HIV_CH_BID_V4404_2002.JQ403041

SYHHLRDLLL

>B.CH.02.HIV_CH_BID_V4424_2002.JQ403044

SYHRLRDLLL

>B.CH.02.HIV_CH_BID_V4478_2002.JQ403047

SYHRLRDLLL

>B.CH.02.T1SHCS901cl_D2_T1.KX792621

SYHRLTDLLL

>B.CH.03.HIV_CH_BID_V4421_2003.JQ403043

SYHRLRDLLL

>B.CH.03.HIV_CH_BID_V4470_2003.JQ403045

SYHRLRDLLL

>B.CH.03.HIV_CH_BID_V4474_2003.JQ403046

SYRHLRDLLL

>B.CH.03.NAB2pre_cl_3.EU023919

GYHRLRDLLL

>B.CH.03.NAB3pre_cl_43.EU023921

SYRHLRDILL

>B.CH.03.NAb4pre_cl_1.EU023922

SYHRLRDLLL

>B.CH.03.R9ZPHI39SGA_1_R9.KX792557

SYHRLRDLLL

>B.CH.03.T2SHCS396SGA_11_T2.KX792831

IYHRLRDLLL

>B.CH.04.HIV_CH_BID_V4408_2004.JQ403042

SYRHLRDLLL

>B.CH.06.T3SHCS566SGA_17_T3.KX792664

SYHRLRDLLL

>B.CH.06.T5ZPHI67SGA_11_T5.KX792688

SYHRLRDLIL

>B.CH.07.T4SHCS617SGA_13_T4.KX792771

SYHRLRDLLL

>B.CH.07.T6SHCS604SGA_10_T6.KX792725

SYRRLRDLLL

>B.CH.08.M2_0803101_NFLG8.KC797225

SYHRLRDLLL

>B.CH.08.T7ZPHI80SGA_1_T7.KX792808

SYHRLRDLLL

>B.CH.x.NAB10pre_cl_2.EU023929

SYHHLRDLLL

>B.CH.x.NAB11pre_cl_18.EU023932

SYHHLRNLLL

>B.CH.x.NAB12pre_cl_7.EU023933

SYHRLRDLLL

>B.CH.x.NAB8pre_cl_11.EU023927

SYHLLRDLLL

>B.CH.x.R11_8.1_cl57.MH714351

SYHRLRDLLL

>B.CH.x.R282_3.4_cl56.MH714324

SYHRLRDLLL

>B.CH.x.T11_10.6_cl17.MH714343

SYHRLRDLLS

>B.CH.x.T294_3.8_cl28.MH714329

SYHRLRDLLL

>B.CH.x.ZEnv07_0504_15.KU600814

SYHRLRDLLL

>B.CH.x.ZEnv16_1202_7.KU600818

SYHRLGDLLL

>B.CH.x.ZEnv91_0505_12.KU600815

SYHRLRDLLL

>B.CN.01.CNHN24.AY180905

SYHRLRDLLW

>B.CN.03.B01.EU363825

SYHHLRDLLL

>B.CN.03.B03.EU363827

SYHHLRDLLL

>B.CN.03.CHNHLJBSF03009c34.AY905493

NYHRLRDLLL

>B.CN.03.SHXDC0081.JF932492

SYHNLRDLLL

>B.CN.04.CHNHLJBF04016c4.AY905497

SYHRLRDLLL

>B.CN.04.CHNHLJBF04029c2.AY905495

SYHRLRDLLL

>B.CN.04.CHNHLJSM04028c3.AY905494

SYHRLRDLLL

>B.CN.04.CNE10.HM215397

SYHRLRDLLL

>B.CN.04.CNE63.HQ699978

SYHRLRDLLL

>B.CN.04.CNE83_U.HQ699990

SYHRLRDLLL

>B.CN.04.CNE99_U.HQ699996

SYHHLRDLLS

>B.CN.04.CNHLJBF04019.EU131792

SYHHLRDLLL

>B.CN.04.CNHLJBF04021.EU131801

SYHRLRDLLL

>B.CN.04.CNHLJBF04023.EU131797

SYHHLRDLLL

>B.CN.04.CNHLJBF04024.EU131791

SYHRLRDLLL

>B.CN.04.CNHLJBF04025.EU131790

SYHHLRDLLL

>B.CN.04.CNHLJBF04026.EU131788

SYHHLRDLLL

>B.CN.04.CNHLJBF04030.EU131808

SYRHLRDLLL

>B.CN.04.CNHLJBM04020.EU131789

SYHRLRDLLL

>B.CN.05.05071.MK053935

SYHLLRDFLL

>B.CN.05.05CNHB_hp3.DQ990880

SYHRLRDLLL

>B.CN.05.CNE57.HM215420

SYHHLRDLLL

>B.CN.05.CNE95_U.HQ699995

SYHRLRDLLL

>B.CN.05.CNE9.HM215428

SYHRLRDLLL

>B.CN.05.CNHLJBM05036.EU131803

SYHRLRDLLL

>B.CN.06.B04.EU363828

SYHRLRDLLL

>B.CN.06.B05.EU363829

SYHRLRDLLL

>B.CN.06.CBJC515_20060418_14.MF591612

SYRRLRDLLL

>B.CN.06.CC056.JF932482

SYHHLRDLLL

>B.CN.06.CNE1.HQ699949

SYRRLRDLLL

>B.CN.06.CNE4.HM215413

SYHLSRDSLL

>B.CN.06.CNE6.HM215423

SYHRLRDLLL

>B.CN.06.CNHLJBF06044.EU131805

SYHRLRDLLL

>B.CN.06.CNHLJBF06051.EU131802

SYHRLRDLLL

>B.CN.06.CNHLJBM06050.EU131798

SYHRLRDLLL

>B.CN.06.CNHLJBM06057.EU131800

SYHRLRDLLL

>B.CN.06.CNHLJSF06056.EU131804

SYHRLRDLLL

>B.CN.06.CNHLJSF06060.EU131810

SYHHLRDLLL

>B.CN.06.CNHLJSM06048.EU131794

SYHLLRDLLL

>B.CN.06.CNHLJ_M06053.EU131807

SYHRLRDLLL

>B.CN.06.CNHLJ_M06058.EU131809

SYHHLRDLLL

>B.CN.07.AH070011.JF932468

SYHQLRDLLL

>B.CN.07.AH070014.JF932469

SYHRLRDLLL

>B.CN.07.AH070017.JF932470

SYHRLRDLLL

>B.CN.07.AH070018.JF932471

SYHHLRDLLL

>B.CN.07.AH070057.JF932472

SYHRLRDLLL

>B.CN.07.BJ070030.JF932473

SYRHLRNLLL

>B.CN.07.BJOX003000.e02.KM217584

SYHRLSDLLS

>B.CN.07.BJOX006000.e05.KM217662

SYHRLRDLLL

>B.CN.07.BJOX014000.e37.KM217802

IYHRLRDLLL

>B.CN.07.BJOX022000.e02.KM217995

SYHRLSDLLS

>B.CN.07.CBJC261.JF932474

SYRHLRDLLL

>B.CN.07.CBJC392.JF932475

SYHRLRDLLL

>B.CN.07.CBJC394.JF932476

SYHRLRDLLL

>B.CN.07.CBJC396.JF932477

SYHRLRDLLL

>B.CN.07.FJ070016.JF932483

SYLRLRDLLL

>B.CN.07.GS070017.JF932484

IYHRLRDLLL

>B.CN.07.GZ070002.JF932485

SYHHLRDLLL

>B.CN.07.GZ070030.JF932486

SYHRLRDLLL

>B.CN.07.HB070006.JF932487

SYHRLRDLLL

>B.CN.07.HB070022.JF932488

SYHRLRDLLW

>B.CN.07.HB070035.JF932489

SYHRLRDLLL

>B.CN.07.JL070038.JF932490

SYHRLRDLLS

>B.CN.07.JS070389.JF932491

SYHLLRDLLL

>B.CN.07.SX070080.JF932493

SYHRLRDLLL

>B.CN.07.hb070025.JF932499

SYHRLRDLLL

>B.CN.07.hen1345.JF932500

SYHRLRDLLL

>B.CN.08.1106.HQ215554

SYHRLRDLLL

>B.CN.08.BJOX035000.e01.KM218138

IYHRLRDLLL

>B.CN.08.BJOX041000.e10.KM218168

SYRRLSDLLL

>B.CN.08.BJOX046000.e14.KM218195

SYHRLSDLLL

>B.CN.08.BJOX047000.e19.KM218228

CYHRLSDLLS

>B.CN.08.CBJC476.JF932478

SYHHLRDLLL

>B.CN.08.CBJC489.JF932479

SYHHLRDLLL

>B.CN.08.CBJC500.JF932480

SYHRLRDLLL

>B.CN.08.CBJC502.JF346917

SYHRLRDLLL

>B.CN.08.CBJC503.JF932481

SYHRLRDLLL

>B.CN.08.CBJC507.JF346918

SYRHLRDLLL

>B.CN.08.CBJC513.JF346919

SYHRLRDLLL

>B.CN.08.cbjc468.JF932498

SYHLLRDLLL

>B.CN.09.09LNA014.JX960597

SYHRLRDLLL

>B.CN.09.09LNA336.JX960599

IYHRSRDLLL

>B.CN.09.09LNA439.JX960598

IYHRLRDLLL

>B.CN.09.09YNRL215042sg.KC899011

SYHRLRDLLL

>B.CN.09.1121.HQ215556

LYHRLRDLLL

>B.CN.09.CBJC485.JF346916

SYHRLRDLLL

>B.CN.09.DEMB09CN002.KC596066

CYHRLSDLLS

>B.CN.09.YN09P0014.JF932494

SYHRLRDLLL

>B.CN.09.ZK042.JF932497

SYHRLRDLLL

>B.CN.10.DEMB10CN002.JX140658

SYQRLRDLLL

>B.CN.12.134_0_27.KX692916

SYHRLRDLLL

>B.CN.12.15_0_17.KX692929

SYHRLRDLLL

>B.CN.12.2019_0_2.KX692941

SYHRLRDLLL

>B.CN.12.2039_2_13.KX693556

SYHSLRDLLL

>B.CN.12.2079_17.KX692973

SYHLLRDLLL

>B.CN.12.2081_10.KX692981

SYHRLRDLLL

>B.CN.12.2092_0_13.KX693001

SYHRLRDLLL

>B.CN.12.2097_0_16.KX693029

SYHHLRDLLL

>B.CN.12.2110_10.KX693036

SYHRLRDLLL

>B.CN.12.2124_3_35.KX693575

SYHRLRDLLL

>B.CN.12.2142_0_13.KX693068

SYHRLRDLLL

>B.CN.12.2157_8.KX693091

SYHRLRDLLL

>B.CN.12.2162_3.KX693093

SYHHLRDLLL

>B.CN.12.2243_1_7.KX693642

SYHQLRDFLL

>B.CN.12.2350_0_10.KX693114

SYHRLRDLLL

>B.CN.12.2357_18.KX693136

SYHLLRDLLL

>B.CN.12.2358_2_16.KX693689

SYHRLRDLLL

>B.CN.12.2360_2_93.KX693720

SYHLLRDLLL

>B.CN.12.2361_0_1.KX693171

SYHHLRDLLL

>B.CN.12.2362_2_19.KX693770

SYHRLRDLLL

>B.CN.12.2363_21.KX693223

SYHRLSDLLL

>B.CN.12.2406_12.KX693238

SYHRLRDLLL

>B.CN.12.2408_0_1.KX693247

SYHHLRDLLL

>B.CN.12.2418_0_18.KX693268

SYHHLRDLLL

>B.CN.12.2460_26.KX693292

SYHRLRDLLL

>B.CN.12.3124_1_18.KX693886

SYRRLSDLLW

>B.CN.12.3559_2.KX693309

SYRHLRDLLL

>B.CN.12.3560_1.KX693314

SYHHLRDLLL

>B.CN.12.3581_19.KX693326

SYHRLRDLLL

>B.CN.12.3596_10.KX693339

SYHRLRDLLL

>B.CN.12.3617_4.KX693362

SYHRLRDLLL

>B.CN.12.3624_20.KX693374

SYHRLRDLLL

>B.CN.12.3627_4.KX693390

SYHLLRDLLL

>B.CN.12.3786_7.KX693411

SYHRLRDLLL

>B.CN.12.3832_20.KX693437

SYHLLRDSLL

>B.CN.12.3841_12.KX693455

SYLRLRDLLL

>B.CN.12.3856_10.KX693469

SYHHLRDFLL

>B.CN.12.3858_11.KX693475

SYHRLRDLLL

>B.CN.12.3879_2.KX693489

SYHHLRDLLL

>B.CN.12.3899_2.KX693491

SYHRLRDLLL

>B.CN.12.3954_0_1.KX693496

SYHRLRDLLL

>B.CN.12.555_0_8.KX693539

SYHHLRDLLL

>B.CN.12.DEMB12CN006.KP109511

SYHRLNDLLS

>B.CN.12.DEMB12CN010.KP109512

SYHRLRDLLL

>B.CN.13.BJMP3116B.KU724103

SYRHLRDLLL

>B.CN.13.BJMP3294B.KU724105

SYHRLSDLLS

>B.CN.98.YN9802.JF932495

SYHLLRDSLL

>B.CN.98.YN9838.JF932496

SYHRLRDLLL

>B.CN.99.plwj11_6.GU647196

IYHRLRDLLL

>B.CN.x.B06.EU363830

SYHRLRDLLL

>B.CO.01.PCM001.AY561236

SYHLLRDSIL

>B.CO.01.PCM013.AY561237

SYHRLRDLLL

>B.CO.01.PCM034.AY561238

SYRHLRDLLL

>B.CO.01.PCM039.AY561239

VYHRLRDLLL

>B.CO.01.PCM074.AY561240

SYHRLRDLLL

>B.CO.06.subject_247_day1_clone_15.HQ377377

SYHRLRDLLL

>B.CU.12.12CU087.KR914675

SYHRLRDLLL

>B.CU.14.14CU005.KR914676

SYHRLRDLLS

>B.CU.14.14CU007.KR914678

SYHRLRDLLL

>B.CU.99.Cu19.AY586542

SYHRLRDLLL

>B.CU.99.Cu43.AY586543

SYHRLRDLLL

>B.CY.03.CY005.EU668963

SYHRLRDLLL

>B.CY.05.CY018.FJ388890

SYHRLRDLLL

>B.CY.05.CY020.FJ388891

SYHRLRDLLL

>B.CY.05.CY028.FJ388895

SYHRLRDLLL

>B.CY.05.CY030.FJ388956

SYHRLRDLLL

>B.CY.05.CY032.FJ388898

SYHRLRDLLL

>B.CY.05.CY035.FJ388958

SYHRLRDLLS

>B.CY.05.CY037.FJ388899

SYHRLRDLLL

>B.CY.05.CY055.FJ388904

SYRRLTDLLS

>B.CY.05.CY056.FJ388905

SYHRLRDLLL

>B.CY.05.CY065.FJ388910

SYHRLRDLLL

>B.CY.05.CY067.FJ388911

SYHRLRDLLL

>B.CY.05.CY068.FJ388912

LYHRLRDLLL

>B.CY.05.CY070.FJ388960

SYHHLRDLLL

>B.CY.05.CY072.FJ388914

SYHRLRDLLL

>B.CY.05.CY074.FJ388915

LYHRLRDLLL

>B.CY.05.CY075.FJ388916

SYRRLRDLLS

>B.CY.05.CY082.FJ388962

SYHRLRDLLL

>B.CY.05.CY087.FJ388918

SYHRLRDLLL

>B.CY.05.CY089.FJ388920

SYHRLRDLLL

>B.CY.05.CY093.FJ388923

SYHRLRDLLL

>B.CY.05.CY096.FJ388963

SYHRLRDLLL

>B.CY.05.CY097.FJ388924

SYHRLRDLLL

>B.CY.05.CY100.FJ388964

SYHRLKDLLL

>B.CY.05.CY110.FJ388927

SYHRLRDLLL

>B.CY.05.CY113.FJ388930

SYHRLRDLIL

>B.CY.05.CY122.FJ403482

SYHRLRDLLL

>B.CY.05.CY124.FJ388933

SYRRLRDLLL

>B.CY.05.CY131.FJ388935

SYHRLRDLLS

>B.CY.05.CY132.FJ388936

SYHHLRDLLL

>B.CY.05.CY137.FJ388937

SYHRLRDLLL

>B.CY.05.CY141.FJ388939

LYRRLTDLLL

>B.CY.05.CY142.FJ388965

SYHRLRDLLL

>B.CY.05.CY149.FJ388940

SYHRLRDLLL

>B.CY.06.CY168.FJ388949

SYHRLRDLLL

>B.CY.06.CY180.FJ388955

SYHRLRDLLS

>B.CY.07.CY181.JF683736

LYRHLRDLLL

>B.CY.07.CY184.JF683738

SYRRLRDCLL

>B.CY.07.CY188.JF683741

SYHRLRDLLL

>B.CY.07.CY189.JF683742

SYHRLRDLLL

>B.CY.07.CY190.JF683743

SYHRLRDLLS

>B.CY.07.CY195.JF683747

SYHRLRDLLL

>B.CY.07.CY197.JF683749

SYHRLRDLLS

>B.CY.07.CY198.JF683750

SYHRLRDLLL

>B.CY.07.CY199.JF683751

SYRHLRDLLL

>B.CY.07.CY201.JF683753

SYPLLRDFLL

>B.CY.07.CY202.JF683754

SYHRLRDLLL

>B.CY.07.CY204.JF683756

SYHRLRDLLL

>B.CY.07.CY214.JF683764

SYHRLRDLLL

>B.CY.07.CY216.JF683765

LYHRLRDLLL

>B.CY.08.CY220.JF683769

SYHRLRDLLL

>B.CY.08.CY224.JF683773

SYHHLRDLLL

>B.CY.08.CY226.JF683775

LYHRLRDLLL

>B.CY.08.CY229.JF683778

SYHHLRDLLL

>B.CY.08.CY232.JF683781

SYHRLRDLLL

>B.CY.08.CY237.JF683784

SYHRLGDLLL

>B.CY.08.CY238.JF683785

IYRHMRDLLL

>B.CY.09.CY241.JF683787

SYHRLRDLIL

>B.CY.09.CY242.JF683788

SYLRLRVLLL

>B.CY.09.CY244.JF683790

SYHRLRDLLL

>B.CY.09.CY250.JF683793

CYHRLRDLLL

>B.CY.09.CY251.JF683794

SYHHLRDLLL

>B.CY.09.CY253.JF683796

SYHHLRDLLL

>B.CY.09.CY254.JF683797

IYHRLRDLLL

>B.CY.09.CY258.JF683801

SYHRLRDLLL

>B.CY.09.CY262.JF683804

SYHRLRDLLL

>B.CY.09.CY263.JF683805

SYHRLRDLLL

>B.CY.09.CY266.JF683807

LYHRLIDLLS

>B.DE.03.HIV_DE_BID_V3307_2003.JQ403048

SYHHLRDLLL

>B.DE.04.9213_d0.JQ416158

SYHRLTDLLL

>B.DE.04.963987.KT124812

SYHRLRDLLL

>B.DE.04.HIV_DE_BID_V4131_2004.JQ403037

SYRHLRDLLL

>B.DE.07.906153.KT124803

SYHRLRDLLL

>B.DE.08.147984.KT124751

SYHRLRDLLL

>B.DE.08.154162.KT124752

SYHRLRDLLL

>B.DE.08.176952.KT124755

SYRHLRDLLL

>B.DE.08.255524.KT124757

SYHRLRDLSL

>B.DE.08.296004.KT124762

SYRRLTDLLL

>B.DE.08.637829.KT124788

SYHRLRDLLL

>B.DE.08.654207.KT124789

SYHHLRDLLL

>B.DE.08.822582.KT124794

LYHRLTDLLL

>B.DE.08.882283.KT124800

SYHHLRDLLL

>B.DE.08.954229.KT124810

SYHLLRDLLL

>B.DE.09.136172.KT124749

SYRRLIDLLL

>B.DE.09.159793.KT124753

FYHRLRDLLL

>B.DE.09.172508.KT124754

LYHRLRDLLL

>B.DE.09.585067.KT124782

SYRHLRDLLL

>B.DE.09.923040.KT124805

SYHRLRDLLL

>B.DE.09.C7V00477_E10_116057.KC247954

SYHHLRDLLL

>B.DE.09.C7V00485_E10_116061.KC247955

SYHRLRDLLL

>B.DE.09.C7V00493_E10_116085.KC247957

SYHRLRDLLL

>B.DE.10.290307.KT124760

SYHRLRDLLL

>B.DE.10.320435.KT124763

SYHRLRDLLL

>B.DE.10.464704.KT124772

SYRHLRDLLL

>B.DE.10.556743.KT124779

SYHRLRDLLL

>B.DE.10.571373.KT124780

SYHRLRDLLS

>B.DE.10.587196.KT124783

SYHRLRDLLL

>B.DE.10.863847.KT124797

LYHRLRDLLL

>B.DE.10.C7V004G9_E10_116111.KC247963

SYHRLRDLLL

>B.DE.10.C7V006H2_E10_152257.KC248026

SYHRLRDLLL

>B.DE.10.C7V00J67_E10_152238.KC248011

SYHRLRDLXX

>B.DE.10.C7V00J75_E10_152239.KC248012

SYHRLRDLLL

>B.DE.10.C7V00QP8_E10_131969.KC247978

SYRRLRDLLL

>B.DE.10.C7V00QX2_E10_152240.KC248013

SYRRLTDLLL

>B.DE.10.C7V00QY0_E10_152242.KC248015

SYHRLRDLLL

>B.DE.10.C7V00R34_E10_152244.KC248017

SYHRLRDLLL

>B.DE.10.C7V00ZJ2_E10_131972.KC247981

SYHRLRDLLL

>B.DE.10.C7V00ZR5_E10_131974.KC247983

SYHRLRDLLL

>B.DE.10.C7V00ZS3_E10_131975.KC247984

SYHRLRDLLL

>B.DE.10.C7V00ZT1_E10_131976.KC247985

SYHRLIDFLL

>B.DE.10.C7V00ZV7_E10_131978.KC247987

SYHRLRDLLX

>B.DE.10.C7V01AF1_E10_131980.KC247989

SYHRLRDLLL

>B.DE.10.C7V01AK1_E10_131983.KC247992

SYHRLRDLLS

>B.DE.10.C7V01AL9_E10_131985.KC247993

XYHRLRDLLL

>B.DE.10.C7V01AM7_E10_131986.KC247994

LYHRLRDSLL

>B.DE.10.C7V01B31_E10_131993.KC247997

LYHRLRDLLL

>B.DE.10.C7V01C48_E10_152265.KC248034

SYHRLRDLLL

>B.DE.10.iso4_w10.KU612900

SYRRLRDLLL

>B.DE.10.iso5_h10.KU612901

SYHRLRDLLL

>B.DE.11.891676.KT124802

SYHRLRDLLL

>B.DE.12.328893.KT124765

SYHRLRDLLS

>B.DE.12.635056.KT124787

SYRRLRDLLL

>B.DE.12.956306.KT124811

SYHHLRDLLL

>B.DE.13.366396.KT124767

LYHRLRDLLL

>B.DE.13.947915.KT124808

IYHRLTDLLS

>B.DE.16.1HD10K_W4_090516_B8_S15.KY324301

SYHRLRDLLL

>B.DE.16.1HD11K_W4_101416_F11_S16.KY324379

SYRHLRDLLL

>B.DE.16.1HD4K_W4_062316Pl4_E2_S52.KY324591

SYHRLRDLLL

>B.DE.16.1HD5K_W4_061616_G9_S52.KY324630

SYHRLRDLLL

>B.DE.16.1HD6K_D0_061016_A9_S8.KY324641

NYHLLRDLLL

>B.DE.16.1HD8K_D0_062916_C9_S77.KY324761

SYRRLRDLLL

>B.DE.16.1HD9K_D0_070816_B5_S32.KY324808

SYRHLRDLLL

>B.DE.86.D117III_child.AF490512

SYHRLRDLLL

>B.DE.86.D31.U43096

LYHRLRDLLL

>B.DE.86.HAN.U43141

SYRRLRDLLL

>B.DE.x.DEMBXXDE001.KC596067

SYHRLRDLLL

>B.DK.01.CTL_016.EF514704

SYHHLRDLLL

>B.DK.01.CTL_017.EF514705

SYHRLRDLLS

>B.DK.01.CTL_018.EF514706

SYHRLRDLLL

>B.DK.01.CTL_023.EF514707

SYHRLRDLLL

>B.DK.01.CTL_030.EF514708

SYHRLRDLLL

>B.DK.01.CTL_033.EF514709

SYRHLRDLLL

>B.DK.01.CTL_035.EF514710

SYHRLRDLLL

>B.DK.01.CTL_041.EF514711

SYHRLRDLLL

>B.DK.01.CTL_043.EF514712

SYHRLRDLLL

>B.DK.04.PMVL_012.EF514699

SYHRLRDLLL

>B.DK.04.PMVL_013.EF514700

SYHHLRDLLL

>B.DK.04.PMVL_018.EF514697

SYHRLRDLLL

>B.DK.04.PMVL_025.EF514702

SYHRLRDLLL

>B.DK.04.PMVL_027.EF514698

SYRRLRDLLL

>B.DK.04.PMVL_039.EF514703

SYHRLRDLLL

>B.DK.04.PMVL_049.EF514701

IYHRLRDLLL

>B.DK.07.PMVL_011.FJ694790

SYRHLRDLLL

>B.DO.05.05DO_160884.EU839597

SYHRLSDLLL

>B.DO.05.05DO_162387.EU839596

SYHRLRDLLL

>B.DO.05.05DO_163007.EU839598

SYHRLRDLLL

>B.DO.08.X2348_c2_8.FJ817365

LYHRLRDLLL

>B.DO.11.DEMB11DR001.KY658702

SYHRLRDLLL

>B.EC.89.EC003.AY173959

SYHRLRDLLL

>B.EC.89.EC102.AY173960

SYRHLRDLLL

>B.ES.04.12_3_PC_1.KC595150

SYHRLRDLLL

>B.ES.04.18122_3Fc1.KC595152

SYHRLRDLLL

>B.ES.04.20020760_Fc1.KC595154

SYHRLSDLLL

>B.ES.04.251248_7Fc1.KC595157

SYQHLRDLLL

>B.ES.04.3227050_2Fc1.KC595160

SYHRLRDLLL

>B.ES.04.3227057_5Fc1.KC595161

SYHRLRDLLL

>B.ES.04.3227058_6Fc1.KC595162

SYHRLRDLLL

>B.ES.04.357182_1Fc1.KC595163

SYHRLRDLLL

>B.ES.04.357184_1Fc1.KC595164

SYHRLRDLLL

>B.ES.04.40022834_5Fc1.KC595165

SYHRLRDLIL

>B.ES.04.5009564_2Fc1.KC595166

IYHRLRDLLL

>B.ES.04.RF_LTNP_09.KC595194

SYHRLRDLLL

>B.ES.04.RF_LTNP_13.KC595197

SYHRLRDLIL

>B.ES.04.RF_LTNP_37.KC595202

SYHRLRDFLL

>B.ES.04.RF_LTNP_40.KC595203

SYHRLRDLLL

>B.ES.05.1540906_2Fc1.KC595151

SYLRLRDLLL

>B.ES.05.279752_5Fc1.KC595158

SYHRLRDLLL

>B.ES.05.9684_12Fc1.KC595168

SYHRLGDLLL

>B.ES.05.MDM6_4gp160.KC595190

SYHRLRDLLL

>B.ES.05.R15.KT200351

SYRRLSDLLL

>B.ES.05.X1890.EU786672

IYHRLRDLLL

>B.ES.06.R14.KT200350

SYHRLRDLLL

>B.ES.06.X1958.EU786674

SYHRLRDLLL

>B.ES.06.X1959.EU786675

SYHRLRDLLL

>B.ES.06.X1998.EU786676

LYHRLRDLLL

>B.ES.06.X2102.EU786677

SYHRLRDLLS

>B.ES.07.RF_LTNP_12.KC595196

SYHRLRDLLL

>B.ES.07.X2149.EU786678

SYRHLRDLLL

>B.ES.07.X2210_3.EU786679

SYHRLRDLLL

>B.ES.07.X2231.EU786680

SYHRLRDLLS

>B.ES.07.X2236_C6.HQ236609

SYHRLRDLLL

>B.ES.07.X2278_c2_B6.FJ817366

SYHRLRDLLL

>B.ES.08.ES_X2515_3.GQ372988

SYHRLRDLLL

>B.ES.08.ES_X2556_3.GQ372990

SYHRLRDLVL

>B.ES.08.P2008.FJ670531

SYHRLRDLLL

>B.ES.08.P2021_3.FJ853620

SYHRLSDLLL

>B.ES.08.R11.KT200349

SYHRLRDLLL

>B.ES.08.R5.KT200354

SYHRLRDLLL

>B.ES.08.R9.KT200358

IYHRLRDLLL

>B.ES.08.X2425_2.FJ670525

SYHRLRDLLL

>B.ES.08.X2510_2.FJ853622

SYHLLRDLLF

>B.ES.08.X2555_2.GU362883

SYHRLIDFLS

>B.ES.08.X2574_2.GU362886

SYHRLTDLLL

>B.ES.09.DEMB09BO001.JX140656

SYHRLRDLLL

>B.ES.09.DEMB09ES007.KC473841

SYHRLRDLLS

>B.ES.09.Leg3.JN054257

SYHHLRDLLL

>B.ES.09.P2149_3.GU362881

SYHHLRDLLL

>B.ES.09.X2689_2.GU362885

SYHRLRDLLL

>B.ES.09.X2730_2_nt0544_9494.MF157736

IYHRLRDLLS

>B.ES.10.DEMB10ES002.KC473842

SYHRLRDLLL

>B.ES.10.R3.KT200352

SYHHLRNLLL

>B.ES.10.R4.KT200353

SYHRLRDLLL

>B.ES.10.R6.KT200355

SYHRLRDLLL

>B.ES.10.R7.KT200356

SYHRLRDLLL

>B.ES.10.R8.KT200357

SYHRLRDLLL

>B.ES.10.X2899_2s_nt0759_9478.MF157735

SYHRLRDLLL

>B.ES.13.DEMB13ES010.KP109518

LYHRLRDLLL

>B.ES.13.P6.KT200348

SYHRLRDLLL

>B.ES.14.100_112.KY465967

SYHRLRDLLS

>B.ES.14.ARP1195.KT276255

SYHRLRDLLL

>B.ES.14.ARP1196.KT276256

LYHRLRDLLL

>B.ES.14.ARP1202.KT276262

SYHRLTDLLL

>B.ES.14.ARP1203.KT276263

SYHRLRDLLL

>B.ES.14.ARP1204.KT276264

SYHRLRDLLL

>B.ES.14.ARP1206.KT276266

SYHHLRDLLL

>B.ES.14.ARP1207.KT276267

SYHRLRDLLL

>B.ES.14.ARP1208.KT276268

SYHRLRDLLL

>B.ES.14.EUR_0031.KU685591

SYHRLRDLLL

>B.ES.14.EUR_0043.KU685583

SYHRLRDLLL

>B.ES.14.EUR_0044.KU685584

SYHRLTDLLL

>B.ES.14.EUR_0045.KU685585

SYHRLRDLLL

>B.ES.14.EUR_0046.KU685586

SYHRLRDLLL

>B.ES.14.EUR_0052.KU685589

SYHRLRDLLL

>B.ES.14.EUR_0053.KU685590

SYHRLRDSLL

>B.ES.15.100594.KY989951

SYHHLRDLLL

>B.ES.15.100596.KY989953

SYHRLRDLLL

>B.ES.15.100597.KY989949

SYHRLSDLLL

>B.ES.15.100598.KY989954

SYHRLRDLLL

>B.ES.15.100600.KY989956

SYHRLRDLLL

>B.ES.15.100_116.KY465969

SYHHLRDLLL

>B.ES.89.As2_3b.KC595171

SYHRLRDLLL

>B.ES.89.As3.KC595172

IYHRLRDLLL

>B.ES.89.As4_2.KC595173

SYHRLTDLLL

>B.ES.89.As5_1b.KC595174

SYHRLRDLLL

>B.ES.89.As8_8.KC595177

SYHRLRDLLL

>B.ES.89.As9_4.KC595178

SYHRLRDLLL

>B.ES.89.RF_LTNP_10.KC595195

SYHRLRDLLL

>B.ES.89.U61.DQ854716

SYHRLRDLLL

>B.ES.98.HC2988965_5Fc1.KC595182

SYHRLRDLLL

>B.ES.99.30_1_21.KC595159

SYHRLRDLLL

>B.ES.x.10246788_1gp160.KC595225

LYHRLRDLLL

>B.ES.x.64.5_8gp160.KC595222

SYHRLRDLLL

>B.ES.x.EC3_180.KC595204

SYHRLRDLLS

>B.ES.x.EC4_1_117.KC595206

SYHRLRDLLS

>B.ES.x.LMP0501.DQ141341

SYHRLRDLLL

>B.ES.x.LMP0502.DQ141342

SYHRLTDLLL

>B.ES.x.LMP0503.DQ141343

SYHRLRDLLL

>B.ES.x.LMP0519.DQ141345

SYHHLRDLLL

>B.ES.x.LMP0608.DQ448819

SYHRLRDLLL

>B.ES.x.MH01.EF531330

SYHRLRDLLL

>B.ES.x.MH02.EF531331

SYHRLRDLLL

>B.ES.x.MH04.EF531333

SYLRLRDLLL

>B.ES.x.P2317_2.JX422195

SYHRLRDLIS

>B.ES.x.X2886_2.JX422209

SYHRLRDLLL

>B.FR.00.309_L_1.AY535455

SYHRLRDLLL

>B.FR.00.310103.KC699018

SYHRLRDLLL

>B.FR.00.750905.KC699022

SYHRLRDLLL

>B.FR.03.LA06ToXa.KU168261

SYHRLRDLLL

>B.FR.05.DEMB05FR001.JX140652

SYHRLRDLLL

>B.FR.06.FHY01223832.MH000288

SYHRLRDLLL

>B.FR.07.590110.KC699029

SYHRLRDLLL

>B.FR.07.590111.KC699030

SYHRLRDLLL

>B.FR.07.940218.KC699040

SYHRLRDLLL

>B.FR.08.330424.KC699028

SYHRLRDLLL

>B.FR.08.750214.KC699032

SYHRLRDLLL

>B.FR.08.940139.KC699038

SYHRLRDLLL

>B.FR.08.940140.KC699039

SYHRLRDLLL

>B.FR.08.DEMB08FR002.JX140654

SYHRLRDLLL

>B.FR.09.770203.KC699035

SYHRLRDLLL

>B.FR.09.840104.KC699036

SYRRLRDLLL

>B.FR.09.920414.KC699037

SYHRLRDLLW

>B.FR.09.DEMB09FR001.KF716494

SYHRLRDLLL

>B.FR.09.DEMB09FR002.KF716495

CYHRLRDLIL

>B.FR.10.660118.KC699031

SYHRLRDLLL

>B.FR.10.751734.KC699034

SYHRLRDLLL

>B.FR.10.BAH74839248.MH000305

SYHRLTDLLS

>B.FR.11.DEMB11FR001.KF716496

SYHRLRDLLS

>B.FR.12.DVB78224816.MH000302

LCHRLRDLLL

>B.FR.12.IWC73075664.MH000292

LYHRLRDLLL

>B.FR.12.OFN23677004.MH000296

SYHRLRDLLL

>B.FR.13.JBF39579748.MH000299

SYRHLRDLLL

>B.FR.88.36.KC699002

SYHRLRDLLL

>B.FR.88.529.KC699006

SYHRLRDLLL

>B.FR.88.562.KC699011

SYHRLRDLLL

>B.FR.89.1058.KC699010

SYHRLRDLLL

>B.FR.89.1197.KC699007

SYHRLRDLLL

>B.FR.89.657.KC699009

SYHRLRDLLL

>B.FR.89.749.KC699008

SYHRLRDLLL

>B.FR.89.757.KC699003

SYHRLRDLLL

>B.FR.89.819.KC699004

SYHRLRDLLL

>B.FR.91.1639.KC699005

SYHRLRDLLL

>B.FR.91.1644.KC699001

SYHRLRDLLL

>B.FR.92.133_1.AY535425

SYHRLRDLLL

>B.FR.92.92FR_BX08.AY713411

SYHRLRDLLL

>B.FR.92.PHI120.AF041125

SYHRLRDLLL

>B.FR.93.153_10.AY535498

SYHRLRDLLL

>B.FR.93.159_10.AY535471

SYRHLRDLLL

>B.FR.93.PHI146.AF041127

GYHRLRDLLL

>B.FR.93.PIH155.AF041130

SYHRLRDLLL

>B.FR.93.PIH160.AF041131

SYHRLRDLLL

>B.FR.95.PIH373.AF041134

SYHRLRDLLS

>B.FR.95.PIH374.AF041133

SYHRLRDLLL

>B.FR.97.130203.KC699014

SYHRLRDLLL

>B.FR.97.440102.KC699016

SYHRLRDLLL

>B.FR.97.440104.KC699017

SYHRLRDLLL

>B.FR.97.60101.KC699012

SYHRLRDLLL

>B.FR.97.751102.KC699024

SYHRLRDLIL

>B.FR.98.750705.KC699020

SYHRLRDLLL

>B.FR.98.920203.KC699026

SYHRLRDLLL

>B.FR.99.130206.KC699015

SYHRLRDLLL

>B.FR.99.60204.KC699013

SYHRLRDLLL

>B.FR.99.750710.KC699021

SYHHLRDLLL

>B.FR.99.751002.KC699023

SYHRLRDLLL

>B.FR.99.751401.KC699025

SYHRLRDLLL

>B.FR.99.P3_1.KF695111

SYHRLRDLLL

>B.FR.x.208.EF033658

LYHRLRDLLL

>B.FR.x.CHA.AF321080

SYHRLRDLLL

>B.GA.88.OYI_397.M26727

SYHRLRDLIL

>B.GB.00.patient_MM4.JN034158

SYHRLRDLLL

>B.GB.01.patient_MM8.JN034139

SYHRLRDLLL

>B.GB.02.MM27_d0032_ipe012_SGA_8.MG902199

LYHRLRDLLL

>B.GB.03.MM33d12p.HM586187

SYHRLRDLLL

>B.GB.04.MM24_d0941_ipe0027_SGA_12.MG902157

SYHRLRDLLL

>B.GB.04.MM39d11p.HM586193

SYHRLRDLLL

>B.GB.04.MM42d22_GN1.HM586198

SYHRLRDLLL

>B.GB.05.MM43d368_GN1.HM586209

SYRRLSDLLL

>B.GB.05.MM45d22_GN1.HM586210

SYHLLRDLLL

>B.GB.05.P1_DAY_1.FJ653437

SYRRLRDLLL

>B.GB.07.UKBH1BD9.JF706474

SYHRLRDLLL

>B.GB.08.B200_B12.HQ595768

SYHRLRDLTL

>B.GB.08.B650c_E2.HQ595771

SYRLLRDFLL

>B.GB.08.B653_C7.HQ595772

LYHRLRDFLL

>B.GB.08.C500_G5.HQ595774

IYHRLRDLLL

>B.GB.08.CH080024_e_p1.HM204626

SYHRLRDLLL

>B.GB.08.CH080038_e_p2.HM204627

SYHRLRDLLL

>B.GB.08.CH080046_e_p1.HM204628

SYHRLRDLLL

>B.GB.08.CH080052_e_p1.HM204629

SYHRLRDLLL

>B.GB.08.CH080060_e_p1.HM204630

SYHRLRDLLL

>B.GB.08.CH080071_e_p2.HM204631

SYHRLRDLLL

>B.GB.08.CH080087_e_p1.HM204632

SYHRLRDLLL

>B.GB.08.CH080095_e_p1.HM204633

SYRHLRDLLL

>B.GB.08.CH080128_e_p1.HM204636

LYHSLRDLLL

>B.GB.08.CH080134_e_p1.HM204637

SYRRLRDLLL

>B.GB.08.CH080142_e_p1.HM204638

SYRRLSDLLL

>B.GB.08.CH080156_e_p1.HM204639

SYHRLRDLLL

>B.GB.08.CH080169_e_p1.HM204640

SYHRLRDLLL

>B.GB.08.CH080175_e_p2.HM204641

SYHHLRDFLL

>B.GB.08.CH080183_e_p1.HM204642

SYHRLRDLLL

>B.GB.08.CH080191_e_p1.HM204643

SYHRLRDLLL

>B.GB.08.CH080203_e_p1.HM204644

SYHRLRDLLL

>B.GB.08.CH080225_e_p2.HM204646

SYHRLRDLLF

>B.GB.08.D650_E9.HQ595775

SYHRLRDLLL

>B.GB.08.DEMB08UK003.KY658697

SYHRLRDLLL

>B.GB.08.E214_E1.HQ595776

LYRRLRDLLL

>B.GB.08.F455b_B4.HQ595778

SYHRLRDLLL

>B.GB.08.G125b_H6.HQ595779

LYHRLRDLLL

>B.GB.08.G230_H7.HQ595780

SYHRLRDLLL

>B.GB.08.G435_G3.HQ595781

SYHRLRDLLL

>B.GB.08.M425_B8.HQ595788

SYHRLRDLLL

>B.GB.08.M535_A7.HQ595790

SYHRLRDLLL

>B.GB.08.P362_E1.HQ595792

SYHRLRDLLL

>B.GB.08.Q230_F6.HQ595794

SYHRLRDLLL

>B.GB.08.R232_F6.HQ595795

SYHRLRDLLL

>B.GB.08.S230_A12.HQ595798

LYHRLRDLLL

>B.GB.08.T460_C6.HQ595803

SYRRLRDLLL

>B.GB.09.A626_p1.JF680906

SYHHLRDLLL

>B.GB.09.B400_G4.HQ595769

SYHHLRDLIL

>B.GB.09.B520_A3.HQ595770

LYRHLRDLLL

>B.GB.09.C600_p1.JF680909

SYHRLRDLLL

>B.GB.09.C625_p1.JF680910

SYHRLRDLLL

>B.GB.09.E355_A10.HQ595777

SYHRLRDLLL

>B.GB.09.F432b_p1.JF680911

SYHRLRDLLL

>B.GB.09.G652_p5.JF680913

SYQRLRDLLL

>B.GB.09.J525_E4.HQ595783

SYHLLRDLLL

>B.GB.09.L000b_p1.JF680919

SYHRLRDLLL

>B.GB.09.L320_p1.JF680920

SYHRLRDLLL

>B.GB.09.L563_H1.HQ595786

SYRRLRDLLL

>B.GB.09.M263_p1.JF680923

LYHHLRDLLS

>B.GB.09.M600c_p1.JF680924

SYHRLRDLLL

>B.GB.09.P150_p1.JF680928

SYHRLRDLLL

>B.GB.09.P362b_p1.JF680929

SYHRLRDLLL

>B.GB.09.S200c_p1.JF680933

LYHRLRDFLL

>B.GB.09.S225_E7.HQ595797

SYRRLRDLLL

>B.GB.09.S263_p4.JF680934

SYHRLRDLLL

>B.GB.09.S321_E3.HQ595801

SYHRLRDLLL

>B.GB.09.S520_G7.HQ595802

SYHRLRDLLL

>B.GB.09.T516_C7.HQ595804

SYHRLRDLLL

>B.GB.09.T520b_F9.HQ595805

SYHRLRDLLL

>B.GB.09.W450b_C12.HQ595806

SYHRLRDLIL

>B.GB.09.W452_p1.JF680937

SYHRLRDLLL

>B.GB.13.13592_1_17.3.MF109359

LYHRLRDLLL

>B.GB.13.13592_1_20.3.MF109363

SYHRLRDLLS

>B.GB.13.13592_1_21.3.MF109364

SYHHLRDLLL

>B.GB.13.13592_1_26.3.MF109369

SYHHLRDLIL

>B.GB.13.13592_1_29.3.MF109372

SYHRLRDLLL

>B.GB.13.13592_1_33.3.MF109376

SYHRLRDLLL

>B.GB.13.13592_1_4.3.MF109388

SYRHLRDLLL

>B.GB.13.13659_1_66.3.MF109454

SYHRLRDLLL

>B.GB.13.13774_1_47.3.MF109472

SFHLLRDLLL

>B.GB.13.13774_1_50.3.MF109475

SYHRLRDLLL

>B.GB.13.13774_1_54.3.MF109479

SYHRLRDLLL

>B.GB.13.13774_1_55.3.MF109480

SYHRLRDLLL

>B.GB.13.13774_1_63.3.MF109487

SYHRLRDLLL

>B.GB.13.13774_1_71.3.MF109494

SYHHLRDLLL

>B.GB.13.13774_1_77.4.MF109499

CYHRLRDLLL

>B.GB.13.13774_1_79.3.MF109501

SYHRLRDLLL

>B.GB.13.13774_1_84.3.MF109506

SYHRLRDLLL

>B.GB.13.15171_1_25.4.MF109656

SYHRLRDLLL

>B.GB.13.15171_1_32.3.MF109664

SYHRLRDLLL

>B.GB.13.15171_1_35.4.MF109667

SYRLLRDLLL

>B.GB.13.15171_1_42.3.MF109675

LYHRLRDFLL

>B.GB.13.15228_1_67.4.MF109705

SYHRLRDLLL

>B.GB.14.13612_1_11.3.MF109395

SYHHLRDLLL

>B.GB.14.13612_1_12.4.MF109396

SYHRLRDLLL

>B.GB.14.13612_1_17.3.MF109400

SYHHLRDLLL

>B.GB.14.13612_1_25.3.MF109408

SYRRLRDLIL

>B.GB.14.13612_1_34.3.MF109418

IYHRLRDLLL

>B.GB.14.13612_1_38.3.MF109422

SYHRLRDFLL

>B.GB.14.13612_1_41.3.MF109426

SYHRLRDLLL

>B.GB.14.13659_1_58.3.MF109446

SYHRLRDLLL

>B.GB.14.13659_1_59.3.MF109447

SYHRLRDLLL

>B.GB.14.13659_1_61.3.MF109449

SYHRLRDLLL

>B.GB.14.13659_1_64.3.MF109452

SYHRLRDLLL

>B.GB.14.13659_1_65.3.MF109453

IYHRLRDLLL

>B.GB.14.13659_1_69.3.MF109456

SYHRLRDLLL

>B.GB.14.13659_1_75.3.MF109461

SYLRLRDLLL

>B.GB.14.14535_1_27.3.MF109524

SYHRLRDLLL

>B.GB.14.14535_1_28.4.MF109525

SYHRLRDLLL

>B.GB.14.14535_1_5.3.MF109533

SYHRLRDLLL

>B.GB.14.14535_1_7.3.MF109534

SYHRLRDLLL

>B.GB.14.14535_1_9.3.MF109536

SYHHLRDLLL

>B.GB.14.14592_1_44.3.MF109540

SYHRLRDLLL

>B.GB.14.14592_1_47.3.MF109543

SYRHLRDLLL

>B.GB.14.14592_1_52.4.MF109546

SYHRLRDLLL

>B.GB.14.14592_1_57.3.MF109551

SYHRLRDLLL

>B.GB.14.14592_1_64.3.MF109558

SYHRLRDLLL

>B.GB.14.14592_1_67.3.MF109561

SYHRLRDLLL

>B.GB.14.14667_1_16.3.MF109578

SYHRLRDLLL

>B.GB.14.14667_1_2.4.MF109590

LYHRLRDLLL

>B.GB.14.14667_1_36.3.MF109596

SYHRLRDLLL

>B.GB.14.14727_1_40.3.MF109605

SYHRLRDLLL

>B.GB.14.14727_1_53.3.MF109616

SYHRLRDLLL

>B.GB.14.14727_1_63.3.MF109624

SYQRLRDLLL

>B.GB.14.14727_1_66.3.MF109627

IYHRLRDLLL

>B.GB.14.14727_1_69.3.MF109630

SYHRLRDLLL

>B.GB.83.CAM1.D10112

SYHRLRDLLL

>B.GB.86.GB8_C1.Y13716

SYHRLRDLLL

>B.GB.91.E21LnD13.JF706393

SYHLLRDLLL

>B.GB.93.NA20_LN23_14_4.JN786861

SYHRLRDFLL

>B.GB.94.746DEN07.AJ535594

SYHRLRDLLL

>B.GB.94.749CD352.AJ535607

SYRRLRGLIL

>B.GB.94.NIBSC_1.KJ019215

SYHRLRDLLL

>B.GB.95.817CD302.AJ535618

LYHRLRDLLL

>B.GB.95.822CD345.AJ535611

SYHRLRDLLL

>B.GB.96.875DEN49.AJ535612

SYHRLRDLLL

>B.GB.97.CW002.AJ418531

SYRRLRDLLL

>B.GB.97.CW010.AJ418494

SYHRLRDLLL

>B.GB.97.CW012.AJ418495

SYHRLRDLLL

>B.GB.97.CW037.AJ418519

SYHRLRDLLL

>B.GB.97.CW048.AJ418521

SYHRLRDLLL

>B.GB.x.AC_46.U36863

SYHRLRDLLL

>B.GB.x.JB.U36869

SYHRLRDLLL

>B.GB.x.M23470_MOC3.U36872

SYHRLRDLLS

>B.GB.x.M2424.4_clone3.AJ286342

IYHRLRDLLL

>B.GB.x.M26864_M4C4.U36875

SYHRLRDLLL

>B.GB.x.M30156_M6C5.U36877

SYHRLRDLLS

>B.GB.x.M737677_DT14.U36879

SYHRLRDLLL

>B.GB.x.M737685_DT10.U36880

SYHRLRDLLL

>B.GB.x.MANC.U23487

SYHRLRDLLS

>B.GB.x.MB314.Y13719

SYHHLRDLLL

>B.GB.x.PE052_1.AJ286330

SYHRLRDLLL

>B.GB.x.PE101_1.AJ286332

SYHRLRDLLL

>B.GB.x.PE104_38.AJ286334

SYHRLRDLLL

>B.GB.x.PE106_4.AJ286336

SHHRLRDLLL

>B.GB.x.PE124_1.AJ286337

IYHLLRDFLL

>B.GB.x.PE131_3.AJ286339

SYHRLRDLLL

>B.GB.x.UK1BR_15.AF491740

SYHRLRDLLL

>B.GB.x.UK6_5a_9.HQ122397

SYHRLRDLLL

>B.GB.x.UK7br2.JN002029

SYHRLRDLLL

>B.GB.x.WB.U36882

SYHRLRDLLL

>B.GE.03.03GEMZ004.DQ207940

SYHRLRDLLF

>B.GE.03.03GEMZ010.DQ207942

SYHRLRDLLL

>B.GM.09.N059733.HQ385456

SYRHLRDLLL

>B.GW.14.DEMB14GW004.MH078546

SYHLLRNLLL

>B.HK.06.HK002.FJ460499

SYHRLRDLLS

>B.HK.06.HK004.FJ460501

SYHRLRDLLL

>B.HT.05.05HT_129389.EU839602

SYHHLRDFLL

>B.HT.05.05HT_129473.EU839603

SYHRLSDLLL

>B.HT.05.05HT_129517.EU839600

SYHRLRDLLL

>B.HT.05.05HT_129696.EU839601

SYHRLRDLLL

>B.HT.05.05HT_129805.EU839604

SYHHLRDLLL

>B.HT.11.DEURF11HT001.MH078551

SYHRLRDLLL

>B.IN.x.11807.EF694037

SYHRLRDLLL

>B.IT.05.131_D1.GU191372

SYHRLRDLLL

>B.IT.09.P400_p2.JF680930

SYHLLRDSLL

>B.IT.09.T300b_p2.JF680936

LYHRLRDLLL

>B.IT.92.136_3_CHILD_2.KF061032

SYHRLRDLLL

>B.IT.94.306_9_CHILD_1.KF061031

SYHRLRDLLL

>B.IT.95.TRO_11.AY835445

SYHRLRDLLL

>B.IT.96.PVO_4.AY835444

SYHRLTDLLL

>B.JM.05.05JM_KJ108.EU839605

SYHRLRDLTL

>B.JM.08.N242_p1.JF680925

SYHRLRDLLL

>B.JM.09.09JM_PF09WX.HM030564

TYHHLRDLLL

>B.JM.09.09JM_PF09XN.HM030559

SYHLLRNFLL

>B.JM.09.09JM_PF09YT.HM030560

SYHRLRDLLL

>B.JM.09.09JM_PF0B8J.HM030561

SYHRLTDLLL

>B.JM.09.09JM_PF0B97.HM030562

ICRHLRDLLL

>B.JM.09.09JM_PF0B9L.HM030565

SYHRLRDLLL

>B.JM.09.09JM_PF0BB5.HM030563

LYHRLRDLLL

>B.JP.00.117.AB428551

SYHHLRDLLL

>B.JP.00.DR2508.AB289588

SYHRLRDLLL

>B.JP.00.DR2510.AB287372

SYHRLRDLLL

>B.JP.01.134.AB428552

SYHRLRDLLL

>B.JP.01.DR388.AB289590

SYHRLRDLIL

>B.JP.03.227.AB428555

SYHRLRDLLL

>B.JP.03.285.AB428558

SYHRLRDLLL

>B.JP.04.04JPDR6075B.AB221126

SYHHLRDLLL

>B.JP.04.DR5913.AB480697

SYHRLSDLLL

>B.JP.04.DR6174.AB480693

SYHRLRDLLL

>B.JP.04.DR6175.AB480695

SYHRLRDLLL

>B.JP.05.426.AB428556

SYHHLRDLLL

>B.JP.05.DR6538.AB287363

SYRHLRDFLL

>B.JP.05.DR6657.AB588243

SYHRLRDLLL

>B.JP.05.DR6737.AB287364

SYHRLRDLLL

>B.JP.05.DR6739.AB588255

SYHRLIDLLL

>B.JP.05.DR6826.AB588264

IYHRLRDLLL

>B.JP.05.DR6871.AB588271

SYHRLRDLLL

>B.JP.05.DR6946.AB588283

SYHRLRDLLL

>B.JP.05.DR7015.AB588296

SYHRLRDLLL

>B.JP.05.DR7060.AB287367

SYHRLRDLLL

>B.JP.05.DR7065.AB287368

SYHQLRDSIL

>B.JP.06.DR7259.AB588311

LYHHLRDLLL

>B.JP.06.DR7374.AB588331

SYHRLRDLLL

>B.JP.08.NMC104_clone_01.AB731663

SYHRLRDLLL

>B.JP.09.NMC127_clone_07.AB731667

SYHRLRDLTL

>B.JP.11.DEMB11JP002.KF716497

SYRHLRDLLL

>B.JP.11.NMC851C_clone_13.AB731669

SYHRLRDLLL

>B.JP.12.DEMB12JP001.KF716498

SYHRLRDLLL

>B.JP.88.02.AB588201

SYHLLRDLLL

>B.JP.88.03.AB588207

SYHRLRDLLL

>B.JP.89.31.AB588209

SYHRLRDLLL

>B.JP.89.33.AB588211

SYHRLRDLLS

>B.JP.89.40.AB588226

SYHLLRDLLL

>B.JP.89.60.AB588233

SYHRLRDLLL

>B.JP.98.DR1120.AB480698

IYHRLRDLLL

>B.JP.x.DR1673.AB564745

LYHRLRDLLL

>B.JP.x.DR1712.AB604946

SYHRLRDLLL

>B.JP.x.ETR.D12582

SYHRLRDLLL

>B.JP.x.JRC03B.AB565496

SYRHLRDLLL

>B.JP.x.JRC05B.AB565497

SYHHLRDLLL

>B.JP.x.JRC65B.AB565502

SYRHLRDLLL

>B.JP.x.KKwt_12.KT961002

SYHRLRDLLL

>B.JP.x.KP_5_48_C05.AB742153

SYHRLRDLLL

>B.JP.x.MOKW_RNL.AB262961

SYHHLRDFLL

>B.JP.x.pJPDR0796B02.AB565478

SYHHLRDLLL

>B.JP.x.pJRC57B09.AB641836

IYHRLRDFLL

>B.KR.00.00LJI12_13346.KU869602

IYRRLRDLLL

>B.KR.02.HP_19_02LGS11_3443.KJ140264

SYRRLRDLLL

>B.KR.02.HP_20_02KJO10_3480.KJ140265

SYRLLRDLLL

>B.KR.02.HP_4_02KGJ10_4782.KJ140249

SYHRLRDLLL

>B.KR.03.03HJY8.JQ316131

SYHRLRDLLL

>B.KR.03.03KDE11.JQ316128

SYRHLRDLLL

>B.KR.03.03KGS5.JQ316132

SYHRLRDLLL

>B.KR.03.03LSH1.JQ316127

SYRRLRDLLL

>B.KR.03.03YGS3.JQ316135

SYHSLRDLLL

>B.KR.03.HP_15_03LSW3_6491.KJ140260

SYRHLRDLLL

>B.KR.04.04CWS5.JQ316133

SYRSLRDLIL

>B.KR.04.04KJS8.JQ316130

SYRHLRDLLL

>B.KR.04.04KJin8_1955.DQ295195

LYHRLRDLLL

>B.KR.04.04KMH5.DQ295193

SYRRLRDLLL

>B.KR.04.04KMK5.JQ316126

SYRNLRDLLL

>B.KR.04.04KYR8.DQ295196

SYHRLRDLIL

>B.KR.04.04LHS6.AY839827

IYRNLRDLLL

>B.KR.04.04LSK7.DQ295192

SYHSLRDLLL

>B.KR.05.05CSR3.DQ837381

LYHRLRDLLL

>B.KR.05.05YJN2.JQ316134

SYRRLRDLLL

>B.KR.07.HP_18_07JHS10_3909.KJ140263

IYRHLRDLLL

>B.KR.09.KOR_HIV_Env_10.KT878030

SYHRLRDLIL

>B.KR.09.KOR_HIV_Env_11.KT878031

SYHRLRDLLL

>B.KR.09.KOR_HIV_Env_12.KT878032

IYHRLRDLLL

>B.KR.09.KOR_HIV_Env_13.KT878033

SYRHLRDLLL

>B.KR.09.KOR_HIV_Env_15.KT878035

IYLHLRDLLL

>B.KR.09.KOR_HIV_Env_16.KT878036

SYHRLRDLLL

>B.KR.09.KOR_HIV_Env_17.KT878037

SYHRLRDLIL

>B.KR.09.KOR_HIV_Env_18.KT878038

SYHRLRDLLL

>B.KR.09.KOR_HIV_Env_19.KT878039

IYHRLKDLLL

>B.KR.09.KOR_HIV_Env_1.KT878021

SYHRLRDLLL

>B.KR.09.KOR_HIV_Env_2.KT878022

SYHRLRDLLL

>B.KR.09.KOR_HIV_Env_3.KT878023

SYHRLRDLTL

>B.KR.09.KOR_HIV_Env_4.KT878024

IYHRLRDLLL

>B.KR.09.KOR_HIV_Env_5.KT878025

IYRQLRDLLL

>B.KR.09.KOR_HIV_Env_6.KT878026

SYRHLRDLLL

>B.KR.09.KOR_HIV_Env_7.KT878027

SYRRLRDLLL

>B.KR.09.KOR_HIV_Env_8.KT878028

IYRRLRDLLS

>B.KR.09.KOR_HIV_Env_9.KT878029

SYHRLRDLLL

>B.KR.12.12KYY10_10742.KF561441

SYHSLRDLLL

>B.KR.14.HP_14_12KTG8_11243_11035_11272.KU869559

VYHRLRDLLL

>B.KR.91.91OSG10.KF561442

SYRRLRDLLL

>B.KR.92.92CYK6_13395.KU896118

IYRRLRDLLL

>B.KR.92.92KYJ5_13316.KU869580

SYHRLRDLLL

>B.KR.92.92PJA12_13307.KU869596

SYRRLRDLLL

>B.KR.92.92SJCL8_15336.KX960971

SYHRLRDLLL

>B.KR.92.93LSW7_10899.KJ140266

SYHRLRDLLL

>B.KR.92.94KHB5_3295.KJ140267

SYRRLRDLLL

>B.KR.92.HP_10_02SHJ8_6986.KJ140255

SYHRLRDLLL

>B.KR.92.HP_11_02PGU10_4780.KJ140256

SYHRLRDLLL

>B.KR.92.HP_12_02LGH10_3448.KJ140257

SYRHLRDLLL

>B.KR.92.HP_16_12JIS11_6075.KJ140261

SYHLLRDFLL

>B.KR.92.HP_2_97JJW2_12101.KJ140247

SYRHLRDLLL

>B.KR.92.HP_3_02LJW8_4757.KJ140248

SYRLLRDLLL

>B.KR.92.HP_6_03JHJ2_3477.KJ140251

SYRRLRDLLL

>B.KR.92.HP_7_96LSM10_3474.KJ140252

IYHRLRDLLL

>B.KR.92.KR2057_C5.AJ417425

SYHRLRDLLL

>B.KR.93.93JIJ6_14923.KY820529

SYHRLRDLLL

>B.KR.93.93LSY2_13324_13322.KU869586

SYRRLRDLLL

>B.KR.93.HP_17_02LSP11_2268.KJ140262

IYHRLRDLLL

>B.KR.93.KRA812_C3.AJ417422

SYHHLRDLLL

>B.KR.95.95KJHw4_15364.KX960978

SYHRLRDLLL

>B.KR.95.HP_5_95PJH6_10862.KJ140250

SYRRLRDLLL

>B.KR.95.KR5076_K4.AJ417415

SYHRLRDLLL

>B.KR.95.KR5086_C8.AJ417431

SYHILRDLLL

>B.KR.96.KR3026_C4.AJ417428

SYHRLRDLLL

>B.KR.96.KR3042_K4.AJ417410

TYRHLRDLLL

>B.KR.96.KR5058_C8.AJ417413

SYHRLRDLLL

>B.KR.96.KR6035_C4X.AJ417419

SYRRLRDLLL

>B.KR.97.WK.AF224507

SYHLLRDLLL

>B.KR.99.99HYH2.JQ316129

SYHRLRDLLL

>B.MM.99.mSTD101.AB097870

SYHRLRDLLW

>B.NL.00.671_00T103.AY423386

SYHRLRDLLL

>B.NL.02.patient_B_5_11.HQ386159

SYRRLRDLLL

>B.NL.02.patient_C_27_5.HQ386180

SYHRLRDLLL

>B.NL.03.patient_A_20_3.HQ386146

SYHRLRDLLL

>B.NL.04.patient_D_27_9.HQ386198

SYHRLRDLLL

>B.NL.05.patient_E_42_6.HQ386218

SYHRLRDLLL

>B.NL.86.H0320_2A12_ACH3202A12.U34603

SYHRLRDLLL

>B.NL.86.H434_8_A3.AY970946

SYHRLRDLLL

>B.NL.87.19298_9E8.GU455480

SYHRLRDLLL

>B.NL.88.19554_16_1H1.GU455465

SYHRLRDLLL

>B.NL.90.H5_25_7G2.EU744159

SYHRLRDLLL

>B.NL.91.H4_078_1D11.EU744114

SYHRLRDLLL

>B.NL.92.19663_31C9.GU455505

IYHRLRDLLL

>B.NL.93.H3_40_10C9.EU744081

SYHRLRDLLL

>B.NL.94.19642_38_1G7.GU455448

SYHRLRGLLL

>B.NL.94.ACH142_E11.DQ178989

SYHRLRDLLL

>B.NL.94.H1_46_5G2.EU744007

SYHRLRDLLL

>B.NL.95.19956_45_1E8.GU455525

SYHRLRDLLL

>B.NL.95.H2_114_7H3.EU744052

SYHRLRDLLL

>B.NL.x.168A.U15030

SYHRLRDLLL

>B.NL.x.ENVVA_P16C1.L08655

SYHRLRDLLL

>B.PE.06.502_0491_wg5.JF320183

SYHHLRDLLL

>B.PE.06.502_0524_FL04.JF320008

SYHRLRDLLL

>B.PE.06.502_0648_FL02.JF320215

SYHHLRDLLL

>B.PE.06.502_0841_FL04.JF320208

IYHRLRDLLL

>B.PE.06.502_2717_RH03.JF320230

LYHRLRDLLL

>B.PE.06.502_2794_FL05.JF320244

SYHRLRDLLW

>B.PE.07.502_0525_wg5.JF320191

SYHRLRDLLL

>B.PE.07.502_1047_wg5.JF320226

SYHRLRDLLL

>B.PE.07.502_1399_wg4.JF320013

LYHRLRDLLL

>B.PE.07.502_2254_FL6.JF320018

LYHRLRDLLL

>B.PE.07.502_2349_wg2.JF320028

SYHRLRDLLL

>B.PE.07.502_2622_wg1.JF320189

SYHQLRDLLL

>B.PE.07.502_2649_wg8.JF320019

SYHHLRDLLL

>B.PE.12.FOST088.MF990465

SYHRLRDLLL

>B.PE.13.DEMB13PE010.MH078552

SYHRLRDLLL

>B.PE.14.DEMB14PE008.MH078553

SYHRLRDLLL

>B.PE.16.DEMB16PE003.MH078554

SYHRLRDLLL

>B.PE.16.DEMB16PE009.MH078555

SYHQLRDLIL

>B.PE.x.H022_7.EF210725

SYHRLRDLLL

>B.PE.x.H029_12.EF210726

SYHRLRDLLL

>B.PE.x.H030_7.EF210727

SYHRLRDLLL

>B.PE.x.H035_18.EF210729

SYHRLRDLLL

>B.PE.x.H061_14.EF210730

SYHRLRDLLL

>B.PE.x.H077_31.EF210734

SYHRLRDLLL

>B.PE.x.H079_2.EF210731

SYHRLRDLLL

>B.PE.x.H080_23.EF210735

SYHRLRDFLL

>B.PE.x.H086_8.EF210732

SYHRLRDLLL

>B.PH.15.1003.MH327746

SYHRLRDLLS

>B.PH.15.DEMB15PH002.KY658689

SYRRLTDLLL

>B.PH.15.DEMB15PH003.KY658690

SYHRLRDLLS

>B.PH.16.1022.MH327757

SYHHLRDFLL

>B.PL.x.DEMBXXPL001.KC596069

SYHRLRDLLL

>B.PY.02.02PY_PSP0019.JN251896

SYHRLRDLLL

>B.PY.02.02PY_PSP0090.JN251901

SYHRLRDLLL

>B.PY.03.03PY_PSP0115.JN251906

SYHHLRDLLL

>B.RU.04.04RU128005.AY682547

SYHRLRDLLL

>B.RU.04.04RU129005.AY751406

SYHRLRDLLL

>B.RU.04.04RU139089.AY751407

SYHRLRDLLL

>B.RU.04.04RU139095.AY819715

IYHRLRDLLL

>B.RU.07.RU_21_07_A9_9.HQ896488

SYHRLRDLLL

>B.RU.09.09RU4457.JX500709

SYHRLRDLLS

>B.RU.10.10RU6629.JX500707

SYHRLRDLLL

>B.RU.11.11RU21n.JX500708

LYHRLRDLLL

>B.SE.00.P2b_20000615.GU204932

SYHRLRDLLL

>B.SE.03.003SE.MF373125

SYHRLRDLLL

>B.SE.03.005SE.MF373127

SYHRLRDLLS

>B.SE.05.007SE.MF373129

SYHHLRDLLL

>B.SE.05.008SE.MF373130

SYHRLRDLLL

>B.SE.05.SE600063.KP411829

SYHRLRDLLL

>B.SE.08.028SE.MF373142

SYHRLRDLLL

>B.SE.09.030SE.MF373144

SYHHLRDLLL

>B.SE.09.SE600001.KP411822

LYHRLRDLLL

>B.SE.10.040CA.MF373151

SYHRLRDLLL

>B.SE.10.SE600012.KP411823

QFLLLRPLIR

>B.SE.10.SE600046.KP411827

NCLRLRDLIL

>B.SE.11.058SE.MF373161

SYHRLRDLLL

>B.SE.11.059SE.MF373162

SYHRLRDLLL

>B.SE.11.SE600023.KP411824

IFLYRRDLLL

>B.SE.11.SE600034.KP411825

SYHRLRDLLL

>B.SE.12.071EG.MF373174

SYHRLRDLLL

>B.SE.12.SE600057.KP411828

LYHRLRDLLL

>B.SE.13.083ET.MF373185

SYHRLRDLLL

>B.SE.13.084TH.MF373186

RYHRLRDLLL

>B.SE.14.099US.MF373201

SYHRLRDLLL

>B.SE.15.101SE.MF373203

SYHLLRDLLL

>B.SE.15.102SE.MF373204

SYHLLRDLLL

>B.SE.91.P1b_19910605.GU204923

SYHRLRDLLL

>B.SE.92.P8a_19921110.GU204941

SYHRLRDLLL

>B.SE.95.P1a_19950824.GU204920

SYHRLRDLLL

>B.SE.96.P2a_19960723.GU204926

SYHRLRDLLL

>B.SE.96.P4a_19960607.GU204934

SYHRLRDLLL

>B.SE.98.s4_25_9.JN251856

SYHRLSDLLL

>B.SG.08.HM024.KY213741

SYHRLRDLLL

>B.SG.08.HM039.KY213740

SYHRLRDLLL

>B.SG.08.HM075.KY213747

SYRHLRDLLL

>B.SG.08.HM080.KY213743

SYHRLRDLLL

>B.SG.08.HM106.KY213751

SYRHLRDLLL

>B.SG.09.HM122.KY213748

SYRHLRDLLL

>B.SG.09.HM141.KY213745

SYHRLRDLLL

>B.TH.00.00TH_C3198.AY945710

SYHRLSDLLL

>B.TH.00.3045A06.A2.KJ952535

SYRHLRDLLL

>B.TH.04.04TH317223.JN248321

SYHRLIDLLS

>B.TH.04.04TH601066.JN248329

SYRRLIDLLL

>B.TH.04.04TH803686.JN248333

SYHRLRDLLL

>B.TH.04.04TH808998.JN248335

SYRHLRDLLS

>B.TH.04.04TH821921.JN248337

SYHRLRDLLL

>B.TH.05.05TH355614.JN248343

SYHRLRDLLL

>B.TH.05.05TH356764.JN248344

SYHQLRDLLL

>B.TH.05.05TH357801.JN248346

GYHRLSDLLS

>B.TH.05.05TH429730.JN248347

SYRQLRDFLL

>B.TH.05.05TH440248.JN248348

SYHRLRDLLW

>B.TH.05.05TH645189.JN248353

SYHRLRDLLL

>B.TH.05.05TH736580.JN248354

SYHRLRDLLL

>B.TH.05.T286588_sga01.JF297222

SYHHLRDLLL

>B.TH.06.AA010a_WG3.JX446800

SYRQLRDLLL

>B.TH.06.AA011a08R.JX446818

SYHRLTDLLL

>B.TH.06.AA093a_RH1.JX447795

SYHRLRDLLL

>B.TH.06.NPBQC.KJ769147

SYHRLRDLLL

>B.TH.07.AA040a_WG11.JX447156

SYHRWRDLVL

>B.TH.08.AA115c03R.JX448103

SYHRLSDLLS

>B.TH.08.MERLBDTRC10.JN860769

SYRRLRDLLL

>B.TH.08.T503963_sga03.JF297229

SYHRLSDLLL

>B.TH.10.DEMB10TH002.KP109514

SYHRLRDLLS

>B.TH.90.BK132.AY173951

SYHRLRDLLL

>B.TH.93.93TH067.U39258

SYHRLRDLLL

>B.TH.96.M081.DQ354116

SYHRLRDLLL

>B.TH.96.M140.DQ354112

SYHSLRNLLL

>B.TH.96.M145.DQ354118

SYHRLRDLLS

>B.TH.96.M149.DQ354119

SYHRLRDLLS

>B.TH.99.99TH_C1416.AY945711

SFHRLRDLLL

>B.TH.x.NKR_0512_8.HM215431

SYRRLRDLLL

>B.TH.x.RPW_0510_2.HM215435

SYHRLRDCLS

>B.TT.00.00TT_CRC08767.EU839606

SYHRLRDLLL

>B.TT.00.00TT_CRC50018.EU839607

SYHRLRDLLL

>B.TT.00.SC46C_A4.HQ217662

SYHHLRDLLL

>B.TT.01.01TT_CRC50069.EU839608

SYHHLRDLLL

>B.TT.01.TT103C_2E1.EU578539

SYHHLRDLLL

>B.TT.01.TT106C_A1.HQ217760

SYHRLRDLLL

>B.TT.01.TT112PC_DUK50127_1H3.EF593260

SYHRLRDLLL

>B.TT.01.TT113PC_2E7.EU578580

SYHRLRDLLL

>B.TT.01.TT114PC_8A12.EU578603

SYHRLRDLLL

>B.TT.93.QH0016_M.AF277059

SYHRLRDLLL

>B.TT.93.QH0065_M.AF277060

SYHHLRDLLL

>B.TT.94.QH0060_F.AF277055

SYHRLRDLLL

>B.TT.94.QH0692_M.AF277065

SYHRLRDLLL

>B.TT.94.QH0705_M.AF277066

SYHRLRDLLL

>B.TT.94.QH0788_M.AF277067

SYHRLRDLLL

>B.TT.94.SC22_QH0126_3B9.EF593245

SYHRLRDLLL

>B.TT.95.QH0791_M.AF277068

SYHRLTDLLL

>B.TT.95.QH0864_M.AF277070

SYHHLRDLLL

>B.TT.95.QH0865_M.AF277071

SYHRLRDLVL

>B.TT.95.QH0908_F.AF277072

SYHRLRDLLL

>B.TT.95.QH0944_M.AF277073

SYHRLRDLLL

>B.TT.97.SC51C_8A2.EU578429

SYHRLRDLLL

>B.TT.98.SC24C_1B1.EU578386

SYHRLRDLLL

>B.TT.98.TT27P_8G3.EU577148

SYHRLRDLLL

>B.TT.98.TT28P_IHV42011_2C7.EF593264

LYHRLRDLLL

>B.TT.99.TT34P_CRC04276_8D1.EF593267

SYHRLRDLLW

>B.TT.99.TT35P_CRC04429_7H12.EF593268

SYHRLRDLLL

>B.TT.x.QZ4589.U32396

SYHRLRDLLL

>B.TW.94.TWCYS_LM49.AF086817

SYHRLRDLLL

>B.UA.01.01UAKV167.DQ823362

SYHRLRDLLL

>B.UA.01.01UAKV252.DQ823363

SYHRLRDLLL

>B.UA.01.01UAKV259.DQ823364

SYHRLRDLLL

>B.US.00.14294_1.DQ853436

SYHRLRDLLL

>B.US.00.4033_P10.JN562768

SYHRLRDLLL

>B.US.00.929172.KT124806

IYHRLRDLLL

>B.US.00.APV_2.DQ869026

SYHRLRDLLL

>B.US.00.ES1_20.EF363123

SYHRLRDLLL

>B.US.00.PRB958_06_TB1_4305.EU289199

SYRRLRDLLL

>B.US.00.RHPA_TF1.JN944917

SYHRLRDLLL

>B.US.00.THRO_TF1.JN944930

SYHRLRDLLL

>B.US.00.WITO_TF1.JN944938

SYHRLRDLLL

>B.US.00.Z18_SGA_F8.EF593288

SYHRLRDLLL

>B.US.00.Z20_B3.EF593289

IYHRLRDLLL

>B.US.01.108051_006.HM769944

LYHRLRDLLL

>B.US.01.45_01D7.JQ609746

SYHRLRDLLL

>B.US.01.6568SP12_13_18_EPI.JN786692

SYHRLRDLLL

>B.US.01.APV_11.DQ869017

SYHRLRDLLL

>B.US.01.APV_13.DQ869019

SYHRLRDLLL

>B.US.01.APV_1.DQ869014

SYHRLRDLLL

>B.US.01.APV_3.DQ869027

SYHRLRDLLL

>B.US.01.APV_4.DQ869028

SYHRLRDLLL

>B.US.01.CRPE_B13.EU578061

SYHRLRDLLL

>B.US.01.L805.FJ469738

SYHRLRDLLL

>B.US.01.P1189_86.62_7A.KT283937

SYHRLKDFLL

>B.US.01.REJO_TF1.JN944911

SYHRLTDLLL

>B.US.01.SMRE_SGA_C2.EF593254

SYHRLRDLLL

>B.US.01.TRJO_TF1.JN944936

SYHHLRGLLL

>B.US.01.Z23_A2.EF593290

SYHRLRDLLL

>B.US.01.Z27_F10.EF593291

IYHRLRDLLL

>B.US.01.Z29_A12.EU577629

SYHHLRDLLL

>B.US.01.Z30_SGA_E13.EF593293

SYHRLRDLLL

>B.US.01.Z31_B7.EF593294

SYRHLRDLLL

>B.US.02.04013171_3_2A2.GU330249

SYHRLRDLLL

>B.US.02.04013211_3_A10.GU330333

SYHRLRDLLL

>B.US.02.04013226_2_flH11.FJ496078

SYHHLRDLLL

>B.US.02.306029_ENV.JX863967

SYHRLRDLLL

>B.US.02.328659.KT124764

SYHRLRDLLL

>B.US.02.494131.KT124777

SYHRLRDLLL

>B.US.02.848017.KT124795

SYHRLRDLLL

>B.US.02.996401.KT124814

LYHRLRDLLL

>B.US.02.AD75_A11.GU331497

SYHRLRDLLL

>B.US.02.APV_14.DQ869020

LYHRLRDLLL

>B.US.02.APV_15.DQ869021

SYHRLRDLLL

>B.US.02.APV_16.DQ869022

SYHRLRDLLL

>B.US.02.APV_17.DQ869023

SYRLLTDFLL

>B.US.02.CR0017Q.FJ469687

LYHHLRDLLL

>B.US.02.CR0023W.FJ469688

SYHRLRDLLL

>B.US.02.CR0058S.FJ469693

SYHHLIDLLL

>B.US.02.EABE4469_A5.HQ217201

SYHRLRDLLL

>B.US.02.F714.FJ469730

SYRHLRDLLL

>B.US.02.F762P.FJ469735

SYHRLRDLLL

>B.US.02.F797.FJ469737

SYHHLRDLLL

>B.US.02.FOJO_C13.EU578104

SYHRLRDLLL

>B.US.02.HEMA_A13.EU578122

SYHRLRDLLL

>B.US.02.HIV_US_BID_V5249_2002.JQ403102

VYHRLRDLLL

>B.US.02.HIV_US_BID_V5276_2002.JQ403105

SYHRLRDLLL

>B.US.02.L8124P.FJ469741

SYHRLRDLLL

>B.US.02.L861P.FJ469748

SYHRLRDLLL

>B.US.02.L893P.FJ469749

IYHRLRDLLL

>B.US.02.MTF.FJ469751

LYHRLRDLLL

>B.US.02.OLLA_A13.EU578230

SYHSLRDLLL

>B.US.02.PRLS02.FJ469754

IYHRLRDLLL

>B.US.02.PRLS08.FJ469757

SYHRLRDLLL

>B.US.02.PRLS09.FJ469758

SYHRLTDLLL

>B.US.02.PRLS16.FJ469760

SYHRLRDLLL

>B.US.02.PRLS18.FJ469762

SCHRLRDLLL

>B.US.02.SHKE_B1.EU578460

SYHRLSDLLS

>B.US.02.Z32_E11.EF593295

SYHRLRDLLL

>B.US.02.Z33_G11.EF593296

IYHRLRDLLL

>B.US.02.Z34_A3.EF593297

SYHRLRDLLL

>B.US.03.04013240_8_TA3.GU330460

SYHRLRDLLL

>B.US.03.04013296_3_B4.GU330538

SYRHLRDLLL

>B.US.03.04013321_2_2C12.GU330550

SYHRLRDLLL

>B.US.03.043S018.HM234503

LYHRLRDLLL

>B.US.03.1BRHGA_E13_DP.KC312470

SYHRLTDLLL

>B.US.03.574194.KT124781

SYHRLRDLLL

>B.US.03.73739_13874_1.MH060766

SYHRLRDLLL

>B.US.03.933384.KT124807

CYHRLRDLLL

>B.US.03.94959_13871_1.MH060965

SYHRLRDLLL

>B.US.03.981600.KT124813

SYHHLRDLLL

>B.US.03.APV_10.DQ869016

SYHRLRDLLL

>B.US.03.APV_18.DQ869024

SYHHLRDLLL

>B.US.03.APV_19.DQ869025

SYHHLRDLLL

>B.US.03.APV_5.DQ869029

SYRRLRDLLL

>B.US.03.APV_6.DQ869030

LYHRLRDLLL

>B.US.03.APV_7.DQ869031

SYHRLRDLLL

>B.US.03.APV_8.DQ869032

SYHRLRDLLL

>B.US.03.AT01480.FJ469686

SYRRLRDLLL

>B.US.03.CR0154X.FJ469701

LYHRLRDLLL

>B.US.03.DEMB03JP004.KC473846

LYHRLRDLLL

>B.US.03.F7157.FJ469731

SYHRLRDLLL

>B.US.03.FERI_SGA_A1.EF593222

SYHRLRDLLL

>B.US.03.H135v1ed8_6.DQ410465

SYHRLRDLLL

>B.US.03.H28v1e3_3.DQ410422

SYHRLRDLLL

>B.US.03.HIV_US_BID_V5279_2003.JQ403106

SYHRLRDLLL

>B.US.03.L8107.FJ469739

LYHRLRDLLL

>B.US.03.L8152.FJ469742

IYHRLIDLLL

>B.US.03.L8180.FJ469743

SYHRLRDLLL

>B.US.03.MEMI_SGA_E14.EF593228

SYHRLRDLLL

>B.US.03.PRLS01.FJ469753

IYHRLRDLLL

>B.US.03.PRLS04.FJ469755

LYHRLRDLLL

>B.US.03.PRLS12.FJ469759

SYHRLRDLLL

>B.US.03.PRLS24.FJ469764

SYHRLRDLLL

>B.US.03.PRLS25.FJ469765

SYHRLSDLLL

>B.US.03.SH8233.FJ469770

SYHHLRDLLL

>B.US.03.TALA_SGA_B16.EF593256

LYHRLRDLLL

>B.US.03.Z35_C1.EF593298

SYHRLRDLLL

>B.US.03.Z36_E1.EF593299

SYHRLRDLLL

>B.US.04.014837G.FJ469684

LYHRLRDLLL

>B.US.04.1218_A14.HQ216370

SYHRLRDLLL

>B.US.04.1249_D15.HQ216397

SYQNLRDLIL

>B.US.04.1330_C22.HQ216485

SYHRLRDLLL

>B.US.04.1352_A5.HQ216504

SYHRLTDFLL

>B.US.04.154v38Renv_re7.DQ410636

SYHRLRDLLL

>B.US.04.268977.KT124758

SYHRLRDLLL

>B.US.04.306081_ENV.JX863968

IYHHLRDLLL

>B.US.04.306129_ENV.JX863969

SYHQLRDLLL

>B.US.04.306131_ENV.JX863970

SYHHLRDLLL

>B.US.04.3405_B10.EU577792

SYHRLRDLLL

>B.US.04.4051_C35.JN562798

SYHRLRDLLL

>B.US.04.4295_1.EU577811

LYHQLRDLLL

>B.US.04.4911_6.EU577862

IYHRLRDLLL

>B.US.04.5417_B11.EF593190

SYHRLRDLLL

>B.US.04.5539_E6.EU577947

SYHRLRDLLL

>B.US.04.5769_B8.EU577989

SYHHLRDLLL

>B.US.04.6052SP9_27_18.KX156438

SYHRLRDLLL

>B.US.04.6064_10.EU578032

SYHHLRDLLL

>B.US.04.64236_13865_1.MH060681

IYHRLKDLLL

>B.US.04.6771SP10_36_122.KX156451

LYHRLRDLLL

>B.US.04.7036_P9.JN562778

SYHHLRDFLL

>B.US.04.7092_A1.EU578047

IYHRLRDLLL

>B.US.04.7115_C17_0.JN562811

SYHRLRDLLL

>B.US.04.71276_13858_1.MH060746

SYHRLRDLLL

>B.US.04.98158_13868_1.MH060990

LYHRLRDLLL

>B.US.04.APV_20.DQ869015

SYHRLRDLLL

>B.US.04.APV_9.DQ869033

SYHRLRDLLL

>B.US.04.C061711D7.KM259097

SYHRLRDLLL

>B.US.04.C61v1e10.DQ410526

SYHRLRDLIL

>B.US.04.C62v1e2_2.DQ410535

IYHRLRDLLL

>B.US.04.CAAN_A2.AY835452

SYHRLRDLLL

>B.US.04.CE116_FL50_24_93.KX156493

SYHRLRDLLL

>B.US.04.CE148_SP96_46_25.KX156542

SYHRLRDLLL

>B.US.04.CE161PL50_6_82.KX156380

LYHRLRDLIL

>B.US.04.CR0068P.FJ469695

SYHRLTDLLL

>B.US.04.CR0080N.FJ469697

SYHRLRDLLL

>B.US.04.CR0116Y.FJ469698

SYHLLRDLLL

>B.US.04.CR0345Q.FJ469722

SYHRLRDLLL

>B.US.04.DIGA3757_D12.HQ217185

SYHRLRDLLL

>B.US.04.ES10_53.EF363127

SYHRLRDLLL

>B.US.04.ES11_2004_culture.KC935957

SYHRLRDLLL

>B.US.04.ES3v1e10_2.DQ410068

SYHHLRGLLL

>B.US.04.ES4_24.EF363124

SYHRLRDLLL

>B.US.04.ES8_43.EF363126

SYHRLRDLLL

>B.US.04.ES9v1e7_1.DQ410205

SYHRLRDLIL

>B.US.04.F7165.FJ469732

SYHRLRDLLL

>B.US.04.F7172.FJ469733

SYHRLRDLLL

>B.US.04.H148v1ed4_1.DQ410498

SYHRLRDLLL

>B.US.04.H1P2111B4b.JQ251132

SYHRLTDLLL

>B.US.04.H22v1ed5_1.DQ410260

SYHRLRDLLL

>B.US.04.H23v1ed8_3.DQ410292

SYHRLRDLLL

>B.US.04.H25v1e1_3.DQ410356

SYHRLRDLLL

>B.US.04.H26v1e14_2.DQ410403

SYHRLRDLLL

>B.US.04.H9v2ed10.DQ410238

SYHRLRDLLL

>B.US.04.HIV_US_BID_V5258_2004.JQ403103

SYHRLRDLLL

>B.US.04.HIV_US_BID_V5261_2004.JQ403104

LYHLLRDLLL

>B.US.04.HIV_US_BID_V5282_2004.JQ403107

SYHRLTDLLL

>B.US.04.JACH1853_A5.HQ217329

LYHRLRDLLL

>B.US.04.JOTO_A11.EU578165

IYHRLRDLLL

>B.US.04.L819.FJ469745

SYHRLRDLLL

>B.US.04.LAHA_B3.EU578189

CYHRLRDLLL

>B.US.04.P070711B1.KM259452

SYHRLRDLLL

>B.US.04.SAMI_WGA1.EU547186

SYHRLRDLIL

>B.US.04.SH858.FJ469772

SYRQLRDLLL

>B.US.04.UNC2009_1.EF593269

SYHRLRDLLL

>B.US.04.UNC4484_13.EF593271

SYHRLRDLLL

>B.US.04.UNC5283_17.EF593273

SYHRLRDLLL

>B.US.04.UNC5548_11.EF593274

SYHRLTDLLL

>B.US.04.UNC5734_10.EF593275

SYHRLRDLLL

>B.US.04.UNC5799_16.EF593276

FYHRLRDLLL

>B.US.04.USPI71101EI7y04051pcWG2B9.JN024210

SYHRLTDFLL

>B.US.04.USPI83747EI6y04121pcWG2B5.JN024100

SYHRLRDLLL

>B.US.05.012286G.FJ469683

SYHRLTDLLL

>B.US.05.04013383_0_SC13.HQ238279

SYHRLRDLLL

>B.US.05.04013396_0_flG8.FJ496085

LYHRLRDLLL

>B.US.05.05US_SAJ_NVS12.JF689852

SYHRLRDLLL

>B.US.05.05US_SAJ_NVS16.JF689854

SYHRLRDLLL

>B.US.05.05US_SAJ_NVS3.JF689856

IYRHLRDLLL

>B.US.05.05US_SAJ_NVS5.JF689857

SYHRLRDLLS

>B.US.05.05US_SAJ_NVS8.JF689859

SYHRLRDLLL

>B.US.05.05US_SAJ_NVS9.JF689860

SYHRLRDLLL

>B.US.05.1423_F32.HQ216575

SYHRLRDLLL

>B.US.05.1444_A22.HQ216584

SYHRLRDLLL

>B.US.05.1446_A37.HQ216601

SYHRLRDLLS

>B.US.05.1451_C1.HQ216634

SYHRLRDLLL

>B.US.05.1470_D34.HQ216686

LYHRLRDLLL

>B.US.05.1508_E6.HQ216727

SYHHLRDLLL

>B.US.05.1586_B28.HQ216742

SYHRLRDLLL

>B.US.05.1588_A22.HQ216762

SYRHLRDLLL

>B.US.05.1599_A26.HQ216795

SYRHLRDLLL

>B.US.05.1624_A19.HQ216816

SYHRLRDLLL

>B.US.05.1631_A1.HQ216841

SYHRLRDLLL

>B.US.05.1632_A28.HQ216874

SYHRLRDLLL

>B.US.05.306019_ENV.JX863965

SYHRLRDLLL

>B.US.05.306026_ENV.JX863966

SYHRLRDLLL

>B.US.05.306142_ENV.JX863971

IYHRLRDLLL

>B.US.05.306144_ENV.JX863972

SYHRLRDLLL

>B.US.05.306159_FL.JX863919

IYHHLRDLLL

>B.US.05.306196_ENV.JX863983

SYHRLRDLLL

>B.US.05.306203_ENV.JX863984

SYRRLRDLLL

>B.US.05.306209_ENV.JX863985

SYHRLRDLLL

>B.US.05.306212_ENV.JX863986

SYHRLRDLLS

>B.US.05.502_0223_wg1.JF320059

SYHRLRDLLL

>B.US.05.502_1400_FL02.JF320043

LYHHLRDLLL

>B.US.05.502_1926_FL01.JF320361

IYHHLRDLLL

>B.US.05.502_2008_FL04.JF320484

SYHRLTDLLL

>B.US.05.502_2136_FL02.JF320185

SYHRLRDLLL

>B.US.05.502_2495_wg02.JF320054

SYHRLRDLLL

>B.US.05.5057_F11.EU577873

SYHRLRDLLL

>B.US.05.509452.KT124778

SYHRLSDLLS

>B.US.05.5479_A8.EU577917

SYHRLTDLLL

>B.US.05.5791_E6.EU578004

SYHRLRDLLL

>B.US.05.8276SP7_27_177.KX156454

SYRRLRDLLL

>B.US.05.C030913F5.KM258984

SYRRLRDLLL

>B.US.05.C031813E5.KM259000

SYHRLRDLLL

>B.US.05.C063011E10.KM259100

SYHHLRDLLL

>B.US.05.C109v1e18.DQ410616

SYHLLRDLLL

>B.US.05.C93v1e18.DQ410553

SYHRLRDLLL

>B.US.05.C94v1e4.DQ410562

SYHRLTDLLL

>B.US.05.C96v1e10.DQ410585

SYHRLRDLLL

>B.US.05.C98v1e19.DQ410596

SYHRLRDLLL

>B.US.05.CB183SP123_29_53.KX156472

SYHQLRDLLL

>B.US.05.CE104FC1_42_9.KX156483

SYHRLRDLLL

>B.US.05.CE125_SP98_52_1.KX156523

SYHRLRDLLL

>B.US.05.CE128FL55_28_78RNA.KX156374

SYHRLRDLVL

>B.US.05.CR0175S.FJ469703

IYHRLRDLLL

>B.US.05.CR0208W.FJ469706

SYHRLRDLLL

>B.US.05.CR0307R.FJ469717

SYHRLRDLLL

>B.US.05.CR0312W.FJ469718

SYHRLRDLLL

>B.US.05.CR0367Z.FJ469724

SYHRLRDLLL

>B.US.05.ES7v2ed7.DQ410115

SYHRLRDLIL

>B.US.05.L8249.DQ886037

SYHRLRDLLL

>B.US.05.L827.FJ469747

SYHRLRDLLL

>B.US.05.MDR_1c.KF990605

SYHRLRDLLL

>B.US.05.N1_WEEK_4.FJ653236

SYHHLRDLLL

>B.US.05.N5_DAY_1.FJ653127

SYHRLRDLTL

>B.US.05.N7_DAY_1.FJ653159

SYHRLRDLLL

>B.US.05.N8_DAY_1.FJ653196

SYHLLRDLLL

>B.US.05.P10_DAY_1.FJ653573

SYHRLRDLLL

>B.US.05.P11_DAY_1.FJ653597

SYHRLRDLLL

>B.US.05.P2_WEEK_8.FJ653474

SYHRLRDLLL

>B.US.05.P3_WEEK_16.FJ653390

SYHRLRDLLL

>B.US.05.P4_WEEK_4.FJ653478

SYHRLRDLLL

>B.US.05.P5_DAY_1.FJ653360

SYRHLRDLLL

>B.US.05.P7_WEEK_20.FJ653547

SYHRLRDLLL

>B.US.05.P8_WEEK_4.FJ653426

SYHRLRDLLL

>B.US.05.P9_WEEK_12.FJ653571

SYHRLRDLIL

>B.US.05.RHMI4089_A3.HQ217476

SYHHLRDLLL

>B.US.05.ROST4216_B15.HQ217546

SYHSLRDLLL

>B.US.05.STCO_3_A2.KC312435

SYHLLRDLLS

>B.US.05.USPI38417EI33y05051pcWG2B2.JN024363

SYHRLRDLLL

>B.US.05.USPI88403EI14y05121pcWG2B7.JN024344

SYHRLRDLLL

>B.US.05.WICU_B13.EU578629

SYHQLRDLLL

>B.US.05.Z62_p1.HQ217818

SYHRLRDLLL

>B.US.05.Z64_p5.HQ217850

SYRHLRNLLL

>B.US.05.Z71_p1.HQ217860

SYHRLRDLLL

>B.US.05.Z74_p4.HQ217889

SYHRLRDLLL

>B.US.06.04013419_7_2B23.GU330736

SYHRLRDLLL

>B.US.06.04013440_5_B11.GU330809

SYHRLRDLLL

>B.US.06.04013446_4_A1.GU330839

SYRRLRDLLL

>B.US.06.06US_SAJ_C164_SC.JF689862

SYHRLRDLLL

>B.US.06.06US_SAJ_C165_TJ.JF689863

SYHRLRDLLL

>B.US.06.06US_SAJ_C166_SG.JF689864

SYHLLRDLLL

>B.US.06.06US_SAJ_C167_LH.JF689865

SYHRLRDLLL

>B.US.06.06US_SAJ_C168_LS.JF689866

IYHRLRDLLL

>B.US.06.06US_SAJ_C169_JS.JF689867

IYHRLRDLLL

>B.US.06.06US_SAJ_C170_JP.JF689868

SYHRLXDLLL

>B.US.06.06US_SAJ_NVS22.JF689870

SYHHLRDLLL

>B.US.06.06US_SAJ_NVS23.JF689871

SYHRLRDLLL

>B.US.06.06US_SAJ_NVS27.JF689872

SYHRLRDLLL

>B.US.06.06US_SAJ_NVS31.JF689873

SYHRLRDLLL

>B.US.06.06US_SAJ_NVS32.JF689874

SYHRLTDLLL

>B.US.06.06US_SAJ_NVS35.JF689875

SYHRLRDLLL

>B.US.06.06US_SAJ_NVS39.JF689876

SYHRLRDLIL

>B.US.06.1711_D38.HQ216908

SYRRLRDLLL

>B.US.06.1791_D10.HQ216925

SYHRLTDLLL

>B.US.06.1794_A1.HQ216949

SYHHLRDLLL

>B.US.06.2857_p1.FJ152546

SYHRLRDLLL

>B.US.06.2866_P4.FJ152547

IYHHLRDLLL

>B.US.06.2886_p1.FJ152548

SYHRLRDLTL

>B.US.06.306227_ENV.JX863987

SYHRLRDLLL

>B.US.06.306238_ENV.JX863988

SYHRLRDLLL

>B.US.06.306261_ENV.JX863989

SYLRLRDLIL

>B.US.06.306272_ENV.JX863990

SYHRLRDLLL

>B.US.06.306308_ENV.JX863991

SYHRLRDLLL

>B.US.06.306318_ENV.JX863992

LYHRLIDLLL

>B.US.06.306335_ENV.JX863993

SYHRLRDLLL

>B.US.06.4059_P28.JN562781

SYHRLRDLLL

>B.US.06.502_0053_wg06.JF320615

SYHRLRDLLL

>B.US.06.502_0062_FL04.JF320613

SYHRLRDLLL

>B.US.06.502_0176_FL06.JF320363

SYHRLRDLLL

>B.US.06.502_0227_FL05.JF320036

SYHRLRDLLL

>B.US.06.502_0309_wg13.JF320048

SYHRLRDLLL

>B.US.06.502_0322_RH04.JF320308

SYHRLRDLLL

>B.US.06.502_0341_FL05.JF320003

SYHRLRDLLL

>B.US.06.502_0346_wg02.JF320097

SYRRLRDLLL

>B.US.06.502_0572_FL06.JF320038

SYRHLRDLLL

>B.US.06.502_0717_FL02.JF320526

SYHRLRDLLL

>B.US.06.502_0762_RH05.JF320329

SYHRLRDLLL

>B.US.06.502_0839_wg01.JF320263

SYHHLRDLLL

>B.US.06.502_0897_RH05.JF320502

SYHRLRDLLL

>B.US.06.502_0923_wg07.JF320160

SYHRLRDLLL

>B.US.06.502_0961_RH08.JF320011

SYHRLRDLLL

>B.US.06.502_1046_FL04.JF320564

GYLRLRDLLL

>B.US.06.502_1055_FL01.JF320169

LYHRLRDLLL

>B.US.06.502_1174_FL09.JF320053

SYHRLRDLLL

>B.US.06.502_1211_FL01.JF320151

LYHRLRDLLL

>B.US.06.502_1512_FL01.JF320356

SYHHLRDLLL

>B.US.06.502_1619_FL06.JF320126

SYHRLRDLLL

>B.US.06.502_2437_RH01.JF320592

SYHRLRDLLL

>B.US.06.502_2667_FL03.JF320145

SYHRLRDLLL

>B.US.06.689801.KT124791

SYHRLRDLLL

>B.US.06.700010135_135P_76.KR423026

IYHHLRDLLL

>B.US.06.700010150_A11.HQ908232

SYRRLRDLLL

>B.US.06.701010027_E_A1.GU331039

SYHRLRDLLL

>B.US.06.701010068_E_J1.GU331147

SYHRLRDLLL

>B.US.06.9002_082206_CSF_1.KM353665

SYHRLRDLLL

>B.US.06.9003_122006_CSF_13.KM353701

SYHHLRDLLL

>B.US.06.BP00054_RH01.JN687749

LYHRLRDLLL

>B.US.06.BP00055_RH01.JN687750

IYRRLRDLLL

>B.US.06.BP00057_RH02.JN687758

SYHHLRDLLL

>B.US.06.B_x_06_701010043_E_A9.HQ908116

SYHRLRDLLL

>B.US.06.C2P6278D11b.JQ251004

SYHHLRDLLL

>B.US.06.CH010032_48_p1.HM204582

SYHRLRDLLL

>B.US.06.CH010111_w48_p1.HM204589

SYHRLRDLLL

>B.US.06.CH106_TF1.JN944897

SYHRLRDLLL

>B.US.06.CH148.PL.191206.BE.3.KY112061

SYHRLRDLLL

>B.US.06.CH16E_A1.EU576345

SYHRLRDLLS

>B.US.06.CH19E_C9.EU576383

IYHRLRDLLL

>B.US.06.CH58_TF1.JN944907

LYHRLRDLLL

>B.US.06.CH77_TF1.JN944909

IYHRLRDLLL

>B.US.06.CR0047U.FJ469691

SYHRLRDLLL

>B.US.06.CR0078.FJ469696

SYHRLRDLLL

>B.US.06.CR0131.FJ469700

SYHRLRDLLL

>B.US.06.CR0215.FJ469708

SYHHLRDLLL

>B.US.06.CR0222X.FJ469709

SYHRLRDLLS

>B.US.06.CR0228Q.FJ469710

SYHRLRDLLL

>B.US.06.CR0276Z.FJ469714

IYHRLRDLLL

>B.US.06.CR0361T.FJ469723

SYHRLRDLIL

>B.US.06.F1P5129E9.JQ250880

SYHRLRDLLL

>B.US.06.GETO5098_B7.HQ217266

SYHRLRDCLL

>B.US.06.HIV_US_BID_V3047_2006.JQ403068

LYRRLRDLLL

>B.US.06.HIV_US_BID_V4503_2006.JQ403095

SYHRLRDLLL

>B.US.06.HIV_US_BID_V4506_2006.JQ403097

SYHRLRDLLL

>B.US.06.IC.1125.0.D4.KX129209

LYHRLRDSLL

>B.US.06.MCRO_C1.EU578223

SYHRLTDLLL

>B.US.06.MDR_5a.KF990608

SYHRLRDLLL

>B.US.06.P042711E7.KM259364

SYHRLRDLLL

>B.US.06.P110712B4.KM259479

SYHRLRDLLL

>B.US.06.P112012B12b.KM259534

SYHRLTDLLL

>B.US.06.VC10014_111006_E1.KJ698288

IYHRLRDSLL

>B.US.06.YOMI_A6.EU578667

SYHRLRDLLL

>B.US.06.Z86_p24.HQ217957

SYQTLRDFLL

>B.US.06.Z91_p1.HQ217967

SYHRLRDLLL

>B.US.06.Z92_p10.HQ217984

SYHRLRDLLL

>B.US.06.Z94_p1.HQ218020

SYHRLRDLLL

>B.US.06.Z95_p1.HQ218034

LYHRLTDLLL

>B.US.07.04013448_8_B16.GU330865

SYHRLRDLLL

>B.US.07.07US_SAJ_C154.JF689877

LYHRLRDLLS

>B.US.07.07US_SAJ_C156.JF689879

SYHRLRDLIL

>B.US.07.07US_SAJ_C161_H1.JF689883

SYHRLRDLLS

>B.US.07.07US_SAJ_C163_H3.JF689885

SYRHLRDLLL

>B.US.07.07US_SAJ_C166_MS.JF689886

SYHRLRDLLL

>B.US.07.07US_SAJ_C200.JF689887

SYHRLRDLLL

>B.US.07.07US_SAJ_NVS42.JF689889

SYHRLRDLLL

>B.US.07.07US_SAJ_NVS48.JF689890

SYHRLRDLLL

>B.US.07.07US_SAJ_NVS54.JF689892

LYHRLRDLLL

>B.US.07.07US_SAJ_NVS55.JF689893

SYHRLRDLLL

>B.US.07.306336_ENV.JX863994

LYHRLRDLLL

>B.US.07.306340_ENV.JX863995

SYHRLRDLLL

>B.US.07.306344_FL.JX863921

LYHRLRDLLL

>B.US.07.306349_ENV.JX864007

SYHRLRDLLL

>B.US.07.306350_ENV.JX864008

SYHRLRDLLL

>B.US.07.306397_ENV.JX864019

SYHRLRDLLL

>B.US.07.306403_ENV.JX864020

SYHRLRDLLL

>B.US.07.306410_ENV.JX864021

SYHRLRDLLL

>B.US.07.306415_ENV.JX864022

SYHRLRDLLL

>B.US.07.502_0287_RH1.JF320375

SYHRLRDLLL

>B.US.07.502_0364_wg2.JF320563

LYHRLRDLLL

>B.US.07.502_0388_RH06.JF320315

SYHRLRDLLL

>B.US.07.502_0823_05.JF320530

SYHSLRNLLL

>B.US.07.502_0938_RH04.JF320631

SYHRLRDLLL

>B.US.07.502_0965_RH01.JF320385

SYHRLRDLLL

>B.US.07.502_1115_wg1.JF320045

SYHRLRDLLL

>B.US.07.502_1368_RH02.JF320173

SYHRLRDLLL

>B.US.07.502_1478_wg4.JF320150

SYHRLRDLLL

>B.US.07.502_1500_RH01.JF320387

SYHRLRDLLL

>B.US.07.502_1504_RH07.JF320394

SYHRLRDLLL

>B.US.07.502_1518_RH03.JF320117

SYHRLRDLLL

>B.US.07.502_1709_04.JF320467

SYHRLRDLLL

>B.US.07.502_1897_wg6.JF320182

SYHRLRDLLL

>B.US.07.502_2000_RH03.JF320279

SYRHLRDLLL

>B.US.07.502_2241_RH13.JF320539

IYHRLSDLLL

>B.US.07.502_2289_05.JF320197

SYHRLRDLLL

>B.US.07.502_2305_01.JF320577

SYHRLRDLLS

>B.US.07.502_2586_RH04.JF320131

LYHLLRDSLL

>B.US.07.5057SP8_9_52.KX156431

SYHRLRDLLL

>B.US.07.700010224_E_A1.HQ216971

SYHRLRDLLL

>B.US.07.700010246_E_A1.GU330994

SYHRLRDLLL

>B.US.07.700010252_A30.HQ908150

SYHRLRDLLL

>B.US.07.700010333_B8_S.HM638503

SYHRLRDLLL

>B.US.07.701010092_E_A15.HQ217045

SYHRLRDLLL

>B.US.07.701010145_C15.HQ908184

SYHRLRDLLL

>B.US.07.891439.KT124801

SYRRLRDLLL

>B.US.07.9006_032007_CSF_1.KM353733

SYHRLRDLLL

>B.US.07.9019_081307_plasma_19.KM354092

LYHHLRDLLL

>B.US.07.9020_082907_CSF_1.KM354114

SYHRLRDLIL

>B.US.07.AVDA2874_E4.HQ217096

SYHRLRDLLL

>B.US.07.BEKA5842_C6.HQ217102

SYHRLRDLLL

>B.US.07.BP00058_RH01.JN687759

LYHRLRDLLL

>B.US.07.BP00063_RH01.JN687761

SYHRLRDLLL

>B.US.07.BP00064_RH01.JN687762

SYHRLRDLLL

>B.US.07.BP00067_RH01.JN687763

LYHRLRDLLL

>B.US.07.BRJO4843_A1.HQ217115

SYHRLRDLLL

>B.US.07.C010312D6.KM258901

SYHRLRDLLL

>B.US.07.C060512F8.KM259082

SYRLLRDLLL

>B.US.07.CH341s_J8.HQ908139

LYHRLRDLLL

>B.US.07.CH_390s_F19.HQ908109

SYRHLRDLLL

>B.US.07.COCE6096_D16.HQ217160

SYHRLRDLLL

>B.US.07.CR0027M.FJ469689

SYHRLRDLLL

>B.US.07.CR0214.FJ469707

SYRHLRDLLL

>B.US.07.CR0275.FJ469713

LYHLLRDLLL

>B.US.07.CR0295S.FJ469716

SYHRLRDLLL

>B.US.07.CR0339X.FJ469721

LYHRLRDLLL

>B.US.07.HALA6323_F24.HQ217308

SYHRLRDLLL

>B.US.07.HIV_US_BID_V3010_2007.JQ403058

SYHRLRDLLL

>B.US.07.HIV_US_BID_V3020_2007.JQ403059

SYHNLRDLLL

>B.US.07.HIV_US_BID_V3021_2007.JQ403060

SYHRLRDLLL

>B.US.07.HIV_US_BID_V3036_2007.JQ403064

IYHRLRDLLL

>B.US.07.HIV_US_BID_V3044_2007.JQ403066

SYHRLRDLLL

>B.US.07.HIV_US_BID_V3053_2007.JQ403071

SYHRLRDLLL

>B.US.07.HIV_US_BID_V3111_2007.JQ403073

SYHRLTDLLL

>B.US.07.HIV_US_BID_V3115_2007.JQ403075

SYHRLRDLLL

>B.US.07.HIV_US_BID_V3118_2007.JQ403077

SYHHLRDLLL

>B.US.07.HIV_US_BID_V3120_2007.JQ403078

SYHRLRDLLL

>B.US.07.HIV_US_BID_V3512_2007.JQ403092

SYHRLRDLLL

>B.US.07.HIV_US_BID_V3515_2007.JQ403093

SYHRLRDSLL

>B.US.07.HIV_US_BID_V4516_2007.JQ403096

SYHRLRDLLL

>B.US.07.JOSA5789_A9.HQ217347

SYHRLRDLLL

>B.US.07.MCBR4209_TA14.HQ217386

SYHRLRDLLL

>B.US.07.MCST_B17.KC312583

SYHRLRDLLL

>B.US.07.MEJA5586_B9.HQ217430

SYHRLRDLLL

>B.US.07.N26_07A21.JQ610064

SYRHLRDLLL

>B.US.07.P013113C4.KM259264

SYHRLRDLIL

>B.US.07.P020813B10.KM259279

SYHRLRDLLL

>B.US.07.P062212A1.KM259406

SYHLLTDLLL

>B.US.07.P120211C11.KM259566

SYHRLRDLLL

>B.US.07.ROCH4447_B17.HQ217509

LYHRLRDLLL

>B.US.07.SADO6038_C6.HQ217601

LYHRLRDLLL

>B.US.07.SPFE4120_D7.HQ217713

SYHRLRDLLL

>B.US.07.TransmitterDay221.GQ256646

SYHRLRDLLL

>B.US.07.WARO_A13.KC312386

SYHRLRDLLL

>B.US.08.08US_SAJ_C202.JF689894

SYHRLRDLLL

>B.US.08.08US_SAJ_C203.JF689895

SYHRLRDLLW

>B.US.08.08US_SAJ_C204.JF689896

SYHHLRDLLL

>B.US.08.08US_SAJ_C205.JF689897

IYRLLRNFLL

>B.US.08.293050.KT124761

SYHRLRDLLL

>B.US.08.306434_ENV.JX864023

SYHRLRDLLL

>B.US.08.306445_ENV.JX864024

SYHRLRDLLL

>B.US.08.306451_ENV.JX864025

SYRRLKDLLL

>B.US.08.700010464_2C1.HQ615981

SYHHLRDLLL

>B.US.08.700010501_B4_BL.HM638517

SYHLLRDFLL

>B.US.08.700010654.3.d0015.ipe026.3B12.MF499166

LYHRLRDLLL

>B.US.08.700010685_A10.KR423340

SYHRLRDLLL

>B.US.08.701010380_G3_BL.HM638584

SYHRLRDLLL

>B.US.08.9016_011408_CSF_1.KM353846

SYHRLRDLLL

>B.US.08.9021_032508_CSF_1.KM354155

SYHRLRDLLL

>B.US.08.9024_010708_CSF_1.KM354232

SYRHLRDLLL

>B.US.08.9027_052708_CSF_1.KM354355

SYHRLRDLLL

>B.US.08.B.700010607.S.0dps.BF13.JX974238

SCHRLRDLLL

>B.US.08.BP00061_RH01.JN687760

SYHRLRDLLL

>B.US.08.BP00069_RH01.JN687773

SYHRLRDLLL

>B.US.08.B_US_08_470_scr_3_A5.HQ908219

SYHRLRDFLL

>B.US.08.CH010211_w2_p1.HM204596

SCHHLRDLLL

>B.US.08.CH302.PL.041608.UT.7.KY112135

SYHHLRDLLL

>B.US.08.CH378.PL.080508.UT.15.KY112149

SYHRLRDLLL

>B.US.08.CH742.SE.091008.UT.8.KY112490

IYHRLRDLLL

>B.US.08.HIV_US_BID_V3024_2008.JQ403061

SYHRLRDLLL

>B.US.08.HIV_US_BID_V3027_2008.JQ403062

SYHRLRDLLL

>B.US.08.HIV_US_BID_V3032_2008.JQ403063

SYHRLRDLLL

>B.US.08.HIV_US_BID_V3046_2008.JQ403067

SYHRLRDLLL

>B.US.08.HIV_US_BID_V3048_2008.JQ403069

LYHRLRDLLL

>B.US.08.HIV_US_BID_V3050_2008.JQ403070

SYQSLRDLIL

>B.US.08.HIV_US_BID_V3114_2007.JQ403074

SYHHLRNLLL

>B.US.08.HIV_US_BID_V3122_2008.JQ403080

SYHNLRDLIL

>B.US.08.HIV_US_BID_V3128_2008.JQ403082

SYHRLRDLLL

>B.US.08.HIV_US_BID_V4120_2008.JQ403031

CYHRLRDLLL

>B.US.08.HIV_US_BID_V4124_2008.JQ403035

SYHRLTDLLL

>B.US.08.HIV_US_BID_V4388_2008.JQ403083

SYHRLRDLLL

>B.US.08.HIV_US_BID_V4389_2008.JQ403084

SYHRLRDLLL

>B.US.08.HIV_US_BID_V4390_2008.JQ403085

SYHLLRDLLL

>B.US.08.HIV_US_BID_V4391_2008.JQ403086

SYHRLTDLLL

>B.US.08.HIV_US_BID_V4392_2008.JQ403087

SYHRLRDLTL

>B.US.08.HIV_US_BID_V4393_2008.JQ403088

SYHRLRDLLL

>B.US.08.HIV_US_BID_V4394_2008.JQ403089

LYRHLRDLLL

>B.US.08.HIV_US_BID_V4397_2008.JQ403091

IYHRLRDLLL

>B.US.08.HIV_US_BID_V4489_2008.JQ403094

SYHRLRDLLL

>B.US.08.N90_08A6.JQ610123

SYHRLRDLLL

>B.US.08.P15supe_A11_FS.JF421359

SYHRLRDLLL

>B.US.09.1064PLASMA21OCT2009.P4A10.KM081848

SYHRLRDLLL

>B.US.09.1670PLASMA29JUL2009.P9E17.KM081861

IYHRLRDLLL

>B.US.09.1674PLASMA02SEP2009.P15O19.KM081893

SYHRLRDLLL

>B.US.09.1675PLASMA07OCT2009.P16F5.KM081913

SYHRLRDLLL

>B.US.09.1677CDNAPBMC02DEC2009.P12O9.KM081963

SYHRLRDLLL

>B.US.09.1906CDNAPBMC21OCT2009.P8F4.KM082128

SYHRLRDLLL

>B.US.09.306501_ENV.JX864027

SYHRLRDLLL

>B.US.09.700010867_A10.KR423423

SYHRLRDLIL

>B.US.09.700010878_B5.KR423436

SYHRLRDLLL

>B.US.09.700010914_A8.KR423444

SYHHLRDLLL

>B.US.09.700010937_A10.KR423482

IYHHLRDLLL

>B.US.09.700011145_A10_Bl_dbl.HM638568

SFHRLRDLLL

>B.US.09.9036_041609_CSF_10.KM354431

SYRHLRDLLL

>B.US.09.9044_100709_CSF_16.KM354585

SYHRLRDLLL

>B.US.09.9045_100809_CSF_11.KM354670

LYHRLRDLLL

>B.US.09.A5340A06.preC.KX587204

SYHLLRDFLL

>B.US.09.A5340A12.preC.KX587391

SYHRLRDLAL

>B.US.09.C1P.GU733713

SYHRLRDLLL

>B.US.09.DEMB09US002.JX140657

SYHRLRDLLL

>B.US.09.DEMB09US003.KC473824

SYRRLRDLLL

>B.US.09.ES38.KC935958

LYHRLRDLLL

>B.US.09.HIV025_p1.JF680914

SYHRLRDLLL

>B.US.09.HIV026_p2.JF680915

SYRLLRNLLL

>B.US.09.HIV028_p1.JF680916

SYHRLRDLLL

>B.US.09.HIV029_p2.JF680917

SYHRLRDLIL

>B.US.09.LTNP1.KC935959

LYHRLRDLLL

>B.US.10.10CB4_45E6.KF526141

LYHRLRDLLL

>B.US.10.1435CDNAPBMC27JAN2010.P7I14.KM081852

SYRHLRDLLL

>B.US.10.1775CDNAPBMC28OCT2010.P13C5.KM082006

LYHHLRDLLL

>B.US.10.1825PLASMA03NOV2010.P10H8.KM082074

IYHRLRDLLL

>B.US.10.505_0686a.WG01.MG196775

SYHRLRDLLL

>B.US.10.505_0695a.WG06.MG196787

SYHRLRDLLL

>B.US.10.505_1278b.RH01.MG196997

SYHRLRDLLL

>B.US.10.505_1962a.WG03.MG197136

SYHRLRDLLS

>B.US.10.505_2483a.WG03.MG197210

IYHRLRDLLL

>B.US.10.574872.JQ182826

SYHRLRDLVL

>B.US.10.850174.JQ182910

SYHRLRDLLL

>B.US.10.9018_040810_CSF_1.KM353922

SYHRLRDLLL

>B.US.10.9048_010410_CSF_10.KM354703

SYHRLRDLLL

>B.US.10.9055_062910_CSF_10.KM354740

SYHRLRDLLL

>B.US.10.9058_072010_CSF_1.KM354781

SYHHLRDLLL

>B.US.10.9062_121610_CSF_1.KM354821

SYHRLRDLLL

>B.US.10.9063_102910_CSF_1.KM354862

SYHRLRDLLL

>B.US.10.A5340A02.preC.KX587037

LYHRLRDLLL

>B.US.10.A5340A07.preC.KX587241

SYHRLRDLLL

>B.US.10.A5340A09.preC.KX587299

SYHRLSNLLL

>B.US.10.CH0040_3_d1485_ipe032_15_08.MG900412

SYHRLRDLLL

>B.US.10.CP1.JN397365

SYHRLRDLLL

>B.US.10.C.HQ846901

SYHRLRDLLL

>B.US.10.DEMB10US001.KC473825

SYHRLRDLLL

>B.US.10.DEMB10US003.KC473826

SYHRLRDLLL

>B.US.10.DEMB10US004.KC473827

SYHRLRDLLL

>B.US.10.DEMB10US007.KC473828

SYHRLRDLLL

>B.US.10.DEMB10US009.KC473829

SYHRLRDLIL

>B.US.10.DEMB10US011.KC473830

SYHRLRDLLL

>B.US.10.Pt1_DNA_2.KU677990

SYHRLRDLLL

>B.US.10.Pt4_DNA_5.KU678074

SYHRLRDLLL

>B.US.10.VC1.JN397364

SYHRLRDLLL

>B.US.11.1853CDNAPBMC22JUN2011.P14J11.KM082106

IYHRLRDLLL

>B.US.11.1928PLASMA09MAY2011.P10O11.KM082156

SYHRLRDLLL

>B.US.11.19CB1_induced.KF526228

SYHRLRDLLL

>B.US.11.20CB4_46F1.KF526265

SYHSLRDFLS

>B.US.11.22CC9_induced.KF526312

IYHRLRDLLL

>B.US.11.23CB6_induced.KF526323

SYHRLRDLLL

>B.US.11.361974.KT124766

SYHRLRDLLL

>B.US.11.479693.KT124773

LYHRLRDLLL

>B.US.11.481811.KT124775

SYHRLRDLLL

>B.US.11.505_0071a.WG04.MG196672

SYRHLRDLLL

>B.US.11.505_0090a.RH2.MG196679

SYHRLSDLLS

>B.US.11.505_0102a.WG01.MG196689

SYRHLRDLLL

>B.US.11.505_0396a.WG8.MG196758

SYRHLRDLLL

>B.US.11.505_0724a.WG02.MG196815

SYHRLRDLLL

>B.US.11.505_0840a.WG03.MG196840

SYHRLRDLIL

>B.US.11.505_0896a.WG08.MG196846

SYHRLRDLLL

>B.US.11.505_1174a.WG06.MG196942

SYHRLRDLLL

>B.US.11.505_1730a.WG08.MG197084

IYHRLRDLLL

>B.US.11.505_1982a.WG07.MG197146

SYHRLRDLLL

>B.US.11.9040_070611_CSF_1.KM354470

SYHRLRDLLL

>B.US.11.9071_022311_CSF_6.KM354904

SYHRLRDLLL

>B.US.11.9076_061411_CSF_11.KM354962

SYHRLRDLLL

>B.US.11.9082_080311_CSF_1.KM355003

SYHHLRDLLL

>B.US.11.9083_081211_CSF_1.KM355044

SYHRLRDLLL

>B.US.11.950965.KT124809

SYHRLRDLLL

>B.US.11.A8110_1A1.KU901727

SYHRLRDLLL

>B.US.11.AMBI_CLONE.KU641402

SYHRLRDLLL

>B.US.11.CP10_3A.KF384798

SYHRLRDLLL

>B.US.11.CP12_10.KF384799

SYHRLRDLLL

>B.US.11.CP13_2.KF384800

SYHRLRDLLL

>B.US.11.CP3_6.KF384801

SYHRLRDLLL

>B.US.11.CP4_2B.KF384802

SYHRLRDLLL

>B.US.11.CP5_3A.KF384803

SYHRLRDLLL

>B.US.11.CP6_2E.KF384804

SYHRLRDLLL

>B.US.11.CP7_2B.KF384805

SYHRLRDLLL

>B.US.11.CP8_4.KF384806

SYHRLRDLLL

>B.US.11.CP9_1A.KF384807

SYHRLRDLIL

>B.US.11.DEMB11US002.KC473831

SYHLLRDLLL

>B.US.11.DEMB11US004.KC473832

SYHRLRDLLL

>B.US.11.DEMB11US006.KC473833

SYHRLRDLLL

>B.US.11.DEMB11US011.KC473834

IYHHLRDLLL

>B.US.11.DEMB11US015.KC473835

SYHRLRDLLL

>B.US.11.ES22_27.KF384808

SYHHLRDLLL

>B.US.11.ES38.JN397362

SYHRLRDLLL

>B.US.11.ES39_42.KF384810

SYHRLSDLLL

>B.US.11.ES40_24.KF384811

SYHRLRDLLL

>B.US.11.F6817_3F1.KU901765

IYHRLRDSLL

>B.US.11.N152_061511_17.KM516891

SYHRLRDFLL

>B.US.11.T4590_4A3.KU901848

SYHQLRDLIL

>B.US.11.VC3_14.KF384812

SYHRLRDLLL

>B.US.11.VC5_3.KF384813

SYHRLRDLLL

>B.US.12.10_12_SP138_41_41.KX156409

SYRQLRDLLL

>B.US.12.2302_PBEM_13.KY778473

SYHRLRDLLL

>B.US.12.231603.KT124756

SYHRLRDLLL

>B.US.12.2452_PBEM_13.KY778615

SYHRLRDLLL

>B.US.12.383086.KT124768

SYHHLRDLLL

>B.US.12.409_133_F_w02VT.MH897911

SYRHLRDLLL

>B.US.12.420_133_F_w02_10VT.MH897913

SYHRLRDLLL

>B.US.12.421_34_F_w01VT.MH897915

SYHRLRDLLL

>B.US.12.426_34_F_w09_20VT.MH897916

SYHRLRDLLL

>B.US.12.432_133_F_w18VT_WG.MH897917

IYHRLRDLLL

>B.US.12.505_0012a.WG03.MG196642

IYRQLRDLLL

>B.US.12.505_0049a.WG07.MG196653

SYHHLRDLLL

>B.US.12.505_0122a.WG06.MG196702

SYRLLRDFLL

>B.US.12.505_0332a.RH26.MG196732

SYHRLRDLLL

>B.US.12.505_0645a.WG11.MG196767

LYHRLRDLLL

>B.US.12.505_0772a.WG2.MG196819

SYHLLRDLLL

>B.US.12.505_0821a.WG06.MG196831

IYHRLRDLLL

>B.US.12.505_0829a.RH03.MG196832

SYHRLRDLLL

>B.US.12.505_0933a.RH6.MG196866

SYHRLSDLLL

>B.US.12.505_0997a.WG03.MG196879

SYHRLRDLLW

>B.US.12.505_0998a.RH31.MG196902

SYQRLRDLLL

>B.US.12.505_1012a.WG08.MG196921

SFHRLRDLLL

>B.US.12.505_1144a.WG02.MG196922

SYHRLRDLIL

>B.US.12.505_1226a.WG08.MG196952

SYHRLRDLLW

>B.US.12.505_1276a.WG03.MG196979

SYHRLRDFLL

>B.US.12.505_1295a.WG10.MG197013

SYHRLRDLLF

>B.US.12.505_1358a.WG08.MG197023

SYHRLRDLLL

>B.US.12.505_1413a.WG05.MG197046

SYHRLRDLLL

>B.US.12.505_1569a.WG11.MG197055

SYRRLRDLLS

>B.US.12.505_1672a.WG7.MG197064

IYHRLRDLLL

>B.US.12.505_1677a.WG10.MG197083

LYHRLSDLLL

>B.US.12.505_1763a.RH14.MG197113

SYHRLRDLIL

>B.US.12.505_1931a.WG01.MG197120

SYHRLRDLLL

>B.US.12.505_1993a.RH01.MG197164

LYHRLTDLLL

>B.US.12.505_2150a.WG07.MG197176

SYHRLRDLLL

>B.US.12.505_2227a.WG04.MG197187

SYHRLRDLLL

>B.US.12.505_2474a.WG06.MG197199

SYHRLRDLLL

>B.US.12.607523.KT124784

SYHRLRDLLL

>B.US.12.608647.KT124785

SYHRLRDLLL

>B.US.12.678584.KT124790

SYHRLIDLLL

>B.US.12.9096_022912_CSF_1.KM355085

SYHRLRDLLL

>B.US.12.9097_032612_CSF_1.KM355159

SYHRLRDLLL

>B.US.12.A5340A03.preC.KX587079

SYHRLRDLLL

>B.US.12.J7180_2B12.KU901792

SYHRLRDLLL

>B.US.12.K8072_2D1.KU901816

SYHRLRDLLL

>B.US.12.M01_1_1.KM986883

ICHQLRDLLL

>B.US.12.N8261_FL_CON.KU901976

SYRHLRDLLL

>B.US.12.T8107_1G7.KU901858

SYHRLRDLLL

>B.US.12.T8250_FL_CON.KU901997

SYHRLRDLAL

>B.US.13.2026_EM_39.KY766175

SYHRLRDLLL

>B.US.13.2115_EM_19.KY778345

SYRRLRDLLL

>B.US.13.2275_EM_21.KY778395

SYHRLRDLLL

>B.US.13.439_133_F_w11VT.MH897918

SYHRLRDLIL

>B.US.13.505_0280a.WG04.MG196711

SYHRLRDLLL

>B.US.13.505_0390a.WG12.MG196745

SYHRLRDLLL

>B.US.13.505_1270a.WG12.MG196964

SYHRLRDLLL

>B.US.13.505_1371a.WG10.MG197035

ICHRLRDLLL

>B.US.13.505_1958a.WG09.MG197127

SYRQLRDLLL

>B.US.13.862898.KT124796

LYHRLRDLLL

>B.US.13.ARC_1a.MK214316

LYHRLRDLLL

>B.US.13.CP02.KX505589

SYHHLRDLLL

>B.US.13.CP03.KX505616

SYHRLRDLLL

>B.US.13.CP05.KX505648

SYHRLRDLLL

>B.US.13.CP06.KX505652

SYHLLRDLLL

>B.US.13.CP07.KX505686

LYHRLRDLLL

>B.US.13.CP08.KX505695

SYHRLTDLLL

>B.US.13.CP09.KX505707

SYHRLRDLLL

>B.US.13.DEMB13US026.KU749387

SYHRLRDLLL

>B.US.13.DONOR7tCD4Rep4.B1.p7a21.KY057587

SYHRLRDLLL

>B.US.13.Donor6.B4.p6c23.KY748513

SYHRLRDLLL

>B.US.13.Donor8.B9.p5k5.KY748576

IYHRLRDLLL

>B.US.13.G4_RV_1.KT284371

SYHRLRDLLL

>B.US.13.IQA264.PBMC.SGA2.KR182173

SYHRLRDLLL

>B.US.13.IQA265.PBMC.SGA10.KR182183

LYHRLTDLLS

>B.US.13.IQA265.PBMC.SGA2.2.KR182187

LYHRLTDLLS

>B.US.13.IQA265.PBMC.SGA6.KR182191

LYRRLTDLLS

>B.US.13.IQA265.PBMC.SGA9.KR182192

LYHRLTDLLS

>B.US.13.IQA267.PBMC.SGA1.1.KR182196

SYHRLRDLLL

>B.US.13.IQA269.PBMC.SGA11.KR182242

SYRHLRDLLL

>B.US.13.IQA270.PBMC.SGA1.1.KR182306

SYHQLRDLLL

>B.US.13.IQA275.Urine.SGA2.KR182341

SYHRLRDLLL

>B.US.13.IQA276.Urine.SGA2.KR182343

SYHRLRDLLL

>B.US.13.IQA280.PBMC.SGA10.1.KR182345

SYHRLRDLLL

>B.US.13.IQA290.PBMC.SGA12.KR182409

SYHRLTDFLS

>B.US.13.IQA291.PBMC.SGA1.KR182465

SYHRLRDLLL

>B.US.13.S1492_T1_Intact.MG171201

SYHHLRDLLL

>B.US.14.2046_EM_26.KY778299

SYHRLRDLLS

>B.US.14.21.00.A3.KT986555

SYHRLSDLLL

>B.US.14.2286.KX505396

SYHLLRDSLL

>B.US.14.2443.KX505419

SYHHLRDLLL

>B.US.14.2454.KX505435

SYHRLRDLLL

>B.US.14.2529.KX505475

SYHHLRDLLL

>B.US.14.2531.KX505508

SYHRLRDLLL

>B.US.14.2A1_W4_0408TIT.H2_S1.KX027840

SYHRLRDLLL

>B.US.14.2C4_D0_0406.C12_S.KX027971

SYHRLRDLLL

>B.US.14.2C5_D0_0422.H4_S5.KX028091

SYHRLRDLLL

>B.US.14.2E2_D0_0728Pl1.B11_S.KX028321

SYHRLRDLLL

>B.US.14.2E4_D0_0721Pl4.E1e_S9.KX028508

SYRHLRDLLL

>B.US.14.CP10.KX505739

SYHRLRDLLL

>B.US.14.DEMB14US030.KY658693

SYHHLRDLLL

>B.US.14.Pt2_DNA_28.KU678026

LYHRLRDLLL

>B.US.14.Pt3_DNA_6_2.KU678060

SYHRLSDLLL

>B.US.14.Pt7_DNA_5.KU678125

SYHRLRDLLL

>B.US.14.Z258.2014_SGA3.KX595124

SYHRLTDLLL

>B.US.15.1HB1_D0_012016_F10_S11.KY323732

SYHRLRDLLL

>B.US.15.1HB2_D0_021916_E8_S93.KY323815

SYHRLRDLLL

>B.US.15.1HB3_D0_102915_F1_S61.KY323909

SYQRLRDLLL

>B.US.15.1HC2_W1_071116_A11_S28.KY324122

SYHRLRDLLL

>B.US.15.1HC3_W4_012616_C11_S78.KY324245

LYHRLSDLLS

>B.US.15.2521.KX505446

ICHRLRDSLL

>B.US.15.26.00.G4.KT986947

SYHRLRDLLL

>B.US.15.2E1_W12_TIT0408.A12e_S5.KX028240

SYHRLRDLIL

>B.US.15.3693.KX505555

SYHRLSDLLL

>B.US.15.454_34_F_w01VT.MH897920

SYHHLRDLLL

>B.US.15.A09.CD4T.C1.20B2.MH264238

SYHRLSDLLL

>B.US.15.A5340A04.w004.KX587137

SYRHLRDLLL

>B.US.15.A5340A08.w005.KX587284

SYHHLRDLLL

>B.US.15.A5340A10.w006.KX587339

SYHRLRDLLL

>B.US.15.B106.2.d.P5.D10.03.14.16.S33.KY113882

SYHRLRDLLL

>B.US.15.B115.2.d.TIT.C7.4.9.16.a4.S4.KY113738

SYHRLRDLLL

>B.US.15.B155.2.d.TIT.D12.4.8.16.a8.S8.KY113566

SYHRLRDLLL

>B.US.15.B199.2.d.P1.H12.3.31.16.S67.KY113379

SYHRLRDLLL

>B.US.15.BVP12tCD4Rep2.3.F4.p3f13.KY057390

SYHLLRDLLS

>B.US.15.DONOR2.A1.3.p7i13.KY612720

SYHHLRNLLL

>B.US.15.Donor4.F6.2.3.p7a7.KY748402

SYHHLRDLLL

>B.US.15.exc_BVP4.3.F4.p7i4.KY057518

SYHRLRDLLL

>B.US.15.p46n.KT223503

SYHRLRDLLL

>B.US.16.1HC1_W24_063016_E12_S4.KY324010

SYHRLRDLLL

>B.US.16.1HD1_W24_071216_D7_S59.KY324473

SYRHLRDLLL

>B.US.16.2609.KX505536

SYRRLTDLLS

>B.US.16.603_D14_D_P1_A11.MG196412

SYHRLRDLLL

>B.US.16.A5340A13.w004.KX587434

SYHHLRDLLL

>B.US.16.A5340A14.w006.KX587450

SYHRLSDLLS

>B.US.79.NYC4.KJ704792

SYHRLRDLLL

>B.US.81.81NY3.AY247224

SYHRLRDLLS

>B.US.83.5157_83.AY835781

SYHRLRDLLL

>B.US.83.RF_HAT3.M17451

SYHRLRDLLL

>B.US.83.SF2_LAV2_ARV2.K02007

SYRRLRDLLL

>B.US.84.5019_84.AY835779

SYHRLRDLLL

>B.US.84.84US_MNp.AY736819

SYHHLRDLLL

>B.US.84.NY5CG.M38431

SYHRLRDLLL

>B.US.84.SC14C.U90935

SYHRLRDLLL

>B.US.84.SC.M17450

SYHRLRDLLL

>B.US.84.SF33.AY352275

SYHRLTDLLL

>B.US.85.5077_85.AY835769

SYHRLRDLLL

>B.US.85.ALA1.M38430

SYHRLRDLLL

>B.US.85.Ba_L.AB221005

SYHRLRDLLL

>B.US.85.CC101_10_10.AY357406

SYHRLRDLLL

>B.US.85.IC.30048.0.E8.KX129293

SYHRLRDLLL

>B.US.85.SFMHS11.AF025758

SYHRLRDLLL

>B.US.85.SFMHS21.AF025764

SYHRLRDLLL

>B.US.85.SFMHS3.AF025751

SYRRLRDLLL

>B.US.85.WCIPR.U69584

SYHRLRDLLL

>B.US.86.5018_86.AY835778

SYHRLRDLLL

>B.US.86.5084_86.AY835775

IYHRLRDLLL

>B.US.86.5096_86.AY835749

SYHLLRDLLL

>B.US.86.5127_86.AY835774

SYHRLRDLLL

>B.US.86.ADA.AY426119

SYHRLRDLLL

>B.US.86.JRCSF.AY426125

SYHRLRDLLL

>B.US.86.SFMHS16.AF025759

SYHHLRDLLL

>B.US.86.SFMHS17.AF025760

SYHRLRDLLL

>B.US.86.SFMHS18.AF025761

SYHRLRDLLL

>B.US.86.SFMHS1.AF025749

SYHRLRDLLL

>B.US.86.SFMHS2.AF025750

SYHRLRDLLL

>B.US.86.SFMHS4.AF025752

SYHRLRDLLL

>B.US.86.SFMHS8.AF025756

NYHRLRDLLL

>B.US.86.YU_2.M93258

SYHRLRDLLL

>B.US.87.5113_87.AY835758

IYHHLRNLLL

>B.US.87.BC_BCSG3.L02317

SYHRLRDLIL

>B.US.87.SFMHS5.AF025753

SYHRLRDLLL

>B.US.87.SFMHS7.AF025755

SYHRLRDLLL

>B.US.87.SFMHS9.AF025757

SYHRLRDLLL

>B.US.88.5160_88.AY835763

SYHRLRDLLL

>B.US.88.SFMHS19.AF025762

SYHRLRDLLL

>B.US.88.SFMHS6.AF025754

SYHRLRDLLL

>B.US.88.WR27.AF286365

SYHRLRDLLL

>B.US.89.1989b3.HQ110679

SYHRLRDLLL

>B.US.89.P896_89_6.U39362

LYHLLRNLLL

>B.US.89.R2_envUS_R2.AF128126

SYHRLRDLLL

>B.US.89.SFMHS20.AF025763

SYHRLRDLLL

>B.US.90.100711i.3.5.MH012793

SYHRLRDLLL

>B.US.90.90US_873.AY713412

SYHRLRDLLL

>B.US.90.BORId9_4A9.EU576292

SYHRLRDLLL

>B.US.90.M1001_x_M10.KT283697

SYHRLRDLIL

>B.US.90.US1.AY173952

SYHRLRDLLL

>B.US.90.US2.AY173953

SYHRLRDLLL

>B.US.90.US3.AY173954

SYHRLRDLLL

>B.US.90.US4.AY173955

SYHRLRDLLL

>B.US.90.WEAU160_GHOSH.U21135

LYHRLIDLLL

>B.US.91.100307i.2.18.MH012589

LYHRLRDLLL

>B.US.91.100383i.3.22.MH012650

SYHRLRDLLS

>B.US.91.100890i.2.1.MH012806

SYHRLRDLLL

>B.US.91.100997i.2.18.MH012866

SYRQLRGLLL

>B.US.91.101421i.2.11.MH012925

LYHRLRDLLL

>B.US.91.101984i.21.MH012971

IYHRLRDLLL

>B.US.91.5048_91.AY835761

SYHRLRDLLL

>B.US.91.DH12_3.AF069140

SYHRLRDLLL

>B.US.91.HOBR0961_A10.GU331649

SYHRLRDLLL

>B.US.91.R3A.AY608577

SYHRLRDLLL

>B.US.91.SUMA_TF1.JN944928

SYHRLRDLLL

>B.US.92.100155m.10.MH012542

SYHRLRDLLL

>B.US.92.102605i_10.MH013129

SYHRLTDLLL

>B.US.92.92US657_1_301657_1.U04908

ICHRLRDLLL

>B.US.92.M1002_x_AC4.KT283706

LYHRLRDLLW

>B.US.93.100002i.2.28.MH012332

SYHRLRDLLL

>B.US.93.100014i.3.15.MH012400

SYHRLRDLLL

>B.US.93.100046i.3.10.MH012257

IYHRWRDLLL

>B.US.93.100052i.2.24.MH012478

SYHRLRDLLL

>B.US.93.102149i.2.1.MH013027

SYHRLRDLLL

>B.US.93.102407i.2.1.MH013075

SYHRLRDLLL

>B.US.93.WCD32P0793.DQ487188

SYHRLTDLLL

>B.US.93.WCM32P0793.DQ487190

SYHRLTDLLL

>B.US.94.5082_94.AY835773

SYHRLRDLLL

>B.US.94.6101.AY612855

SYHRLRDLLL

>B.US.94.7165_18.AY835437

SYHRLRDLLL

>B.US.94.94US_33931N.AY713410

SYHHLRDSLL

>B.US.94.M1003_x_D6.KT283729

SYHRLRDLLL

>B.US.94.MACS4.DQ313246

SYHRLRDLLL

>B.US.94.PRB926_04_A9_4237.EU289197

SYHRLRDLLL

>B.US.95.1_95TC14.JQ609870

SYHRLRDLLL

>B.US.95.3988_25.AY835436

SYHRLRDLLL

>B.US.95.5073_95.AY835768

SYHRLRDLLL

>B.US.95.5768_4.AY835435

SYHRLRDLLL

>B.US.95.6240_08_TA5_4622.EU289190

SYHRLRDLLL

>B.US.95.6535_3.AY835438

SYHRLRDLLL

>B.US.95.M1007_x_Q6.KT283753

SYHRLRDLLL

>B.US.95.PRB931_06_TC3_4930.EU289198

SYHRLRDLLL

>B.US.95.Pt9_1995_9.KU678164

SYHRLRDLLL

>B.US.95.USPI90770EI72y95091pcWG2B7.JN024303

SYHRLRDLLL

>B.US.96.1027_03.AY332237

SYHLLRDFLL

>B.US.96.1057_01.AY331292

IYHRLRDLLS

>B.US.96.1304_d31.AY308762

IYHRLRDLLL

>B.US.96.5155_96.AY835753

SYHRLRDLLL

>B.US.96.61792_03_p29.EU575474

SYRRLRDLLL

>B.US.96.6244_13_B5_4576.EU289191

SYHRLRDLLL

>B.US.96.62615_03_p10.EU575593

IYHRLRDLLL

>B.US.96.C26_12_1BH.U84819

SYHRLRDLLL

>B.US.96.USPI55751EI32y96071pcWG2B15.JN024428

SYHRLRDFLL

>B.US.97.1001_07.AY331282

SYHHLRDLLL

>B.US.97.1006_08.AY331284

LYHRLRDLLL

>B.US.97.1012_08.AY331285

SYHRLRDLLL

>B.US.97.1013_03.AY331287

SYHRLRDLLL

>B.US.97.1018_06.AY331289

SYHRLRDLLL

>B.US.97.1051_11.KT124744

IYQSLRDLLL

>B.US.97.1053_06.KT124745

SYHRLRDLLL

>B.US.97.1054_TC4_1499.EU289185

IYHRLRDLLL

>B.US.97.395718.KT124770

SYHRLRDLLL

>B.US.97.4012_C11.JN562755

SYHHLRDLLL

>B.US.97.4013_C11.JN562794

IYHRLRDLLL

>B.US.97.5002_P10.JN562786

SYRHLRDLLL

>B.US.97.5003_P33.JN562773

SYHRLRDLLL

>B.US.97.62357_14_D3_4589.EU289189

SYHRLRDLLL

>B.US.97.624708_SGA_D14.EF593200

SYHRLRDLLL

>B.US.97.6305404_SGA_TA3.EF593201

SYHRLRDLLL

>B.US.97.63068_05_A13.EU575670

SYHRLRDLLL

>B.US.97.63215_03_p20.EU575697

GYHHLRDLLL

>B.US.97.63358_p3_4013.EU289192

SYHRLRDLLL

>B.US.97.901009_SGA_B3.EF593204

SYHHLRDLLL

>B.US.97.9014_01_TB1_4769.EU289195

SYHQLRDFLL

>B.US.97.9015_07_B8.EU575815

LYQSLRDLLL

>B.US.97.9019_03_p10.EU575850

SYHRLRDLLL

>B.US.97.9022_09_p10.EU575927

SYHRLTDLLL

>B.US.97.M02_3_SW.U84854

SYHRLRDLLL

>B.US.97.PRB95604_SGA_A4.EF593232

SYHRLRDLLL

>B.US.97.SS1196_1.AY835442

SYHRLRDLLL

>B.US.97.ZP6248_07_3A1.JN400469

SYHRLRDLLL

>B.US.97.zp62995_p2.EF593304

CYHRLRDLLL

>B.US.97.zp9024_p17.EF593311

LYHHLRDLVL

>B.US.98.1056_TA11_1826.EU289186

IYHSLRDLLL

>B.US.98.1058_08.AY331294

LYHRLRDLLL

>B.US.98.1059_09.KT124746

SYHRLRDLLL

>B.US.98.119534.KT124747

SYHRLRDLLL

>B.US.98.15384_1.DQ853463

SYHRLRDLLL

>B.US.98.394242.KT124769

SYHRLRDLLL

>B.US.98.902020_SGA_A11.EF593208

SYHLLRDLLL

>B.US.98.9021_14_B2_4571.EU289196

SYHRLRDLLL

>B.US.98.9026_07_p16.EU576016

SYHRLRDLLL

>B.US.98.9032_08_B33.EU576141

LYHRLRDLLL

>B.US.98.98USHVTN1925c1.AY560107

SYHRLRDLLL

>B.US.98.98USHVTN3605c9.AY560108

LYHRLRDLLL

>B.US.98.98USHVTN8229c6.AY560109

SYHRLRDLLL

>B.US.98.98USHVTN941c1.AY560110

SYHRLRDLLL

>B.US.98.AC10_29.AY835446

SYHHLRDLLL

>B.US.98.WC3_0498_4.EF175212

SYHHLRDLIL

>B.US.98.Z02_C6.EF593282

SYHRLRDLLL

>B.US.98.Z03_B10.EU577426

SYHRLRDLLL

>B.US.98.Z05_D6.EF593284

SYHRLRDLLL

>B.US.98.zp9023_p5.EF593310

SYRHLRDLIL

>B.US.98.zp9025_p9.EF593312

SYHRLRDLLL

>B.US.98.zp9028_p5.EF593314

SYHRLRDLLL

>B.US.98.zp9030_p19.EF593316

TYHRLRDLLL

>B.US.98.zp9033_p5.EF593318

IYHSLRDLLL

>B.US.99.10017SP14_10_73_PRO.JN786786

SYHRLRDLLL

>B.US.99.12008_09_A7.EU575441

SYHRLRDLLL

>B.US.99.169_1999.JN599165

SYHRLTDLLL

>B.US.99.18_99TA3.JQ610043

SYHSLRDLLL

>B.US.99.284160.KT124759

LYHRLTDLLL

>B.US.99.4030_C11.JN562762

SYHRLRDLLL

>B.US.99.489910.KT124776

SYHRLRDLLL

>B.US.99.7766SP15_39_41_EPI.JN786752

SYHRLRDLLL

>B.US.99.868558.KT124798

SYHRLRDLLL

>B.US.99.907712_SGA_TA1.EF593212

SYRHLRDLIL

>B.US.99.9079_09_TA10.EU576268

SYHRLRDLLL

>B.US.99.AD17_3C_TA4.GU331247

SYHRLRDLLL

>B.US.99.CA110_SP14_27_1.JN786831

SYHRLRDLLS

>B.US.99.HIV_US_BID_V5239_1999.JQ403100

SYHRLRDLLL

>B.US.99.PRB957_06_A13.EU576617

SYHRLRDLLL

>B.US.99.PRB959_03.AY331296

SYHRLRDLLL

>B.US.99.Z10_B13.EU577469

SYRHLRDLLL

>B.US.99.Z13_G7.EF593286

SYHRLRDLLS

>B.US.99.Z16_G28.EU577525

IYHRLRDLLL

>B.US.99.zp12007_p27.EF593300

SYHRLRDFLL

>B.US.x.108048_002.JQ085297

SYHRLRDLLL

>B.US.x.108060.GQ153937

IYHRLRDLLW

>B.US.x.108069_005.JQ085295

SYHHLRDLLL

>B.US.x.109_24.FJ798397

SYHRLRDLLL

>B.US.x.1229I.FJ469685

SYHRLRDLLL

>B.US.x.135_10.FJ798416

SYHHLRDLLL

>B.US.x.172297_028.JQ085287

SYHRLRDLLL

>B.US.x.172946_025.JQ085289

SYHRLRDLLL

>B.US.x.172976_032.JQ085293

LYHRLRDLLL

>B.US.x.546BMB4_546BM_B4.AF217150

SYHRLRDLLL

>B.US.x.601_D14_2E_19.MH262735

SYHRLRDLLL

>B.US.x.605_W29_h8.MH263121

SYHRLRDLLL

>B.US.x.608_D14_MI10.MH263144

SYHRLRDLLL

>B.US.x.609_D14_12_11.MH263194

SYHLLRDLLL

>B.US.x.611_D14_MH7.MH263362

SYHRLRDLLL

>B.US.x.613_W23_MAB_12.MH263520

SYHHLRDLLL

>B.US.x.616_W23_MM_15.MH263630

IYHRLRDLLL

>B.US.x.701.s000.10.KX984368

SYHRLRDLTL

>B.US.x.702.s000.10.KX984437

LYHRLRDLLL

>B.US.x.703.w005.p.12.A6_S79.KX984537

LYHSLRDLLL

>B.US.x.704.s000.1.KX984588

SYRHLRNLLL

>B.US.x.7047C_c01.AY842786

SYHRLRDLLL

>B.US.x.7069C_c02.AY842808

SYHRLRDLPL

>B.US.x.707.w009.p.B12_S49.KX984699

SYHRLRDLIL

>B.US.x.708.s000.2C11_S79.KX984753

SYHRLIDLLL

>B.US.x.711.s000.c.G1_S53.KX984877

SYHRLRDLLL

>B.US.x.7120C_c01.AY842824

SYHRLRDLLL

>B.US.x.91C22_D0_26_11_B5_S82.MH632763

TYHRLRDLLL

>B.US.x.91C33_D0_9_4_c9_S49.MH632949

SYHHLRDLLL

>B.US.x.91C34_D0_YBAc10_S26.MH632956

SYHRLRDLLL

>B.US.x.91C35_D0_3HG_010517_D6_S46.MH632822

SYHRLRDLLL

>B.US.x.9241_pre_L8.MH575991

SYHHLRDLIL

>B.US.x.9242_W12_12H20.MH576349

SYHHLRDLLL

>B.US.x.9243_W12_12E19.MH576262

SYHRLRDLLL

>B.US.x.9244_rebound_S21_G8.MH576152

SYHHLRDLLL

>B.US.x.9245_pre_H6.MH576107

LYHRLRDLLL

>B.US.x.9246_W12_12K21.MH576084

SYHRLSDLLL

>B.US.x.9247_pre_A1.MH576039

LCHRLRDLLL

>B.US.x.9248_pre_B8.MH575857

LYHRLRDLLL

>B.US.x.9249_pre_G3.MH575796

LYHRLRDLLL

>B.US.x.9250_pre_F9.MH575728

SYHRLRDLLL

>B.US.x.9251_rebound_S60_H12.MH575694

SYHHLRDFLL

>B.US.x.9252_rebound_S71_D12.MH575680

SYHRLRDLLL

>B.US.x.9254_W12_12BL5.MH575478

SYRRLRDLLL

>B.US.x.9255_W12_12C4.MH575379

SYHRLRDLLL

>B.US.x.9341_D0_1A_1118G10_S87.MH633039

SYHRLRDLLL

>B.US.x.9341_D0_1_2812A3_S72.MH633031

SYHRLRDLLL

>B.US.x.9342_D0_2_2812a6_S1.MH633155

SYHHLRDLLL

>B.US.x.9342_D0_2_3012b10_S11.MH633164

SYHRLRDLLL

>B.US.x.9343_D0_3_2812_A2_S55.MH633199

SYHRLRDLLL

>B.US.x.A2520TOB8U.GU727873

SYHRLRDLLL

>B.US.x.A3533TOB8U.GU728308

SYHRLRDLLL

>B.US.x.A4521TOB8U.GU728201

SYHRLRDLLL

>B.US.x.A7540TOB8U.GU728333

SYHRLRDLLL

>B.US.x.A8524TOB8U.GU728279

SYHRLRDLLL

>B.US.x.AC160_T9_Day_1034_Dom.EU616649

SYHRLRDLLL

>B.US.x.AC_04_0_Days_Consen_fa.DQ127534

SYHRLRDLLL

>B.US.x.AC_16_0_Days_Consen_fa.DQ127537

SYHRLRDLLL

>B.US.x.AC_59_41_Days_Seq1_fa.DQ127548

SYRRLRDLLL

>B.US.x.AD3.v6.AF219627

SYHRLRDLLL

>B.US.x.AD358_m2.KT808396

SYHRLRDLLL

>B.US.x.B0519TOB8U.GU727957

SYHRLRDLLL

>B.US.x.B1539TOB8U.GU728244

LYHRLRDLLL

>B.US.x.B1542TOB8U.GU728346

SYHRLRDLLL

>B.US.x.B3535TOB8U.GU728216

IYHSLRNLLL

>B.US.x.B4522TOB8U.GU728253

SYHRLRDLLL

>B.US.x.B4578TOB8U.GU728290

LYHRLRDLLL

>B.US.x.B6515TOB8U.GU728376

SYHRLRDLLL

>B.US.x.B7500TOB8U.GU728327

SYHRLRDLLL

>B.US.x.B8526TOB8U.GU728163

SYHRLRDLVL

>B.US.x.B9517TOB8U.GU727884

LYHLLRDLLL

>B.US.x.B9543TOB8U.GU727890

SYHRLRDLLX

>B.US.x.BRVA.M21098

SYHRLRDLLL

>B.US.x.CR0036W.FJ469690

SYHRLRDLLL

>B.US.x.CR0050Z.FJ469692

SYLRLRDLLL

>B.US.x.CR0059T.FJ469694

SYHRLRDLLL

>B.US.x.CR0127W.FJ469699

SYHRLRDLLL

>B.US.x.CR0164U.FJ469702

SYHRLRDLLL

>B.US.x.CR0192W.FJ469704

SYHRLTDLLL

>B.US.x.CR0206U.FJ469705

SYHRLRDLLL

>B.US.x.CR0234.FJ469711

SYHRLRDLLL

>B.US.x.CR0248X.FJ469712

IYHLLRDSLL

>B.US.x.CR0317N.FJ469719

SYQRLRDLLL

>B.US.x.CR0382N.FJ469725

SYRRLRDLLL

>B.US.x.CR0413T.FJ469726

SYHRLRDLLL

>B.US.x.D3532TOB8U.GU728276

SYHRLRDLLX

>B.US.x.D4535TOB8U.GU728225

LYHRLRDLLL

>B.US.x.D8520TOB8U.GU728277

SYHRLRDLLL

>B.US.x.DS_br_13.EU850430

IYHRLRDLLL

>B.US.x.E0510TOB8U.GU728412

SYHRLRDLLL

>B.US.x.E2514TOB8U.GU728293

SYHRLRDLLL

>B.US.x.E6537TOB8U.GU727880

SYHRLRDLLL

>B.US.x.E7538TOB8U.GU728304

SYHRLRDLLL

>B.US.x.E9516TOB8U.GU728410

SYHRLRDLLL

>B.US.x.E9534TOB8U.GU728053

LYHRLRDLLL

>B.US.x.F1531TOB8U.GU728295

SYHRLRDLLL

>B.US.x.F1540TOB8U.GU728134

SYHRLRDLLL

>B.US.x.F5510TOB8U.GU728383

SYHHLRDLLL

>B.US.x.F703.DQ886031

SYRHLRDLLL

>B.US.x.F710.FJ469729

SYHRLRDLLL

>B.US.x.F7174.DQ886032

SYHRLGDLLL

>B.US.x.F7204.DQ886033

SYHRLRDLLL

>B.US.x.F7509TOB8U.GU727898

SYHHLRDLLL

>B.US.x.G2535TOB8U.GU728005

SYHRLRDLLL

>B.US.x.G3518TOB8U.GU728357

SYHRLSDLLL

>B.US.x.G8521TOB8U.GU728280

SYHRLRDLLL

>B.US.x.G8598TOB8U.GU728339

SYHRLRDLLL

>B.US.x.H0002GH.DQ222211

SYHRLRDLLL

>B.US.x.H4570TOB8U.GU728205

SYRHLRDLLL

>B.US.x.H5519TOB8U.GU727913

SYHRLRDLLS

>B.US.x.H9514TOB8U.GU728268

SYHRLIDLLL

>B.US.x.HCPI_01_02.GU367409

XYHRLRDLLL

>B.US.x.HCPI_02_01.GU367407

SYHRLRDLLL

>B.US.x.HCPI_03_01.GU367410

IYHHLRDFLL

>B.US.x.HCPI_06_01.GU367408

SYHRLRDLLL

>B.US.x.HCPI_12_01.GU367399

IYHRLRDLLL

>B.US.x.HCPI_15_02.GU367403

SYHRLRDSLL

>B.US.x.HCPI_18_01.GU367405

SYHRLRDFLL

>B.US.x.HP003A.KP754463

SYHRLIDLLL

>B.US.x.HP013A.KP754464

SYHRLRDLLL

>B.US.x.HP015A.KP754465

SYRHLRDLLL

>B.US.x.HP022A.KP754466

SYHRLRDLLL

>B.US.x.HP024A.KP754467

SYHRLTDLLL

>B.US.x.HP025B.KP754468

TYHRLRDLLL

>B.US.x.HP029K.KP754469

SYHRLRDLLL

>B.US.x.HP038C.KP754470

SYHRLRDLLL

>B.US.x.HP042K.KP754471

SYHRLRDLLL

>B.US.x.HP043C.KP754472

SYHRLRDLLL

>B.US.x.HP051B.KP754473

SYHRLRDLLL

>B.US.x.INMEd6_1.DQ444258

SYHRLRDLLL

>B.US.x.J1526TOB8U.GU728371

SYHRLRDLLL

>B.US.x.J3544TOB8U.GU728348

SYHRLRDLLL

>B.US.x.J4540TOB8U.GU728322

SYHRLRDLLL

>B.US.x.J8534TOB8U.GU728025

SYHRLRDLLL

>B.US.x.J9520TOB8U.GU728227

SYHRLRDLLL

>B.US.x.JHU559_FDC_ccG2S.EF440778

SYHRLRDLLL

>B.US.x.K0516TOB8U.GU728404

SYRHLRDLLL

>B.US.x.K1573TOB8U.GU728137

SYHRLRDLLL

>B.US.x.K4511TOB8U.GU728263

SYHRLRDLLL

>B.US.x.K6570TOB8U.GU728152

SYRHLRDLLL

>B.US.x.K7576TOB8U.GU728195

SYHRLRDLLL

>B.US.x.K9548TOB8U.GU728056

IYHRLRDLLL

>B.US.x.L0576TOB8U.GU728368

SYHRLRDLLL

>B.US.x.L3516TOB8U.GU728392

SYHRLRDLLL

>B.US.x.L4530TOB8U.GU728072

SYHQLRNLIL

>B.US.x.L4548TOB8U.GU728155

SYHRLRDLLL

>B.US.x.L6514TOB8U.GU727887

LYHRLRDLLL

>B.US.x.L7510TOB8U.GU728352

SYHRLRDLLL

>B.US.x.L7549TOB8U.GU728209

SYHRLRDLLL

>B.US.x.L8146.DQ886034

SYHRLRDLLL

>B.US.x.L8157.DQ886035

SYHRLRDLLS

>B.US.x.L8185.DQ886036

SYHRLRDLLL

>B.US.x.L8188.FJ469744

SYHRLRDLLL

>B.US.x.L8574TOB8U.GU728150

SYHSLRDLLL

>B.US.x.L896.FJ469750

SYHRLIDLLL

>B.US.x.M0513TOB8U.GU728413

SYRHLRDLVL

>B.US.x.M1504TOB8U.GU728313

SYHRLRDLLL

>B.US.x.M1546TOB8U.GU728345

SYRHLRDLLL

>B.US.x.M4526TOB8U.GU728179

SYHRLRDLLL

>B.US.x.M6509TOB8U.GU727958

SYHRLRDLLS

>B.US.x.M7573TOB8U.GU728238

SYHRLRDLLL

>B.US.x.M9512TOB8U.GU728409

SYHRLRDLLL

>B.US.x.MACS2BR_13.AF491737

SYHRLRDLLL

>B.US.x.Macs1_SPLN_15.FJ687543

SYHRLRDLLL

>B.US.x.Macs3ln8.JN002043

SYHRLRDLLL

>B.US.x.N1505TOB8U.GU728094

SYHQLRDLLL

>B.US.x.N4549TOB8U.GU728337

LYHRLRDLLS

>B.US.x.N7525TOB8U.GU727886

SYHRLRDLLL

>B.US.x.N8517TOB8U.GU728349

SYHRLRDLLL

>B.US.x.NC7.AF049495

SYHRLRDLLL

>B.US.x.NCQ.FJ469752

LYHRLRDLLL

>B.US.x.O1529TOB8U.GU728041

SYHRLRDFLL

>B.US.x.O5532TOB8U.GU727972

SYHRLRDLLL

>B.US.x.O5858TOB8U.GU728416

SYHRLRDLLL

>B.US.x.O6535TOB8U.GU728101

SYHRLRDLLL

>B.US.x.O9545TOB8U.GU728051

SYHRLRDLLX

>B.US.x.O9574TOB8U.GU728189

SYHRLRDLLL

>B.US.x.P0514TOB8U.GU728341

SYHRLRDXLX

>B.US.x.P1049_1_E1.KU252650

SYHRLRDLLL

>B.US.x.P1534TOB8U.GU728294

SYHRLRDLLL

>B.US.x.P5594TOB8U.GU728191

SYHRLRDLLS

>B.US.x.P7574TOB8U.GU728240

SYHRLRDLLL

>B.US.x.PRLS05.FJ469756

SYHHLRDLLL

>B.US.x.PRLS17.FJ469761

SYHRLRDLLL

>B.US.x.PRLS19.FJ469763

SYHRLRDLLL

>B.US.x.PRLS28.FJ469766

SYHRLRDLLL

>B.US.x.Q0508TOB8U.GU728176

CYHRLRDLLL

>B.US.x.Q2524TOB8U.GU727943

SYHRLRDLLL

>B.US.x.Q4533TOB8U.GU728073

SYRHLRDLLL

>B.US.x.Q7513TOB8U.GU728158

SYHRLRDLLX

>B.US.x.Q8571TOB8U.GU728151

LYHRLRDLLL

>B.US.x.R0500TOB8U.GU727977

SYHRLRDLLL

>B.US.x.R5508TOB8U.GU728343

SYHQLRDLLL

>B.US.x.R5520TOB8U.GU728222

SYHRLRDLLL

>B.US.x.R6526TOB8U.GU727875

SYHRLRDSLL

>B.US.x.R9577TOB8U.GU728323

SYHRLRDLLL

>B.US.x.S1532TOB8U.GU728234

SYHRLRDLLL

>B.US.x.S2513TOB8U.GU728287

SYHRLRDLLL

>B.US.x.S3526TOB8U.GU728039

SYHRLRDLLL

>B.US.x.S6536TOB8U.GU727879

SYHRLRDLLL

>B.US.x.S7508TOB8U.GU727988

SYHRLRDLLL

>B.US.x.S8511TOB8U.GU728271

SYHRLRDLLL

>B.US.x.SF128.M95292

SYHRLRDLLL

>B.US.x.SF162_control_passage_12.EF367179

SYHRLRDLIL

>B.US.x.SH8127.FJ469767

SYHRLRDLLL

>B.US.x.SH8183.FJ469768

SYHRLRDLLL

>B.US.x.SH8229.FJ469769

SYHQLRDLIL

>B.US.x.SH8241.FJ469771

SYHRLRDLLL

>B.US.x.T0548TOB8U.GU728359

SYHRLRDLLL

>B.US.x.T1520TOB8U.GU728204

SYHRLRDLLS

>B.US.x.T3511TOB8U.GU728406

SYHRLRDLLL

>B.US.x.T4544TOB8U.GU728311

SYHRLRDLLL

>B.US.x.T5529TOB8U.GU728223

SYHRLRDLLL

>B.US.x.T5570TOB8U.GU728030

SYHRLRDLLL

>B.US.x.T8510TOB8U.GU728157

CYRHLRDLLL

>B.US.x.T9524TOB8U.GU728229

SYHRLRDLLL

>B.US.x.TYBE.AY189526

SYRHLRDLLL

>B.US.x.V1528TOB8U.GU728370

SYHRLRDLLL

>B.US.x.V4547TOB8U.GU728321

IYHRLRDLLL

>B.US.x.V8518TOB8U.GU728336

SYHHLRDLLL

>B.US.x.V9527TOB8U.GU728226

GYHLLRDLLL

>B.US.x.V9576TOB8U.GU728185

SYHRLRDLLL

>B.US.x.W0504TOB8U.GU727980

SYHRLRDLLL

>B.US.x.W0515TOB8U.GU728297

SYHHLRDLLL

>B.US.x.W4541TOB8U.GU728334

SYHRLRDLLL

>B.US.x.W7526TOB8U.GU727917

IYHHLRDLLL

>B.US.x.W9532TOB8U.GU728283

CYHRLRDLLL

>B.US.x.X1537TOB8U.GU728237

SYHRLRDLLL

>B.US.x.X2519TOB8U.GU728288

SYRRLRDLLL

>B.US.x.X3578TOB8U.GU728054

LYHRLRDLLL

>B.US.x.X5515TOB8U.GU728181

SYHRLRDLLL

>B.US.x.X5533TOB8U.GU728050

SYRHLRDLLL

>B.US.x.X6521TOB8U.GU727919

SYHRLRDLLL

>B.US.x.X9542TOB8U.GU728014

SYHRLRDLLL

>B.US.x.Y1525TOB8U.GU728369

LYHRLRDLLL

>B.US.x.Y2579TOB8U.GU728255

SYHRLRDLLS

>B.US.x.Y3517TOB8U.GU728395

SYHRLRDLLL

>B.US.x.Y4501TOB8U.GU728093

SYHRLRDLLL

>B.US.x.Z2576TOB8U.GU728396

SYHRLRDLLL

>B.US.x.Z7547TOB8U.GU728330

SYHRLRDLLL

>B.US.x.Z8529TOB8U.GU728274

IYHRLRDLLL

>B.US.x.s33_d39_c31.EU604640

SYHRLRDLLL

>B.US.x.s35_d0_c23.EU604556

SYHRLRDLLL

>B.US.x.sample_C_BID_D617.JX503075

SYHRLRDLLL

>B.UY.01.01UYTRA1092.AY781126

SYHLLRDLLL

>B.UY.01.01UYTRA1179.AY781127

SYHRLRDLLL

>B.UY.02.02UY_TSU1290.JN235958

SYHRLRDLLL

>B.UY.99.99UY_TRA0177.JN235965

SYHRLRDLLL

>B.YE.02.02YE507.AY795904

SYRHLRDLLL

>B.YE.02.02YE508.AY795905

IYRHLRDLLL

>B.ZA.00.TV047.KJ948657

SYHRLRDLLL

>B.ZA.02.TV1057.KJ948660

SYHRLRDLLL

>B.ZA.03.03ZAPS045MB2.DQ396398

SYHRLRDLLL

>B.ZA.09.21231341.HQ595755

SYHRLRDLLL

>B.ZA.09.DEMB09ZA022.KP109515

SYHRLRDLLL

>B.ZA.09.MSM071.KF725915

SYHRLRDLLL

>B.ZA.09.MSM082.KF725918

SYRHLRDLLL

>B.ZA.10.MSM186.KF725946

SYHHLRDLLL

>B.ZA.10.MSM249.KF725977

SYRHLRDLLL

>B.ZA.10.MSM255.KF725981

LYHRLRDLLL

>B.ZA.10.MSM256.KF725982

SYHRLRDLLL

>B.ZA.10.MSM262.KF725985

TYHRLRDLLL

>B.ZA.10.MSM263.KF725986

SYHHLRDLLL

>B.ZA.10.MSM265.KF725988

SYHRLRDLLL

>B.ZA.10.MSM284.KF726002

SYHRLRDLLL

>B.ZA.10.MSM285.KF726003

SYHRLRDLLL

>B.ZA.10.MSM286.KF726004

SYHRLRDLLL

>B.ZA.10.MSM293.KF726008

SYHRLRDLLL

>B.ZA.10.MSM294.KF726009

SYHRLRDLLL

>B.ZA.10.MSM304.KF726016

IYHRLRDLLL

>B.ZA.10.MSM306.KF726018

CYHLLRNLIL

>B.ZA.10.MSM310.KF726021

LYHRLRDLLW

>B.ZA.10.MSM316.KF726023

SYHRLRDLLL

>B.ZA.85.R68.MH234643

IYHRLRDLLL

>B.ZA.85.R84.FJ647145

SYHRLRDLLL

>B.ZA.86.R1296.MH234639

SYHRLRDFLL

>B.ZA.87.R459.MH234640

SYHRLRDLLL

>B.ZA.99.99ZASM1.AY505010

LYHHLRDLLL

>B.x.00.LA02FolC.KU168257

SYHRLRDLLL

>B.x.02.LA03BlEr.KU168258

LYHRLRDLIL

>B.x.02.LA04GuFu.KU168259

LYHRLGDLLL

>B.x.03.LA05MeAl.KU168260

SYHRLRDLLL

>B.x.09.P430_p3.JF680931

SYHRLRDLLL

>B.x.10.DEURF10HA002.KY658703

SYHHLRDLLL

>B.x.x.17Sens.KC834604

SYHRLRDLLL

>B.x.x.24Res.KC834602

SYHRLRDLLL

>B.x.x.Pat10day1clone11.KT452465

SYHRLRDLLL

>B.x.x.Pat11day1clone3.KT452481

SYHRLRDLLL

>B.x.x.Pat12day1clone11.KT452273

SYHRLRDLLL

>B.x.x.Pat13day1clone4.KT452314

SYHRLRDLLL

>B.x.x.Pat14day1clone6.KT452508

SYHRLRDLLL

>B.x.x.Pat15day1clone7.KT452341

SYHRLRDLLL

>B.x.x.Pat16day353clone25.KT452551

SYRHLRDLLL

>B.x.x.Pat17day117clone34.KT452596

CYHRLTDLLL

>B.x.x.Pat18day107clone14.KT452612

SYHRLRDLLL

>B.x.x.Pat19day1clone1.KT452359

SYHRLRDLLL

>B.x.x.Pat1day1clone2.KT452109

IYHRLRDLLL

>B.x.x.Pat20day1clone7.KT452090

SYHRLRDLLL

>B.x.x.Pat2day156clone13.KT452155

SYHRLRDLLS

>B.x.x.Pat4day1clone1.KT452407

SYHHLRDLLL

>B.x.x.Pat6day1clone4.KT452194

SYHRLRDLLL

>B.x.x.Pat7day1clone4.KT452218

SYHRLRDLLL

>B.x.x.Pat9day1clone7.KT452245

SYHHLRDLLL

>C.AR.01.ARG4006.AY563170

SYRQLRDLIL

>C.BE.93.VI882.HQ912708

SYHRLRDLTL

>C.BE.x.VI829_1.EU191613

SYHRLRDFIL

>C.BI.91.BU910112.U39233

SYHRLRDLIL

>C.BI.91.BU910213.U39237

SYHRLRDLLL

>C.BI.91.BU910316.U39239

SYHRLRNLIL

>C.BI.91.BU910423.U39243

CYHRLRDLLL

>C.BI.91.BU910518.U39240

SYRRLRDLLL

>C.BI.91.BU910611.U39246

CYHRLRDLLL

>C.BI.91.BU910717.U39245

SYRRLRDLVL

>C.BI.91.BU910812.U39251

LYHRLRDLLL

>C.BR.02.02BR2022.JN692434

SYHRLRDLIL

>C.BR.04.04BR013.AY727522

SYHRLRDLIL

>C.BR.04.04BR021.AY727523

SYHRLRDLLL

>C.BR.04.04BR038.AY727524

SYHRLRDLIL

>C.BR.04.04BR073.AY727525

IYHRLRDLLL

>C.BR.07.DEMC07BR003.JX140663

SYHRLRDFLL

>C.BR.09.2009SCNEUT46.KX181928

SYHRLRDLTL

>C.BR.09.2009SCNEUT47.KX181929

SYHQLRDLTL

>C.BR.09.2009SCNEUT48.KX181930

SYHRLRDLIL

>C.BR.09.2009SCNEUT49.KX181931

SYHRLRDLIS

>C.BR.09.2009SCNEUT50.KX181932

SYHRLRDLIS

>C.BR.09.2009SCNEUT51.KX181933

SYHRLRDLTL

>C.BR.09.2009SCNEUT53.KX181934

SYHRLRDLIL

>C.BR.09.2009SCNEUT54.KX181935

SYHRLRDLIL

>C.BR.09.2009SCNEUT55.KX181936

SYRRLRDLIL

>C.BR.09.2009SCNEUT56.KX181937

SYHRLRDLVL

>C.BR.09.2009SCNEUT57.KX181938

SYHRLRDLIL

>C.BR.09.2009SCNEUT58.KX181939

IYHRLRDLIL

>C.BR.09.2009SCNEUT59.KX181940

CYHRLRDLIL

>C.BR.09.2009SCNEUT60.KX181941

SYHRLRDLLL

>C.BR.09.DEMC09BR036.KU749391

CYHRLRDLAL

>C.BR.10.10BR_MG032.KT427678

SYHRLRDLLL

>C.BR.10.10BR_MG040.KT427674

SYHRLRDLTL

>C.BR.10.10BR_PE023.KT427736

LYRRLRDLIS

>C.BR.10.10BR_SP033.KT427810

SYHRLRDFIL

>C.BR.10.10BR_SP041.KT427806

SYHRLRDLIL

>C.BR.10.10BR_SP060.KT427800

SYHRLRDLLL

>C.BR.10.DEMC10BR024.KU749392

SYHRLRDLIL

>C.BR.11.DEMC11BR035.KU749393

SYHRLRDLLL

>C.BR.92.BR025_d.U52953

SYHRLRDLIL

>C.BR.98.98BR004.AF286228

SYHRLRDLLS

>C.BW.00.00BW07621.AF443088

SYHRLRDFIL

>C.BW.00.00BW076820.AF443089

SYHRLRDFIL

>C.BW.00.00BW087421.AF443090

SYHRLRDFIL

>C.BW.00.00BW147127.AF443091

LYRLLSDFIS

>C.BW.00.00BW16162.AF443092

SYHRLRDFTL

>C.BW.00.00BW1686.AF443093

SYHQLRDFIL

>C.BW.00.00BW17593.AF443094

SYHRLRDLIL

>C.BW.00.00BW17732.AF443095

CYHRLRDFIL

>C.BW.00.00BW17835.AF443096

SYHRLRDFIL

>C.BW.00.00BW17956.AF443097

CYRRLRDFIL

>C.BW.00.00BW18113.AF443098

CYHRLRDFIL

>C.BW.00.00BW192113.AF443101

SYHRLRDLIL

>C.BW.00.00BW20361.AF443102

SYHQLRDLIL

>C.BW.00.00BW20636.AF443103

CYHRLKDFVL

>C.BW.00.00BW20872.AF443104

SYHRLRDLIL

>C.BW.00.00BW2127214.AF443105

SYHRLRDLIL

>C.BW.00.00BW22767.AF443107

SYHRLRDFIL

>C.BW.00.00BW38193.AF443108

SYHRLRDLTL

>C.BW.00.00BW38428.AF443109

SYHRLRDLIL

>C.BW.00.00BW38713.AF443110

SYHRLRDFIL

>C.BW.00.00BW3876_9.AF443111

SYHRLRDFIL

>C.BW.00.00BW3886_8.AF443112

SYHRLRDFIL

>C.BW.00.00BW3891_6.AF443113

SYHRLRDFIL

>C.BW.00.00BW3970_2.AF443114

SYHHLRDFIL

>C.BW.00.00BW5031_1.AF443115

SYRHLRDFIL

>C.BW.00.DEMC00BW010.KY658704

SYHRLRDCIL

>C.BW.00.DEMC00BW013.KY658705

SYHRLRDFIL

>C.BW.00.DEMC00BW014.KY658706

CYHRLRDLIL

>C.BW.04.1811_B3_23.KC154012

SYHRLRDFIL

>C.BW.05.2865_A11_12.KC154013

SYRRLRDLIL

>C.BW.05.3312_D6_2.KC154014

CYHRLRDFIL

>C.BW.06.3603_C11_13.KC154015

SYHQLRDFIL

>C.BW.06.B005018_8_F6.3.KF114881

CYRQLRDFIL

>C.BW.07.B005582_7_G7.8.KF114882

FYHQLRDFIL

>C.BW.10.mpp_00063_amp2.KR861313

SYHRLRDFIL

>C.BW.10.mpp_00115_amp2.KR861315

SYHQLRDFIL

>C.BW.10.mpp_00117_amp2.KR861316

SYHRLRDFIL

>C.BW.10.mpp_00120_amp2.KR861317

SYHRLRDLLL

>C.BW.10.mpp_00197_amp2.KR861325

SYHQLRDFIL

>C.BW.10.mpp_00205_amp2.KR861326

SYHRLRDFIL

>C.BW.10.mpp_00241_amp2.KR861329

SYHRLRDFIL

>C.BW.10.mpp_00270_amp2.KR861331

SYHRLRDFIL

>C.BW.10.mpp_00307_amp2.KR861333

SYHRLRDFIL

>C.BW.10.mpp_00361_amp2.KR861337

IYHRLRDFIL

>C.BW.10.mpp_00380_amp2.KR861340

SYHRLRDLIL

>C.BW.10.mpp_00489_amp2.KR861345

CYHRLRDFIL

>C.BW.11.mpp_00038_amp2.KR861312

CYRQLRDFTL

>C.BW.11.mpp_00088_amp2.KR861314

SYHRLRDLVL

>C.BW.11.mpp_00145_amp2.KR861320

SYHRLRDFIL

>C.BW.11.mpp_00157_amp2.KR861321

SYHRLRDFIL

>C.BW.11.mpp_00160_amp2.KR861322

LYHRLRDFIL

>C.BW.11.mpp_00168_amp2.KR861323

SFHRLRDLTL

>C.BW.11.mpp_00188_amp2.KR861324

SYHRLRDCIL

>C.BW.11.mpp_00259_amp2.KR861330

CYHRLKDFIL

>C.BW.11.mpp_00286_amp2.KR861332

SYHRLRDFIL

>C.BW.11.mpp_00321_amp2.KR861334

CYRRLRDFAL

>C.BW.11.mpp_00323_amp2.KR861335

CYRQLRDFIL

>C.BW.11.mpp_00354_amp2.KR861336

SYHQLRNFIL

>C.BW.11.mpp_00373_amp2.KR861339

CYHRLRDFIL

>C.BW.11.mpp_00437_amp2.KR861341

SYHRLRDLIL

>C.BW.11.mpp_00454_amp2.KR861342

SYHRLRDLTL

>C.BW.11.mpp_00456_amp2.KR861343

SYHQLRDFIL

>C.BW.12.mpp_00037_amp2.KR861311

SYHRLRDFIL

>C.BW.12.mpp_00144_amp2.KR861319

SYHRLRDFIL

>C.BW.12.mpp_00219_amp2.KR861327

SYRQLRNFIL

>C.BW.12.mpp_00478_amp2.KR861344

SYHRLRDCIL

>C.BW.13.bcpp_00147_amp2.KR861270

SYHRLRDFIL

>C.BW.13.bcpp_00192_amp2.KR861279

CYHRLRDFIL

>C.BW.13.mpp_00238_amp2.KR861328

SYHRLRDLTL

>C.BW.14.bcpp_00030_amp2.KR861257

SYHRLRDCTL

>C.BW.14.bcpp_00048_amp2.KR861259

CYHRLRDFIL

>C.BW.14.bcpp_00090_amp2.KR861264

CYHRLRDFIL

>C.BW.14.bcpp_00127_amp2.KR861269

SYHRLRDFIL

>C.BW.14.bcpp_00155_amp2.KR861271

CYHRLIDFTL

>C.BW.14.bcpp_00166_amp2.KR861273

SYHRLRDCIL

>C.BW.14.bcpp_00180_amp2.KR861276

SYHRLRDFIL

>C.BW.14.bcpp_00201_amp2.KR861280

SYHRLRDFIL

>C.BW.14.bcpp_00204_amp2.KR861281

CYHRLRDFTL

>C.BW.14.bcpp_00207_amp2.KR861282

SYHRLRDFIL

>C.BW.14.bcpp_00211_amp2.KR861283

SYHQLRDFIL

>C.BW.14.bcpp_00395_amp2.KR861302

IYRRLRDFTL

>C.BW.14.bcpp_00403_amp2.KR861304

SYHRLRDFIL

>C.BW.14.bcpp_00460_amp2.KR861308

SYHRLRDFIL

>C.BW.96.96BW01B03.AF110959

SYHRLRDFIL

>C.BW.96.96BW06.AF290028

SYHQLRDFIL

>C.BW.96.96BW15B03.AF110973

SYHRLRDLIL

>C.BW.96.96BW17.AF110981

LYHHLRDFIL

>C.BW.96.96BWM032.AF443075

SYHRLRDFLL

>C.BW.96.96BWMO1_5.AF443074

CYHRLRDFIL

>C.BW.96.DEMC96BW001.KY658707

SYHRLRDFIL

>C.BW.96.DEMC96BW002.KY658708

SYHRLRDFIL

>C.BW.96.DEMC96BW006.KY658709

CYHRLRDFTL

>C.BW.96.DEMC96BW007.KY658710

SYHRLRDFIL

>C.BW.96.DEMC96BW009.KY658711

SYHRLRDFIL

>C.BW.98.98BWMC122.AF443076

SYHRLRDFIL

>C.BW.98.98BWMC134.AF443077

CYHRLRDFIL

>C.BW.98.98BWMC14A3.AF443078

SYHQLRNFIL

>C.BW.98.98BWMO1410.AF443079

SYHRLRDLIL

>C.BW.98.98BWMO18D5.AF443080

SYHRLRDFIL

>C.BW.98.98BWMO36A5.AF443081

SYHRLRDFIL

>C.BW.98.98BWMO37D5.AF443082

SYHQLRDFIL

>C.BW.99.99BW393212.AF443083

SYHRLRDLIL

>C.BW.99.99BW46424.AF443084

SCHRLRDCIL

>C.BW.99.99BW4745.AF443085

SYHHLRDFIL

>C.BW.99.99BW47547.AF443086

SYHRLRDFIL

>C.BW.99.99BWMC168.AF443087

SYHRLRDLTL

>C.BW.x.mpp_00140_amp2.KR861318

SYHRLRDFIL

>C.CN.06.06YNLC028sg.KC898980

SYHRLSDFVL

>C.CN.06.06YNLC32sg.KC899004

SYHRLRDLIL

>C.CN.06.CNE58.HM215421

SYHRLRDFIL

>C.CN.06.CNE86_U.HQ699991

SYHQLRNFIL

>C.CN.06.CNE88_U.HQ699992

SYHRLRDFIL

>C.CN.07.07CNYN328.KF835515

LYHRLRDFIS

>C.CN.07.07CNYN336.KF835522

LYHRLSDFIL

>C.CN.07.CNE103_U.HQ699999

SYHRLRDFIL

>C.CN.07.CNE106.HQ699985

SYHRLRDFIL

>C.CN.07.CNE17.HM215403

SYHRLTDFIL

>C.CN.07.CNE23.HM215408

SYHRLRDCIL

>C.CN.07.CNE2.HQ699950

SYHRLRDFIS

>C.CN.07.CNE30.HM215411

SHHRLRDFIL

>C.CN.07.CNE66.HQ699981

SYHRLRDFIS

>C.CN.09.09YNLX090sg.KC898995

SYHRLSDLIL

>C.CN.09.09YNLX133sg.KC898996

SYHRLRNFIL

>C.CN.09.DH21.KF250403

SYHRLRDFIS

>C.CN.09.DH22.KF250404

SYHRLSDFIS

>C.CN.10.CNGX752_R11.KC807921

SYHHLRDLLL

>C.CN.10.CNGX757_R5.KC807928

SYHHLRDLIL

>C.CN.10.YNFL19.KC870038

SYHLLRDFIL

>C.CY.05.CY040.FJ388901

IYHRLRDFIL

>C.CY.05.CY069.FJ388913

SYHRLRDFIL

>C.CY.06.CY166.FJ388948

SYHQLRDFIL

>C.CY.06.CY176.FJ388952

SYHRLRDLIL

>C.CY.07.CY187.JF683740

SYHRLRDFIL

>C.CY.07.CY203.JF683755

SYHQLRDFIL

>C.CY.07.CY205.JF683757

CYHLLRDFIL

>C.CY.08.CY219.JF683768

SYHQLRDFIL

>C.CY.08.CY221.JF683770

SYHQLRDFIL

>C.CY.09.CY260.JF683803

FCHRLRSYFL

>C.DE.10.622166.KT124786

SYHRLRDFIL

>C.DJ.91.DJ259A.L22940

SYRRLRDLIL

>C.DJ.91.DJ373A.L23065

SYHRLRDLIL

>C.DK.01.CTL_015.EF514713

SYHRLRDFIL

>C.ES.06.DEMC06ES003.KC473844

SYHRLRDFIL

>C.ES.07.Read4_HIV_C.KX228820

SYHRLRDFIL

>C.ES.08.P2085_G3.HQ236594

SYHRLRDLIL

>C.ES.08.X2363_2.EU786681

SYHRLRDFIL

>C.ES.09.P2321_H4.HQ236601

SYHRLRDLLL

>C.ES.14.ARP1198.KT276258

SYHLLRDFIL

>C.ES.15.100_118.KY496624

SYHRLRDLLS

>C.ES.x.MH08.EF531335

SYHRLRDCIS

>C.ET.02.02ET_288.AY713417

SYHRLRDLIL

>C.ET.08.ET104.KU319528

SYHHLR-ILL

>C.ET.08.ET106.KU319529

SYRRLRDLLS

>C.ET.08.ET108.KU319530

SYHRLRDLIL

>C.ET.08.ET115.KU319531

SYRHLRDLIL

>C.ET.08.ET119.KU319532

SYRHLRDVLL

>C.ET.08.ET122.KU319533

SYHRLRDFTL

>C.ET.08.ET124.KU319534

IYRQLRDLIL

>C.ET.08.ET128.KU319536

SYHRLRDLIL

>C.ET.08.ET130.KU319537

SYHRLRDLLL

>C.ET.08.ET135.KU319538

SYHRLRDLLL

>C.ET.08.ET136.KU319539

IYHRLRDVIL

>C.ET.08.ET145.KU319540

SYHRLRDLLL

>C.ET.08.ET147.KU319541

SYHRLRDLLL

>C.ET.08.ET148.KU319542

SYHQLRDFIL

>C.ET.08.ET149.KU319543

CYHRLRDLIL

>C.ET.08.ET150.KU319544

SYHRLRDFIL

>C.ET.08.ET154.KU319545

LYHHLRDLLL

>C.ET.08.ET155.KU319546

SYHLLRDSIL

>C.ET.08.ET159.KU319547

CYRRLRDLTL

>C.ET.08.ET164.KU319548

SYHRLRDFVL

>C.ET.08.ET165.KU319549

CYHRLRDLIL

>C.ET.08.ET167.KU319550

SYHRLRDFIL

>C.ET.08.ET171.KU319551

SYRHLRDLLL

>C.ET.86.ETH2220.U46016

SYHRLRDLIL

>C.FI.91.FIN9126.AF219262

SYRRLRDLIL

>C.FI.91.FIN9149.AF219263

LYHRLRDFIL

>C.FI.91.FIN9158.AF219264

SYHRLRDFIL

>C.FI.92.FIN9210.AF219266

SYHRLRDFIL

>C.FI.92.FIN9277.AF219269

SYHRLRDLLL

>C.FI.93.FIN9307.AF219270

SYHQLRDFIL

>C.FI.94.FIN9401.AF219274

SYHRLRDFIL

>C.GB.01.27_C27_42.FJ977094

LYHRLRDSIL

>C.GB.01.28_C28_g.FJ977093

SYHRLRDFIL

>C.GB.01.38_C38_22.FJ977095

SYHRLRDLLS

>C.GB.05.N3_DAY_1.FJ653288

SYHRLRDFIL

>C.GB.08.M320d_F9.HQ595787

SYHRLRDFIL

>C.GB.08.M520c_B9.HQ595789

SYHRLRDFIL

>C.GB.08.S315_D10.HQ595800

SYHQLRTFIL

>C.GB.09.N341_p1.JF680927

SYHRLRDFIL

>C.GB.13.13592_1_23.3.MF109366

SYHRLRDFTL

>C.GB.13.13592_1_24.3.MF109367

SYHQLRDFIL

>C.GB.13.13592_1_5.3.MF109389

SYHRLRDLLL

>C.GB.13.13774_1_82.3.MF109504

SYHRLRDFIL

>C.GB.13.14667_1_32.4.MF109593

SYHRLRDLLS

>C.GB.13.15171_1_38.3.MF109670

SYHRLRDCIL

>C.GB.13.15171_1_43.3.MF109676

SYRHLRDFIL

>C.GB.13.15228_1_46.3.MF109684

SYHRLRDFAL

>C.GB.13.15228_1_57.3.MF109695

SYHRLRDFIL

>C.GB.13.15228_1_68.4.MF109706

SYHRLRDFIL

>C.GB.13.15228_1_69.3.MF109707

IYHRLRDFIL

>C.GB.14.13612_1_2.3.MF109413

SYRHLRDLLL

>C.GB.14.13612_1_29.3.MF109412

SYHQLRDFIL

>C.GB.14.13659_1_46.3.MF109435

CYHRLRDFIL

>C.GB.14.13659_1_48.3.MF109437

SYHRLRDFIL

>C.GB.14.13659_1_53.3.MF109441

SYHRLRDLIL

>C.GB.14.14535_1_12.4.MF109513

SYHRLRDCIS

>C.GB.14.14535_1_25.3.MF109522

IYHRLRDLLL

>C.GB.14.14535_1_8.3.MF109535

SYHQLRDFIL

>C.GB.14.14592_1_43.5.MF109539

SYHQLRDFIL

>C.GB.14.14592_1_63.3.MF109557

SYHHLRDFIL

>C.GB.14.14592_1_69.3.MF109563

SYHQLRDFIL

>C.GB.14.14667_1_30.3.MF109591

SYRQLRDFIL

>C.GB.14.14667_1_31.3.MF109592

SYHRLRDFLL

>C.GB.14.14667_1_39.4.MF109599

SYHRLRDFIL

>C.GB.14.14727_1_41.4.MF109606

SYHHLRDFIL

>C.GB.14.14727_1_42.3.MF109607

SYHQLRDFIL

>C.GB.14.14727_1_49.3.MF109612

SYRRLRDLVL

>C.GB.14.14727_1_54.7.MF109617

SYHRLRDFIL

>C.GE.03.03GEMZ033.DQ207941

SYHRLRDFIL

>C.GM.99.N005312.HQ385458

SYHRLRDFIL

>C.IL.98.98IS002.AF286233

SYHRLRDFIL

>C.IL.99.99ET14.AY255825

SYHRLRDLIL

>C.IL.99.99ET17.AY255826

SYHRLRDFIL

>C.IL.99.99ET1.AY255823

SYRRLTDLLS

>C.IL.99.99ET7.AY255824

SYHHLRDFIL

>C.IN.00.DEMC00IN005.KP109480

SYHRLRDFIL

>C.IN.00.DEMC00IN006.KP109481

CYHRLRDFIL

>C.IN.00.DEMC00IN007.KP109482

SYHRLRDFIL

>C.IN.00.DEMC00IN008.KP109483

SYHRLRDFIS

>C.IN.00.DEMC00IN009.KP109484

SYHRLRDFIL

>C.IN.00.DEMC00IN010.KP109485

SYHRLRDLIS

>C.IN.00.NARI_VB105_J10.EU521729

SYHLLRDFIS

>C.IN.03.D24.EF469243

LYHRLTDLIS

>C.IN.04.C.IN.04.NIRT379.1.KF766537

SYHRLRDFVL

>C.IN.05.C.IN.05.NIRT333.1.KF766540

SYHRLRDFIL

>C.IN.05.C.IN.05.NIRT723.1.KF766541

SYHRLRDFIL

>C.IN.07.NARI_IVC4_NEM_J2.EU908218

SYHRLRDLIL

>C.IN.07.NARI_VB52_J19.EU521727

SYHRLRDFIL

>C.IN.09.T125_2139.KC156210

SYHRLRDFIL

>C.IN.11.NIRT009.KX069227

LYHRLRDFIL

>C.IN.11.NIRT010.KX069228

SYHRLRDFIL

>C.IN.13.NIRT_ENV003.KX756602

LYHRLRDFIL

>C.IN.13.NIRT_ENV011.KX756610

SYHRLRDFIL

>C.IN.13.NIRT_ENV012.KX756611

LYHHLRDFIL

>C.IN.13.NIRT_ENV013.KX756612

SYHRLRDFIS

>C.IN.13.NIRT_ENV014.KX756613

SYHRLXDFIS

>C.IN.13.NIRT_ENV015.KX756614

SYHRLRDCIL

>C.IN.13.NIRT_ENV017.KX756615

SYHRLRDFIS

>C.IN.14.NIRT004.KX069222

SYHRLRDFIL

>C.IN.14.NIRT_ENV001.KX756600

CYHRLRDFIL

>C.IN.14.NIRT_ENV002.KX756601

SYHRLRDLTL

>C.IN.14.NIRT_ENV004.KX756603

SYHRLRDFIS

>C.IN.14.NIRT_ENV005.KX756604

SYHRLRDFIS

>C.IN.14.NIRT_ENV006.KX756605

SYHRLRDFIX

>C.IN.14.NIRT_ENV007.KX756606

SYHRLRDFIL

>C.IN.14.NIRT_ENV008.KX756607

SYHRLRDCIL

>C.IN.14.NIRT_ENV010.KX756609

CYHRLRDFIL

>C.IN.15.MK076687.MK076687

SYHRLRDFIL

>C.IN.15.NIRT001.KX069219

SYHRLRDFIL

>C.IN.15.NIRT002.KX069220

CYHRLRDFIL

>C.IN.15.NIRT003.KX069221

SYHRLRDFIL

>C.IN.15.NIRT005.KX069223

LYHRLRDFIL

>C.IN.15.NIRT006.KX069224

SYHRLRDLLL

>C.IN.15.NIRT007.KX069225

SYHRLRDLLL

>C.IN.15.NIRT008.KX069226

SYHRLRDFIS

>C.IN.15.SC007.KY713228

SYHRLRDLIL

>C.IN.15.SC008.KY713229

SYHRLRDFIS

>C.IN.15.SC013.KY713230

SYHRLRDFIL

>C.IN.15.SC015.KY713231

SYHRLRDLIS

>C.IN.15.SC022.KY713233

CYHRLRDFIL

>C.IN.15.SC023.KY713248

CYHRLRDFIL

>C.IN.15.SC085.KY713235

SYHRLRDFIL

>C.IN.15.SHE001.KY713236

SYHRLRDFIL

>C.IN.16.MK076716.MK076716

SYHRLRDFIL

>C.IN.93.93IN999.AF067154

SYHRLRDFIL

>C.IN.94.20635_4.JQ715368

SYHRLKDCIL

>C.IN.94.94IN11246.AF067159

SYHRLRDFIL

>C.IN.94.94IN476.AF286223

SCHRLRDFIL

>C.IN.95.95IN21068.AF067155

SYHRLRDFIL

>C.IN.98.98IN012.AF286231

SYHRLRDLIL

>C.IN.98.98IN022.AF286232

LYRRLRDFIS

>C.IN.99.01IN565_10.AY049708

SYHRLRDFIL

>C.IN.99.DEMC99IN001.KP109486

SYHRLRDFTL

>C.IN.99.DEMC99IN002.KP109487

SYHRLRDFIL

>C.IN.99.DEMC99IN003.KP109488

SYHRLRDLIL

>C.IN.99.DEMC99IN004.KP109489

CYHQLRDFIS

>C.IN.x.CALCMANDAL.AJ276221

SYHRLRDLIL

>C.IN.x.NARI_VB96_J21.EU521728

LYHRLRDFIL

>C.IN.x.VB39.EF694033

LYHQLRDFIL

>C.IN.x.VB49.EF694036

SYHRLRDFIS

>C.KE.00.KER2010.AF457054

SYHRLRDCIL

>C.KE.02.QD022_1957I_ENV_A4.FJ396028

SYRRLRDLIL

>C.KE.04.04KE344827V3.KT022362

SYHRLRDLLL

>C.KE.04.04KE585573V2.KT022366

SYHRLRDFIL

>C.KE.05.05KE369195V4.KT022371

SYHRLKDLAL

>C.KE.11.093_KE.KU921723

SYHRLRDFLL

>C.KE.11.282_KE.KU921743

SYHRLRDLLS

>C.KE.11.473_KE.KU921790

SYHRLRDFIL

>C.KE.11.525_KE.KU921807

SYHRLRDFIL

>C.KE.11.650_KE.KU921827

LYHRLTDLLS

>C.KE.91.KNH1268.AY945738

LYHRLRDLIL

>C.KE.95.QB099_391M_ENV_C8.FJ866132

SYHRLRDFIL

>C.KE.97.QC406_70M_ENV_F3.FJ866133

SYHQLRDLLL

>C.MM.99.mIDU101_3.AB097871

CYHRLTDFIS

>C.MW.02.329MW_BF2002.JN983803

SYHQLRDFIL

>C.MW.02.591MW_BF2002.JN983804

SYHRLRDFIL

>C.MW.02.703MW_BF2002.JN983805

SYHRLRDLIL

>C.MW.02.BF1266_431a.HM215360

CYHRLRDFTL

>C.MW.03.0393_C3.KC894074

SYHRLRDFIL

>C.MW.03.0626_E6.KC894075

SYHRLRDFVL

>C.MW.03.0665_F2.KC894076

SYHRLRDFIL

>C.MW.03.0682_E4.KC894077

SYHQLRDFIL

>C.MW.03.089_G2.KC894073

SYHRLRDLIL

>C.MW.03.CHV0011179_0114_H10.FJ444184

SYHQLRNFIL

>C.MW.03.CHV0011202_0334_H1.FJ444207

SYHQLRDFIL

>C.MW.03.CHV0011247_0478_H2.FJ444251

SYHQLRDFLL

>C.MW.03.CHV0011273_0595_G9FRAMESHIFT.FJ444277

SYHRLRDFIL

>C.MW.04.0985_H7.KC894078

SYHRLRDFIL

>C.MW.04.1086_B2.KC894079

SYHLLRDFIL

>C.MW.04.1172_H1.KC894080

SYHQLRDFIL

>C.MW.04.1176_A3.KC894081

SYHQLRDFIL

>C.MW.04.1394_C9.KC894082

SYHRLRDFIL

>C.MW.04.CHV0011472_1196_D7.FJ444476

SYHRLRDFIL

>C.MW.04.CHV0011496_1335_H3.FJ444500

SYHRLRDCIL

>C.MW.04.CHV0011518_1373_G4.FJ444522

SYHRLRDFIL

>C.MW.05.2010_F5.KC894083

CYHQLRDFTL

>C.MW.05.2060_G9.KC894085

CYHRLRDFIL

>C.MW.05.2103_E8.KC894086

SYHQLRDFVL

>C.MW.05.3003_2_D3.KC894087

SYHRLRDFIL

>C.MW.05.3004_2_B8.KC894088

SYHRLRDFIL

>C.MW.05.3011_2_F4.KC894089

SYHRLRDLIL

>C.MW.05.3012_2_C9.KC894090

SYHRLRDFIL

>C.MW.05.3022_2_A2.KC894091

SYHRLRDFIL

>C.MW.05.3025_2_C3.KC894092

SYHRLRDFIL

>C.MW.05.3026_2_H9.KC894093

SYHRLRDFIL

>C.MW.05.3027_2_F11.KC894094

SYRQLRDFIL

>C.MW.05.3029_2_E2.KC894095

SYHRLRDFIL

>C.MW.05.3034_2_D8.KC862818

SYHRLRDFIL

>C.MW.05.3036_2_C12.KC894097

SYHRLRDFIL

>C.MW.05.3037_6.KC894098

SYHLLRDFIL

>C.MW.05.3039_10.KC862876

SYHQLRDFLL

>C.MW.05.3040_2_C8.KC894100

SYHRLRDFIL

>C.MW.05.3041_21.KC862923

SYHRLRDFIL

>C.MW.05.3042_2_C9.KC894101

SYHRLRDFLL

>C.MW.05.3044_1.KC862949

SYHRLRDFIL

>C.MW.05.3048_2_E3.KC894103

SYHRLRDFIL

>C.MW.05.3049_1.KC863009

SYHHLRDFIS

>C.MW.05.3050_2_C3.KC894385

SYHQLRDFIL

>C.MW.06.2052_G10.KC894084

SYHRLRDFIL

>C.MW.07.702010141_CH141.w12.KC156212

SYHRLRDLLL

>C.MW.07.703010054_2A2.KC894106

CYHRLRDFIL

>C.MW.07.703010085_CH085.w4a.KC156211

LYHHLRDFLL

>C.MW.07.703010131_CH131_TF.KC156114

SYHRLRDCVL

>C.MW.07.703010167_CH167.w8.KC156213

SYHQLRDFIL

>C.MW.07.703010200_CH200_TFc.KC156119

SYHRLRDFIL

>C.MW.07.703010217_B6.KC894109

SYRQLRDFIL

>C.MW.07.703010228_CH228_TFa.KC156120

SYHRLRDLLL

>C.MW.07.CH0010.w24.e11.KC148599

SYHRLRDLIL

>C.MW.07.CH0047.w2.e11.KC148663

SYHQLRDFIW

>C.MW.07.CH010073_w16_p1.HM204583

SYRLLRDFIL

>C.MW.07.CH010102_e_p2.HM204588

SYHRLRDFIL

>C.MW.07.CH010141_w12_p1.HM204591

SYHRLRDLLL

>C.MW.07.CHV0008489_703010193_D2_stop.FJ443615

CYHLLRDFLL

>C.MW.07.DEMC07MW001.KP109520

SYHQLRDFIL

>C.MW.08.0301_G12.HM070477

SYHQLRDFIL

>C.MW.08.0702bmL_H19.HM070526

SYHRLRDLIS

>C.MW.08.1209_B7.HM070539

SYHRLRDFIL

>C.MW.08.3009_B4.HQ595984

IYHRLRDFIL

>C.MW.08.3305_D5.HM070584

SYHRLRDCIL

>C.MW.08.3404_A1.HQ596030

SYHRLRDFIL

>C.MW.08.3902_E2.HM070639

SYHRLRDCIL

>C.MW.08.4403bmLwk4_fl11.KF527172

LYHRLRDFLL

>C.MW.08.4707_G3.HM070807

CYHQLRDFIL

>C.MW.08.5807_C2.HQ596147

SYHRLRDFIL

>C.MW.08.702010118_C10.HQ615984

SYHRLRDFIL

>C.MW.08.702010293_CH293.w8a.KC156216

CYHQLRDFIL

>C.MW.08.702010432_CH432.w4.KC156218

IYHRLRDFIL

>C.MW.08.702010440_CH440.w4.KC156219

SYHRLRDFIL

>C.MW.08.703010269_CH269.w24.KC156215

SYHLLRDFLL

>C.MW.08.703010479_D7.HQ615962

SYHQLRDLLL

>C.MW.08.703010588_E7.HQ615963

CYHRLRDFIL

>C.MW.08.703010632_A1.HQ615964

SYHQLRDFIL

>C.MW.08.703010760_H9.HQ615982

SYHRLRDFLL

>C.MW.08.CH010259_w16_p1.HM204597

IYHQLRDFIL

>C.MW.08.CH010432_w4_p1.HM204613

SYHQLRDFIL

>C.MW.08.CH0275.w24.e1.KC149493

SYHRLRDFIL

>C.MW.08.CH427.PL.011608.UT.5.KY112213

SYHRLRDFIS

>C.MW.08.CH455.PL.012908.UT.1.KY112254

SYHRLRDLIS

>C.MW.08.CH596.PL.030408.UT.6.KY112386

SYHRLRDLLL

>C.MW.08.DEMC08MW002.KP109521

SYHQLRDFIL

>C.MW.08.DEMC08MW004.KP109522

SYHHLRDFIL

>C.MW.09.0404_C6.HQ595814

SYHRLRDLIL

>C.MW.09.052_2899_I_A7.KC634164

SYHRLRDLIL

>C.MW.09.3002_CSF_Visit_1_amplicon11.KC186976

SYHRLRDFIL

>C.MW.09.3006_CSF_Visit_1_amplicon4.KC187352

SYHQLRDFIL

>C.MW.09.3009_Plasma_Visit_1_amplicon6.KC186690

SYHRLRDFIL

>C.MW.09.3017_CSF_Visit_1_amplicon11.KC187578

SYHQLRDFIL

>C.MW.09.4001_CSF_Visit_1_amplicon30.KC187323

SYHRLRDFIL

>C.MW.09.4002_CSF_Visit_1_amplicon9.KC186955

SYHRLRDFIL

>C.MW.09.4004_Plasma_Visit1_amplicon1.KC187164

SYHRLRDFIL

>C.MW.09.4007_Plasma_Visit1_amplicon16.KC187128

SYHRLRDFIL

>C.MW.09.4013_Plasma_Visit1_amplicon28.KC186723

SYHRLRDCIL

>C.MW.09.4014_CSF_Visit1_amplicon15.KC186899

SYRHLRDFIL

>C.MW.09.4015_CSF_Visit1_amplicon14.KC186242

SYHRLRDLAL

>C.MW.09.4016_Plasma_Visit1_amplicon23.KC187207

SYHQLRNFLL

>C.MW.09.4017_Plasma_Visit1_amplicon8.KC186306

SYRQLRDFIL

>C.MW.09.4026_CSF_Visit1_amplicon4.KC187437

SYRRLRDFTL

>C.MW.09.4027_Plasma_Visit_1_amplicon5.KC186647

SYHRLRDLTL

>C.MW.09.4029_CSF_Visit1_amplicon1.KC186730

SYHRLRDFIL

>C.MW.09.4030_CSF_Visit1_amplicon28.KC187550

SYHRLRDLIL

>C.MW.09.4031_CSF_Visit1_amplicon14.KC187665

CYHRLRDFVL

>C.MW.09.4032_CSF_Visit1_amplicon11.KC187376

SYHQLRDFIL

>C.MW.09.4034_Plasma_Visit1_amplicon20.KC186377

SYHRLRDCIL

>C.MW.09.4036_Plasma_Visit1_amplicon22.KC187723

SYHRLRDFLL

>C.MW.09.4037_CSF_Visit1_amplicon10.KC186458

CYHQLRDFIL

>C.MW.09.4039_Plasma_Visit1_amplicon12.KC187620

SYHRLRDFLL

>C.MW.09.4041_CSF_Visit_1_amplicon1.KC186388

SYHRLRDFLL

>C.MW.09.4045_Plasma_Visit1_amplicon14.KC187049

SYHRLRDLIL

>C.MW.09.4046_CSF_Visit1_amplicon2.KC187073

SYHRLRDFIL

>C.MW.09.4048_CSF_Visit1_amplicon4a.KC186320

SYHRLRDFVL

>C.MW.09.4049_Plasma_Visit1_amplicon16.KC187482

LYHRLTDLIL

>C.MW.09.4050_CSF_Visit1_amplicon7a.KC186143

CYHQLRDFIL

>C.MW.09.703010256_CH256.w96.KC156214

SYHRLRDFIL

>C.MW.09.CH1064.PL.080409.A2.08.KY111994

SYHRLRDFLL

>C.MW.09.DEMC09MW006.KP109523

SYHRLRDFIL

>C.MW.09.DEMC09MW007.KP109524

SYRHLRDFIL

>C.MW.09.DEMC09MW008.KP109525

SYHRLRDFIL

>C.MW.09.DEMC09MW009.KP109526

SYRQLRDFIL

>C.MW.09.DEMC09MW010.KP109527

SYHHLRDFIL

>C.MW.10.052_1168_I_A2.KC634109

SYHRLRDFIL

>C.MW.10.3032_CSF_Visit_1_amplicon1.KC187500

SYHLLRDFIL

>C.MW.10.3036_CSF_Visit_1_amplicon19.KC187630

SYHRLRDFIL

>C.MW.10.3037_CSF_Visit_1_amplicon10.KC186203

SYHRLRDCIL

>C.MW.10.3040_CSF_Visit_1_amplicon1.KC187280

SYHRLRDLLL

>C.MW.10.4055_CSF_Visit1_amplicon17.KC187249

LYRRLRDFIL

>C.MW.10.4056_Plasma_Visit1_amplicon12.KC186594

SYHRLRDFIL

>C.MW.10.4058_CSF_Visit1_amplicon15.KC186169

CYRRLGDFIL

>C.MW.10.4059_CSF_Visit1_amplicon15.KC186502

SYHRLRDFIL

>C.MW.10.4061_Plasma_Visit1_amplicon21.KC186446

SYHQLRDFIL

>C.MW.11.052_2173_P_A2.KT896551

SCHRLRDFIL

>C.MW.11.703011244.3.d0847.ipe015.3.13.MF500628

SYHRLRDFIL

>C.MW.11.CH0694_3_d1009_ipe018_15_49.MG898649

SYHRLRDLLL

>C.MW.12.CH1754.3.d0832.ipe021_15.20.MF502153

SYHRLRDFIL

>C.MW.13.CH1012_3_d1655_ipe017_180_10.MG899863

SYHRLRDLLL

>C.MW.14.703010505_w323_3.MF353063

LYHRLRDFIS

>C.MW.93.93MW_965.AY713413

SYHQLRDFIL

>C.MW.96.C007_1E12_2B.HM638662

SYHRLRDLIL

>C.MW.96.C009_B8_3B.HM638713

LYHQLRDFIL

>C.MW.96.C011_B9_3B.HM638770

SYHRLRDFIL

>C.MW.96.C012_F12_3S.HM638803

SYHRLRDFIL

>C.MW.96.C018_E8_3S.HM638848

SYHQLRDFIL

>C.MW.96.C019_E1_3S.HM638911

SYHQLRDFIL

>C.MW.96.C030_F8.KC894126

SYHRLRDFIL

>C.MW.96.C034_B2.KC894127

SYHQLRDFIL

>C.MW.96.C047_E10_2S.HM638986

SYHRLRDFIL

>C.MW.96.C047_H7_3B.KC894128

SYHRLRDFIL

>C.MW.96.C059_2B3.KC863127

SYHQLRDFIL

>C.MW.96.C061_D6.KC894129

SYHRLRDFIL

>C.MW.96.C070_D9_2S.HM639015

SYHRLRDFIL

>C.MW.96.C083C8_3B.HM639114

SYHRLRDFIL

>C.MW.96.C083_B7_3B.KC894130

SYHRLRDFIL

>C.MW.96.C111_G3_3S.HM639196

SYHRLRDLLL

>C.MW.96.C111_H2.KC894132

SYHRLRDLLL

>C.MW.96.C113_E6_2S.HM639260

LYHRLTDFLL

>C.MW.96.C120_3D3.KC863183

SYHRLRDFIL

>C.MW.x.BF1677F2_613a.HM215361

SYHRLRDFLL

>C.MW.x.BF942_218d.HM215362

SYHQLRDFIL

>C.NG.10.10NG020523.KX389612

SYHRLRDFIL

>C.NP.00.11NP115.KU341731

SYHRLRDFIS

>C.NP.07.11NP091.KU341730

CYHRLRDLIS

>C.NP.08.11NP004.KU341722

SYHRLRDFIS

>C.NP.08.11NP084.KU341728

SYHRLRDLIS

>C.NP.09.11NP076.KU341726

SYHRLRDFIS

>C.NP.10.11NP065.KU341725

SYHRLRDFIS

>C.NP.11.11NP003.KJ158421

SYHRLRDFIL

>C.NP.11.11NP007.KJ158423

CYHRLTDLIS

>C.NP.11.11NP008.KJ158424

SYHRLRDFIL

>C.NP.11.11NP010.KJ158425

SYHRLRDFIL

>C.NP.11.11NP014.KU341723

SYRRLRDFIS

>C.NP.11.11NP015.KJ541837

SYHRLRNFVL

>C.NP.11.11NP016.KU341724

SYHRLRDLIS

>C.NP.11.11NP028.KJ158428

SYHRLRDLIL

>C.NP.11.11NP041.KJ158430

SYRHLRDLLS

>C.NP.11.11NP068.KJ158433

SYHRLRDFIL

>C.NP.11.11NP069.KJ158434

SYHRLRDLIL

>C.NP.11.11NP071.KJ541839

SYHRLRDFIS

>C.NP.11.11NP074.KJ541840

TYPRLRDFIS

>C.NP.11.11NP075.KJ541841

SYHRLRDFIL

>C.NP.11.11NP080.KJ158435

SYHRLRDFIL

>C.NP.11.11NP082.KJ541844

SYHRLRDFIS

>C.NP.11.11NP092.KJ158438

SYHRLRDLIL

>C.NP.11.11NP093.KJ158439

SYHRLRDLIL

>C.NP.11.11NP095.KJ541846

LYHQLRNSIL

>C.NP.11.11NP102.KJ158440

CYHRLRDFIL

>C.NP.11.11NP104.KJ541848

SYHRLRDFIL

>C.NP.11.11NP107.KJ541849

SYHRLRDFIL

>C.PK.14.DEMC14PK009.KU749412

LYHRLRDLLL

>C.SE.03.004ZM.MF373126

SNHRLRDFIL

>C.SE.04.006CD.MF373128

SYHRLRDFIL

>C.SE.05.010BI.MF373131

SYRRLRDLLL

>C.SE.05.011BI.MF373132

SYHRLRDLLL

>C.SE.06.013ET.MF373134

SYHRLRDLIL

>C.SE.06.014ET.MF373135

SYHRLRDFIL

>C.SE.06.016SE.MF373136

SYHRLRDFIL

>C.SE.06.017BI.MF373137

SYRRLRDLIL

>C.SE.06.018SE.MF373138

CYHRLRDLLL

>C.SE.06.019KE.MF373139

LYHRLRDLLL

>C.SE.06.SE600516.KP411839

CYHQLRTFIL

>C.SE.07.SE600108.KP411830

SYHRLRDLLL

>C.SE.07.SE600119.KP411831

SYHRLRDFIL

>C.SE.07.SE600122.KP411832

SYHHLRDFIL

>C.SE.08.SE600210.KP411833

CYRRLRDLVL

>C.SE.08.SE600213.KP411834

SSHRWRDLFW

>C.SE.09.031ER.MF373145

SYRQLRDLIL

>C.SE.09.033ET.MF373146

SYRRLKDLLL

>C.SE.09.035ZA.MF373148

SYHRLRDLVL

>C.SE.09.SE600314.KP411836

SYHQLRDFIL

>C.SE.10.041SE.MF373152

SYRRLRDLIL

>C.SE.10.043SE.MF373154

SYRRLRDLLS

>C.SE.10.SE600412.KP411837

SYRRLRDLLL

>C.SE.11.053SE.MF373156

SYRQLRDFLL

>C.SE.11.054ET.MF373157

SYQRLRDCIL

>C.SE.11.055ER.MF373158

SYHRLRDFIL

>C.SE.11.056IT.MF373159

CYRRLRDLLL

>C.SE.11.061SO.MF373164

SYHRLRDLIL

>C.SE.11.062ZA.MF373165

SYHRLRDFIL

>C.SE.11.063BI.MF373166

SYHHLRDLLL

>C.SE.12.066SE.MF373169

SYHRLRDLIS

>C.SE.12.067SE.MF373170

SYHRLRDLLL

>C.SE.12.068SE.MF373171

SYHRLRDLLL

>C.SE.12.072SO.MF373175

SYRRLRDLLL

>C.SE.12.073NP.MF373176

SYHRLRDFIL

>C.SE.12.074SE.MF373177

SYRRLRDLIL

>C.SE.12.075ZA.MF373178

LYHQLRDFTL

>C.SE.12.076SO.MF373179

SLLHLRDLIE

>C.SE.13.085BI.MF373187

SYHRLRDLLL

>C.SE.13.090ET.MF373192

SYHRLRDLLL

>C.SE.13.SE600311.KP411835

SYRGWRDLFL

>C.SE.14.091SE.MF373193

CYHQLRDFTL

>C.SE.14.095CD.MF373197

SYHRLRDCIL

>C.SE.14.096SE.MF373198

SYRQLRDLLL

>C.SE.14.097SO.MF373199

SYRHLRDFIL

>C.SE.15.100ET.MF373202

SYHRLRDLLL

>C.SN.90.90SE_364.AY713416

SYRRLRDFIL

>C.SN.96.5056135.MH705137

SYHQLRDFIL

>C.SO.89.89SM_145.AY713415

SYHRLRDFIL

>C.TH.x.PWJ_0513_39.HM215433

CYHRLRDFIL

>C.TZ.00.142_F3_B1.HQ659585

SYRQLRDFIL

>C.TZ.00.234_F1_1.HQ697934

SYHRLRDFIL

>C.TZ.00.390_F1_B7.HQ697983

SYHRLRDFIL

>C.TZ.00.410_F2_7.HQ698009

SYHQLRDFIL

>C.TZ.01.304_F2_1_11.HM215286

SYHRLRDFIL

>C.TZ.01.346_F4_D2_12.HM215302

SYRRLRDFIL

>C.TZ.01.556_F2_3_25.HM215317

SYHRLRDFIL

>C.TZ.01.569_F1_37_10.HM215318

CYHRLRDFIL

>C.TZ.01.98_F4_H5_13.HM215354

SYHRLRDFIL

>C.TZ.01.A125.AY253304

SYHRLRDFIL

>C.TZ.01.A207.AY253307

SYHRLRDCIL

>C.TZ.01.A246.AY253308

CYHRLTDFIS

>C.TZ.01.A260.AY253310

CYHRLRDFIL

>C.TZ.01.A301.AY253312

SYHQLRDFIL

>C.TZ.01.A388.AY253317

SYHQLRDFIL

>C.TZ.01.A93.AY253303

SYHRLRDFIL

>C.TZ.01.BD16_10.AY253320

SYHRLRDFLL

>C.TZ.01.BD22_11.AY253321

SYHRLRDFIL

>C.TZ.01.BD39_8.AY253313

CYHLLRDFIL

>C.TZ.01.BD9_11.AY253322

CYHQLRDFIL

>C.TZ.02.CO178.AY734556

SYHRLRDFIL

>C.TZ.02.CO3056.AY734550

SYHRLRDFIL

>C.TZ.02.CO328.AY734551

SYHRLRDFIL

>C.TZ.02.CO3305.AY734558

SYHLLRDFIL

>C.TZ.02.CO6130.AY734559

LYHRLRDFIW

>C.TZ.02.CO6721.AY734560

SYHLLRDFIL

>C.TZ.03.CO0077V1.KX907339

SYHRLRDFIL

>C.TZ.03.CO0921V2.KX907356

SYHRLRDFIL

>C.TZ.03.CO0984V2.KX907358

SYHRLRDFIL

>C.TZ.03.CO3873V1.KX907388

SYHQLRDFIL

>C.TZ.03.CO6471V1.KX907407

CYHRLRDLIL

>C.TZ.03.CO6838V1.KX907424

SYHQLRDFIL

>C.TZ.03.CO6854V1.KX907426

SYHLLGDFIL

>C.TZ.03.CO6980V0.KX907433

LYHHLRDFIL

>C.TZ.04.CO0041V3.KX907337

SYHRLXDFIL

>C.TZ.04.CO0377V4.KX907345

SYHRLRDLLL

>C.TZ.04.CO3728V2.KX907384

SYHRLRDFIL

>C.TZ.04.CO3979V3.KX907392

CYHRLRDFIL

>C.TZ.04.CO6033V4.KX907394

SYHQLRDFIL

>C.TZ.04.CO6040V4.KX907395

SYHRLRDFIL

>C.TZ.04.CO6322V4.KX907405

SYHRLRDFIL

>C.TZ.04.CO6631V3.KX907413

SYRQLRDFIL

>C.TZ.04.CO6644V2.KX907415

LYHRLRDFTL

>C.TZ.04.CO6812V3.KX907422

SYHRLRDFIL

>C.TZ.05.CO0053V5.KX907338

SYHQLRDLIL

>C.TZ.05.CO0933V4.KX907357

SYHRLRDFIL

>C.TZ.05.CO3063V5.KX907363

SYHRLRDLIL

>C.TZ.05.CO3168V4.KX907366

SYHRLRDFIL

>C.TZ.05.CO3426V5.KX907374

SYRRLRDLIL

>C.TZ.05.CO3637V5.KX907380

SYHHLRDFIL

>C.TZ.05.CO3672V4.KX907382

SYHRLRDFIL

>C.TZ.05.CO3800V5.KX907385

SYHLLRDFIL

>C.TZ.05.CO6106V5.KX907398

SYHHLRDFIL

>C.TZ.05.CO6647V5.KX907416

SYHRLRDLLL

>C.TZ.06.CO3278V6.KX907368

SYHRLRDFIL

>C.TZ.06.CO3440V7.KX907375

SYHRLRDFIL

>C.TZ.06.CO3892V6.KX907390

CYRLLIDFIL

>C.TZ.06.CO3969V6.KX907391

SYHQLRDFIL

>C.TZ.06.CO6022V7.KX907393

CYHRLRDLIL

>C.TZ.06.CO6139V7.KX907399

CYHRLRDLIL

>C.TZ.06.CO6146V7.KX907400

SYHRLRDFTL

>C.TZ.06.CO6847V7.KX907425

CYHRLRDFTL

>C.TZ.06.CO6911V7.KX907428

SYHRLRDFIL

>C.TZ.08.707010457_CH457.w8.KC156220

SYHRLRDFTL

>C.TZ.08.707010562_G9.HQ615976

CYHRLRDLLL

>C.TZ.97.97TZ04.AF361874

SYHRLRDFIL

>C.TZ.97.97TZ05.AF361875

SYRRLRDFIL

>C.TZ.98.98TZ013.AF286234

SYHRLRDFIL

>C.TZ.98.98TZ017.AF286235

SYHRLRDFIL

>C.UG.90.UG268A2.L22948

SYRRLRDLLL

>C.US.05.p07Jenv48.EU663619

SYHRLRDFIL

>C.US.11.17TB4_4G8.KF526226

SYRRLRDLIL

>C.US.14.M0118v1d14_5M04_C5.KT982201

SYHRLRDCIL

>C.US.98.98US_MSC3018.AY444800

LYHRLRDLIL

>C.US.98.98US_MSC5016.AY444801

SYHQLRDFIL

>C.US.x.V2537TOB8U.GU728173

SYHRLRDLLL

>C.UY.01.TRA3011.AY563169

SYHRLRDLIL

>C.YE.02.02YE511.AY795906

SYHRLRDFTL

>C.ZA.00.00ZAPCP1.AY529667

SYHRLRDFIL

>C.ZA.00.1069MB.AY838567

SYHRLRDFIS

>C.ZA.00.1119MB.AY463229

SYHRLRDFIL

>C.ZA.00.1134MB.AY463217

SYHQLRDFIL

>C.ZA.00.1157M3M.AY585266

SYHQLRDFIL

>C.ZA.00.1162MB.AY463224

SYHQLRNFIL

>C.ZA.00.1165MB.AY463230

SYQRLRDFIL

>C.ZA.00.1168MB.AY463231

SYHQLRDFIL

>C.ZA.00.1170MB.AY463225

SYHQLRNFIL

>C.ZA.00.1171MB.AY463232

SYHRLRDFIS

>C.ZA.00.1176MB.AY463218

SYHQLRDFTL

>C.ZA.00.1178MB.AY463233

SYHQLRDFIL

>C.ZA.00.1184MB.AY838566

SYHRLRDFIL

>C.ZA.00.1189MB.AY838565

SYHQLRDFIL

>C.ZA.00.1192M3M.AY463219

SYHLLRDFIL

>C.ZA.00.1195MB.AY463220

SYHRLRDFIL

>C.ZA.00.1197MB.AY463234

SYHQLRDFIL

>C.ZA.00.1210MB.AY463221

SYHQLRNFIL

>C.ZA.00.1214MB.AY463236

SYRQLRDFIL

>C.ZA.00.1217MB.AY463226

SYHQLRDFIL

>C.ZA.00.1225MB.AY463227

SYHQLRDFIL

>C.ZA.00.1228MB.AY463222

SYHQLRNFIL

>C.ZA.00.CM1_N1.JX845606

SYHRLRDFIL

>C.ZA.00.CM2_E1.JX845587

SYHRLRDFIS

>C.ZA.00.CM3_M11.JX845593

SYRQLRDFIL

>C.ZA.00.CM5_A1.JX845611

SYHQLRDFIL

>C.ZA.00.CP4_L2.JX845600

SYHRLRDLIS

>C.ZA.00.Du151_May00_16.KF146947

SYHRLRDLIL

>C.ZA.00.J112MA.AY838568

SYHRLRDFIL

>C.ZA.00.J38MA.AY463228

SYHHLRDFIL

>C.ZA.00.TM20_13.EU161645

SYHRLRDFIL

>C.ZA.01.01ZADU36_5.FJ846632

SYHHLRDFIL

>C.ZA.01.01ZARP1.AY529666

SYHQLRDFIL

>C.ZA.01.01ZATM18b.AY529672

SYHRLRDFIL

>C.ZA.01.01ZATM45.AY228557

SYHRLRDFIS

>C.ZA.01.2004MB.AY463235

CYRRLRDLLL

>C.ZA.01.2134MB.AY463237

SYRRLRDCIL

>C.ZA.01.C.ZA.01.Du114_B1.KC863203

SYHRLRDCIL

>C.ZA.01.J54Ma.AY463223

SYHQLRNFIL

>C.ZA.02.02ZAPS001MB1.DQ275648

SYRRLRVFIL

>C.ZA.02.02ZAPS005MB1.DQ351235

SYHRLRDFIL

>C.ZA.02.02ZAPS006MB1.DQ351220

CYHRLRDFIL

>C.ZA.02.02ZAPS008MB1.DQ275647

SYHRLRDCIL

>C.ZA.02.02ZAPS013MB1.DQ351222

SYHQLRNFIL

>C.ZA.02.02ZAPS014MB1.DQ351218

SYHQLRDFIL

>C.ZA.02.02ZAPS015MB1.DQ369995

SYHRLRGFIL

>C.ZA.02.RP4.DQ447270

SYHRLRDLIL

>C.ZA.02.RP6.DQ447269

SYHRLRDFIL

>C.ZA.03.03ZAPS017MB1.DQ351224

IYHQLRDFIL

>C.ZA.03.03ZAPS020MB1.DQ275653

SYHRLRDFIL

>C.ZA.03.03ZAPS021MB1.DQ369978

SYHQLRNFIL

>C.ZA.03.03ZAPS023MB1.DQ351225

GYHQLRDFTL

>C.ZA.03.03ZAPS024MB1.DQ396367

SYHQLRDLIL

>C.ZA.03.03ZAPS025MB1.DQ351226

SYHRLRDLIS

>C.ZA.03.03ZAPS026MB1.DQ369985

SYHRLRDFIL

>C.ZA.03.03ZAPS027MB1.DQ351223

SYHQLRDFIL

>C.ZA.03.03ZAPS030MB1.DQ369996

SYHRLRDLIL

>C.ZA.03.03ZAPS032MB1.DQ445633

SYHHLRDFIL

>C.ZA.03.03ZAPS034MB1.DQ369979

CYHQLRDFIL

>C.ZA.03.03ZAPS042MB1.DQ369977

SYHQLRDFIL

>C.ZA.03.03ZAPS043MB1.DQ351227

SYHRLRDCIL

>C.ZA.03.03ZAPS044MB1.DQ396384

CYHQLRDFIL

>C.ZA.03.03ZAPS046MB1.DQ369984

SYHLLRDFTL

>C.ZA.03.03ZAPS048MB1.DQ396364

SYHRLRDFIL

>C.ZA.03.03ZAPS049MB1.DQ369986

SYHRLRDFIL

>C.ZA.03.03ZAPS050MB1.DQ369980

LYHRLRDFIL

>C.ZA.03.03ZAPS051MB1.DQ396385

SYHQLRDFIL

>C.ZA.03.03ZAPS052MB1.DQ369987

SYHHLRDFIL

>C.ZA.03.03ZAPS054MB2.DQ369988

SYHQLRDFIL

>C.ZA.03.03ZAPS055MB1.DQ396373

SYHRLRDFIL

>C.ZA.03.03ZAPS056MB1.DQ396374

SYHQLKTFIL

>C.ZA.03.03ZAPS057MB2.DQ369989

SYHRLRDCIS

>C.ZA.03.03ZAPS063MB1.DQ396388

SYHRLRDFTL

>C.ZA.03.03ZAPS066MB2.DQ396375

SYHQLRDFIL

>C.ZA.03.03ZAPS067MB2.DQ396389

SHHQLRDFIL

>C.ZA.03.03ZAPS071MB1.DQ396376

SYHQLRDFIL

>C.ZA.03.03ZAPS073MB1.DQ275649

SYHRLRDFIL

>C.ZA.03.03ZAPS074MB2.DQ351228

SYHRLRDFIL

>C.ZA.03.03ZAPS077B1.DQ093591

SYHQLRNFIL

>C.ZA.03.03ZAPS079B1.DQ093592

SYHQLRDFIL

>C.ZA.03.03ZAPS081MB1.DQ351219

SYHRLRDFIL

>C.ZA.03.03ZAPS083MB1.DQ351229

SYHQLRDFIL

>C.ZA.03.03ZAPS086MB1.DQ275654

CYHQLRDFIL

>C.ZA.03.03ZAPS088MB1.DQ275651

CYHQLRDFIL

>C.ZA.03.03ZAPS089MB1.DQ351216

SYHQLRDFIL

>C.ZA.03.03ZAPS091MB1.DQ275645

SYHRLRDCIL

>C.ZA.03.03ZAPS094MB1.DQ396377

SYHQLRNFIL

>C.ZA.03.03ZAPS095MB1.DQ275652

SYHQLRNFTL

>C.ZA.03.03ZAPS097MB1.DQ351230

SYHQLRDFIL

>C.ZA.03.03ZAPS099MB1.DQ275655

NYHLLRDFIL

>C.ZA.03.03ZAPS103MB2.DQ275656

SYHRLRDFIL

>C.ZA.03.03ZAPS104MB1.DQ369990

SYHQLRDFIL

>C.ZA.03.03ZAPS105MB2.DQ445632

CYHQLRDFIL

>C.ZA.03.03ZAPS108MB1.DQ396378

SYHHLRDFIL

>C.ZA.03.03ZAPS112MB2.DQ396386

SYHQLRDFIL

>C.ZA.03.03ZAPS113MB2.DQ396365

SYHRLRDFIL

>C.ZA.03.03ZAPS116MB1.DQ445635

SYHRLRDFIS

>C.ZA.03.03ZAPS118MB1.DQ396368

SYHQLRDFIL

>C.ZA.03.03ZAPS122MB1.DQ396370

SYHRLRDLIL

>C.ZA.03.03ZAPS123MB1.DQ396369

SYHQLRDFIL

>C.ZA.03.03ZAPS124MB1.DQ369976

CYHRLRDFIL

>C.ZA.03.03ZAPS125MB1.DQ396390

SYHLLRDFIL

>C.ZA.03.03ZAPS126MB1.DQ275657

SYHQLRDFTL

>C.ZA.03.03ZAPS128MB1.DQ275643

SYHQLRDFIL

>C.ZA.03.03ZAPS130MB1.DQ275658

SYHRLRDFIL

>C.ZA.03.03ZAPS131MB1.DQ396380

SYHRLRDFIL

>C.ZA.03.03ZAPS133MB1.DQ275646

SYHQLRDFIL

>C.ZA.03.03ZAPS136MB1.DQ351231

SYHRLRDFIL

>C.ZA.03.03ZAPS140MB1.DQ369981

SYHHLRDFIL

>C.ZA.03.03ZAPS143MB1.DQ396391

SYHLLRDFIL

>C.ZA.03.03ZAPS151MB1.DQ396392

SYHRLRDFIL

>C.ZA.03.03ZAPS152MB1.DQ396399

SYHQLRDFIL

>C.ZA.03.03ZAPS155MB1.DQ396371

SYHHLRDFIL

>C.ZA.03.03ZASK005B2.DQ011175

SYHQLRNFIL

>C.ZA.03.03ZASK006B2.AY878056

SYHQLRDFIL

>C.ZA.03.03ZASK010B2.DQ164104

SYHQLRDFIL

>C.ZA.03.03ZASK011B2.AY901965

SYHQLRDFIL

>C.ZA.03.03ZASK013B2.DQ275660

SYHLLRDFIL

>C.ZA.03.03ZASK016MB2.DQ351233

SYHRLRDFIL

>C.ZA.03.03ZASK019B2.AY878063

SYHQLRDFIL

>C.ZA.03.03ZASK020B2.AY878064

CYHLLRDFIS

>C.ZA.03.03ZASK026B2.DQ011165

CYHRLRDFIL

>C.ZA.03.03ZASK034B1.AY878065

SYHRLRDCIL

>C.ZA.03.03ZASK036B1.AY901966

LYHRLRDCIL

>C.ZA.03.03ZASK039B2.AY878068

SYRQLRTFIL

>C.ZA.03.03ZASK042B2.AY878069

SYHQLRDFIL

>C.ZA.03.03ZASK058B2.AY901967

SYHQLRDFIL

>C.ZA.03.03ZASK061B1.AY901968

SYHQLRDFIL

>C.ZA.03.03ZASK062B1.DQ164113

CYHRLRDFIL

>C.ZA.03.03ZASK066B1.AY901969

SYHHLRDLLL

>C.ZA.03.03ZASK067B1.DQ275642

SYHQLRDFIL

>C.ZA.03.03ZASK072B1.DQ093593

SYHQLRNFIL

>C.ZA.03.03ZASK073B1.AY901970

SYRQLRDFIL

>C.ZA.03.03ZASK076B1.AY901975

SYHLLRDFIL

>C.ZA.03.03ZASK078B1.AY901971

SYHRLRDFIS

>C.ZA.03.03ZASK084B1.AY901981

LYHRLRDCTL

>C.ZA.03.03ZASK092B1.AY878057

SYHQLRDFIL

>C.ZA.03.03ZASK094B1.AY878070

SYHHLRDLLL

>C.ZA.03.03ZASK097B1.AY878060

CYHLLRDFIL

>C.ZA.03.03ZASK098B1.AY878061

LYHRLRDFML

>C.ZA.03.03ZASK103B1.DQ164106

SYHRLRDFIL

>C.ZA.03.03ZASK104B1.DQ396395

SYHLLRDFIL

>C.ZA.03.03ZASK107B1.DQ056410

SYHRLRDFIL

>C.ZA.03.03ZASK110B1.DQ056411

SYHLLRDFIL

>C.ZA.03.03ZASK111B1.DQ056404

SYHRLRDFIL

>C.ZA.03.03ZASK113B1.DQ351237

SYHQLRDFIL

>C.ZA.03.03ZASK117B1.DQ056408

CYHRLRDFIL

>C.ZA.03.03ZASK118B1.DQ011169

SYHQLRNLIL

>C.ZA.03.03ZASK120B1.DQ011176

CYHQLRDFIL

>C.ZA.03.03ZASK211B1.DQ093601

SYHQLRDFIL

>C.ZA.03.03ZASK212B1.DQ093596

SYHRLRDFIL

>C.ZA.03.03ZASK213B1.DQ093607

SYHRLRDLIL

>C.ZA.03.03ZASK215M6W.DQ275661

SYHQLRDFIL

>C.ZA.03.03ZASK223B1.DQ093597

SYHRLRDFIL

>C.ZA.03.03ZASK224MB1.DQ275664

SYHLLRDFIL

>C.ZA.03.03ZASK226B1.DQ164108

SYHRLRDFIL

>C.ZA.03.03ZASK232B1.DQ093589

SYHLLRDCTL

>C.ZA.03.03ZASK233B1.DQ351234

CYRRLRDFIL

>C.ZA.03.04ZAPS194MB1.DQ275650

SYHRLRDFIL

>C.ZA.03.04ZASK082B1.AY901972

SYHRLRDCIL

>C.ZA.03.503_14197_T1.KT183301

SYRQLRDFIL

>C.ZA.03.C.ZA.x.Du246_B3.KC863229

SYHRLRDFIL

>C.ZA.03.C.x.03.Du10_F1.KC863250

SYHRLRDFIL

>C.ZA.03.SK023B2.AY772690

LYRQLRDCIL

>C.ZA.03.SK029B2.AY772691

SYHRLRDFIL

>C.ZA.03.SK033B2.AY772692

SYHRLRDSIL

>C.ZA.03.SK040B1.AY703908

CYHQLRTFIL

>C.ZA.03.SK041B1.AY772693

SYHRLRDFIL

>C.ZA.03.SK043B1.AY772700

SYHRLRDFIL

>C.ZA.03.SK065B1.AY772694

IYHRLRDFIL

>C.ZA.03.SK091B1.AY772701

SYHQLRDFIL

>C.ZA.03.SK112B1.AY772695

SYHHLRDFIL

>C.ZA.03.SK116B1.AY772696

SYHQLRDFIL

>C.ZA.04.04ZAPS157MB1.DQ351232

SYHRLRDFTL

>C.ZA.04.04ZAPS160B1.DQ164107

SYHQLRNFIL

>C.ZA.04.04ZAPS161B1.DQ164115

LYHQLRDFTL

>C.ZA.04.04ZAPS165MB1.DQ351221

SYHQLRDFIL

>C.ZA.04.04ZAPS168B1.DQ164121

SYHRLRDFIL

>C.ZA.04.04ZAPS169MB1.DQ396393

SYHRLRDFIL

>C.ZA.04.04ZAPS172MB1.DQ275659

SYHHLRDFIL

>C.ZA.04.04ZAPS177MB1.DQ396394

SYHRLRDFLL

>C.ZA.04.04ZAPS188B1.DQ164122

SYHRLRDFIL

>C.ZA.04.04ZAPS189B1.DQ164117

SYHQLRDFIL

>C.ZA.04.04ZAPS190B1.DQ093602

SYHRLRDFIL

>C.ZA.04.04ZAPS195B1.DQ164118

SYHRLRDFIL

>C.ZA.04.04ZAPS197MB1.DQ369997

SYHRLRDFIL

>C.ZA.04.04ZAPS198MB1.DQ445637

SYHRLRDFIL

>C.ZA.04.04ZAPS199B1.DQ164110

SYHHLRDFVL

>C.ZA.04.04ZAPS202B1.DQ093598

SYHRLRDFIL

>C.ZA.04.04ZAPS205B1.DQ093599

SYHRLRDLLS

>C.ZA.04.04ZAPS206B1.DQ164126

LYQRLRDFIL

>C.ZA.04.04ZAPS214B1.DQ093588

SYHQLRDFIL

>C.ZA.04.04ZAPS216B1.DQ093600

SYHQLRDFIL

>C.ZA.04.04ZAPS217B1.DQ164119

LYHHLRDFIL

>C.ZA.04.04ZASK031B2.AY901974

SYHRLRDFIL

>C.ZA.04.04ZASK083B2.DQ093585

SYHLLRNFIL

>C.ZA.04.04ZASK127B1.DQ011172

SYHLLRDFIL

>C.ZA.04.04ZASK128B1.DQ093586

SYHRLRDFIL

>C.ZA.04.04ZASK131B1.DQ056412

SYHQLRDFIL

>C.ZA.04.04ZASK132B1.DQ093594

SYHQLRDFIL

>C.ZA.04.04ZASK135B1.DQ011166

SYRHLRDFIL

>C.ZA.04.04ZASK136B1.DQ011177

SYHRLRDFIL

>C.ZA.04.04ZASK139B1.AY878072

SYRQLRNFIL

>C.ZA.04.04ZASK142B1.DQ011167

LYHHLRDFIL

>C.ZA.04.04ZASK145B1.AY901976

SYHQLRDFIL

>C.ZA.04.04ZASK146.AY772699

SYHRLRDFIL

>C.ZA.04.04ZASK147B1.DQ164114

SYHHLRDFIL

>C.ZA.04.04ZASK148B1.DQ093590

SYHQLRDFIL

>C.ZA.04.04ZASK150B1.DQ011178

LYHQLRDFIL

>C.ZA.04.04ZASK151B1.AY901977

SYHLLRDCIL

>C.ZA.04.04ZASK154B1.AY878071

SYHQLRNFIL

>C.ZA.04.04ZASK155B1.AY901978

SYHQLRDFIL

>C.ZA.04.04ZASK156B1.DQ011171

SYHRLRDLLL

>C.ZA.04.04ZASK159B1.DQ011179

SYHRLRDFIL

>C.ZA.04.04ZASK160B1.DQ011173

CYHQLRDFIL

>C.ZA.04.04ZASK161B1.DQ011170

SYHQLRDFIL

>C.ZA.04.04ZASK163B1.AY901979

SYHQLRDFIL

>C.ZA.04.04ZASK164B1.DQ056405

SYHQLRDFIL

>C.ZA.04.04ZASK165B1.DQ396387

SYHRLRDFIL

>C.ZA.04.04ZASK167B1.DQ164127

SYHRLRDFIL

>C.ZA.04.04ZASK168B1.AY878058

SYHQLRNFIL

>C.ZA.04.04ZASK169B1.DQ396381

SYHRLRDFIL

>C.ZA.04.04ZASK170B1.DQ093595

SYHRLRDFIL

>C.ZA.04.04ZASK171B1.DQ351217

SYHRLRDFIL

>C.ZA.04.04ZASK173B1.DQ093604

SYHRLRDLTL

>C.ZA.04.04ZASK174B1.AY901980

SYHRLRDFIL

>C.ZA.04.04ZASK175B1.DQ164129

SYRQLRDFIL

>C.ZA.04.04ZASK176B1.DQ056416

SYHRLRDFIL

>C.ZA.04.04ZASK178B1.DQ093587

SYHQLRNFTL

>C.ZA.04.04ZASK180B1.AY878059

SYHLLRDFIL

>C.ZA.04.04ZASK181B1.AY878062

CYHRLRDFIL

>C.ZA.04.04ZASK182B1.AY878054

LYHQLRDFIL

>C.ZA.04.04ZASK183B1.AY878055

SYHQLRDFTL

>C.ZA.04.04ZASK184B1.DQ056418

SYHRLRDFIL

>C.ZA.04.04ZASK185B1.DQ011174

SYHQLRNFIL

>C.ZA.04.04ZASK190B1.DQ056409

SYHHLRDFIL

>C.ZA.04.04ZASK191B1.DQ369993

SYHRLRDLIL

>C.ZA.04.04ZASK192B1.DQ396382

SYHQLRDFVL

>C.ZA.04.04ZASK193B1.DQ396396

SYHQLRDFIL

>C.ZA.04.04ZASK196B1.DQ056413

SYHRLRDFIL

>C.ZA.04.04ZASK200B1.DQ396383

SYHLLRDFIL

>C.ZA.04.04ZASK201B1.DQ396397

SYHRLRDFIL

>C.ZA.04.04ZASK202B1.DQ011180

SYHRLRDFIL

>C.ZA.04.04ZASK204B1.DQ056414

SYHRLRDFIL

>C.ZA.04.04ZASK206B1.DQ056415

SYHRLRDLIL

>C.ZA.04.04ZASK208B1.DQ056406

SYHHLRDFIL

>C.ZA.04.04ZASK217B1.DQ056417

CYHRLRDLTL

>C.ZA.04.04ZASK234B1.DQ093605

SYHRLRDFLL

>C.ZA.04.CAP30_5w_F4.GQ999973

SYHRLRDFIL

>C.ZA.04.CAP61_8w_F3.GQ999975

SYHRLRDCIS

>C.ZA.04.SK134B1.AY703909

SHHRLRDFIL

>C.ZA.04.SK140B1.AY901973

SYHHLRDFIL

>C.ZA.04.SK143B1.AY703910

SYHLLRDFIL

>C.ZA.04.SK144B1.AY703911

SYHRLRDLLS

>C.ZA.05.05ZAFV11.DQ382369

SYHQLRDFVL

>C.ZA.05.05ZAFV12.DQ382370

SYHRLRDFIL

>C.ZA.05.05ZAFV13.DQ382371

CYHQLRDFIL

>C.ZA.05.05ZAFV14.DQ382372

CYHRLRDFIL

>C.ZA.05.05ZAFV15.DQ382373

SYRQLRDFIL

>C.ZA.05.05ZAFV20.DQ382374

SYHRLRDFTL

>C.ZA.05.05ZAFV22.DQ382375

SYHRLRDFIL

>C.ZA.05.05ZAFV23.DQ382376

SYHRLRDFIL

>C.ZA.05.05ZAFV25.DQ382377

CYHRLRDFIL

>C.ZA.05.05ZAFV26.DQ382378

SYHQLRSFIL

>C.ZA.05.05ZAFV27.DQ382379

SYHLLRDFIL

>C.ZA.05.05ZAFV28.DQ382380

SYHRLRDFIL

>C.ZA.05.05ZAFV2.DQ382361

SYHLLRDFIL

>C.ZA.05.05ZAFV3.DQ382362

SYHRLRDFIL

>C.ZA.05.05ZAFV5.DQ382363

SYHRLRDFIL

>C.ZA.05.05ZAFV6.DQ382364

SYHRLRDLIL

>C.ZA.05.05ZAFV7.DQ382365

SYHLLRDFIL

>C.ZA.05.05ZAFV8.DQ382366

SYHQLRDFIL

>C.ZA.05.05ZAFV9.DQ382367

IYHRLRDFIL

>C.ZA.05.05ZAPSK240B1.DQ369991

SYHRLRDFIL

>C.ZA.05.05ZASK243B1.DQ396372

SYHLLRDFIL

>C.ZA.05.05ZASK244B1.DQ369992

SYHRLRDFTL

>C.ZA.05.05ZASK245B1.DQ369982

SYHRLRDFIL

>C.ZA.05.05ZASK246B1.DQ369983

CYHQLSNFIL

>C.ZA.05.05ZASK247B1.DQ369994

SYHQLRDFIL

>C.ZA.05.18814602_H8_F3.HQ615948

SYHRLRDCIL

>C.ZA.05.19157834_3A5.JQ754193

SYRLLKDFIL

>C.ZA.05.19252094_A5_G2.HQ615953

SYHRLRDFIL

>C.ZA.05.19314479_A2_5.HQ615958

LYHRLTDFIW

>C.ZA.05.CAP174_4w.GQ999981

CYHQLRDFVL

>C.ZA.05.CAP200_B8a.KC894135

SYHQLRDFIL

>C.ZA.05.CAP206_8w_F1.GQ999982

SYHQLRDFIL

>C.ZA.05.CAP210_5w.GQ999983

CYHRLRDLLL

>C.ZA.05.CAP228_8w_F2.GQ999984

SYHHLRDFIL

>C.ZA.05.CAP229_7w.GQ999985

SYHQLRDFIL

>C.ZA.05.CAP244_8w_F1.GQ999986

SYHRLRDFIL

>C.ZA.05.CAP248_9w.GQ999987

SYHQLRDFIL

>C.ZA.05.CAP255_8w_F1.GQ999988

SYHLLRDFIL

>C.ZA.05.CAP256_6w.GQ999989

SYHHLRDFTL

>C.ZA.05.CAP257_7w_F1.GQ999990

SYRHLRDFIL

>C.ZA.05.CAP258_2_00_X_23.JN681227

SYHQLRNFIL

>C.ZA.05.CAP45_5w_F1.GQ999974

SYHRLRDFIL

>C.ZA.05.CAP63_5w_F4.GQ999976

SYRQLRNFIL

>C.ZA.05.CAP65_6w.GQ999977

SYHRLRDLLS

>C.ZA.05.CAP84_3w_F2.GQ999978

CYHQLRDFIL

>C.ZA.05.CAP85_5w_F1.GQ999979

FYHRLRDFIL

>C.ZA.05.CAP88_5w_F2.GQ999980

SYHLLRDFIL

>C.ZA.05.CAP8_3w_F2.GQ999972

SYHRLRDFIL

>C.ZA.05.CHV0008955_CAP225.1.06C4.FJ443982

SYHHLRDFIL

>C.ZA.05.CHV0008960_CAP217.1.07H2.FJ443986

SYHQLRDFIL

>C.ZA.06.19705993.HQ595744

SYHRLRDFIL

>C.ZA.06.19707346_E8_C6.HQ615951

SYHQLRNFIL

>C.ZA.06.19738501.HQ595745

SYHRLRDFIS

>C.ZA.06.2759058_F10_B6.HQ615959

SYRHLRDFIL

>C.ZA.06.2768732_C5_16.HQ615952

SYHRLRDFIL

>C.ZA.06.2833264.HQ595757

SYHRLRDFLL

>C.ZA.06.704010017_B7.KC894111

SYHLLRDFIL

>C.ZA.06.BP00031_env.JN687821

SYHRLRDFIL

>C.ZA.06.C.x.06.CF04_A2.KC863324

SYHQLRDFVL

>C.ZA.06.C.x.06.CF05_H4.KC863361

LYHRLRDFIL

>C.ZA.06.C.x.06.CF08_D9g.KC863367

CYHRLTDFIL

>C.ZA.06.CAP177_1A3.KC894133

SYHQLRDFIL

>C.ZA.06.CAP221_B14.KC894137

SYHQLRDFIL

>C.ZA.06.CAP228_4170_069wpi_A24.MK205450

SYHQLRNFIL

>C.ZA.06.CAP266_2_00_E9_H6.JN681229

SYHQLRDFIL

>C.ZA.06.CHV0005480_CAP69.1.12TA8.FJ443300

SYHLLRDFIL

>C.ZA.06.CHV0005989_CAP129.1.15B2.FJ443417

SYHRLRDFIL

>C.ZA.06.CHV0005998_CAP37.1.18G1.FJ443426

SYHQLRDLIL

>C.ZA.06.CHV0006046_CAP224.1.18B11.FJ443473

SYHRLRDFLL

>C.ZA.06.CHV0006062_CAP260.2.00G11.FJ443488

SYHQLRDFIL

>C.ZA.06.CHV0006073_CAP136.1.16Tb5.FJ443498

CYHQLRDFIL

>C.ZA.06.CHV0006091_CAP222.1.11A6.FJ443515

SYHQLRNFIL

>C.ZA.06.CHV0008645_CAP269.2.00G7.FJ443911

SYHRLRDFIL

>C.ZA.07.19715820_A10_H2.HQ615950

SYHQLRDFIL

>C.ZA.07.20104663_E11_D2.HQ615945

SYHRLRDFIL

>C.ZA.07.20198102_E9_G1.HQ615944

SYHRLKDFIS

>C.ZA.07.20258279_v1.HQ595762

SYHRLRDLLL

>C.ZA.07.20296368.HQ595746

SYHRLRDFIL

>C.ZA.07.2891391_A2_E1.HQ615955

SYHRLRDFIL

>C.ZA.07.2935054.HQ595758

CYHRLRDLLL

>C.ZA.07.2969249.HQ595766

SYHQLRNFIL

>C.ZA.07.34_D12.KC894386

SYHHLRDFIL

>C.ZA.07.3514597.HQ595759

LYHQLRDFLL

>C.ZA.07.3545883_G1_E1.HQ615942

SYHQLRNFIL

>C.ZA.07.503_01009_4.KT183064

SYHRLRDFLS

>C.ZA.07.503_02051_66.KT183087

CYHRLRDFIL

>C.ZA.07.503_02182_B5.KT183090

SYHRLRDLLL

>C.ZA.07.503_02660_1.KT183094

SYHQLRNFIL

>C.ZA.07.503_02854_C1.KT183103

SYHLLRDFIS

>C.ZA.07.503_05649_G6.KT183148

SYHRLRDLIL

>C.ZA.07.503_07352_C1.KT183196

SYHQLRDFIL

>C.ZA.07.503_07518_E9.KT183211

SYHQLRDFIL

>C.ZA.07.503_07711_D6.KT183216

SYHQLRDFIL

>C.ZA.07.503_09003_5.KT183229

SYHRLRDFIL

>C.ZA.07.503_10430_2.KT183253

SYHRLRDFLS

>C.ZA.07.503_11204_39.KT183265

SYHRLRDFLS

>C.ZA.07.503_11441_B1.KT183271

SYHRLRDFIL

>C.ZA.07.503_13503_A5.KT183279

SYHQLRDFIL

>C.ZA.07.5456037.HQ595761

LYHRLRDFIL

>C.ZA.07.704010042_CH042_TF.KC156123

SYHQLRDFIL

>C.ZA.07.704010069_C6.KC894114

SYHQLRNFIL

>C.ZA.07.704010083_B8.KC894116

SYHQLRNFIL

>C.ZA.07.704010124_E6.KC894117

SYHQLRDFIL

>C.ZA.07.704809221_1B3.KC894118

CYHQLRNFIL

>C.ZA.07.704810053_2B7.KC894119

SYHRLRDFIL

>C.ZA.07.705010067_CH067_TF.KC156125

SYRQLRDFIL

>C.ZA.07.705010154_E4.KC894120

SYHQLRDFIL

>C.ZA.07.705010162_CH162.mo6.KC156115

SYRRLRDFIL

>C.ZA.07.705010198_CH198_TF.KC156130

SYHLLRDFIL

>C.ZA.07.706010018_2E3.KC894124

SYHHLRDFIL

>C.ZA.07.706010164_CH164_TF.KC156127

SYHRLRDFLL

>C.ZA.07.75_C5.KC894115

SYHRLRDFIL

>C.ZA.07.BP00005_RH03.JN687704

SYHRLRDCIL

>C.ZA.07.BP00008_RH01.JN687706

SYHRLRDFIL

>C.ZA.07.BP00009_SUP_RH01.JN687717

SYHQLRNFIL

>C.ZA.07.BP00010_RH01.JN687718

SYHRLRDFIL

>C.ZA.07.BP00029_RH01.JN687735

SYHRLRDFIL

>C.ZA.07.BP00035_RH01.JN687736

SYHRLRDFLL

>C.ZA.07.C.CAP239.w117.805dps.4_21_T44.JX976681

SYHRLRDFIL

>C.ZA.07.C.x.07.CF09_A6.KC863573

SYHRLRDFIL

>C.ZA.07.C.x.07.CF10_D10.KC863587

CYHQLRDFIL

>C.ZA.07.C.x.07.CF13_A2.KC863599

SYHRLRDFIL

>C.ZA.07.C.x.07.CF17_G11b.KC863629

SYRQLRDFIL

>C.ZA.07.C.x.07.CF21_H2.KC863647

SYHQLRNFIL

>C.ZA.07.CH010028_w24_p1.HM204581

SYHQLRDFIL

>C.ZA.07.CH010090_w8_p1.HM204585

SYHQLRDFIL

>C.ZA.07.CH010207_w4_p1.HM204594

SYHRLRDFIL

>C.ZA.07.CH010210_w2_p2.HM204595

SYHLLRDFIL

>C.ZA.07.CH010273_w4_p1.HM204598

SYHQLRDFIL

>C.ZA.07.CH010298_w12_p1.HM204600

LYRQLRDFIL

>C.ZA.07.CH010301_w12_p1.HM204601

SYHQLRDFIL

>C.ZA.07.CH010316_w16_p1.HM204602

SYHRLRDLIS

>C.ZA.07.CH010330_w16_p1.HM204604

SYHQLRDFIL

>C.ZA.07.CH010343_w12_p1.HM204605

SYHRLRDFIL

>C.ZA.07.CH010355_w2_p1.HM204606

CYHRLRDFIL

>C.ZA.07.CH010368_w8_p2.HM204607

SYHQLRDFIL

>C.ZA.07.CH010383_w12_p1.HM204608

SYHQLRSFIL

>C.ZA.07.CH010384_w16_p2.HM204609

SYHRLRDFIL

>C.ZA.07.CH010392_w4_p2.HM204610

SYHRLRDFLL

>C.ZA.07.CH010408_w12_p1.HM204611

SYHRLRDFIL

>C.ZA.07.CH010453_w12_p3.HM204615

SYHRLRDFIL

>C.ZA.07.CH010461_w12_p1.HM204616

SYHRLRDFIL

>C.ZA.07.CHV0008259_705010026_G3.FJ443547

SYHQLRTFIL

>C.ZA.07.CHV0008370_704010056_F8.FJ443732

SYHQLRDFIL

>C.ZA.07.CHV0008570_CAP237.1.22C5.FJ443838

SYHRLRDFIS

>C.ZA.07.CHV0008598_CAP40.2.01F2.FJ443865

SYHQLRNFIL

>C.ZA.07.CHV0008639_705010015_H7.FJ443905

SYHQLRDFIL

>C.ZA.07.CHV0008962_705010110_F12.FJ443988

SYHRLRDLIL

>C.ZA.07.CHV0010304_706010151_2B2.FJ444091

IYHQLRTFIL

>C.ZA.07.DEMC07ZA011.JX140664

SYHQLRDFIL

>C.ZA.08.1245045.HQ595742

SYHRLRDFIL

>C.ZA.08.20721190.HQ595747

SYHRLRDFIL

>C.ZA.08.20883229_C9_H6.HQ615943

SYHRLRDFIS

>C.ZA.08.20915593.HQ595749

SYHRLTDFIS

>C.ZA.08.20927783.HQ595750

SYHQLRDFIL

>C.ZA.08.21203310_G7_C3.HQ615946

SYHRLRDLIL

>C.ZA.08.499_F5a.JQ777160

SYHRLRDFAL

>C.ZA.08.503_00146_D3.KT183053

CYHRLRDFIL

>C.ZA.08.503_00885_10.KT183056

SYHRLRDFIL

>C.ZA.08.503_01918_H10.KT183083

SYHRLRDFIL

>C.ZA.08.503_03257_D1b.KT183125

SYHRLRDFIL

>C.ZA.08.503_03576_12.KT183128

CYHRLKDCAL

>C.ZA.08.503_04394_C2.KT183135

SYHRLRDFLL

>C.ZA.08.503_05780_4.KT183153

SYHQLRDFIL

>C.ZA.08.503_06150_1.KT183155

SYRQLRDFIS

>C.ZA.08.503_06310_27.KT183168

CYHRLRDFIL

>C.ZA.08.503_06485_10_2.KT183172

SYHRLRDLLL

>C.ZA.08.503_06877_E1.KT183183

SYRRLRDCAL

>C.ZA.08.503_07001_A1.KT183188

SYHQLRDFIL

>C.ZA.08.503_07464_A5.KT183201

SYHRLRDFIL

>C.ZA.08.503_08252_19.KT183218

SYHLLRDFIL

>C.ZA.08.503_09289_T4.KT183243

SYHRLRDFIL

>C.ZA.08.503_10071_D2B.KT183245

SYHRLRDFIL

>C.ZA.08.503_10230_16.KT183250

SYHRLRDFLL

>C.ZA.08.503_11070_A10.KT183258

SYHQLRDFIL

>C.ZA.08.503_11474_32.KT183274

SYHLLRDCIS

>C.ZA.08.503_15405_30.KT183336

SYHRLRDCLL

>C.ZA.08.705010121_E9.HQ615983

SYHQLRDFLL

>C.ZA.08.705010185_CH185.mo6.KC156116

SYHQLRDFIL

>C.ZA.08.705010528_G3.HQ615966

SYHRLRDFIL

>C.ZA.08.705010534_CH534.w12.KC156221

SYHRLRDFIL

>C.ZA.08.706010471_F4.HQ615967

SYHQLRDFIL

>C.ZA.08.707PKE01F1.HM623548

SYRQLRNFIL

>C.ZA.08.707PKE02F4B.HM623549

CYHRLRDFIL

>C.ZA.08.707PKE04F1.HM623550

SYHQLRNFIL

>C.ZA.08.707PKE05F1.HM623551

CYHQLRDFIL

>C.ZA.08.707PKE06F1.HM623552

SYHRLRDLIL

>C.ZA.08.707PKE07F6.HM623553

SYHRLRDCIL

>C.ZA.08.707PKE09F1.HM623554

SYHRLRDFIL

>C.ZA.08.707PKE10F1.HM623555

SYHHLRDFIL

>C.ZA.08.707PKE11F2.HM623556

SYHRLRDFIL

>C.ZA.08.707PKE12F4.HM623557

SYHRLRDFIL

>C.ZA.08.707PKE15F1.HM623559

SYHQLRDFIL

>C.ZA.08.707PKE17F1.HM623561

SYHRLRDFIS

>C.ZA.08.707PKE18F2.HM623562

SYHLLRDFTL

>C.ZA.08.707PKE19F1.HM623563

SYRQLRDFIL

>C.ZA.08.707PKE21F2.HM623564

SYHRLRDFAL

>C.ZA.08.707PKE23F1.HM623566

SYHQLRDFIL

>C.ZA.08.707PKE24F1.HM623567

SYRHLRDFIL

>C.ZA.08.707PKE25F1.HM623568

SYHRLRDFIL

>C.ZA.08.707PKE26F5.HM623569

SYHQLRDFIL

>C.ZA.08.707PKE27F2.HM623570

SYHLLRDFIL

>C.ZA.08.707PKE28F1.HM623571

SYHRLRDFIL

>C.ZA.08.707PKE29F2.HM623572

LYHRLRDFIL

>C.ZA.08.707PKE31F2.HM623573

SYHRLRDFIL

>C.ZA.08.707PKE33F1.HM623574

SYHQLRDFIL

>C.ZA.08.707PKE34F2.HM623575

NYHLLRDFIL

>C.ZA.08.707PKE35F1.HM623576

SYHRLRDFIL

>C.ZA.08.707PKE36F1.HM623577

CYHRLRDFIL

>C.ZA.08.707PKE38F2.HM623579

SYHRLRDFIL

>C.ZA.08.707PKE39F1.HM623580

SYHLLRDFIL

>C.ZA.08.707PKE40F5.HM623581

SYHQLRDFIL

>C.ZA.08.707PKE43F2.HM623582

SYHQLRDFIL

>C.ZA.08.BP00016_RH01.JN687726

SYHRLRDFIL

>C.ZA.08.BP00018_RH01.JN687728

SYHLLRDFIL

>C.ZA.08.BP00023_RH02.JN687730

SYHQLRDFVL

>C.ZA.08.BP00025_RH02.JN687731

SYHQLRDFIL

>C.ZA.08.BP00028_RH01.JN687734

SYHHLRDFIL

>C.ZA.08.CAP291.2.00_H2.15.KF114884

FYHQLRDFTL

>C.ZA.08.CAP301_2_00_C3_20.KC154017

SYHQLRNFIL

>C.ZA.08.CAP304_2_00_F6_6.KC154018

SYHLLRDFIL

>C.ZA.08.CAP306_2_00_F9_1.KC154019

SYHRLRDCVL

>C.ZA.08.CAP308_2_00_E11_35.KC154020

SYHRLRDFVL

>C.ZA.08.CAP311_2_00_G5_1.KC154021

SYHRLRDFIL

>C.ZA.08.CAP317_2_00_D4_10.KC154022

SYHHLRDFIL

>C.ZA.08.CAP323_2_00_B6_45.KC154023

SYHRLRDFIL

>C.ZA.08.CAP326_2_00_D9_2.KC154024

SYHRLRDFIL

>C.ZA.08.CAP327.2.00_C6.37.KF114885

SYHQLRDFIL

>C.ZA.08.CAP330_2_00_F2_41.KC154025

IYHRLRDFIL

>C.ZA.08.CH0078.w60.e12.KC149008

SYHQLRDFIL

>C.ZA.08.CH010540_e_p1.HM204617

SYHRLRDFIL

>C.ZA.08.CH010581_e_p1.HM204618

SYHRLRDFIL

>C.ZA.08.CH010605_w12_p1.HM204619

SYHRLRDFIL

>C.ZA.08.CH0534_e1.HM204624

SYHRLRDFIS

>C.ZA.08.DEMC08ZA011.JX140666

SYHQLRNFIL

>C.ZA.08.Me178_G6.16.KF114893

SYHQLRNFIL

>C.ZA.08.SO032_A2.8_1.KF114894

SYHRLRDLLL

>C.ZA.08.TRP290_2_00_23_6.JN681253

SYHQLRNFIL

>C.ZA.08.TRP292_2_00_12_4.JN681254

SYHQLRNFIL

>C.ZA.08.TRP307_2_00_24_1.JN681255

SYHRLRDLIS

>C.ZA.08.TRP310_2_00_20_2.JN681256

SYHLLRDFIL

>C.ZA.09.20286961_C1_H8.HQ615960

SYHLLRDFIL

>C.ZA.09.20965238.HQ595751

SYHQLRDFIL

>C.ZA.09.20970668.HQ595752

IYHRLRDFIL

>C.ZA.09.21197826_3G10.JQ754244

SYHRLRDFIL

>C.ZA.09.21200845.HQ595765

SYHRLRDFIL

>C.ZA.09.21261106_C12_H2.HQ615956

CYHRLTDFIL

>C.ZA.09.21283649.HQ595756

SYHQLRDFIL

>C.ZA.09.21369737_G11_F2.HQ615941

SYHRLRDFIL

>C.ZA.09.21399975_E2_B3.HQ615957

SYHRLRDFIL

>C.ZA.09.21492713_B11_E3.HQ615947

CYHQLRDFIL

>C.ZA.09.21561324_D3_B5.HQ615961

SYHRLRDLLS

>C.ZA.09.3611665.HQ595760

SYHRLKDFIL

>C.ZA.09.503_01244_10.KT183068

SYHRLRDFIL

>C.ZA.09.503_02869_RH10.KT183114

SYHQLRNFIL

>C.ZA.09.503_05130_2.KT183141

CYHQLRDFIL

>C.ZA.09.503_13580_04.KT183289

SYHRLRDLLL

>C.ZA.09.704MC003N.GU080161

SYHLLRDFVL

>C.ZA.09.704MC004N.GU080162

SYHQLRNCIL

>C.ZA.09.704MC007F.GU080164

SYHRLRDCIS

>C.ZA.09.704MC008F.GU080165

CYHRLRDFIS

>C.ZA.09.704MC008N.GU080166

SYHLLRDFIL

>C.ZA.09.704MC010N.GU080168

SYHRLRDFVL

>C.ZA.09.704MC013F.GU080171

CYHQLRDFIL

>C.ZA.09.704MC016N.GU080173

SYHQLRDFIL

>C.ZA.09.704MC018F.GU080176

SYHRLRDFIL

>C.ZA.09.704MC019F.GU080178

SYHHLRDFIL

>C.ZA.09.704MC019N.GU080179

SYHRLRDFIL

>C.ZA.09.704MC020F.GU080180

CYHQLRDFIL

>C.ZA.09.704MC020N.GU080181

SYHRLRDCIL

>C.ZA.09.704MC021F.GU080182

SYHRLRDFIL

>C.ZA.09.704MC021N.GU080183

SYHQLRDFIL

>C.ZA.09.704MC022F.GU080184

SYHRLRDFIL

>C.ZA.09.704MC023F.GU080185

CYHQLRDFIL

>C.ZA.09.704MC024F.GU080186

IYHRLRDFIL

>C.ZA.09.704MC024N.GU080187

SYHRLRDFIL

>C.ZA.09.704MC028N.GU080190

SYHHLRDFIL

>C.ZA.09.704MC029N.GU080191

SYHRLRDLLS

>C.ZA.09.704MC030N.GU080192

SYHRLRDFIL

>C.ZA.09.704MC033F.GU080194

SYHRLRDFIL

>C.ZA.09.704MC034F.GU080195

SYHRLRDFIL

>C.ZA.09.704MC035N.GU080197

SYHRLRDFIL

>C.ZA.09.704MC037F.GU080198

SYHQLRDFIL

>C.ZA.09.704MC045F.GU080199

SYHQLRDFLL

>C.ZA.09.704Mc031F.GU080193

SYHQLRDFIL

>C.ZA.09.707PKE03N1.HM623590

SYHRLRDFIL

>C.ZA.09.707PKE04N1.HM623591

SYHQLRNFIL

>C.ZA.09.707PKE05N1.HM623592

SYHRLRDLIL

>C.ZA.09.707PKE06N1.HM623593

CYHRLRDLLL

>C.ZA.09.707PKE10N1.HM623595

SYHRLRDFIL

>C.ZA.09.707PKE11N2.HM623596

SYHRLRDFIL

>C.ZA.09.707PKE13N2.HM623597

SYHQLRNFIL

>C.ZA.09.707PKE15N2.HM623598

SYHQLRNFVL

>C.ZA.09.707PKE17N8.HM623599

SYHHLRDFIL

>C.ZA.09.707PKE18N3.HM623600

SYHQLRDFIL

>C.ZA.09.707PKE20N1.HM623601

SYHRLRDFVL

>C.ZA.09.707PKE21N1.HM623602

SYHRLRDFIL

>C.ZA.09.707PKE22N2.HM623603

SYHRLRDCIL

>C.ZA.09.707PKE23N2.HM623604

SYHQLRDFTL

>C.ZA.09.707PKE24N5.HM623605

SYHHLRTFIL

>C.ZA.09.707PKE25N6.HM623606

SYHRLRDFIS

>C.ZA.09.707PKE26N5.HM623607

SYHRLRDFLL

>C.ZA.09.707PKE27N1.HM623608

SYHRLRDFIL

>C.ZA.09.707PKE28N1.HM623609

SYHQLRNFIL

>C.ZA.09.707PKE29N1.HM623610

SYHQLRDFIL

>C.ZA.09.707PKE41N2.HM623611

SYHRLRDFIL

>C.ZA.09.707PKE44F2.HM623583

SYHRLRDCIL

>C.ZA.09.707PKE49F2.HM623584

SYHRLRDLIL

>C.ZA.09.707PKE50F1.HM623585

SYHRLRDSIL

>C.ZA.09.707PKE51F3.HM623586

SYHQLRDFIL

>C.ZA.09.707PKE52F1.HM623587

CYHQLRDFIL

>C.ZA.09.CAP200_4250_176wpi_E1.MK205709

SYHQLRDFIL

>C.ZA.09.CAP331.2.00_D7.39.KF114886

LYHRLRDSIL

>C.ZA.09.CAP332.2.00_C10.1.KF114887

SYHRLRDFIL

>C.ZA.09.CAP340_2_00_B3_16.KC154026

SYHHLRDFIL

>C.ZA.09.CAP341_2_00_C10_18.KC154027

SYHRLRDFIL

>C.ZA.09.CM019P_SGA02.MF284922

SYHRLRDFIL

>C.ZA.09.CM021P_SGA22.MF284952

CYHRLRDLIL

>C.ZA.09.CM029C_SGA07.MF284958

SYHQLRDFIL

>C.ZA.09.CM041P_SGA03.MF284962

SYHHLRDFIL

>C.ZA.09.CM050C_SGA05.MF284967

SYHRLRDLIL

>C.ZA.09.CM052C_SGA07B.MF284977

SYHHLRDFIL

>C.ZA.09.CM089P_SGA05.MF285011

SYRRLRDFTL

>C.ZA.09.CM112C_SGA04.MF285022

SYHQLRDFIL

>C.ZA.09.CM132P_SGA04.MF285058

SYHQLRDFVL

>C.ZA.09.DEMC09ZA008.JX140667

SYHHLRDFIL

>C.ZA.09.DEMC09ZA009.JX140668

SYHLLRDFIL

>C.ZA.09.Ko243_H6.3.KF114892

LYHQLRDFTL

>C.ZA.09.MSM001.KF725883

SYHQLRTFIL

>C.ZA.09.MSM002.KF725884

SYHRLRDLLL

>C.ZA.09.MSM003.KF725885

SYHRLRDFIL

>C.ZA.09.MSM011.KF725889

SYHQLRDFIL

>C.ZA.09.MSM012.KF725890

SYHRLRDFIL

>C.ZA.09.MSM014.KF725891

SYHLLRDFIL

>C.ZA.09.MSM015.KF725892

SYHRLRDFIL

>C.ZA.09.MSM025.KF725895

CYHRLRDFIL

>C.ZA.09.MSM029.KF725896

SYHHLRDFIL

>C.ZA.09.MSM034.KF725897

SYHRLRDFIL

>C.ZA.09.MSM041.KF725900

SYHQLRNFIL

>C.ZA.09.MSM052.KF725903

SYHQLRDFIL

>C.ZA.09.MSM055.KF725905

IYHLLRNFIL

>C.ZA.09.MSM061.KF725908

SYHLLRDFIL

>C.ZA.09.MSM065.KF725912

SYHQLRDFIL

>C.ZA.09.MSM068.KF725914

SYHHLRDFIL

>C.ZA.09.MSM075.KF725916

SYHRLRDFIL

>C.ZA.09.MSM077.KF725917

SYHRLRDFIL

>C.ZA.09.MSM084.KF725920

SYHQLRDFIL

>C.ZA.09.MSM086.KF725921

SYHRLRDFIL

>C.ZA.09.MSM102.KF725922

SYHRLRNFIL

>C.ZA.09.TRP343_2_00_21_2.JN681257

SYHQLTTFIL

>C.ZA.09.TRP347_2_00_B1_1.JN681258

SYHRLRDLIL

>C.ZA.09.TRP363_2_00_10_3.JN681259

SYHQLRDFIL

>C.ZA.10.CAP357_3101_034wpi_MN0005.MK205753

CYHRLRDFIL

>C.ZA.10.CAP378.2.00_D2.5.KF114888

SYHQLRDFIL

>C.ZA.10.CAP382_2_00_D7_19.KC154028

CYHRLRDFIL

>C.ZA.10.DEMC10ZA001.JX140669

SYHHLRDFIL

>C.ZA.10.MSM105.KF725924

SYHQLRDFIL

>C.ZA.10.MSM114.KF725926

SYHRLRDFIL

>C.ZA.10.MSM115.KF725927

SYHRLRDFLL

>C.ZA.10.MSM118.KF725928

SYHRLRDFIL

>C.ZA.10.MSM126.KF725929

SYHRLRDFIL

>C.ZA.10.MSM129.KF725930

SYHRLRDFIL

>C.ZA.10.MSM131.KF725931

SYHQLRDFIL

>C.ZA.10.MSM138.KF725933

SYHRLRDFIL

>C.ZA.10.MSM146.KF725934

SYHQLRNFIL

>C.ZA.10.MSM150.KF725935

SYHQLRDFIL

>C.ZA.10.MSM151.KF725936

CYHRLRDFTL

>C.ZA.10.MSM161.KF725937

CYHRLRDLIL

>C.ZA.10.MSM162.KF725938

CYHRLRDFIL

>C.ZA.10.MSM168.KF725942

SYHRLRDFIS

>C.ZA.10.MSM179.KF725944

SYHQLRNCIL

>C.ZA.10.MSM181.KF725945

SYHRLKDFIS

>C.ZA.10.MSM190.KF725948

SYHRLRDFIL

>C.ZA.10.MSM202.KF725949

SYHRLRDFIL

>C.ZA.10.MSM211.KF725953

CYHQLRDFVL

>C.ZA.10.MSM212.KF725954

SYHQLRDFIL

>C.ZA.10.MSM213.KF725955

CYHRLRDFIL

>C.ZA.10.MSM217.KF725956

SYHQLRDFIL

>C.ZA.10.MSM218.KF725957

SYHRLRDFIL

>C.ZA.10.MSM219.KF725958

SYHRLRDFIL

>C.ZA.10.MSM223.KF725959

SYHLLRDFIL

>C.ZA.10.MSM224.KF725960

SYHRLRDLLL

>C.ZA.10.MSM226.KF725961

CYHRLRDFIL

>C.ZA.10.MSM227.KF725962

SYHRLRDFIL

>C.ZA.10.MSM231.KF725964

IYHQLRDFIL

>C.ZA.10.MSM239.KF725968

SYHRLRDFIL

>C.ZA.10.MSM240.KF725969

SYRLLRDFIL

>C.ZA.10.MSM241.KF725970

SYHQLRTFIL

>C.ZA.10.MSM242.KF725971

SYHRLRDFIL

>C.ZA.10.MSM243.KF725972

SYHQLRDFIL

>C.ZA.10.MSM246.KF725974

SYHRLRDFIL

>C.ZA.10.MSM247.KF725975

CYHQLRDFIL

>C.ZA.10.MSM248.KF725976

SYHQLRDFIL

>C.ZA.10.MSM253.KF725979

SYHRLRDFIL

>C.ZA.10.MSM254.KF725980

SYHRLRDFLL

>C.ZA.10.MSM257.KF725983

SYHQLRDFIL

>C.ZA.10.MSM261.KF725984

SYHLLRDFIL

>C.ZA.10.MSM264.KF725987

FYHRLRDLLL

>C.ZA.10.MSM267.KF725989

SYHRLRDCIL

>C.ZA.10.MSM268.KF725990

SYHRLRDFIL

>C.ZA.10.MSM274.KF725994

SYHRLRDFIL

>C.ZA.10.MSM275.KF725995

SYHRLRDFIL

>C.ZA.10.MSM277.KF725996

CYHRLRDLIL

>C.ZA.10.MSM279.KF725997

SYHRLRDFIL

>C.ZA.10.MSM280.KF725998

SYHRLRDFIL

>C.ZA.10.MSM281.KF725999

SYHRLRDFIL

>C.ZA.10.MSM282.KF726000

SYHRLRDFIL

>C.ZA.10.MSM283.KF726001

CYHQLRDFIL

>C.ZA.10.MSM297.KF726010

LYHLLRDFIL

>C.ZA.10.MSM298.KF726011

SYHLLRDLIL

>C.ZA.10.MSM301.KF726014

SYHQLRDFIL

>C.ZA.10.MSM303.KF726015

SYHRLRDFLL

>C.ZA.10.MSM309.KF726020

SYHQLRDFIL

>C.ZA.10.MSM311.KF726022

SYHRLRDFIL

>C.ZA.10.MSM321.KF726025

SYHRLRDFIL

>C.ZA.11.CH0152_3_d1360_ipe026_30_34.MG901120

SYHRLRDLIL

>C.ZA.12.DEMC12ZA087.KP109516

SYHRLRDFIL

>C.ZA.12.DEMC12ZA096.KP109517

SYHRLRDFIL

>C.ZA.13.DEMC13ZA146.KU749415

SYHRLRDFIL

>C.ZA.13.DEMC13ZA149.KU749416

SYHRLKDFIL

>C.ZA.13.DEMC13ZA152.KU749417

SYHQLRDFIL

>C.ZA.13.HIV1_FR004_D11_CVL_1.MH933705

SYHQLRTFIL

>C.ZA.13.HIV1_FR006_D1_CVL_1.MH933704

SYHQLRDFIL

>C.ZA.14.HIV1_FR014_D7_plasma_1.MH933706

LYHRLRDFIL

>C.ZA.89.pZAC_R3714.JN188292

SYHRLRDFIL

>C.ZA.97.97ZA003.AY118165

SYHQLRDFIL

>C.ZA.97.97ZA009.AY118166

SYHRLRDFIL

>C.ZA.97.97ZA012.AF286227

IYHQLRDFIL

>C.ZA.98.98ZA445.AY158533

SYHRLRDFIL

>C.ZA.98.98ZA502.AY158534

SYHRLRDLIL

>C.ZA.98.98ZA528.AY158535

SYHRLRDFIL

>C.ZA.98.98ZADu104.AY529659

SYHRLRDFLL

>C.ZA.98.CTSC2.AY043176

SYHRLRDFIL

>C.ZA.98.Du123_6.DQ411850

SYHRLRDFIL

>C.ZA.98.Du172_17.DQ411853

SYHRLRDLIL

>C.ZA.98.Du21.AY529661

SYHQLRDFIL

>C.ZA.98.Du23.AY529662

FYHQLRDFIL

>C.ZA.98.TV001.AY162223

SYHRLRDFIL

>C.ZA.98.TV002.AY162224

CYHRLRDFIL

>C.ZA.98.TV004.AF391234

SYHRLRDFIL

>C.ZA.98.TV006A.AF391235

SYHRLRDFIL

>C.ZA.98.TV007A.AF391238

SYHRLRDLIL

>C.ZA.98.TV008A.AF391240

SYHLLRDFIL

>C.ZA.98.TV010.AF391242

CYHRLRDFIL

>C.ZA.98.TV012.AY162225

LYHRLRDFIL

>C.ZA.98.TV013B.AF391246

SYHQLRDFIL

>C.ZA.98.TV014A.AF391247

SYHRLRDFIL

>C.ZA.98.TV018.AF391249

SYHQLRDLIL

>C.ZA.98.TV019.AF391250

SYHQLRDFIL

>C.ZA.99.98ZADu281.AY529664

SYHHLRDFIL

>C.ZA.99.98ZADu301.AY529665

SYHRLRDFIL

>C.ZA.99.99ZACM9.AF411967

SYHRLRDFIL

>C.ZA.99.99ZALT46.EU293450

SYHRLRDLTL

>C.ZA.99.99ZALT4.EU293444

SYHRLRDCTL

>C.ZA.99.99ZASW12p.AY529668

SYHRLRDFIL

>C.ZA.99.99ZASW20.AY529669

SYHRLRDFIL

>C.ZA.99.99ZASW27.AY529670

CYHRLRDFIL

>C.ZA.99.99ZASW30_b.AY529673

SYHRLRDFIL

>C.ZA.99.99ZASW9.AY529675

LYHQLRDFIL

>C.ZA.99.99ZATM10.AY228556

SYHRLRDFIL

>C.ZA.99.99ZATM12.AY529676

SYHQLRDFIL

>C.ZA.99.99ZATM1b.AY529677

SYHRLRDFIL

>C.ZA.99.99ZATM2.AY529678

SYHQLRNFIL

>C.ZA.99.COT6.DQ447266

SYHRLRDFIL

>C.ZA.99.COT9.DQ447272

SYHRLRDFIL

>C.ZA.99.DU179.AY043174

SYHRLRDFIL

>C.ZA.99.DU422.AY043175

SYHQLRDFIL

>C.ZA.99.Du156_12.DQ411852

SYHQLRDFIL

>C.ZA.99.LT10.AY522722

SYHRLRDFIL

>C.ZA.99.LT15.AY522723

SYHRLRDFIL

>C.ZA.99.LT17.AY522724

SYHLLRDFIL

>C.ZA.99.LT18.AY522725

SYHQLRDFIS

>C.ZA.99.LT1.AY522721

SYHRLRDFIL

>C.ZA.99.LT21.AY522726

SYHRLRDFIL

>C.ZA.99.LT25.AY522727

SYHRLRDFIL

>C.ZA.99.LT28.AY522728

SYHRLRDLIL

>C.ZA.99.LT36.AY522729

SYHRLRDFIL

>C.ZA.99.LT38.AY522730

SYHQLRDLLL

>C.ZA.99.LT39.AY522731

SYHHLRDFIL

>C.ZA.99.LT40.AY522732

SYHRLRDFLL

>C.ZA.99.LT42.AY522733

SYHHLRDFIL

>C.ZA.99.LT45.AY522734

SYHRLRDFIL

>C.ZA.99.LT50.AY522736

SYRRLRDLIL

>C.ZA.99.LT5.AY522735

SYRRLRDCIL

>C.ZA.99.TM3.DQ447268

SYHRLRDFVL

>C.ZA.99.TM7.DQ447267

SYHRLRDFIL

>C.ZA.99.ZA8119636.KU168308

IYHQLRDFIL

>C.ZA.99.ZASW7.AF411966

SYHRLRDFIL

>C.ZA.x.035b9d6.GU216739

SYHRLRDCIL

>C.ZA.x.221b4a6.GU216805

SYHQLRNFIL

>C.ZA.x.312b8e6.GU216844

SYHRLRDFIL

>C.ZA.x.SA_C101_D2.HQ625580

SYHRLRDFIL

>C.ZA.x.SA_C10_D4.HQ625593

CYHQLRDFIL

>C.ZA.x.SA_C21_A2.JX905394

SYHRLRDFIL

>C.ZA.x.SA_C23_B5.HQ625591

SYHQLRDFIL

>C.ZA.x.SA_C26_F5.HQ625570

SYHRLRDFIL

>C.ZA.x.SA_C2.HQ625595

SYHRLRDFLL

>C.ZA.x.SA_C32_D9.HQ625590

SYRRLRDFIL

>C.ZA.x.SA_C36_C1.HQ625589

SYHRLRDCAL

>C.ZA.x.SA_C37_B4.HQ625572

SYHRLRDFLL

>C.ZA.x.SA_C3_B3.HQ625566

SYHQLRDFIL

>C.ZA.x.SA_C48_E1.HQ625573

SYHRLRDFIL

>C.ZA.x.SA_C61_B6.HQ625587

SYHLLRDFIL

>C.ZA.x.SA_C62_C3.HQ625574

SYHQLRTFIL

>C.ZA.x.SA_C65_G10.HQ625586

SYHQLRDFIL

>C.ZA.x.SA_C67_D7.HQ625575

SYRLLRDFIL

>C.ZA.x.SA_C72_F9.HQ625585

SYHRLRDFIL

>C.ZA.x.SA_C74_D12.HQ625576

SYHHLRDFIL

>C.ZA.x.SA_C75_A2.HQ625584

SYHQLRDFIL

>C.ZA.x.SA_C76_B10.HQ625577

CYRQLRNFIF

>C.ZA.x.SA_C80.HQ625565

SYHLLRDFIL

>C.ZA.x.SA_C82_G11.HQ625583

CYHRLRDFTL

>C.ZA.x.SA_C86_A9.HQ625578

SYHRLRDFLL

>C.ZA.x.SA_C87_A10.HQ625582

IYHRLRDFIL

>C.ZA.x.SA_C8_A6.HQ625594

SYHRLRDCIL

>C.ZA.x.SA_C90_E5.HQ625579

SYHRLRDCIL

>C.ZA.x.SA_C91_H4.HQ625581

SYHRLRDFIL

>C.ZA.x.SA_C9_G4.HQ625567

SYHLLRDFIL

>C.ZA.x.pCeCAP188_1_D1_14m.KC894134

SYHQLRNFIL

>C.ZA.x.sa_c12_d7.HQ625568

SYHHLRDFIL

>C.ZA.x.sa_c18_g3.HQ625592

CYRRLRDFIS

>C.ZA.x.sa_c34_d12.HQ625571

SYHQLRDFIL

>C.ZA.x.sa_c44_e5.HQ625588

CYHRLRDFIL

>C.ZM.01.16B_PB_305.GU939049

CYHQLRDFIL

>C.ZM.01.16M_PB_653.GU939100

CYHQLRNFIL

>C.ZM.01.1M_PL_2006.HM036825

SYHRLRDFIL

>C.ZM.01.22B.PB.801.KY229671

SYHQLRNFIL

>C.ZM.01.4B.PB.203.KY229391

SYHRLRDFIL

>C.ZM.02.02ZM110.AB254142

SYHRLRDLIL

>C.ZM.02.02ZM114.AB254146

SYHRLRDFIL

>C.ZM.02.02ZM115.AB254148

SYHRLRDFIL

>C.ZM.02.02ZMBC.AB254149

SYHRLRDFIL

>C.ZM.02.02ZMDB.AB254153

SYHRLRDFIL

>C.ZM.02.02ZMJM.AB254156

SYHRLRDFIL

>C.ZM.02.13B.PB.103.KY229251

SYHRLKDFIL

>C.ZM.02.14M_BML_1012.HM036760

SYHRLTDFIL

>C.ZM.02.18B.PL.2d.KY229291

SYHQLRDFIL

>C.ZM.02.19B.PL.11.KY229414

SYHRLRDFIL

>C.ZM.02.20B.PB.1.KY229510

SYHRLRDLIL

>C.ZM.02.21M_BML_1012.GU939143

SYHRLRDFIL

>C.ZM.02.22M.PB.7801.KY229678

SYHQLRNFIL

>C.ZM.02.2B.PB.101.KY229337

SYHRLRDFIL

>C.ZM.02.2M.PB.2804.KY229352

SYHRLRDFIL

>C.ZM.02.32M_BML_1032.HM036864

SYHRLRDLLL

>C.ZM.02.3B.PB.4.KY229356

SYHRLRDLIL

>C.ZM.02.3M.PB.351.KY229374

SYHRLRDFIL

>C.ZM.02.5B.PB.408.KY229434

SYHRLRDFTL

>C.ZM.02.5M.PB.504.KY229453

SYRRLRDFTL

>C.ZM.02.6B.PL.102.KY229460

SYHRLRDFIL

>C.ZM.02.6M.PL.108.KY229472

SYHRLRDFIL

>C.ZM.02.7M_BML_3302A1.HM037006

SYHRLRDSIL

>C.ZM.02.ZM180M_SGA_A5.EU166385

FYHRLRDLIL

>C.ZM.02.ZM197M.DQ388515

SYHRLRDFIL

>C.ZM.02.ZM206F_SGA_A5.EU166449

SYHHLRDFIL

>C.ZM.02.ZM211M_5JUL02_PB6.JX239298

SYHRLRDFIL

>C.ZM.02.ZM215F.DQ422948

SYHRLRDFML

>C.ZM.02.ZM229M_SGA_D17.EU166594

SYHRLRDFIL

>C.ZM.02.ZM233M.DQ388517

SYHLLRDFIL

>C.ZM.02.ZM235F_SGA_A1.EU166653

SYHQLRDFIL

>C.ZM.03.31M_BML_203.HM036843

SYHLLRDFIL

>C.ZM.03.33M_BML_110.HM036903

SYHRLRDFIL

>C.ZM.03.9B.PB.202.KY229542

SYHRLRDFIL

>C.ZM.03.Z185FPL31JUL03ENV1.1.GQ485357

SYHQLRDFIL

>C.ZM.03.Z221MPL7MAR03ENV2_1.HM068598

SYHLLRDFIL

>C.ZM.03.ZM178F_SGA_B14.EU166359

IYHRLRDFTL

>C.ZM.03.ZM184F_SGA_TA16.EU166432

SYHRLRDFIL

>C.ZM.03.ZM214M.DQ388516

SYHHLRDLIL

>C.ZM.03.ZM237M_29JUL03_PL016.JX213387

SYHRLRDFIL

>C.ZM.03.ZM246FM_BULK_60.EU166715

CYRQLRDLLL

>C.ZM.03.ZM246F_flD5.FJ496194

CYRQLRDLLL

>C.ZM.03.ZM246M_040403_SGA_T4.EU166769

SYRHLRDFIL

>C.ZM.03.ZM247F_flH1.FJ496207

SYHRLRDFIL

>C.ZM.03.ZM247M_1NOV03_PB3.JX239338

SYHLLRDSIL

>C.ZM.03.ZM249M_flF1.FJ496214

FYHRLRDFIL

>C.ZM.04.10B.PB.102.KY229617

CYHRLRDFIL

>C.ZM.04.10M_BML_1801.HM036739

CYHRLRDFIL

>C.ZM.04.11B.PL.1009.KY229633

SYHRLRDFIL

>C.ZM.04.11M.PL.803.KY229641

CYHRLRDFIL

>C.ZM.04.12B_PL_201.GU939124

SYHRLRDFIL

>C.ZM.04.15B.PB.509.KY229379

SYHRLRDCIL

>C.ZM.04.17M_BML_11603.HM036792

SYHRLRDFIL

>C.ZM.04.23B.PB.3148.KY229650

SYRRLRDLIL

>C.ZM.04.Z205FPB5NOV04ENV5.2.GQ485436

SYHHLRDFIL

>C.ZM.04.ZM267F_29JUN04_PL24B.JX213407

SYHRLRDFIL

>C.ZM.05.ZM211F_22JUN05_PB3.JX239272

CYHRLTDFIL

>C.ZM.05.ZM282F_3MAR05_PB2.JX239362

SYHRLRDFIL

>C.ZM.05.ZM284M_9APR05_PL001.JX213435

SYHRLRDFIL

>C.ZM.05.ZM289M_19MAY05_PL010.JX213443

CYHRLRDFIL

>C.ZM.05.ZM297M_9JUN05_PL03B.JX213454

SYHRLRDFIL

>C.ZM.05.ZM373_200373_4.GU329051

SYHRLRDFIL

>C.ZM.05.ZM375_200375_6.GU329057

SYHRLRDFIL

>C.ZM.05.ZM376_200376_8.GU329069

SYHRLRDCIL

>C.ZM.05.ZM377_200377_3.GU329080

LYHRLRDFIL

>C.ZM.05.ZM378_200378_20.GU329105

CYHRLRDLIL

>C.ZM.05.ZM379_200379_12.GU329116

CYHRLRDSIL

>C.ZM.05.ZM381_200381_2.GU329131

SYHRLRDFIL

>C.ZM.05.ZM382_200382_2.GU329145

SYHRLRDLIL

>C.ZM.05.ZM383_200383_9.GU329161

SYHRLRDLVL

>C.ZM.05.ZM388_200388_2.GU329185

SYHRLRDFTL

>C.ZM.05.ZM389_200389_2.GU329195

SYHRLRDFIL

>C.ZM.05.ZM393_200393_1.GU329205

SCHRLRDFIL

>C.ZM.05.ZM394_200394_7.GU329220

SYHRLRDFIL

>C.ZM.05.ZM395_200395_8.GU329240

SYHRLRDFIL

>C.ZM.05.ZM399_200399_24.GU329269

SYHQLRDFIL

>C.ZM.05.ZM400_200400_13.GU329282

SYHRLRDFIS

>C.ZM.05.ZM401_200401_2.GU329289

SYHRLRDFIL

>C.ZM.05.ZM402_200402_4.GU329303

SYHRLRDFIL

>C.ZM.05.ZM403_200403_16.GU329320

SYHRLRDFIL

>C.ZM.05.ZM405_200405_40.GU329324

SYHRLRDCIL

>C.ZM.05.ZM406_200406_26.GU329333

SYHRLRDFIL

>C.ZM.05.ZM408_200408_32.GU329359

LYHRLTDFIL

>C.ZM.05.ZM410_200410_50.GU329368

SYHRLRDFIL

>C.ZM.05.ZM411_200411_13.GU329387

SYHRLRDFIL

>C.ZM.05.ZM412_200412_7.GU329394

LYRLLRDFIL

>C.ZM.05.ZM413_200413_16.GU329411

SYHRLRDFIL

>C.ZM.05.ZM414_200414_24.GU329428

SYHRLRDFIL

>C.ZM.05.ZM415_200415_4.GU329439

LYHRLRDFTL

>C.ZM.05.ZM416_200416_20.GU329473

SYHRLRDFLL

>C.ZM.05.ZM417_200417_12.GU329489

SYHQLRDFTL

>C.ZM.05.ZM418_200418_9.GU329494

SYHRLRDFIL

>C.ZM.05.ZM419_200419_4.GU329501

CYHRLRDFIL

>C.ZM.05.ZM420_200420_3.GU329512

CYHRLRDFIL

>C.ZM.06.Z1024F_2Dec06_3A4.KX983797

SYHRLRDFIL

>C.ZM.06.ZM1072M_16AUG06_PL015.JX213352

SYHRLRDLLL

>C.ZM.06.ZM282M_15AUG06_PB1.JX239395

IYHRLRDFIL

>C.ZM.07.235080.JN977604

SYHRLRDFVL

>C.ZM.07.DEMC07ZM013.KU749425

SYHRLRDFIL

>C.ZM.07.Donor_584.KT252545

SYRQLRDFIL

>C.ZM.07.Z1022M_14DEC07_D23.KX983735

LYHRLRDFIL

>C.ZM.07.Z1023M_23MAY07_A12.KX983777

SYHRLRDFTL

>C.ZM.07.Z1047M_24AUG07_B17.KX983831

SYHRLRDCIL

>C.ZM.07.Z1800M_21JUL07_E16.KX983891

FYHRLRDFIL

>C.ZM.07.ZM1464M_10MAR07_PL170.JX213359

SYHRLRDFIL

>C.ZM.07.ZM503F_9FEB07_PL100.JX213464

SYHQLRDFIL

>C.ZM.08.Z1781M_12FEB08_C1.KX983851

LYHRLTDFIL

>C.ZM.09.DEMC09ZM004.KF716466

CYHRLRDLIL

>C.ZM.09.DEMC09ZM015.KU749426

SYHRLRDFIL

>C.ZM.09.Z331F_15Apr09_PL_NFLG_SGA22.KR820304

SYHQLRDFIL

>C.ZM.09.Z331M_18Apr09_PL_NFLG_SGA10.KR820314

SYHQLRNFIL

>C.ZM.09.Z3576F_28Mar09_PL_NFLG_SGA1.KR820324

SYHRLRDFVL

>C.ZM.09.Z3576M_18Apr09_PL_NFLG_SGA10.KR820326

SYHRLRDFVL

>C.ZM.09.Z3618F_11Jul09_PL_NFLG_SGA11.KR820342

CYHRLRDFIL

>C.ZM.09.Z3678F_23Sep09_PL_NFLG_SGA19.KR820376

LYHRLRDSIL

>C.ZM.10.Z4248F_11Jun10_PL_NFLG_SGA1.KR820394

SYHRLRDFIL

>C.ZM.10.Z4248M_4Jun10_PL_NFLG_SGA1.KR820415

SYHRLRDFIL

>C.ZM.11.DEMC11ZM003.KP109494

SYHRLRDFIL

>C.ZM.11.DEMC11ZM005.KP109495

LYHRLRDLGL

>C.ZM.11.DEMC11ZM006.KF716467

SYHHLRDFIL

>C.ZM.11.DEMC11ZM008.KP109496

SYHRLRDLIL

>C.ZM.89.ZAM18.AB485647

SYHRLRDFIL

>C.ZM.96.96ZM651.AF286224

SYHRLRDFIL

>C.ZM.96.96ZM751.AF286225

SYHRLRDFIL

>C.ZM.x.HIV1084i.AY805330

SYHRLRDCIL

>C.ZM.x.SE12808.AY494971

SYHRLRDFIL

>C.ZM.x.m44_030.KU200938

SYHRLRDFIL

>C.ZW.09.C535_p2.JF680908

CYHRLRDFLL

>C.x.03.LA07SeJe.KU168262

SYHRLRDLLL

>C.x.09.J520_p2.JF680918

SYRQLRDFIL

>C.x.x.pCe3045_2D5.KC894102

SYHQLRDFIL

>D.BE.93.VI824.HQ912709

SYHRLRDLIL

>D.BR.10.10BR_RJ095.KJ787684

SYHRLRDLLL

>D.BR.10.10BR_RJ108.KJ787683

SYRRLSDLLS

>D.BR.96.patient_96BRRJ100.DQ141204

SYHRLRDLLL

>D.CA.05.091_WK48.GU191441

SYHRLRDLVL

>D.CD.02.CG_0382_02V_NGSID3.KY392769

SYHRLRDLLL

>D.CD.02.LA18ZiAn.KU168272

IYRRLRDLLL

>D.CD.03.LA17MuBo.KU168271

SYRRLRDLVL

>D.CD.84.84ZR085.U88822

SYHRLRELIL

>D.CD.85.Z2Z6_Z2_CDC_Z34.M22639

SYHRLRDLIL

>D.CD.87.PBS5635.MH705152

SYHRLRDLLL

>D.CD.x.JY1_Z84.J03653

SYHRLRDLIL

>D.CG.07.P940_2_C3.HM068555

SYHRLRDLLL

>D.CI.90.CI13.AJ277820

SYHRLRDLLL

>D.CM.01.01CM_0009BBY.AY371155

SYHRLRDLIL

>D.CM.01.01CM_0175BA.AY371156

SYHRLRDLIL

>D.CM.01.01CM_4412HAL.AY371157

SYHRLRDLIL

>D.CM.10.DEMD10CM009.JX140670

SYHRLRDLIL

>D.CY.06.CY163.FJ388945

SYHRLRDLIL

>D.ES.07.P1741_a2.HM068556

SYHRLRDLLL

>D.ES.09.P2351.JN054274

SYHRLRDLLL

>D.FI.93.FIN93167.AF219271

SYHRLRDLIL

>D.FI.93.FIN93178.AF219272

SYHRLRDLLL

>D.FR.x.DGOB.AF321082

SYHRLRDLIL

>D.GB.08.N535_F5.HQ595791

SYHRLRDLIL

>D.GB.13.15228_1_51.3.MF109689

SYHRLRDLLL

>D.GB.13.15228_1_72.3.MF109710

SYHRLSDLLL

>D.GB.13.15228_1_75.4.MF109713

SYHRLTDLLL

>D.GB.14.14535_1_3.3.MF109532

LYHRLRDLLL

>D.GM.94.N73603.HQ385448

SYHHLRDLIL

>D.KE.01.NKU3006.AF457090

SYHRLRDLIL

>D.KE.11.385_KE.KU921767

SYHRLRDLLL

>D.KE.11.DEMD11KE003.KF716476

SYRHLRDLLL

>D.KE.93.QA465_59M_ENV_D1.FJ866137

SYRHLRDLIL

>D.KE.95.QA013_70I_ENV_M12.FJ866135

SYHRLRDLIL

>D.KE.97.ML415_2.AY322189

SYHRLRDFLL

>D.KE.97.QB857_110I_ENV_B3.FJ866138

SYHQLRNLLL

>D.KE.99.QD435_100M_ENV_E1.FJ866141

SYHRLRDLIL

>D.KR.91.91RDH12_15991.MH425161

SYHRLRDLLL

>D.SE.12.077UG.MF373180

SYHRLRDLIL

>D.SE.99.P4b_19990121.GU204938

SYRHLRDLIL

>D.SN.90.SE365A2.L22945

SYHRLRDLIL

>D.TD.99.MN011.AJ488926

SYHQLRDLIL

>D.TZ.00.54_F4_A10.HQ659623

SYHRLRDLTL

>D.TZ.01.A280.AY253311

SYRHLRDLIL

>D.TZ.04.CO6405V4.KX907406

SYHRLRDLIL

>D.TZ.08.707010549_A1.HQ615975

SYHHLRDLIL

>D.TZ.87.87TZ4622.U65075

SYHRLRDLIL

>D.UG.01.231965_c01.JQ361079

IYHRLRDLIL

>D.UG.01.231966_c02.JX512899

SYHRLRDLLL

>D.UG.01.605MPC9.EU853116

SYHRLRDLVL

>D.UG.05.2769MP10.KF986106

SYHRLRDLLL

>D.UG.05.2810MP1.KF986131

SYHHLRDLLL

>D.UG.05.394MP1.KF986058

SYHRLRDLIL

>D.UG.05.888MP1.KF986033

SYHRLRDLTL

>D.UG.05.D053826R11_1.JX658588

SYHRLSDLLL

>D.UG.05.p190049.JX236668

SYHRLRDLVL

>D.UG.06.191821_E6_1.HM215270

SYHRLRDLIL

>D.UG.06.890MP1.KF986074

SYHRLTDLLL

>D.UG.06.927MP1.KF986041

SYHRLRDLIL

>D.UG.07.9009SA_A4_2.HM215351

SYHRLRDLIF

>D.UG.07.p191647.JX236670

SYHRLRDLIL

>D.UG.07.p191882.JX236673

SYHRLRDLLL

>D.UG.07.pSC191727.JX236679

SYHHLRSLIL

>D.UG.08.p191859.JX236672

SYHHLRDLIL

>D.UG.10.DEMD10UG004.KF716479

SYHRLRDLIL

>D.UG.11.DEMD11UG003.KF716480

SYHRLRDLLL

>D.UG.90.UG269A.L22949

SYHRLRDLIL

>D.UG.90.UG274A2.L22950

SYRRLRDLLL

>D.UG.91.UG270.AB485651

SYHRLRDLIS

>D.UG.92.92UG001.AJ320484

SYHRLRDLIL

>D.UG.92.92UG024_D.U08805

RYHHLRDLIL

>D.UG.92.CD4_dependent_92UG046.AY623599

SYRHLRDLIL

>D.UG.93.93UG_065.AY713418

SYHRLRDLLL

>D.UG.94.94UG114.U88824

SYHRLRDLIL

>D.UG.95.42_877.MH705143

SYHRLRDLIL

>D.UG.96.32MPC1.EU852998

SYHRLRDLIL

>D.UG.96.A03836B1_6.JX658585

SYHRLRDLLL

>D.UG.96.HFFP1.KF986082

SYHRLRDLIL

>D.UG.97.108FPc01.EU852934

SYHRLRDLLL

>D.UG.97.183MPc02.EU852966

SYHRLRDLVS

>D.UG.97.295FP01.EU852982

SYHRLRDLIL

>D.UG.97.326MPc07.EU853014

SYHRLRDLLL

>D.UG.97.338MPc01.EU853046

SYHRLRDLIL

>D.UG.97.pt197.EU281998

SYHRLRDLIL

>D.UG.97.pt632.EU281996

SYHRLRDLLL

>D.UG.97.pt827.EU281995

SYHRLRDXXL

>D.UG.98.372FPc02.EU853078

SYHRLRDLIL

>D.UG.98.602MPC2.EU853094

SYHRLRDLLL

>D.UG.98.98UG57128.AF484502

SYHRLTDLIL

>D.UG.98.98UG57131.AF484505

SYHRLRDLIL

>D.UG.98.98UG57132.AF484506

SYHHLRNFLL

>D.UG.98.98UG57140.AF484511

SYHRLRDLIL

>D.UG.98.98UG57143.AF484514

SYHRLRDLIL

>D.UG.98.98UG57146.AF484513

SYHRLRDLLL

>D.UG.98.99UGJ32228.AF484516

SYHRLRDLIL

>D.UG.99.99UGA03349.AF484518

CYHRLRDLIL

>D.UG.99.99UGA07412.AF484477

SYHRLRDLIL

>D.UG.99.99UGA08483.AY304496

SYHRLRDLLL

>D.UG.99.99UGB21875.AF484480

SYHRLRDLIL

>D.UG.99.99UGB25647.AF484481

SYHRLRDLLL

>D.UG.99.99UGB32394.AF484483

SYRHLRDLIL

>D.UG.99.99UGD23550.AF484485

SYHRLRDLIL

>D.UG.99.99UGD26830.AF484486

SYHRLRDLLL

>D.UG.99.99UGE08364.AF484487

SYHRLRDLLL

>D.UG.99.99UGE13613.AF484515

SYHHLRDLVL

>D.UG.99.99UGE23438.AF484489

SYHRLRDLLL

>D.UG.99.99UGF03726.AF484519

SYRHLRDLLL

>D.UG.99.99UGF05734.AF484490

SYHHLRDLVL

>D.UG.99.99UGG10555.AF484494

SYHRLRDLIL

>D.UG.99.99UGG35093.AF484495

CYHRLRDLVL

>D.UG.99.99UGJ27597.AF484497

SYHRLRDLIL

>D.UG.99.99UGK09259.AF484498

SYHRLRDLLL

>D.UG.99.99UGK09958.AF484499

SYHQLRNLIS

>D.UG.x.C971_412.U36871

GYRHLRDLLL

>D.UG.x.DM1_40.EF575364

SYHHLRDLIL

>D.UG.x.DM2_12.EF575370

SYHRLRDLIL

>D.UG.x.DM3_21.EF575388

SYHRLRDLLL

>D.UG.x.DM4_032.EF575400

SYHRLRDLLL

>D.UG.x.DM5_17.EF575417

SYHRLRDLIL

>D.UG.x.DM6_11.EF575429

IYHRLRDSLL

>D.UG.x.DM7_27.EF575447

SYHHLRDLIL

>D.UG.x.DM9_29.EF575482

SYHRLRDLLL

>D.UG.x.WHO15_474.U36886

SYHHLRDLIL

>D.US.07.HIV_US_BID_V3121_2007.JQ403079

SYHRLRDLLL

>D.YE.01.01YE386.AY795903

SYHRLRDLIL

>D.YE.02.02YE516.AY795907

SYHQLRDLIL

>D.ZA.84.R2.AY773338

SYHRFRDLLL

>D.ZA.85.R214.AY773339

SYHRSRDLLF

>D.ZA.85.R286.AY773340

SYHRLRDLLL

>D.ZA.86.R482.AY773341

GYHRSRDLLL

>D.ZA.90.R1.EF633445

SYRRLRDLLL

>D.ZM.05.ZM387_200387_5.GU329179

SYHRLRDLLL

>D.x.09.N320b_p1.JF680926

SYHRLRDLIL

>F1.AO.06.AO_06_ANG32.FJ900266

SYRHLRDFIL

>F1.AO.06.AO_06_ANG40.FJ900267

SYRHLRDFIL

>F1.AO.06.AO_06_ANG58.FJ900268

SYRHLRDFIL

>F1.AR.02.ARE933.DQ189088

SYRHLRDFIL

>F1.BE.93.VI850.AF077336

SYRHLRDFIL

>F1.BE.94.14_00_37.DQ313239

SYRRLRDFIL

>F1.BR.01.01BRRJSB153.MG365763

SYRHLRDFIL

>F1.BR.02.02BR082.FJ771006

SYRHLRDFIL

>F1.BR.06.06BR564.FJ771008

SYRHLRDFIL

>F1.BR.06.06BR579.FJ771009

SYHHLRDLIL

>F1.BR.06.P1356_F12.HQ236573

SYRHLRDFIL

>F1.BR.07.07BR844.FJ771010

SYRHLRDFIL

>F1.BR.08.08BRRJ35.MG365767

SYRHLRDFIL

>F1.BR.10.10BR_PE107.KJ849782

SYRHLRDFIL

>F1.BR.10.10BR_RJ015.KJ849791

SYRLLRDFIL

>F1.BR.10.10BR_RJ055.KT427774

SYRHLRDFIL

>F1.BR.10.10BR_RJ084_2.KT427868

SYRHLRTFIL

>F1.BR.10.DEMF110BR015.KU749395

SYRQLRDLIL

>F1.BR.11.11BRRJPR69.MG365766

SYRHLRDFIL

>F1.BR.11.11BRRJPR90.MG365768

SYRHLRDLIL

>F1.BR.11.DEMF111BR037.KU749396

SYRHLRDFIL

>F1.BR.12.12BRRJP05.MG365762

SYRHLRDFIL

>F1.BR.12.12BRRJPR51.MG365764

SYRHLRDFIL

>F1.BR.12.2012BRRJNEUT32.KX181918

SYRHLRDFIL

>F1.BR.12.2012BRRJNEUT38.KX181921

SYRHLRDLIL

>F1.BR.12.2012BRRJNEUT39.KX181922

SYRHLRDFIL

>F1.BR.12.2012BRRJNEUT40.KX181923

SYRHLRDFIL

>F1.BR.12.2012BRRJNEUT43.KX181926

SYRHLRDFIL

>F1.BR.13.2013BRRJNEUT31.KX181917

SYRHLRDFIL

>F1.BR.13.2013BRRJNEUT35.KX181919

SYRHLRDFIL

>F1.BR.13.2013BRRJNEUT42.KX181925

SYRLLRDFIL

>F1.BR.89.BZ163.AY173958

SYHHLRDLLL

>F1.BR.93.93BR020_1.AF005494

SYRHLRDFIL

>F1.CY.08.CY222.JF683771

SYRHLRDFIL

>F1.DE.x.MVP_30846.EU446022

SYRHLRDFIL

>F1.ES.06.MFU14_2_E7.HQ236559

SYRLLRDFIL

>F1.ES.07.P1599_G11.HQ236575

SYRHLRDFIL

>F1.ES.07.P1655_F8.HQ236580

SYRHLRDFIL

>F1.ES.08.X2674_C4.HQ236616

SYRLLRDFIL

>F1.ES.09.P2165_d2_2.HM068554

SYRHLRDFIL

>F1.ES.09.X2687_f3.HM068551

SYRHLRDLIL

>F1.ES.11.DEMF110ES001.JX140671

SYRHLRDLIL

>F1.ES.11.VA0053_nfl.KJ883138

SYRHLRDLIL

>F1.ES.14.100347.MF381271

SYHLLRDFIL

>F1.ES.16.100349.MF381284

SYRHLRDFIL

>F1.ES.x.P1146.DQ979023

SCRLLRDFIL

>F1.ES.x.X1093_2.DQ979025

SYRHLRDFIL

>F1.ES.x.X1670.DQ979024

SYRQLRDFIL

>F1.FI.93.FIN9363.AF075703

SYRHLRDFIL

>F1.FR.04.LA22LeRe.KU168276

SYRHLRDFIL

>F1.FR.96.96FR_MP411.AJ249238

SYRHLRDFIL

>F1.FR.96.PHI420.AY231157

SYRHLRDFIL

>F1.FR.x.FGIL.AF321084

SYHHLRDLIL

>F1.GB.14.13659_1_45.3.MF109434

SYRHLRDFIL

>F1.RO.03.LA20DuCl.KU168274

SYRHLRDFIL

>F1.RO.96.BCI_R07.AB485659

SYRHLRDFIL

>F1.RU.04.RUSP816_B4.HQ236605

SFHLLRDFIL

>F1.x.03.LA21LeAn.KU168275

SYRHLRDFIL

>F2.CM.01.A1699.MH705144

SYRHLRDFIL

>F2.CM.02.02CM_0016BBY.AY371158

SYRHLRDFIL

>F2.CM.10.DEMF210CM001.JX140672

SYRHLRDFIL

>F2.CM.10.DEMF210CM007.JX140673

SYRHLRDFIL

>F2.CM.11.DEMF211CM025.KU749420

SYHLLRDFIL

>F2.CM.11.DEURF11CM026.KU749422

SYRHLRDFIL

>F2.CM.93.CA20.AJ277824

SYRHLRDFIL

>F2.CM.95.95CM_MP255.AJ249236

SYRHLRDLLL

>F2.CM.95.95CM_MP257.AJ249237

SYHCLRNFIL

>F2.CM.97.CM53657.AF377956

SYRHLRDFIL

>F2.ES.08.P2059_a_B3.JN054264

SYRLLRDFIL

>F2.ZA.10.MSM237.KF725967

SYRHLRDLIL

>G.BE.96.DRCBL.AF084936

SYHRLRDFIL

>G.CD.03.LA23LiEd.KU168277

SYHRLRDFTL

>G.CD.87.87_2580.MH705162

SYHRLRDFIL

>G.CD.87.P406.MH705155

SYHRLRDFIL

>G.CD.87.PBS1191.MH705134

SYHRLRDFIL

>G.CM.01.01CM_4049HAN.AY371121

SYHRLRDCVL

>G.CM.01.A1786.FJ389367

SYHHLRDFVL

>G.CM.03.CM44_10.KU168302

SYHRLRDFLL

>G.CM.04.178_15.FJ389363

SYHHLRDFIS

>G.CM.04.314_40.FJ389364

SYHRLRDFIL

>G.CM.04.515_28.FJ389365

SYHRLRDFIL

>G.CM.04.944_5.FJ389366

CYHRLRDXXL

>G.CM.05.144_26.MH705145

LYHRLTDFIL

>G.CM.06.740_14.KP718915

CYHRLRDFTL

>G.CM.07.920_49.KP718923

SYHRLRDFIL

>G.CM.07.BS03_A1_01062011.KR051438

SYHRLRDFIL

>G.CM.07.BS12_D9_01082011.KR051445

SYHRLRDFVL

>G.CM.07.BS46_A2_21032012.KR051448

CYHRLRDFIL

>G.CM.07.BS48.KR017776

GYHRLRDFIL

>G.CM.07.BS51_H2_26082011.KR051461

SYHRLRDFIS

>G.CM.08.789_10.KP718925

SYHHLRDFVL

>G.CM.09.10056_D8_21022012.KR051421

SYRHLRDFVL

>G.CM.09.11439_F5.KR051428

SYRHLRDFVL

>G.CM.10.12541_E2.KR051437

SYHRLRDFVL

>G.CM.10.DEMG10CM008.JX140676

CYHRLRDFTL

>G.CM.10.DEURF10CM020.KP109502

LYHRLRDLIL

>G.CM.11.DEMG11CM046.KY658701

SYHRLRDLVL

>G.CM.96.96CMABB55.AY772535

SYHRLRDFIL

>G.CM.97.97CM_MP801.AM279346

SYHRLRDFVL

>G.CM.98.98CM_MP1033.AM279365

SYHRLRDFVL

>G.CM.99.99CM_MP1287.AM279351

SYHRLRDFVL

>G.CM.99.99CM_MP1416.AM279359

SYHRLRDFIL

>G.CM.99.99CM_MP1417.AM279350

SYHRLRDFIL

>G.CN.06.sh52.HM067749

SYHLLRDFVL

>G.CN.08.GX_2084_08.JN106043

LYHRLRDFIL

>G.CN.13.GZ8H3748.13.MH431770

SYHRLRDFIL

>G.CN.16.224GX.KY275364

LYHRLTDFIL

>G.CU.99.Cu74.AY586547

SYHRLRDLVL

>G.CU.99.Cu85.AY586548

SYHRLRDLLL

>G.CU.99.Cu87.AY586549

SYHRLRDLLL

>G.ES.00.X558.AF423760

SYHRLRDFVL

>G.ES.02.P402_2_11.EU885759

SYHHLRDFIL

>G.ES.02.X1193_1.EU885761

SYHHLRDFIL

>G.ES.03.X1254_3.EU885762

SYHRLRDLLL

>G.ES.04.X1632_s2_b10.FJ817370

SYRRLRDFIL

>G.ES.05.P962.EU786670

SYHRLRDFIL

>G.ES.05.X1628_2.FJ670520

SYHRLRDLVL

>G.ES.05.X1854_2_10.EU885763

SYHRLRDFIL

>G.ES.06.X2088_9.EU885764

SYHRLRDFIL

>G.ES.07.MFU54_D1_3.JF327806

SYHRLRDFIS

>G.ES.07.X2160_r25.EU885765

SYHRLRDLVL

>G.ES.07.X2234_H11.HQ236608

SYHHLRDFIL

>G.ES.08.P1981_2.FJ670530

SYHRLRDFIL

>G.ES.08.P2091_a.JN054265

SYHRLRDFIL

>G.ES.08.X2470_F8_12.GQ222685

SYHRLRDFIL

>G.ES.08.X2558.GQ862781

SYHRLRDLLL

>G.ES.08.X2571_b1.JF327808

SYHHLRDFTL

>G.ES.09.P2196_3s_nt0558_9495.MF157743

SYHRLRDFIL

>G.ES.09.X2634_2.GU362882

SYHHLRDFIL

>G.ES.09.X2636_b.JN054287

SYHRLRDFVL

>G.ES.09.X2693_a.JN054296

SYHRLRDFIL

>G.ES.14.ARP1201.KT276261

SYHRLRDFVL

>G.ES.14.EUR_0033.KU685592

SYHHLRDFIL

>G.FR.95.PHI355.AY231155

SYHPLRDFIL

>G.FR.95.PHI365.AY231156

SYHRLRDFIL

>G.FR.x.523.EF033660

SYHRLRDFIL

>G.GA.x.LBV217.U09664

SYHQLRDFIL

>G.GB.13.15171_1_17.3.MF109647

SYHRLRDFIL

>G.GB.14.14592_1_74.4.MF109568

CYHRLRDFIL

>G.GB.14.14667_1_38.4.MF109598

SYHRLRDLLL

>G.GH.03.03GH175G.AB287004

SYHRLRDFAL

>G.GW.08.LA57LmNe.KU168300

CYHRLRDFIL

>G.KE.06.06KE275457V6.KT022379

SYHRLRDFIL

>G.KE.09.DEMG09KE001.KF716477

LYHRLRDFIL

>G.KE.93.HH8793_12_1.AF061641

SYHRLRDFIL

>G.NG.01.01NGPL0669.DQ168576

SYHRLRDFIW

>G.NG.01.01NGPL0674.DQ168575

SYHLLRDFIL

>G.NG.01.01NGPL0760.DQ168579

SYHLLRDFIL

>G.NG.01.PL0567.DQ168573

SYHRLRDFTL

>G.NG.08.08NG_SC13.JN248582

SYHLLRDFIL

>G.NG.08.P1909_D2.HQ236584

SYHHLRDLVL

>G.NG.08.P1992_G10_4.GQ324613

SYHRLRDFIS

>G.NG.09.09NG010079.KX389636

SYHRLRDFIL

>G.NG.09.09NG010105.KX389635

LYHRLRDFIL

>G.NG.09.09NG010157.KX389631

SYHRLRDFIL

>G.NG.09.09NG010205.KX389628

SYHRLRDLVL

>G.NG.09.09NG010261.KX389626

SYHRLRDLLL

>G.NG.09.09NG010315.KX389625

SYXHLRDLIL

>G.NG.09.09NG_SC21.JN248584

LYHRLRDFVL

>G.NG.09.09NG_SC26.JN248586

IYHRLRDFVL

>G.NG.09.09NG_SC31.JN248591

SYHRLRDFIL

>G.NG.09.09NG_SC62.JN248593

SYHRLRDFIS

>G.NG.10.10NG020133.KX389620

SYRHLRDFIL

>G.NG.10.10NG020134.KX389619

LYHRLRDFAL

>G.NG.10.10NG020303.KX389618

CYHRLRDLIL

>G.NG.10.10NG020420.KX389615

SYHRLRDLIL

>G.NG.11.11NG050158.KX389641

SYHRLRDFVL

>G.NG.11.11NG050272.KX389642

SYHHLRDFIL

>G.NG.11.11NG050489.KX389645

SYHRLRDFIL

>G.NG.11.DEMG11NG006.KY953200

IYHRLRDLVL

>G.NG.11.DEMG11NG007.KY953201

SYHRLRDFVL

>G.NG.11.DEMG11NG008.KY953202

SYHRLRDFIS

>G.NG.12.12NG060248.KX389646

SYHRLRDFIL

>G.NG.12.12NG060409.KX389648

SYHRLRDLIS

>G.NG.92.92NG083_JV10832.U88826

SYHRLRDLVL

>G.NG.95.NG1928.AF069947

SYHRLRDFIL

>G.NG.95.NG1929.AF069943

SYRRLRDFIL

>G.NG.95.NG1937.AF069937

SYHRLRDFIS

>G.NG.95.NG1939.AF069935

SYHRLRDFIL

>G.PT.x.PT2695.AY612637

SYHHLRDFIL

>G.PT.x.PT3037.FR846408

SYHRLRDFIL

>G.PT.x.PT3306.FR846409

SYHRLRDFIL

>G.PT.x.PT988.FR846410

SYHHLRDFVL

>G.RU.12.RU_SRD_2012.MF614606

SYHRLRDFIL

>G.RU.89.RU_ShRMT_1989.MF614605

SYHRLRDCIL

>G.RU.x.RU570_revertant_passage_20.EF367231

SYRRLRDFIL

>G.SE.93.SE6165_G6165.AF061642

SYHRLRDSIL

>G.ZA.01.TV546.KJ948662

SYHRLRDLLS

>G.ZM.03.8B.PB.103.KY229519

SYHRLRDFIL

>G.ZM.03.8M.PB.4902.KY229535

SYHRLRDFIL

>G.x.03.LA24HoCe.KU168278

SYHRLTDLVS

>G.x.08.X2483_F9.HQ236614

SYHHLRDFIL

>G.x.08.X2486_F2.HQ236615

SYHRLRDFIL

>G.x.x.X0558_8_F3.HQ236606

SYHRLRDFVL

>H.BE.93.VI991.AF190127

SYRRLRDLLS

>H.BE.93.VI997.AF190128

SYRLLRDSLL

>H.CD.01.CG_0260_02V_NGSID16.KY392779

SYRLLRDFLL

>H.CD.01.CG_0536_02_NGSID14.KY392777

SYRHLRDLLL

>H.CD.01.CG_0538_02_NGSID15.KY392778

SYRLLRDSLL

>H.CD.04.LA19KoSa.KU168273

SYHHLRDLLL

>H.CF.02.LA25LeMi.KU168279

SYRLLRDLLL

>H.CF.90.056.AF005496

SYRLLRDLLL

>H.GB.00.00GBAC4001.FJ711703

SYRRLRDLLL

>J.CD.02.CG_0331_02V_NGSID13.KY392776

SYHRLRDCVL

>J.CD.03.LA26DiAn.KU168280

SYHRLRDFVL

>J.CD.97.J_97DC_KTB147.EF614151

SYHRLRDFVL

>J.CM.04.04CMU11421.GU237072

SYHRLRDFIL

>J.SE.94.SE9173_7022.AF082395

SYHRLRDFVL

>K.CD.87.P3844.MH705156

SYHHLRDLIL

>K.CD.97.97ZR_EQTB11.AJ249235

SYRHLRDLVL

>K.CM.96.96CM_MP535.AJ249239

SYRQLRNLIL

>01_AE.AF.07.569M.GQ477441

SYHRLRDFIL

>01_AE.BE.x.VI1888cl194.EU191617

SYHRLRDFIS

>01_AE.CF.90.90CF11697.AF197340

SYHRLRDFIL

>01_AE.CF.90.90CF4071.AF197341

SYHRLRDFIL

>01_AE.CF.90.90CR402_CAR_E_4002.U51188

SYHRLRDFIL

>01_AE.CH.x.ZEnv32_0111_5.KU600816

SYRRLRDLLS

>01_AE.CH.x.ZEnv92_1008_8.KU600817

SYHRLRDFIS

>01_AE.CM.11.1156_26.KP718930

SYHRLRDLLL

>01_AE.CM.93.CA10_3.EU191614

SYHRLRDFIL

>01_AE.CN.02.YN0203.JX112860

SYHRLRDLVS

>01_AE.CN.02.YN0221.JX112861

SYHRLRDLTL

>01_AE.CN.02.YN0229.JX112863

SYHRLRDLIL

>01_AE.CN.02.YN0236.JX112866

SYHRLRDFIL

>01_AE.CN.05.05GX014.GU564225

SYHRLRDFGL

>01_AE.CN.05.05GX034.GQ845124

SYHLLRDFIL

>01_AE.CN.05.05GX079.GQ845125

SYHLLRDFSL

>01_AE.CN.05.05GX128.GQ845126

SYHLLRDFSL

>01_AE.CN.05.05GX162.GU564229

SYHLLRDFSL

>01_AE.CN.05.FJ051.DQ859178

SYHLLRDFIS

>01_AE.CN.05.FJ053.DQ859179

SYHRLRDFIL

>01_AE.CN.05.Fj052.EF036528

SYRRLRDCIL

>01_AE.CN.05.Fj055.EF036527

SYRRLRDFIL

>01_AE.CN.05.Fj056.EF036529

SYHRLRDFIL

>01_AE.CN.05.Fj057.EF036530

SYRRLRDFIL

>01_AE.CN.05.Fj065.EF036534

SYHRLRDFIL

>01_AE.CN.05.Fj066.EF036535

SYHLLRDFTS

>01_AE.CN.06.AE02.EU363850

SYHLLRDFIL

>01_AE.CN.06.CNE3.HM215410

SYHRLRDLLL

>01_AE.CN.06.CNE59.HM215422

SYHRLRDLLS

>01_AE.CN.06.CNE5.HM215415

SYHRLRDFIS

>01_AE.CN.06.CNE8.HM215427

SYHRLRDLIL

>01_AE.CN.06.FJ054.DQ859180

SYRRLRDFIS

>01_AE.CN.06.Fj061.EF036536

SYRHLRDFVL

>01_AE.CN.06.Fj062.EF036531

SYHRLRDFIL

>01_AE.CN.06.Fj064.EF036533

SYHRLRDFIL

>01_AE.CN.06.YN192_31.GU475046

SYHRLRDFIL

>01_AE.CN.07.07CNYN312.KF835499

SYHRLRDFIL

>01_AE.CN.07.07CNYN315.KF835502

SYHRLRDFIL

>01_AE.CN.07.07CNYN316.KF835503

SYHRLRDFIL

>01_AE.CN.07.07CNYN317.KF835504

SYHRLRDFIL

>01_AE.CN.07.07CNYN318.KF835505

SYHRLRDLAL

>01_AE.CN.07.07CNYN326.KF835513

LYHRLSDLIL

>01_AE.CN.07.07CNYN327.KF835514

SYRLLRDFIL

>01_AE.CN.07.07CNYN329.KF835516

SYHHLRDFIL

>01_AE.CN.07.07CNYN332.KF835518

SYRLLRDFSL

>01_AE.CN.07.07CNYN333.KF835519

SYHRLRDFIL

>01_AE.CN.07.07CNYN337.KF835523

SYHRLRDLTL

>01_AE.CN.07.07CNYN342.KF835527

SYRRLKDFSL

>01_AE.CN.07.07CNYN343.KF835528

SYHRLRDCIL

>01_AE.CN.07.07CNYN354.KF835533

LYHRLRDFTL

>01_AE.CN.07.07CNYN357.KF835536

SYHRLRDLIL

>01_AE.CN.07.07CNYN359.KF835538

SYRRLRDFIL

>01_AE.CN.07.07CNYN364.KF835542

SYHRLRDFIL

>01_AE.CN.07.07CNYN366.KF835543

SYHRLRDFIL

>01_AE.CN.07.07JSWX045.FJ441290

SYHRLRDFIS

>01_AE.CN.07.BJ17A_6.GU475028

SYHRLRDLIL

>01_AE.CN.07.BJ3_4.GU475040

SYHRLRDFSL

>01_AE.CN.07.BJOX028000.e04.KM218078

SYHRLRDFSL

>01_AE.CN.07.BJX4_6.GU475020

SYRLLRDFSL

>01_AE.CN.07.CNE28.HM215409

LYHRLRDFIS

>01_AE.CN.07.CNE55.HM215418

SYHRLRDLLL

>01_AE.CN.07.CNE56.HM215419

SYHRLRDFIL

>01_AE.CN.07.CNE71_U.HQ699988

SYHRLRDLTL

>01_AE.CN.07.FJ070010.JX112809

SYHLLRDFSL

>01_AE.CN.07.FJ070013.JX112810

SYRLLRDFIL

>01_AE.CN.07.FJ070017.JX112811

SYRLLRDFSL

>01_AE.CN.07.FJ070033.JX112813

SYHRLRDFIL

>01_AE.CN.07.FJ070035.JX112814

SYHLLRDFSL

>01_AE.CN.07.FJ070040.JX112817

SYRLLRDFSL

>01_AE.CN.07.FJ070043.JX112818

SYHRLRDFVL

>01_AE.CN.07.GD070010.JX112819

SYHLLRDFSL

>01_AE.CN.07.GD070058.JX112820

SYHRLRDLLL

>01_AE.CN.07.GD070059.JX112821

SYHRLRDFIS

>01_AE.CN.07.GD070090.JX112823

LYRLLRDFSL

>01_AE.CN.07.GD070092.JX112824

SYHRLRDFSL

>01_AE.CN.07.GD070096.JX112825

SYHLLRDFSL

>01_AE.CN.07.GD070118.JX112826

SYHLLRDFSL

>01_AE.CN.07.GD070120.JX112827

SYHRLRDFIL

>01_AE.CN.07.GX070003.JX112829

SYHLLRDFSL

>01_AE.CN.07.GX070005.JX112830

LYHLLRDFIL

>01_AE.CN.07.GX070006.JX112831

SYHLLRDFSL

>01_AE.CN.07.GX070043.JX112832

SYHLLRDFSL

>01_AE.CN.07.GX070044.JX112833

SYHLLRGFIL

>01_AE.CN.07.GX070076.JX112834

SYHLLRDFSL

>01_AE.CN.07.GX070143.JX112835

SYHLLRDFSL

>01_AE.CN.07.GX070145.JX112836

SYHLLRDFSL

>01_AE.CN.07.GX070149.JX112837

SYHLLRDCSL

>01_AE.CN.07.GX070154.JX112838

SYHLLRDFSL

>01_AE.CN.07.GX070167.JX112839

SYHLLRDFSL

>01_AE.CN.07.GX11_13.GU475042

SYHRLRDFIL

>01_AE.CN.07.GX142_2.GU475031

SYHLLRDFSL

>01_AE.CN.07.GX155_55.GU475043

SYHLLRDFIL

>01_AE.CN.07.GX24_8.GU475015

LYHLLSDFIL

>01_AE.CN.07.GX25_29.GU475030

SYHLLRDFSL

>01_AE.CN.07.GZ070004.JX112840

LYHLLRDFSL

>01_AE.CN.07.GZ070123.JX112843

SYHRLRDLLL

>01_AE.CN.07.GZ070126.JX112844

SYHRLRDFIL

>01_AE.CN.07.GZ070127.JX112845

SYHLLRDFSL

>01_AE.CN.07.GZ187_10.GU475036

SYHRLRDFIL

>01_AE.CN.07.JS070901.JX112850

SYHLLRNSIL

>01_AE.CN.07.JS071001.JX112851

SYHLLRDFSL

>01_AE.CN.07.JS071101.JX112853

SYHRLRDFIS

>01_AE.CN.07.LN070008.JX112854

SYHRLRDFSL

>01_AE.CN.07.LN070013.JX112856

CYHRLRDFIL

>01_AE.CN.08.08LNA002.JX960612

SYHRLRDFSL

>01_AE.CN.08.08LNA003.JX960606

SYRRLRDFIL

>01_AE.CN.08.08LNA004.JX960617

SYHRLRDFGL

>01_AE.CN.08.BJ6_17.GU475029

SYHRLRDFIL

>01_AE.CN.08.GX35_33.GU475023

SYHRLRDFSL

>01_AE.CN.08.GX68_5.GU475044

LYHRLTDFVL

>01_AE.CN.08.GX73_29.GU475038

CYHRLRDFIL

>01_AE.CN.08.GX74_20.GU475021

SYHLLRDFTL

>01_AE.CN.08.GX83_47.GU475027

SYRLLRDFSL

>01_AE.CN.08.GX88_47.GU475018

SYHLLRDFIL

>01_AE.CN.08.GX8C_31.GU475039

LYHRLRDFTL

>01_AE.CN.08.GX90_1.GU475019

SYHRLRDFIL

>01_AE.CN.08.SH6_81.GU475032

SYHRLRDFIL

>01_AE.CN.08.SHX335_24.GU475033

SYHLLRDFIL

>01_AE.CN.08.SHX346_60.GU475034

SYHLLRDSIL

>01_AE.CN.09.09GX268.MG655196

SYRLLRDFGL

>01_AE.CN.09.09GX285.MG655197

SYHLLRNFIL

>01_AE.CN.09.09GX286.MG655198

SYHHLRDFIL

>01_AE.CN.09.09GX300.MG655199

SYHLLRDFIL

>01_AE.CN.09.09GX310.MG655200

SYHLLRDFSL

>01_AE.CN.09.09GX311.MG655201

CYHLLRNFIL

>01_AE.CN.09.09GX319.MG655202

SYHRLRDFIL

>01_AE.CN.09.09GX332.MG655204

SYRLLRDFTL

>01_AE.CN.09.09LNA005.JX960630

SYHRLRDFIL

>01_AE.CN.09.09LNA007.JX960627

LYHRLRDFIL

>01_AE.CN.09.09LNA013.JX960623

SYHRLRDFSL

>01_AE.CN.09.09LNA025.JX960634

SYHRLRDFSL

>01_AE.CN.09.09LNA040.JX960615

SYHRLRDFSL

>01_AE.CN.09.09LNA086.JX960639

SYHRLRDFIL

>01_AE.CN.09.09LNA136.JX960631

SYHRLRDFIL

>01_AE.CN.09.09LNA340.JX960607

SYHRLRDFIL

>01_AE.CN.09.09LNA353.JX960628

SYHRLRDFSL

>01_AE.CN.09.09LNA379.JX960603

SYHRLRDFIL

>01_AE.CN.09.10LNA016.JX960629

SYHRLRDFSL

>01_AE.CN.09.10LNA105.JX960626

SYHRLRDFIL

>01_AE.CN.09.1109.HQ215555

SYRRLRDLIL

>01_AE.CN.09.1119.HQ215553

SYHRLRDFSL

>01_AE.CN.09.YN09P0011.JX112867

SYHRLRDFSL

>01_AE.CN.09.YN09P0015.JX112868

SYHRLRDFIL

>01_AE.CN.09.ZK056.JX112870

SYHRLRDFIL

>01_AE.CN.10.10LNA057.JX960633

LYHHLRDFIL

>01_AE.CN.10.10LNA103.JX960608

SYHRLRDFIL

>01_AE.CN.10.10LNA124.JX960609

SYHRLRDLLL

>01_AE.CN.10.10LNA571.JX960611

SYRRLRDFTL

>01_AE.CN.10.10LNA821.JX960616

SYHRLRDFSL

>01_AE.CN.10.10LNA976.JX960632

SYHRLRDFSL

>01_AE.CN.10.CYM059.JX112796

SYHRLRDFIL

>01_AE.CN.10.CYM075.JX112797

SYHRLRDLLL

>01_AE.CN.10.CYM105.JX112798

LYHRLRDFIL

>01_AE.CN.10.CYM124.JX112799

SYHRLRDFIL

>01_AE.CN.10.CYM136.JX112800

SYHRLRDFIL

>01_AE.CN.10.CYM138.JX112801

SYRRLRDFIL

>01_AE.CN.10.CYM139.JX112802

SYHRLRDLIL

>01_AE.CN.10.CYM140.JX112803

SYHRLRDFIL

>01_AE.CN.10.CYM143.JX112804

SYRRLRDSIL

>01_AE.CN.10.CYM147.JX112805

SYHRLRDLTL

>01_AE.CN.10.CYM152.JX112807

SYHRLRDLIL

>01_AE.CN.10.CYM154.JX112808

SYRRLRDFIL

>01_AE.CN.10.DE00110CN007.KP109506

LYHRLRDFIS

>01_AE.CN.10.DE00110CN009.KP109507

SYHRLRDFSL

>01_AE.CN.10.JL100005.JX112846

SYHRLRDFIL

>01_AE.CN.10.JL100020.JX112849

SYHRLRDFSL

>01_AE.CN.10.YNFL03.KC870029

SYHLLRDFIS

>01_AE.CN.10.YNFL20.KC870039

SYHLLRDFIS

>01_AE.CN.10.YNFL23.KC870041

SYHLLRDFTS

>01_AE.CN.12.DE00112CN011.KP109508

SYHRLRDFIL

>01_AE.CN.15.15JS01.MG655205

SYHRLRDFIL

>01_AE.CN.15.15JS06.MG655206

SYRLLRDSSL

>01_AE.CN.15.15JS07.MG655207

SYHRLRDFIL

>01_AE.CN.15.15JS102.MG655208

SYHRLRDFTL

>01_AE.CN.15.15JS111.MG655209

SYHRLRDFVL

>01_AE.CN.15.15JS48.MG655211

SYHRLIDFIS

>01_AE.CN.15.15JS62.MG655212

SYHRLRDFIL

>01_AE.CN.15.15JS65.MG655213

LYRRLRDFIL

>01_AE.CN.15.15JS75.MG655214

SYHRLRDFVL

>01_AE.CN.15.15JS76.MG655215

SYHHLRDFIL

>01_AE.CN.15.15JS79.MG655216

SYHRLIDLLS

>01_AE.CN.15.15JS95.MG655217

SYHRLRDLIL

>01_AE.CN.15.15JS99.MG655218

SYHRLRDFTL

>01_AE.ES.09.X2721.JN054300

SYRRLRDFVL

>01_AE.GB.08.CH080100_e_p1.HM204634

LYHRLSDFIL

>01_AE.GB.10.Donor_N094_20_Month.KP873161

LYHRLRDFIL

>01_AE.GB.13.13592_1_16.3.MF109358

SYHRLRDFIL

>01_AE.GB.13.13774_1_46.3.MF109471

LYHRLRDLLL

>01_AE.GB.14.13612_1_24.3.MF109407

SYRRLRDFIS

>01_AE.GB.14.14727_1_55.3.MF109618

SYRHLRDLVL

>01_AE.GB.14.14727_1_60.3.MF109621

SYHLLRDFTL

>01_AE.HK.04.HK001.DQ234790

SYHRLRDFIL

>01_AE.IR.10.10IR.THR48F.AB703616

SYHRLKDLAL

>01_AE.JP.11.DE00111JP003.KF859741

SYHRLRDFIL

>01_AE.JP.x.DR0492.AB253423

SYHRLRDFIL

>01_AE.JP.x.DR2594.AB253668

LYHRLRDFIL

>01_AE.JP.x.DR6824.AB253427

SYHRLRDFIL

>01_AE.JP.x.JRC77AE.AB565504

SYHRLRDLLL

>01_AE.MM.14.fKSDU26.KU820849

SYHRLRDFTL

>01_AE.MM.99.mCSW105.AB097872

SYHRLRDFIL

>01_AE.PH.15.1008.MH327750

SYHRLRDFIL

>01_AE.PH.15.DE00115PH006.MH078560

SYRRLKDLVS

>01_AE.PH.15.DE00115PH012.KY658688

SYHRLRDFIL

>01_AE.PH.16.1010.MH327752

SYRHLRDFIL

>01_AE.PH.16.1024.MH327759

SYHRLRDLIL

>01_AE.PH.16.1025.MH327760

SYHRLRDFIL

>01_AE.PH.16.1027.MH327762

SYHRLRDLIL

>01_AE.PH.16.1029.MH327764

SYRRLRDFIL

>01_AE.PH.16.1031.MH327766

SYRRLRDFIL

>01_AE.PH.16.DE00116PH013.MH078562

SYHRLRDFIL

>01_AE.PH.17.1021.MH327756

SYHRLRDFIL

>01_AE.PH.17.1026.MH327761

SYRRLRDLLL

>01_AE.PH.17.1028.MH327763

SYRRLRDFIL

>01_AE.SE.09.034SE.MF373147

SYHRLRDFIL

>01_AE.SE.11.057SE.MF373160

SYHRLRDFVL

>01_AE.SE.11.SE601017.KP411840

SYTGLRDLIL

>01_AE.SE.11.SE601018.KP411841

CYHRLRDSIM

>01_AE.SE.11.SE601021.KP411842

SYHRLRDLIL

>01_AE.SE.12.069SE.MF373172

LYHHLRDFIL

>01_AE.SE.12.070SE.MF373173

SYHRLRDLVL

>01_AE.SG.08.FREE019.KY213720

SYHLLRNFIS

>01_AE.SG.08.HM019.KY213735

SYHRLRDFIL

>01_AE.SG.08.HM032.KY213733

CYHRLRDFIL

>01_AE.SG.08.HM033.KY213722

SYRRLRDFSL

>01_AE.SG.08.HM034.KY213736

SYHRLRDFGL

>01_AE.SG.08.HM038.KY213738

SYHRLRDFIL

>01_AE.SG.08.HM051.KY213723

SYHRLRDFIL

>01_AE.SG.08.HM069.KY213739

SYHRLRDFSL

>01_AE.SG.08.HM073.KY213719

SYHRLRDFIS

>01_AE.SG.08.HM086.KY213716

SYHRLRDFIL

>01_AE.SG.08.HM089.KY213724

SYHRLRDFIL

>01_AE.SG.08.HM097.KY213717

SYHRLRDFIL

>01_AE.SG.09.HM123D.KY213718

CYRRLRDLLL

>01_AE.SG.09.HM130.KY213715

SYHRLRDLIS

>01_AE.SG.09.HM140.KY213749

SYHRLRDLIL

>01_AE.SG.09.HM149.KY213734

SFHLLRDSTL

>01_AE.SG.09.HM153B.KY213750

SYHRLRDFIL

>01_AE.TH.00.00TH_C2101.AY945716

SYRRLRDLIL

>01_AE.TH.00.00TH_C3347.AY945721

SYHRLTDFIL

>01_AE.TH.00.00TH_C4118.AY945722

SYHRLRDFIL

>01_AE.TH.00.00TH_C4151.AY945724

SYHRLRDFIL

>01_AE.TH.00.00TH_C4382.AY945725

SYHRLRDFIL

>01_AE.TH.00.3019A06.C1.KJ952346

SYHRLRDFIL

>01_AE.TH.00.3043A03.B11.KJ952505

SYHRLRDFIL

>01_AE.TH.00.3046A02.B6.KJ952574

SYHRLRDLIL

>01_AE.TH.00.3063A10.B1.KJ952667

SYHRLRDLIS

>01_AE.TH.00.3090A02.C6.KJ952765

SYHRLRDFIL

>01_AE.TH.00.3104A06.C4.KJ952852

SYHRLRDFIL

>01_AE.TH.00.3111A02.A2.KJ952897

SYRRLRDLTL

>01_AE.TH.00.3118A16.A1.KJ952968

SYHRLRDLLL

>01_AE.TH.00.3131A12.C11.KJ953089

SYHRLRDLLL

>01_AE.TH.00.3135A10.A11.KJ953119

SYHRLRDFIL

>01_AE.TH.00.3153A14.A1.KJ953282

SYHRLRDFIS

>01_AE.TH.00.3189A08.C7.KJ953475

SYHRLRDFIL

>01_AE.TH.00.3203A14.A1.KJ953555

SYHRLTDFIS

>01_AE.TH.00.3218A16.C18.KJ953646

SYHRLRDFIL

>01_AE.TH.00.C1705.DQ789392

SYHRLRDLTS

>01_AE.TH.00.OUR595I.AY358052

SYHRLRDFIS

>01_AE.TH.00.OUR721I.AY358067

SYHRLRDFIL

>01_AE.TH.00.OUR724I.AY358060

SYRRLRDLTL

>01_AE.TH.00.OUR810I.AY358063

SYHRLRDLLL

>01_AE.TH.01.01TH_C1436.AY945713

SYRRLRDLIS

>01_AE.TH.01.01TH_C2570.AY945719

SYHRLRDFIL

>01_AE.TH.01.01TH_C3256.AY945720

SYHRLRDFIL

>01_AE.TH.01.LA09DuCe.KU168264

LYRRLRDFIS

>01_AE.TH.01.OUR414I.AY358050

SYHRLRDFIL

>01_AE.TH.01.OUR609I.AY358040

SYHRLRDLIS

>01_AE.TH.01.OUR642I.AY358041

SYRHLRDFIL

>01_AE.TH.01.OUR647I.AY358056

SYHRLRDFIS

>01_AE.TH.01.OUR674I.AY358038

SYHRLKDLAL

>01_AE.TH.01.OUR702I.AY358059

SYHRLTDFIS

>01_AE.TH.01.OUR788I.AY358068

SYHRLRDFLS

>01_AE.TH.01.OUR830I.AY358064

SYHRLRDFIL

>01_AE.TH.01.R2184_c04.JN944665

SYHRLRDFIL

>01_AE.TH.02.OUR769I.AY358062

SYHRLRDFIL

>01_AE.TH.03.TH7229.KU168309

SYHRLRDLVS

>01_AE.TH.04.04TH107542.JN248318

SYHRLRDLVL

>01_AE.TH.04.04TH328531.JN248324

SYHRLRDLLL

>01_AE.TH.04.04TH427990.JN248327

SYHHLRDFTL

>01_AE.TH.04.04TH505841.JN248328

SYHLLRDSIL

>01_AE.TH.04.04TH613543.JN248330

SYRRLRDLLL

>01_AE.TH.04.04TH807015.JN248334

SYRRLRDLLL

>01_AE.TH.04.04TH817196.JN248336

LYHRLRDFVL

>01_AE.TH.04.AA027a_wg4.JX447021

SYHRLRDFIY

>01_AE.TH.04.AA074a07R.JX447529

SYHRLRDFIL

>01_AE.TH.04.AA075a_WG7.JX447542

SYHRLRDFIL

>01_AE.TH.04.BKM.DQ314732

SYHRLRDFIL

>01_AE.TH.04.T256254_13.KC748982

SYHRLRDLVL

>01_AE.TH.04.T276248_sga01.JF297221

SYHRLRDFIL

>01_AE.TH.04.T500617_sga02.HQ691003

SYHRLRDLTS

>01_AE.TH.04.T502281_sga03.HQ691014

LYHRLRDLTL

>01_AE.TH.05.05TH130087.JN248339

SYHRLRDFIL

>01_AE.TH.05.05TH327568.JN248341

LYHRLRDLLL

>01_AE.TH.05.05TH342968.JN248342

SYHRLRDFIL

>01_AE.TH.05.05TH741452.JN248355

SYHHLRDFTL

>01_AE.TH.05.05TH841749.JN248356

SYHRLSDFIL

>01_AE.TH.05.356272_c02.JN944654

SYHRLRDFIS

>01_AE.TH.05.AA004a_wg4a.JX446712

SYHRLRDFIL

>01_AE.TH.05.AA023a13R.JX446977

LYHRLRDLLL

>01_AE.TH.05.AA029b08R.JX447048

SYHRLRDFIL

>01_AE.TH.05.AA033a_wg6a.JX447081

SYHRLRDFIL

>01_AE.TH.05.AA049a_WG13.JX447268

SYHLLRDFIS

>01_AE.TH.05.AA062b07R.JX447395

SYHRLRDFIS

>01_AE.TH.05.AA064a_WG2.JX447413

SYRRLRDFIS

>01_AE.TH.05.AA066a09R.JX447447

LYHRLKDFTL

>01_AE.TH.05.AA077a_RH10.JX447561

LYHRLRDFIL

>01_AE.TH.05.AA078a11R.JX447579

SYHRLRDFIL

>01_AE.TH.05.AA079a_WG4.JX447592

SYHRLRDLIL

>01_AE.TH.05.AA081a14.JX447619

SYHRLRDFIL

>01_AE.TH.05.AA094b09R.JX447816

SYHRLRDCIL

>01_AE.TH.05.AA097a09R.JX447855

SYHRLRDFIL

>01_AE.TH.05.AA101a_WG1.JX447936

SYHRLRDFSL

>01_AE.TH.05.AA103a06R.JX447958

SYHRLRDFIL

>01_AE.TH.05.AA107a_wg4.JX448022

SYHRLRDLLL

>01_AE.TH.05.AA118_703357_c02.JN944658

SYHRLRDFIL

>01_AE.TH.05.AA122a02R.JX448217

LYHRLRDFAL

>01_AE.TH.05.AA125a11R.JX448256

SYHRLRDLSL

>01_AE.TH.05.AA126a07R.JX448271

SYHRLRDFGL

>01_AE.TH.05.T293735_sga06.HQ690964

SYHRLRDLIS

>01_AE.TH.06.101PL1.EU743788

SYHRLRDFIL

>01_AE.TH.06.102CC2.EU743789

SYHRLRDFIL

>01_AE.TH.06.104PB4.EU743790

SYHRLRDLTL

>01_AE.TH.06.105PL2.EU743792

SYHRLRDLLL

>01_AE.TH.06.105PL3.EU743793

SYHRLRDLLL

>01_AE.TH.06.107CC2.EU743794

SYHRLRDLIS

>01_AE.TH.06.21PL2.EU743757

LYHRLRDFVS

>01_AE.TH.06.22PL1.EU743758

SYHRLRDFTL

>01_AE.TH.06.29CC1.EU743759

SYHRLRDFIS

>01_AE.TH.06.41PB3.EU743764

HYHLLRDFIL

>01_AE.TH.06.45PB1.EU743766

SYHRLRDFIL

>01_AE.TH.06.47PL1.EU743768

SYHRLRDFIL

>01_AE.TH.06.50PB2.EU743769

SYHRLRDFIS

>01_AE.TH.06.50PL1.EU743770

CYHRLRDLIL

>01_AE.TH.06.52PL7.EU743773

SYHRLRDFIL

>01_AE.TH.06.55PL1.EU743774

SYHRLRDFIS

>01_AE.TH.06.60PL2.EU743777

SYHLLRDFVL

>01_AE.TH.06.62PL1.EU743778

SYHRLRDFSL

>01_AE.TH.06.644039_c01b.JN944657

SYHRLRDFIL

>01_AE.TH.06.65CC4.EU743780

SYHRLRDFIL

>01_AE.TH.06.65PL1.EU743781

SYHRLRDFIL

>01_AE.TH.06.98CC2.EU743782

SYHRLRDFIL

>01_AE.TH.06.98PB2.EU743784

SYHRLRDFIL

>01_AE.TH.06.99PL2.EU743787

CYHLLRDFTL

>01_AE.TH.06.AA002a_WG1.JX446666

SYRLLRDFSS

>01_AE.TH.06.AA003b12R.JX446699

SYHRLRDFSL

>01_AE.TH.06.AA006a02.JX446736

SYHRLRDFIS

>01_AE.TH.06.AA014a01.JX446855

SYHRLRDFIS

>01_AE.TH.06.AA017a_wg1.JX446899

NYHRLRDFIS

>01_AE.TH.06.AA018a07R.JX446910

SYHRLRDFIS

>01_AE.TH.06.AA022a_RH2.JX446961

CYRRLRDFTL

>01_AE.TH.06.AA034a_wg2.JX447089

SYHRLRDFIL

>01_AE.TH.06.AA035a10R.JX447107

SYHRLRDFSL

>01_AE.TH.06.AA038a_WG3.JX447132

SYHRLRDFIW

>01_AE.TH.06.AA042a10R.JX447203

SYHRLRDFIL

>01_AE.TH.06.AA044a_RH2.JX447222

SYHRLRDFIS

>01_AE.TH.06.AA055a_WG4.JX447312

SYHRLRDFIL

>01_AE.TH.06.AA056a_WG5.JX447316

SYHRLRDLIL

>01_AE.TH.06.AA058a04R_434239.JX447346

SYHLLRDFSL

>01_AE.TH.06.AA059a_WG5.JX447356

LYHRLRDFTL

>01_AE.TH.06.AA063a_WG37.JX447409

CYHRLRDCIL

>01_AE.TH.06.AA068a_14.JX447465

SYHRLRDFIL

>01_AE.TH.06.AA073a_RH1.JX447515

LYHRLTDLIS

>01_AE.TH.06.AA076a03R.JX447550

SYHRLRDFIL

>01_AE.TH.06.AA082a_WG9.JX447646

SYHLLRDFIL

>01_AE.TH.06.AA083a08R.JX447657

SYHLLRDFIL

>01_AE.TH.06.AA085a_wg2.JX447682

SYRLLRDSIS

>01_AE.TH.06.AA086a06R.JX447699

SYHRLRDFIL

>01_AE.TH.06.AA088a_wg14.JX447712

LYHRLRDFTL

>01_AE.TH.06.AA099a_WG9.JX447891

LYHRLRDFIS

>01_AE.TH.06.AA104a_RH3.JX447984

SYHLLRDFIL

>01_AE.TH.06.AA109a01R.JX448039

RYHRLRDFIL

>01_AE.TH.06.AA116_427299_c12.JN944655

SYHRLRDFIL

>01_AE.TH.06.AA119_816763_c02.JN944659

SYHRLRDFIL

>01_AE.TH.06.AA121a04R.JX448198

SYHRLRDFIL

>01_AE.TH.06.AA123a04R.JX448238

SYHRLRDLIS

>01_AE.TH.06.AA127a02R.JX448279

LYHRLRDFTL

>01_AE.TH.06.AA129a03R.JX448292

SYHLLRDFIL

>01_AE.TH.06.AA130a07R.JX448301

SYHRLRDLIW

>01_AE.TH.06.T501602_sga01.JF297225

SYHRLRDFIL

>01_AE.TH.06.T614109_sga13.HQ691090

LYHRLRDFTL

>01_AE.TH.07.AA005a07R.JX446728

SYHRLRDLVL

>01_AE.TH.07.AA008a_RH10.JX446776

SYHRLRDFIL

>01_AE.TH.07.AA009b01R.JX446790

SYHRLRDFSL

>01_AE.TH.07.AA012a_RH12.JX446845

SYHRLRDFAL
[truncated: 90,011 more chars]
